# A genomic glance through the fog of plasticity and diversification in *Pocillopora*

**DOI:** 10.1038/s41598-017-06085-3

**Published:** 2017-07-20

**Authors:** Erika C. Johnston, Zac H. Forsman, Jean-François Flot, Sebastian Schmidt-Roach, Jorge H. Pinzón, Ingrid S. S. Knapp, Robert J. Toonen

**Affiliations:** 1 Hawai‘i Institute of Marine Biology, University of Hawai‘i at Mānoa, Kāne‘ohe, HI 96744 USA; 2 Université libre de Bruxelles (ULB), Avenue F.D. Roosevelt 50, B-1050 Bruxelles, Belgium; 3 Australian Institute of Marine Science, 4810 Townsville, Australia; 4 Carl von Ossietzky University of Oldenburg, 26129 Oldenburg, Germany; 5 Department of Psychiatry, UT Southwestern Medical Center, Dallas, TX USA

## Abstract

Scleractinian corals of the genus *Pocillopora* (Lamarck, 1816) are notoriously difficult to identify morphologically with considerable debate on the degree to which phenotypic plasticity, introgressive hybridization and incomplete lineage sorting obscure well-defined taxonomic lineages. Here, we used RAD-seq to resolve the phylogenetic relationships among seven species of *Pocillopora* represented by 15 coral holobiont metagenomic libraries. We found strong concordance between the coral holobiont datasets, reads that mapped to the *Pocillopora damicornis* (Linnaeus, 1758) transcriptome, nearly complete mitochondrial genomes, 430 unlinked high-quality SNPs shared across all *Pocillopora* taxa, and a conspecificity matrix of the holobiont dataset. These datasets also show strong concordance with previously published clustering of the mitochondrial clades based on the mtDNA open reading frame (ORF). We resolve seven clear monophyletic groups, with no evidence for introgressive hybridization among any but the most recently derived sister species. In contrast, ribosomal and histone datasets, which are most commonly used in coral phylogenies to date, were less informative and contradictory to these other datasets. These data indicate that extant *Pocillopora* species diversified from a common ancestral lineage within the last ~3 million years. Key to this evolutionary success story may be the high phenotypic plasticity exhibited by *Pocillopora* species.

## Introduction

Scleractinian corals within the genus *Pocillopora* (Lamarck, 1816) are among the most widely distributed and abundant reef building corals, found throughout the Pacific and Indian Oceans, and the Red Sea^1, 2^. Previous classifications of the genus based on morphology have been controversial due to high levels of inter- and intraspecific colony variation^3^ with more than 40 described species, of which only about 17 are generally accepted^4^. Recently, the genus has been the focus of many studies to delineate species boundaries using a variety of genetic markers^2, 5–13^. Of these genetic markers, the mitochondrial open reading frame (mtORF), a putative protein-coding region of unknown function^14^, has been one of the most informative, resulting in the current delimitation of five distinct mtORF clades^11^. Some of the mtORF haplotypes are highly isolated whereas others are geographically widespread^8, 10, 12, 13, 15^. Although they show some agreement with nuclear markers^6, 9^, micro-skeletal morphology^11, 12^, life history^16, 17^, and with geography^10^, they are less concordant with gross colony morphology^12, 13^, which may indicate that mtORF clades do not correspond to true species or that taxonomic relationships may be confused due to introgression^18^ and/or phenotypic plasticity^11, 19, 20^. To add complexity, *Pocillopora*, like other scleractinian reef-builders, are also considered holobionts: an assemblage of species that includes the host coral animal as well as symbiotically associated dinoflagellate algae (*Symbiodinium* Freudenthal, 1962), bacteria, viruses, archaea and protists^21^ that together form the ecological unit of a coral and are extracted along with the host genetic material.

To test these hypotheses, we generated RAD-seq data^22^ from 15 coral holobiont metagenomic libraries representing seven nominal species of *Pocillopora*. We then compared several datasets (1) mtDNA assemblies obtained by reference to the complete mitochondrial genome of *P. damicornis* (Linnaeus, 1758) (accession number: NC_009797^14^); (2) histone reference assemblies identified from *de-novo* contigs using BLAST^23^; (3) ribosomal contigs identified by reference to the 18 S, ITS1, 5.8 S, ITS2, and 28 S region of *P. damicornis* (accession number: AY722785^24^); (4) contigs that mapped to the coral transcriptomic data of Bhattacharya *et al*.^25^ and Traylor-Knowles *et al*.^26^; (5) all loci from the complete holobiont metagenomic libraries that passed filtering; and (6) holobiont single nucleotide polymorphism (SNP) loci of high quality that were shared by all *Pocillopora* taxa. Our objectives were to create a rooted phylogeny for the genus *Pocillopora* with age estimates for each node, and to determine if there was concordance among the various datasets.

## Results

### Holobiont and coral phylogenetic analyses

**Figure 1:**
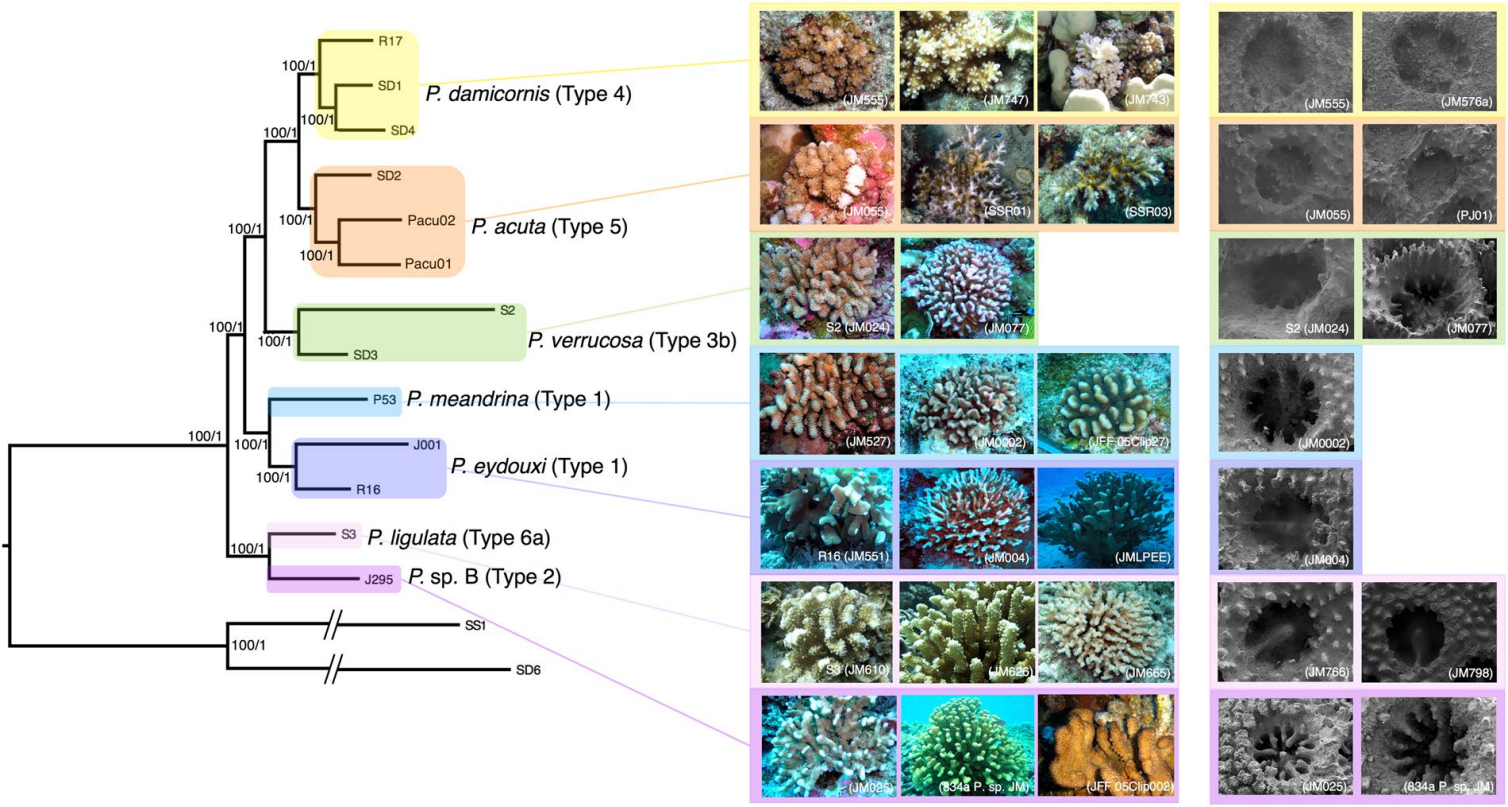
Maximum likelihood and Bayesian (ML/B) phylogenetic analysis of holobiont ezRAD data for 13 *Pocillopora* taxa where yellow: *Pocillopora damicornis*; orange: *P. acuta*; green: *P. verrucosa*; light blue: *P. meandrina*; dark blue: *P. eydouxi*; light purple: *P. ligulata*; dark purple: *P*. sp. B. and two outgroups, *Stylophora pistillata* (SD6), *Seriatopora hystrix* (SS1). Colony images provided by J. Maragos (JM), S. Schmidt-Roach (SSR), J-F. Flot (JFF). All SEM images were provided by Z. Forsman.

The holobiont and transcriptomic data yielded identical, well-resolved phylogenies with all five clades showing strong Bayesian posterior probability and maximum-likelihood bootstrap support (Figs 1 and S1; pp ≥ 95; bootstrap ≥70). The holobiont data and transcriptomic data contained libraries that consisted of approximately 25,000–330,000 and 530–7,000 contigs (Table S1), respectively, when clustered at a depth of 6 contigs in pyRAD v3.0.6^27^.

**Figure 2:**
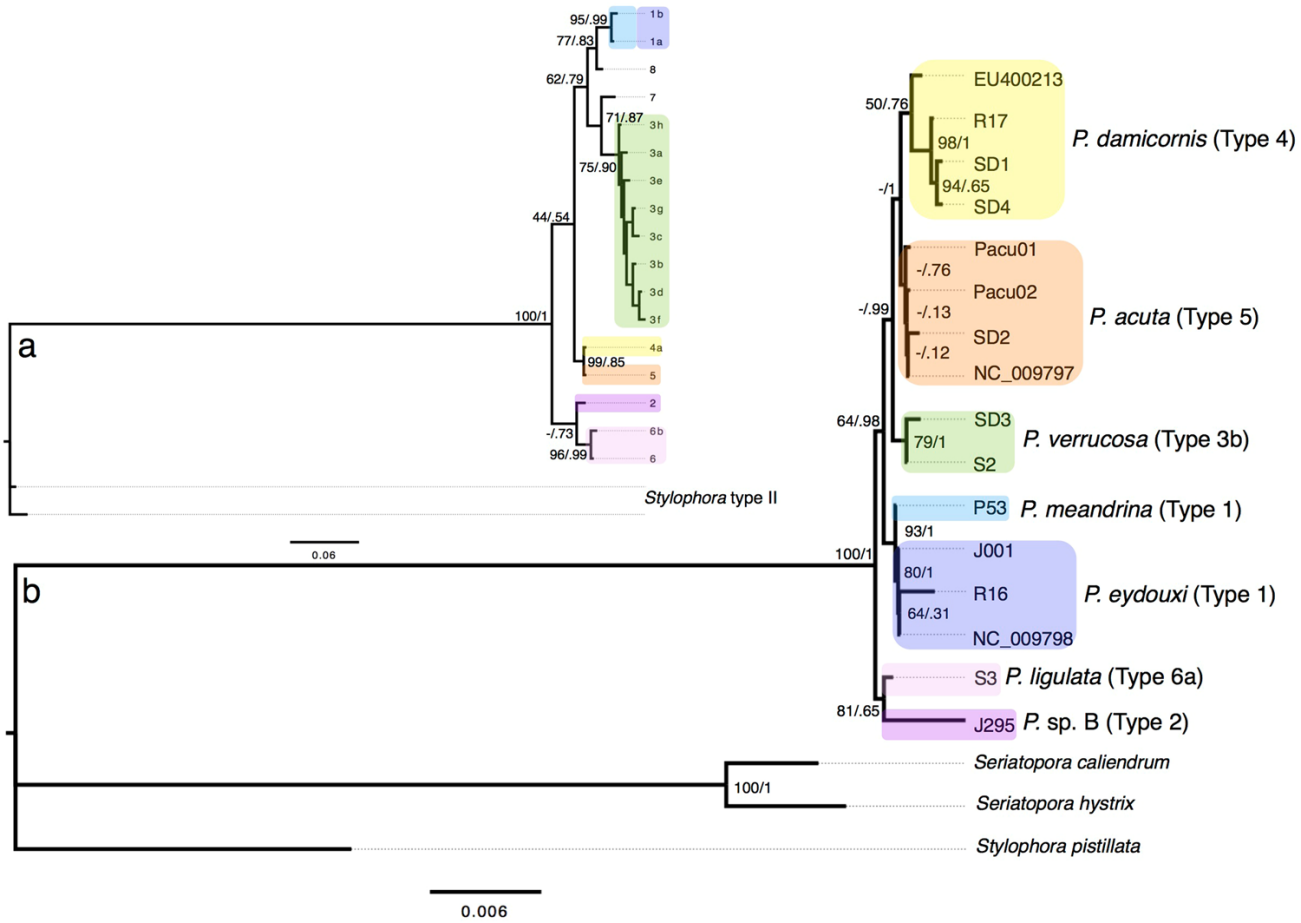
Phylogenetic analysis of mtORF and mitochondrial genomes. Reanalyzed data from (**a**) Pinzón *et al*.^2^: maximum likelihood and Bayesian (ML/B) phylogenetic analysis of the mtORF alignment of 382 bp. Tip labels indicate mtORF haplotype after Pinzón *et al*.^2^. (**b**) Maximum likelihood and Bayesian (ML/B) phylogenetic analysis of pocilloporid mitochondrial genomes (17,884 bp) (bottom). mtORF type designation after Pinzón *et al*.^2^ and species names after Schmidt-Roach *et al*.^11^ (Table 1). Scale bar represents substitutions per site. *Pocillopora* taxa are in yellow: *Pocillopora damicornis*; orange: *P. acuta*; green: *P. verrucosa*; light blue: *P. meandrina*; dark blue: *P. eydouxi*; light purple: *P. ligulata*; dark purple: *P*. sp. B. and three outgroups, *Stylophora pistillata*, *Seriatopora caliendrum and Seriatopora hystrix*.

The mitochondrial genome also showed high posterior support at clade nodes, yielding good support for the monophyly of each lineage (Fig. 2b). Bootstrap support in the maximum-likelihood phylogeny, however, was reduced for the three most recently diverged species (following the names proposed in the most recent formal taxonomical review of this genus by Schmidt-Roach *et al*.^11^), *Pocillopora verrucosa* (Ellis & Solander, 1786), *P. damicornis*, and *P. acuta* (Lamarck, 1816). Samples S2 and S3 had the greatest mean coverage depth across the mitochondrial genome (135.5 and 205.8, respectively), resulting in 100% coverage of the mitochondrial genome for sample S2 (Table S2). Coverage across the mitochondrial genome was most reduced for individuals in the *P. damicornis* clade (SD1, SD4, and R17: 44%, 35.1%, and 37.7% respectively).

Bayesian and maximum likelihood phylogenetic analysis of the histone dataset recovered a topology similar to that of the holobiont and transcriptomic data, however *P. acuta* was recovered as paraphyletic (Fig. S2b). Across the histone marker, individuals J295, SD2, and SD6 had the lowest percent coverage (30.3, 13.7, and 4.5%, respectively), and individuals S2,S3, and SD3 had the highest percent coverage (100, 95.3, and 96.1%, respectively) (Table S2). The tree topology without these low coverage samples is similar to that of the complete tree, and despite the low coverage of some individuals in the histone reference, the topology matches other approaches, so we included all data in these comparisons.

In contrast, posterior support for the topology recovered using the ribosomal region was generally low for the majority of nodes and the species *P. acuta* and *P. verrucosa* appear polyphyletic (Fig. S2a). Based on ribosomal genes alone, placement of individuals J001, R16, and J295 was most highly supported, whereas the placement of P53 was least supported (Fig. S2a). The positions of individuals P53, J001, and R16 based on ribosomal data (Fig. S2a) differed from that seen in the holobiont, mitochondrial, histone, and transcriptomic phylogenies (Figs 1, 2b, S2b and S1). Despite the relatively high percent coverage of the ribosomal reference for all individuals (avg. 88%, Table S2), this phylogeny (Fig. S2a) was the least well resolved and the most inconsistent with all other approaches reported here (Fig. S2b).

### SNP analyses

**Figure 3:**
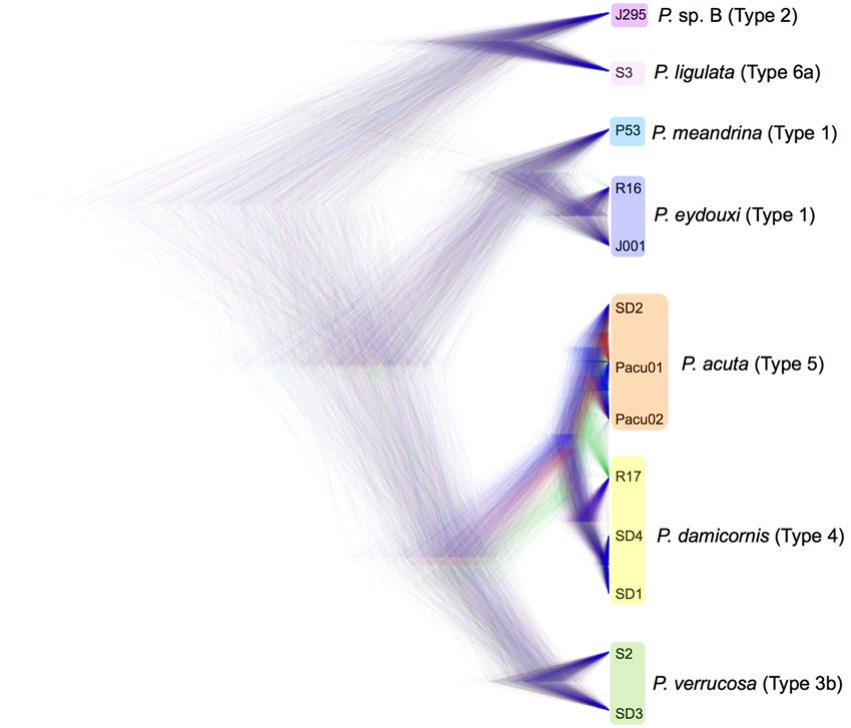
Species tree of *Pocillopora*. This cloudogram represents the posterior distribution of species trees from the Bayesian phylogenetic analysis program SNAPP using 430 unlinked, biallelic SNPs shared across all *Pocillopora* taxa. Higher density areas of the tree indicate greater topology agreement. The different colors represent differing topologies; trees with the highest clade credibility are shown in blue and trees represented in green and red represent trees with different topologies. Colored groups in the tree are labeled by the species name most often associated with this clade in the current literature (Table 1). Yellow: *Pocillopora damicornis*; orange: *P. acuta*; green: *P. verrucosa*; light blue: *P. meandrina*; dark blue: *P. eydouxi*; light purple: *P. ligulata*; dark purple: *P*. sp. B.

The SNAPP results, plotted into a cloudogram represent the underlying tree topology distributions^28^, showed clear divergence between lineages and well-resolved monophyly between all mtORF clades with the exception of the two most recently diverged sister species, *P. damicornis* and *P. acuta* (Fig. 3). The phylogenetic position of individuals Pacu02 and R17, in particular, shows evidence of alternative placement with some of the loci in this analysis, which was otherwise congruent with the holobiont, transcriptome, and mitochondrial analyses described above. Alternate trees emerging from the SNAPP analysis might derive from contamination by loci other than the coral host or can be evidence of introgressive hybridization, or incomplete lineage sorting among recently derived taxa^28^. By comparing results of different analyses, we can draw inferences about the likely mechanism driving alternate tree topologies in the SNAPP cloudogram: contamination ought to be distributed at random with respect to topology, whereas incomplete lineage sorting should be proportional to the time since the most recent ancestor, and introgression should be limited to species capable of hybridizing. The only alternate tree topologies common enough to appear in the cloudogram involve the most recently derived sister taxa: the *P. damicornis*/*acuta* complex (Fig. 3).

### Conspecificity matrix

**Figure 4:**
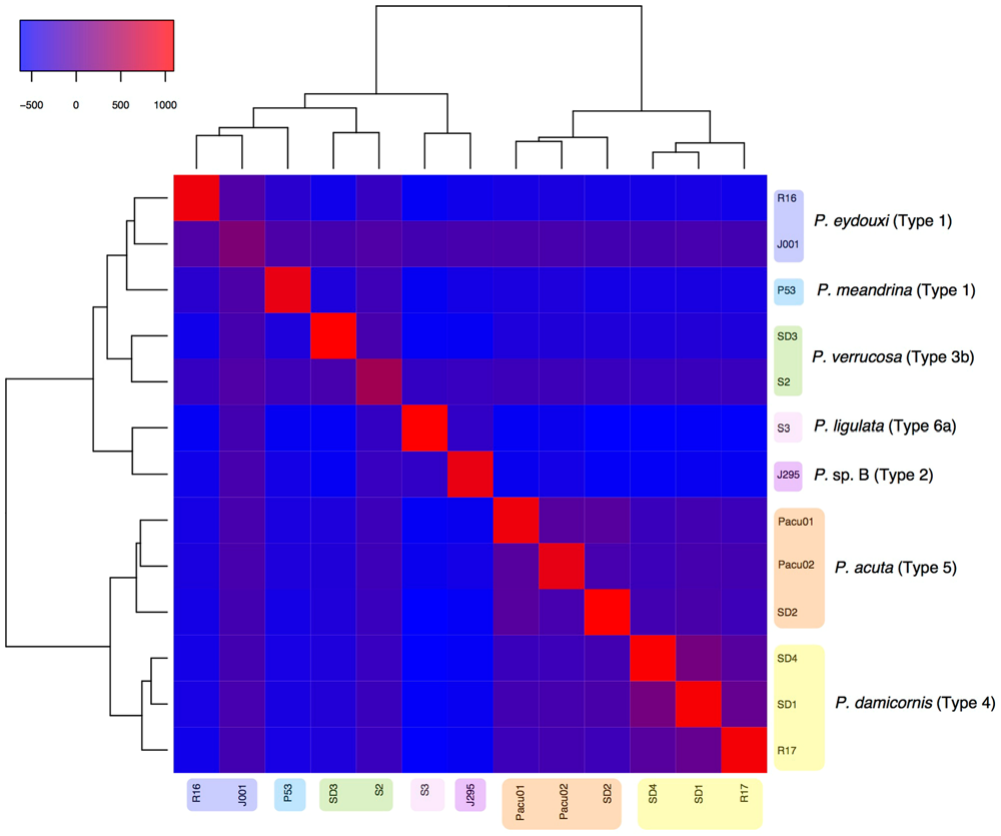
Conspecificity matrix obtained by calculating, for each pair of individuals, the number of RAD-seq markers supporting their conspecificity in haploweb analyses minus the number of markers supporting the contrary hypothesis (i.e., that they belong to different species). Two libraries had a lot of missing data (S2, J001) and had therefore a conspecificity score near zero with all individuals; still, clustering in the matrix placed each individual (including these two) in a position congruent with species delineations (colored boxes). Yellow: *Pocillopora damicornis*; orange: *P. acuta*; green: *P. verrucosa*; light blue: *P. meandrina*; dark blue: *P. eydouxi*; light purple: *P. ligulata*; dark purple: *P*. sp. B.

The groupings obtained from the conspecificity matrix approach^29^ were congruent with the mitochondrial, holobiont and transcriptomic phylogenies, and individuals of the same morphospecies clustered with high conspecificity scores (see colored boxes on the sides of Fig. 4). Conspecificity scores were high between sister species: *P. acuta* and *P. damicornis*, as well as between *P. ligulata* (Dana, 1846) and *P*. sp. B, and two libraries: *P. verrucosa* (S2) and *P. eydouxi* (J001), had a lot of missing data and therefore conspecificity scores near zero with all individuals. Conspecificity provides a sensitive test for introgression among taxa, and contrary to a previous study on bdelloid rotifers, where conspecificity signal away from the diagonal suggested considerable introgression between species^29^, here we find only individuals from closely related species present a high conspecificity signal (Fig. 4).

### Divergence time estimation

**Figure 5:**
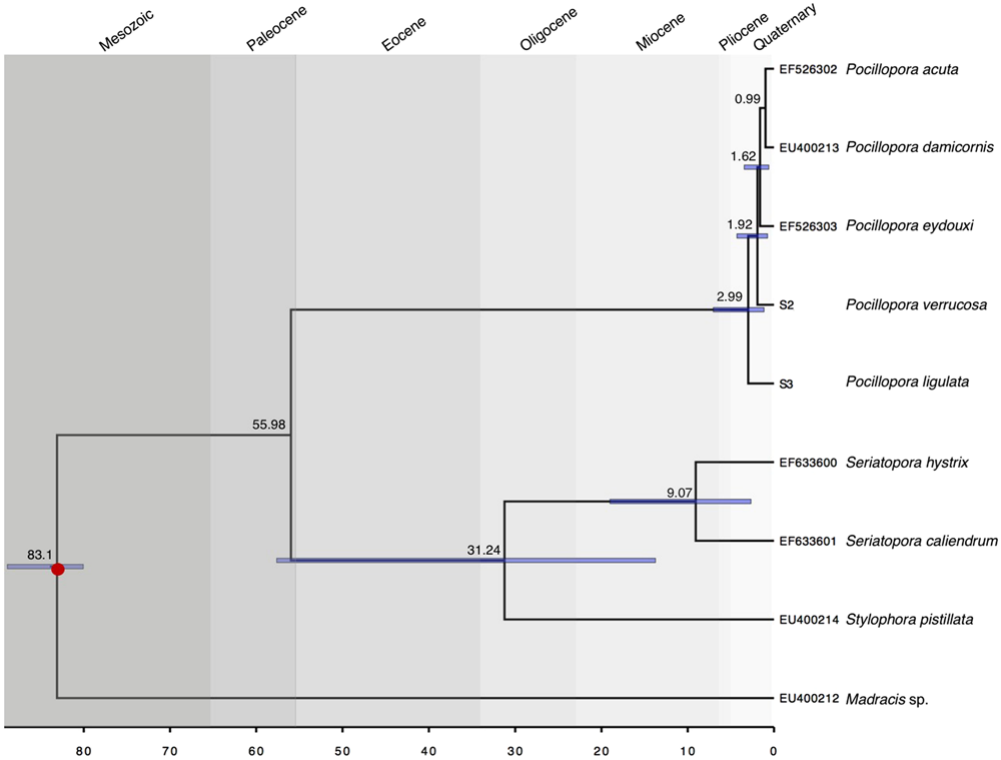
Reduced mitochondrial genome time-calibrated phylogeny consisting of protein coding regions (COX1: 1,549 bp; ND5 CDS: 12,937 bp; large ribosomal subunit: 1,972 bp; ATP8: 220 bp; total: 16,678 bp) of *Pocillopora* estimated using BEAST v2.3.2. The node represented with a red dot was time constrained by the first appearance of *Madracis* (*M. johnwellsi*) in the fossil record during the upper Campanian^30^ (72.1–83.6 mya). Values above nodes are median node ages and blue node bars represent the 95% highest posterior density interval of node ages.

Divergence among the genera *Madracis* (Milne Edwards & Haime, 1849), *Stylophora* (Schweigger, 1820), *Seriatopora* (Lamarck, 1816), and *Pocillopora* was inferred from the time-calibrated phylogeny generated from a reduced mitochondrial dataset (16,678 bp) anchored by the earliest occurrence of *Madracis* (*M. johnwellsi* (Frost and Langenheim 1974)) in the fossil record during the Campanian^30^ (83.6 MYA; Fig. 5). The median divergence time of *Pocillopora* from *Stylophora* was estimated to be ~55.98 Mya and *Seriatopora* from *Stylophora* ~31.24 Mya. The median estimates of the ages of *Pocillopora* species were as follows: *P. ligulata*: ~2.99 Mya; *P. verrucosa*: ~1.92 Mya; *P. eydouxi* (Milne Edwards, 1860): ~1.62 Mya; and *P. damicornis* and *P. acuta*: ~0.99 Mya (Fig. 5).

## Discussion

Here we show that a reduced representation (RAD) genomic approach generally supports previous work using the mtORF marker^2, 6, 7, 9–14^. A similar concordance was also observed between mtORF delimitations and the complete holobiont dataset, the mitochondrial genome data, and the data that mapped onto a published coral transcriptome^25, 26^.

Our phylogenetic and conspecificity matrix analyses supported the reciprocal monophyly among all species represented by the individuals in our dataset, with no evidence for introgressive hybridization among most species of *Pocillopora* except possibly the most recently derived sister species *P. damicornis* and *P. acuta*, which show evidence of potential hybridization or incomplete lineage sorting. Neither outcome would be surprising given their median divergence age of less than a million years, but further sampling and analyses are clearly needed to infer whether hybridization is occurring or if these species are still in the process of diverging. Here, we provide evidence for reciprocal monophyly among the majority of currently recognized *Pocillopora* species. Although our geographic sampling is not as exhaustive as some previous studies^2, 10, 13^, we include the extremes of the geographic and morphological range in the genus to show strong concordance among a variety of different approaches that together provide support for the mtORF marker as a species level marker. The exception to this generalization is that *P. meandrina* (Dana, 1846) and *P. eydouxi* share a common haplotype (mtORF type 1) and cannot be differentiated with this marker. Obviously additional sampling is needed both across the geographic ranges of the nominal taxa, and across the range of morphological variation seen within the genus to confirm species boundaries within *Pocillopora* and determine whether previously unrecognized narrow range endemics or cryptic species exist. However, given the striking monophyly of the taxa included here, we predict that future sampling will reveal low level genetic variation within valid nominal species, and do not expect to see evidence of frequent hybridization between any of these species.

Combosch and Vollmer^31^ reported a lack of monophyly between three morphospecies sampled from the Tropical Eastern Pacific (TEP) using RAD-seq, which contrasts with our findings. Although we do not have extensive sampling of TEP lineages, some of our seven species have broad geographic coverage that includes the TEP. There are three possible reasons for this inconsistency between studies. First, morphological misidentification to species is rampant in this genus^2, 9, 11, 12^, and it takes only a single misidentified individual in pooled samples to show mixed signal and bias the results toward introgression, therefore our RAD-seq libraries were all generated using PCR-free library preparation methods from individually barcoded individuals to eliminate this potential bias^22^. Second, Combosch and Vollmer^31^ used pools of individuals based on ORF and ITS2 types, with heterozygous ITS2 types considered as likely hybrids. Pooling small numbers of individuals into a single library may result in fewer individuals per pool than mean sequencing depth^32^ and PCR error and unequal representation of individuals in the pool can bias results^33, 34^. Another alternative may be that heterozygous ITS2 types provide an unreliable indication of hybrid origin. Consistent with this second alternative, our findings are in agreement with the reciprocal monophyly of mtORF types reported by Combosch and Vollmer^31^, but are not consistent with individuals possessing heterozygous ITS2 types being identified as likely hybrids. Further, our results indicate that relationships reconstructed from ribosomal genes are most at odds with the remainder of the dataset. Phylogenies based on morphology, mtDNA and ITS have often been at odds (e.g., Figs 1, 2 and S2a), which has resulted in controversy over interpretation of ITS data as resulting from hybridization, or from incomplete lineage sorting^31^. Our data offer insights to this long-standing debate and suggests that ribosomal DNA clades, although sometimes useful to delineate pocilloporid species (e.g. *P. ligulata* in Hawai’i or *Stylophora* sp. A and sp. B in Madagascar^35, 36^) should not to be trusted blindly when dealing with *Pocillopora* species. A third potential source of bias is that among anonymous RAD-seq libraries of the holobiont, non-coral loci (e.g., contamination of coral libraries from *Symbiodinium* or other commensal or ingested organisms) could be misinterpreted as shared genetic variation that provides misleading evidence for hybridization. Other RAD-seq methods result in short reads that are challenging to identify via BLAST, particularly in the absence of a reference genome^34^. ezRAD is unique in this regard because it allows assembly of long contiguous portions of the genome, up to complete mtDNA genomes^37–39^, that can then be grouped in different subsets: comparing the results obtained from each subset adds confidence to our findings if they are congruent, as was largely the case here.

*De novo* assembly of longer contigs allows us to ensure that some subsets of loci being analyzed originate from the coral host rather than from a symbiotic or prey contaminant. For example, comparing the subset of our loci that mapped with high confidence to transcribed genes of *P. damicornis* ^25, 26^ as compared to those that mapped to either of two *Symbiodinium* genomes^40, 41^ (see supplementary materials), allowed us to compare initial phylogenetic reconstruction based on holobiont metagenomics loci (the complete anonymous locus dataset) to subsets of the data that can be positively identified as either coral host or *Symbiodinium* loci. The concordance of each dataset, with the exception of the ribosomal and known symbiont loci, indicates that discordant information in these data is not positively misleading, and the biological signal is strong enough to withstand noise introduced by phylogenetically unrelated sequences.

Concordance between the holobiont, coral transcriptomic, coral mitochondrial, and coral SNP phylogenies presented here indicates strong support for reciprocal monophyly of each of these species, other than the most recently derived sister species. However, we cannot determine whether introgression or incomplete lineage sorting is responsible for blurring monophyly among these sister taxa for these datasets. Further, comparing the datasets (transcriptomic, mitochondrial genome, histone, ribosomal and SNPs) allows for an examination of consistency and reliability of phylogenetic reconstruction among the approaches and subsets of available data. Most of the datasets agree with previously reported mtORF designations (Fig. 2a) and provide strong support for the topology recovered by our overall holobiont dataset (Fig. 1). The most difficult species to resolve using the mtORF marker have been *P. meandrina* and *P. eydouxi*, which share the same mitochondrial haplotype (Table 1, type 1) but are distinct in microskeletal morphology^11^, and are also resolved in our phylogenetic analyses of the holobiont, transcriptomic, histone, ribosomal and SNP data, as well as by the conspecificity matrix approach (Figs 1, 3 and 4 and S1, 2). The striking outliers to general concordance of the species trees reconstructed among the datasets include: (1) the trees based on the *Symbiodinium* reads (but we have too few reads that map to the symbionts to place much confidence in these trees, Fig. S3), (2) the histone dataset (Fig. S2b), and (3) the ribosomal dataset (Fig. S2a), which each reveal some striking differences that likely explain some of the contradictory conclusions about species boundaries and hybridization reported in this group to date. For example, in our ribosomal dataset (Fig. S2a) we were unable to resolve *P. acuta* (mtORF type 5) and *P. verrucosa* (mtORF type 3), similar to two previous studies: Schmidt-Roach *et al*.^9^ who were unable to resolve *P. damicornis* and *P. verrucosa*; and Pinzón *et al*.^2^ who were unable to resolve *P. damicornis, P. verrucosa*, and a yet unnamed haplotype (mtORF type 7), using ITS2. Further study is needed to determine whether the discordance between the ribosomal genes most commonly used in phylogenetic studies are a peculiarity of this RAD-seq dataset or an inability to phase the nuclear genes in this approach with *Pocillopora*, or if this is an issue with corals in general^42–44^.

**Table 1:** Summary of the taxonomic names and genetic lineages for identification of *Pocillopora* used to date.

Veron and Pichon 1976^79^	Flot *et al*. 2008^6^, (Flot *et al*. 2010^7^)	Pinzón *et al*. 2011^8^, 2013^2^	Souter 2010^80^	Schmidt-Roach *et al*. 2012^16^, 2013^9,15^, 2014^11^	Marti-Puig *et al*. 2013^12^	Gélin *et al*. 2017^13^
*P. damicornis*	*P. damicornis* (a)	Type 4	n/a	*P. damicornis* (α)	1b	PSH 4
*P. damicornis*	*P. damicornis* (b)	Type 5	Type F	*P. acuta* (β)	1a	PSH 5
*P. damicornis*	n/a	n/a	n/a	*P. aliciae* (δ)	n/a	PSH 3
*P. verrucosa, P. damicornis*	(?) *P. molokensis*	Types 3, 7	Type NF	*P. verrucosa* (γ)	IIa	PSH 13, 14, 15, 16
n/a	n/a	n/a	n/a	*P. bairdi*	n/a	PSH 16
*P. eydouxi*	*P. eydouxi*	Type 1	n/a	*P. eydouxi*	IIb	PSH 9
*P. meandrina*	*P. meandrina*	Type 1	n/a	*P. meandrina*	IIb	PSH 9
n/a	n/a	n/a	n/a	*P*. cf. *brevicornis* (ε)	n/a	PSH 10
n/a	*P. ligulata*	Type 6	n/a	*P. ligulata* Dana, 1846	IIIb	PSH 2
n/a	*P*. sp. B	Type 2	n/a	*P*. cf. *effuses* Veron, 2000	IIIa	PSH 1
n/a	n/a	Type 8	n/a	*P*. sp. (unidentified)	n/a	PSH 6

*Pocillopora* corals are notorious for extreme phenotypic plasticity, and nearly continuous morphological transition from one morphospecies to another is common^11, 12, 19, 45^. Light and water movement are among the most important variables that induce morphological change in corals^46, 47^. For example, in the Gulf of California, five morphospecies of *Pocillopora* have been recorded^48^, however only mtORF type 1 (*P. meandrina* and *P. eydouxi*) has been documented to occur in that geographic region^8^. Additionally, Paz-García and colleagues recently documented colonies *in-situ* switching between three different morphospecies found in the Gulf of California (all mtORF type 1) resulting from shifts in environmental conditions in as little as six months^19^. Adding to these previous data, our results indicate that the high morphological diversity within *Pocillopora* is not a consequence of hybridization but is rather due to plasticity, as reported previously for the closely related genus *Stylophora* ^36^.

The exception to reciprocal monophyly among the seven species in our study was between the most recent sister species, which is expected given the young age of extant species (Fig. 5). Based on these data, the radiation that gave rise to extant *Pocillopora* species is estimated to have occurred less than 3 Myr ago. However, fossil evidence indicates that *Pocillopora* originated during the Eocene^49, 50^ (56-33.9 Mya), and was one of the dominant genera in the Caribbean during the Pliocene, and most of the Pleistocene^51^. By the middle of the Pleistocene however, there was only one Caribbean *Pocillopora* species remaining, *P. palmata* (Geister, 1977), which went extinct ~82,000 years ago^51, 52^. *Pocillopora* was rare in Indonesian Miocene assemblages^53^. Our age estimates for the radiation that gave rise to the extant members of this genus suggest that surviving Pacific *Pocillopora* likely experienced a bottleneck and subsequent rapid expansion during the Plio-Pleistocene. Major geological and climatic events between 4-2.5 Mya, such as the Northern Hemisphere glaciation, which brought with it strong glacial-interglacial cycles^54, 55^, a reduction in the El Niño effect^56^, and the closure of the Isthmus of Panama^57^, most likely had a strong impact on *Pocillopora* species, which appear to have undergone rapid speciation. In contrast to the clear species boundaries and reciprocal monophyly of *Pocillopora* reported here, a recent study on the sister genus *Stylophora*, which also underwent recent morphological diversification in the Red Sea during the same time, indicates that it remains a syngameon united by some gene flow^58^.

Today, *Pocillopora* species occur in 97.7% of the Indo-Pacific ecoregions^59^ and show high abundance in many reefs from low to high latitudes^1^. The wide geographic distribution of this genus, despite their relatively recent origin, suggests a rapid dispersal and establishment across the entire Indo-Pacific region within less than three million years. This evolutionary success story may be facilitated by their high phenotypic plasticity, which has been suggested for other organisms, to stimulate diversification by allowing adaptation to diverse conditions^60^.

## Conclusion

Our results indicate that species of *Pocillopora* are genetically distinct, but also highlight that morphological data must be supplemented with genetic data (mtORF at minimum) for accurate identification of species in this genus. The widely used mtORF marker shows promise as a species-level barcoding marker because it shows strong concordance with the reciprocal monophyly recovered in the holobiont, transcriptomic, mitochondrial, SNP, and conspecifity data. However limited resolution of this mitochondrial marker still leaves some taxa unresolved (e.g., *P. meandrina* and *P. eydouxi*) limiting its use as a universal barcode in the genus. The lack of evidence for introgressive hybridization between species here indicates that gross morphological plasticity is characteristic of *Pocillopora* species, and that caution should be used when interpreting poorly resolved gene trees from only a few genetic markers, particularly the commonly used ribosomal gene markers, which appear contradictory to other datasets in these analyses. Our fossil calibrated phylogeny further suggests that extant *Pocillopora* species are young (likely not older than ~3 Mya). This rooted phylogeny provides a template upon which ecological, demographic, life history, and population genetic questions may be further investigated to better understand the evolutionary processes that have shaped this widespread coral genus.

## Methods

### Taxon sampling

Tissue samples were collected from the Tropical East Pacific, Hawai’i, and Australia in 2013. The dataset includes 13 samples from the *Pocillopora* genus and two outgroup samples from closely related genera, *Stylophora pistillata* (Esper, 1797) and *Seriatopora hystrix* (Dana, 1846). All tissue samples were stored in either salt-saturated DMSO (dimethyl sulfoxide) buffer^61^ or >95% ethanol until DNA was extracted.

### DNA extraction and quantification

Genomic DNA was extracted from tissues using the OMEGA (BIO-TEK) E-Z 96 Tissue DNA Kit but instead of the 1 × 200 µl recommended elution, 2 × 100 µl were collected in HPLC grade H_2_O in order to capture higher molecular weight genomic DNA. HPLC grade water was used instead of the supplied buffer so the sample volume could be reduced, via a speed-vac, without concentrating the salts, which might interfere with downstream steps. Extractions were inspected on a 1% agarose gel, using TAE buffer, GelRed (Biotum, Inc) gel stain and the Bioline Hyperladder 1 (200–10,000 bp). Samples were considered acceptable if there was a high band or a smear with at least half of the sample above 2,500 bp. Extractions were quantified using the AccuBlueTM (Biotium, Inc.) High Sensitivity dsDNA quantification kit with 8 standards and measured using a Molecular Devices SpectraMax M2 microplate reader at λ_Ex_/λ_Em_ 485/530 nm.

### Library preparation

ezRAD libraries^22^ were generated following the protocol of Knapp *et al*. (2016). Briefly, all samples were adjusted to approximately 1 µg of DNA in 25 µl based on the AccuBlue microplate readings prior to digestion by either dilution or concentration via evaporation with a speed-vac at room temperature. Genomic DNA was digested using the isoschizomer restriction enzymes *Mbo*I and *Sau*3AI (New England BioLab), which both cleave at GATC recognition sites. Digestions were performed in 50 µl reactions consisting of: 18 µl HPLC grade water, 5 µl Cutsmart Buffer (provided with restriction enzyme), 1 µl *Mbo*I (10 units), 1 µl *Sau*3AI (10 units) and 25 µl dsDNA (~1 µg) with the following thermocycler profile: 37 °C for 3 hours, then 65 °C for 20 mins. All digested samples were then cleaned using Beckman Coulter Agencourt AMPure XP purification beads at a 1:1.8 (DNA:beads) ratio following the standard protocol. The digests were run on a 1% agarose gel (as above) and were considered fully digested when there was a smear with little to no DNA above 5,000 bp.

### Illumina sequencing

All libraries were generated following the Illumina TruSeq Sample Prep v2 Low Throughput protocol. All libraries were size selected at 300–500 bp and passed through quality control steps (bioanalyzer and qPCR) and sequenced at the Hawai’i Institute of Marine Biology (HIMB) Genetics Core Facility (GCF). With the exception of libraries S2 and S3, which were sequenced on the MiSeq platform, all libraries were sequenced as paired-end 100 bp runs on the Genome Analyzer IIX system (GAIIx, Illumina, Inc.).

### Reference assemblies

Raw Illumina reads were sorted by barcode and lists of paired reads were trimmed on both the 5′ and 3′ ends for the adapter sequences using TRIM GALORE! (Andrews 2010). A PHRED score of 20 was used for all libraries. Both paired and unpaired reads were kept but reads <99 bp in length were discarded. Paired reads were validated and then merged using PEAR v0.9.6 with default settings^62^. Merged and non-overlapping reads were concatenated into a single file for each library for the ‘holobiont’ dataset. Below we describe how subsets of these reads were gathered into the ‘transcriptome’ and ‘symbiont’ datasets.

### Consensus sequence comparisons

To generate consensus sequences for the holobiont dataset, each sample was clustered using pyRAD v3.0.63^27^ with the following parameters: (6) restriction overhang = GATC, (8) Mindepth = 6, (9) NQual = 4, (10) clustering threshold = 0.85, (11) Datatype = gbs, (12) MinCov = 2, (13) MaxSH = 3, (26) maxSNPs = 20, (29) trim overhang = 2,2 (31) maj. base call = 2. Outgroups used were SD6 (*Stylophora pistillata*) and SS1 (*Seriatopora hystrix*).

To generate the transcriptome dataset holobiont libraries were mapped to the *Pocillopora damicornis* transcriptome, which consists of 29,875 contigs^25^, using BWA v0.7.12^63^ with the MEM algorithm for single reads and default parameters (with the exception of restricting the output to only map scores of 10 and higher). SAM files were converted to BAM files using SAMTOOLS^64^ and BAM files were converted to FastQ files using BEDTools^65^. Consensus sequences were generated by clustering in pyRAD using the same parameters as were used for the holobiont libraries.

Library S2 was one of the highest quality libraries and was selected for *de-novo* assemblies. Assemblies were conducted using the GENEIOUS v 8.1.4 assembler with the *de-novo* low sensitivity/fast settings. Contigs >200 bp were compared against a local version of the National Center for Biological Information (NCBI) GenBank nt database which was downloaded on 4/13/2015 using the Basic Local Alignment Search Tool (BLAST) Megablast program^23^ to identify loci and to avoid contigs that may arise from assembly artifacts, or chimeric assemblies from multiple portions of the coral holobiont. The contigs were sorted by e-scores and the consensus sequence of one particularly long contig with high coverage and long blast hits (a contig blasting to coral histone proteins 2, 3, and 4; 4,519 bp) was selected to serve as a reference sequence. All libraries were assembled to this reference sequence using the default parameters (high sensitivity iterated up to five times and the medium/read mapping settings) in GENEIOUS v8.1.4. Consensus sequences were made from each library (not including the reference sequence) using the 75% majority option and N’s were called if coverage was 2X or less. Multiple sequence alignments were constructed using MUSCLE^66^ with 8 iterations. All libraries were also assembled to the *Pocillopora damicornis* mitochondrial genome (accession number: NC_009797^14^) using the same default settings as above. The entire mitochondrial genomes of *S. pistillata* (accession number: EU400214^67^), *S. hystrix* (accession number: NC_010244^67^), *Seriatopora caliendrum* (Ehrenberg, 1834) (accession number: NC_010245^67^), *Pocillopora eydouxi* (accession number: NC_009798^14^), *P. damicornis* (accession number: NC_009797^14^), and *P. damicornis* (accession number: EU400213^67^), along with the mitochondrial consensus sequences generated per library, were aligned (17,884 bp in length) as described above. All libraries were also mapped to a partial sequence of the ribosomal 18 S, ITS1, 5.8 S, ITS2, and 28 S region (1,399 bp) of *P. damicornis* (accession number: AY722785; Chen *et al*. unpublished data) using the same settings above. The mtORF type assignments were confirmed for each library by mapping the mitochondrial genome consensus sequences against reference mtORF sequences of each type^2^ in GENEIOUS v 8.1.4.

### Phylogenetic analyses

Phylogenetic trees were computed using EXABAYES v1.4.1^68^ and RAxML 8.1.15^69^ for the complete holobiont dataset and reads that mapped either to the transcriptome, the mitochondrial genome, the histone marker, or the ribosomal region. With EXABAYES v1.4.1 default parameters were used for both the holobiont and transcriptomic data, and default parameters were used for the mitochondrial, histone, and ribosomal data with the exception that 10,000,000 generations were sampled. By default, EXABAYES v1.4.1 applies the GTR model for nucleotide evolution with 1,000,000 generations, sampling frequency of 500, and a burn-in of 2,000 generations. Final trees were produced using CONSENSE in EXABAYES v1.4.1, which generates a consensus of all sampled trees after burn-in. Trees were visualized in FigTree v1.4.2 (http://tree.bio.ed.ac.uk/software/figtree/). For all of our Maximum Likelihood analyses (RAxML 8.1.15^69^) we used the GTRGAMMA model of nucleotide evolution and conducted a rapid bootstrap analysis and search for the best scoring tree in a single run (-f a). For our holobiont and transcriptomic data we used 100 rapid bootstrap replicates to estimate clade support, for the mitochondrial genomes we used 1000 rapid bootstrap replicates to estimate clade support, and for the histone and ribosomal data we used 10,000 bootstrap replicates to estimate clade support. Trees were visualized in FigTree v1.4.2.

### SNAPP Analysis

Species trees were estimated from single nucleotide polymorphism (SNP) data drawn from the holobiont dataset, which was analyzed using the SNAPP package in BEAST2^28^. To generate the unlinked, biallelic SNPs, required by SNAPP, we used contigs of length 140bp-300bp of the library, S2, to generate a reference against which the all other libraries were aligned. This reference was generated by dereplicating contigs using Rainbow ^70^ and clustering contigs using VSEARCH (–cluster_smallmem). As this process outputs both consensus and centroid sequences, we extracted the consensus sequences and indexed them to use as a reference building with SAMTOOLS^64^ and BWA v0.7.12^63^. Each holobiont library was then mapped to this reference using BWA v0.7.12^63^ with settings described above. The resulting SAM files were converted to BAM format using SAMTOOLS^64^, and read group information was added using PICARD (http://broadinstitute.github.io/picard/). The GENOME ANALYSIS TOOLKIT (GATK)^71^ was used to re-align around indels. Following dDocent^72^, we used FREEBAYES^73^ to call variants (−0 -E 3 -G 5 -z.1 -X -u -n 4 -! 10–min-repeat-entropy 1 -V –b) and filtered our VCF file to remove indels and extract unlinked, biallelic SNPs using VCFTOOLS^74^ (–min-meanDP 3–remove-indels–thin 300–remove-indv SD6–remove-indv SS1–max-missing 1). Both outgroups were removed for this analysis to allow more SNPs to be recovered within the ingroup, which resulted in 430 high quality informative SNPs shared across all *Pocillopora* libraries. The VCF file was converted to binary nexus format using PGDSpider v.2.0.9.1^75^. In BEAUti all taxa were treated as distinct species and the priors, *u* and *v*, were calculated from the data. In BEAST 2.3.2 the MCMC chain was run for 3,000,000 generations, sampling every 1,000 generations^28^. Convergence was assessed in TRACER^76^ and the first 10% was removed as burn-in.

### Conspecificity matrix

The ‘holobiont’ libraries were clustered using pyRAD v3.0.63^27^ with the following parameters: (6) restriction overhang = GATC, (8) Mindepth = 6, (9) NQual = 4, (10) clustering threshold = 0.85, (11) Datatype = gbs, (12) MinCov = 11, (13) MaxSH = 3, (26) maxSNPs = 20, (29) trim overhang = 2,2 (31) maj. base call = 2. The minimum coverage per locus was set to 11 libraries, in comparison to pyRAD analyses above where MinCov = 2, to recover a dataset with <15% missing data. The alignment of phased haplotypes for each locus (pyRAD output file *.alleles) were then converted into FASTA alignment. Species delimitation was performed for each locus using the haploweb approach^7^, and the species delineations gathered across all loci were condensed into one conspecificity matrix as in Debortoli *et al*.^29^: briefly, for each pair of individuals in the dataset we computed a conspecificity score equal to the number of loci supporting their conspecificity minus the number of loci supporting their heterospecificity. The resulting matrix (akin to a similarity matrix) was subsequently clustered using the R package “heatmap3”^77^ to reveal groups of individuals sharing common pools of alleles for many loci.

### Divergence time estimation

Mitochondrial genomes of the species *Madracis sp.* (Locke, Weil and Coates, 2007; formerly *M. mirabilis*) (accession number: EU400212^67^), *Stylophora pistillata* (EU400214^67^), *Seriatopora caliendrum* (EF633601^67^), *S. hystrix* (EF633600^67^), *Pocillopora ligulata* (Dana, 1846) (S3, this study), *P. verrucosa* (S2, this study), *P. eydouxi* (EF526303^14^), *P. damicornis* (EU400213^67^), and *P. acuta* (EF526302^14^) were aligned in GENEIOUS v8.1.4 using MUSCLE^66^ with five iterations and were then checked manually. From this alignment, four mitochondrial gene regions (COX1: 1,549 bp; ND5 CDS: 12,937 bp; large ribosomal subunit: 1,972 bp; ATP8: 220 bp) were extracted and the appropriate model of nucleotide evolution was determined to be GTR for each region based on AICc scores using jModeltest 2.1.4^78^. For divergence estimates we used the gamma site model, with gamma category count set to 4, the relaxed clock log normal, and the birth death model in BEAST 2.3.2^28^. We constrained the age of the *Madracis* node to the Lower Campanian (83.6 MYA) using the gamma prior (Alpha = 2.0, Beta = 2.0, Offset = 80.0) based on the fossil record of *Madracis johnwellsi*, which first appears in Tibet during this time^30^. The Markov Chain Monte Carlo was run for 40,000,000 generations, storing every 1000 generations. Convergence and mixing were checked using Tracer v1.6^76^, then 10% of trees were discarded as burn-in, and the maximum clade credibility tree with median node heights was generated with TreeAnnotator v.2.3.2^28^.

### Data Availability

Raw genetic data is available through the short read archive at NCBI, BioProject PRJNA386062, and final DNA alignments of the following are available as Supplementary Material:

- Mitochondrial genome alignments
- Histone alignment
- rDNA alignment

## Electronic supplementary material

Supplementary materials
