# Supplementary material for: A genomic glance through the fog of plasticity and diversification in Pocillopora
Source: Sci Rep. 2017 Jul 20;7:5991. doi: 10.1038/s41598-017-06085-3 (PMC5519588; doi:10.1038/s41598-017-06085-3)
Supplement: Supplementary file 1 — Supplementary materials [file 41598_2017_6085_MOESM1_ESM.doc]

**Supplementary Materials**

**A genomic glance through the fog of plasticity and diversification in *Pocillopora***

Erika C. Johnston^1*^, Zac H. Forsman^1^, Jean-François Flot^2^, Sebastian Schmidt-Roach^3^, Jorge H. Pinzón^4^, Ingrid S.S. Knapp^1^, Robert J. Toonen^1^

^1^Hawaiʻi Institute of Marine Biology, University of Hawaiʻi at Mānoa, Kāneʻohe, HI 96744, USA

^2^Université libre de Bruxelles (ULB), Avenue F.D. Roosevelt 50, B-1050 Bruxelles, Belgium

^3^Australian Institute of Marine Science, 4810 Townsville, Australia and

Carl von Ossietzky University of Oldenburg, 26129 Oldenburg, Germany

^4^Department of Psychiatry, UT Southwestern Medical Center, Dallas, TX USA

*Symbiont phylogenetic analysis*

The genomes of *Symbiodinium kawagutii*^40^ and *Symbiodinium minutum*^41^ were concatenated into a single file in GENEIOUS v 8.1.4 and the holobiont libraries were mapped to this reference using BWA v0.7.12^63^ with the MEM algorithm for single reads and default parameters (with the exception of restricting the output to only map scores of 10 and higher). SAM files were converted to BAM files using SAMTOOLS^64^ and BAM files were converted to FastQ files using BEDTools^65^. Consensus sequences were generated by clustering in pyRAD using the same parameters as were used for the holobiont libraries: (6) restriction overhang = GATC, (8) Mindepth = 6, (9) NQual = 4, (10) clustering threshold = 0.85, (11) Datatype = gbs, (12) MinCov = 2, (13) MaxSH = 3, (26) maxSNPs = 20, (29) trim overhang = 2,2 (31) maj. base call = 2. Outgroups used were SD6 (*Stylophora pistillata*) and SS1 (*Seriatopora hystrix*).

Phylogenetic trees were computed using EXABAYES v1.4.1^70^ and RAxML 8.1.15^69^. In our Bayesian analysis, we used default parameters with the exception that 10,000,000 generations were sampled. Final trees were produced using CONSENSE in EXABAYES v1.4.1^68^. In our Maximum Likelihood analysis (RAxML 8.1.15^69^) we used the GTRGAMMA model of nucleotide evolution and conducted a rapid bootstrap analysis and search for the best scoring tree in a single run (-f a) with 10,000 bootstrap replicates. Trees were visualized in FigTree v1.4.2 (http://tree.bio.ed.ac.uk/software/figtree/).

*Results*

Coverage across the concatenated *Symbiodinium* genomes was not consistent. Approximately 400 – 30,000 contigs from the holobiont libraries mapped to the concatenated reference of the *S. kawagutii* and *S. minutum* genomes (Table S1). This phylogeny showed no pattern of clade support consistent with that of any of the other phylogenies generated and has low support for the majority of nodes (Fig. S3).

*Discussion*

We find complete discordance with our symbiont dataset and our host phylogenetic analyses, indicating either that phylogenetic signal was too weak to detect genetic structure (only avg. 5,359 loci were sampled with avg. 310 reads per library; Table S1), or that the *Symbiodinium* communities associated with *Pocillopora* do not show a strong pattern of genetic structure among host species (Fig. S3). For the contigs that did map to the concatenated reference of the *S. minutum* and *S. kawagutii* genomes, we recovered very low coverage (Table S1) and disagreement between the coral host and these datasets may instead be a reflection of this extremely low coverage.


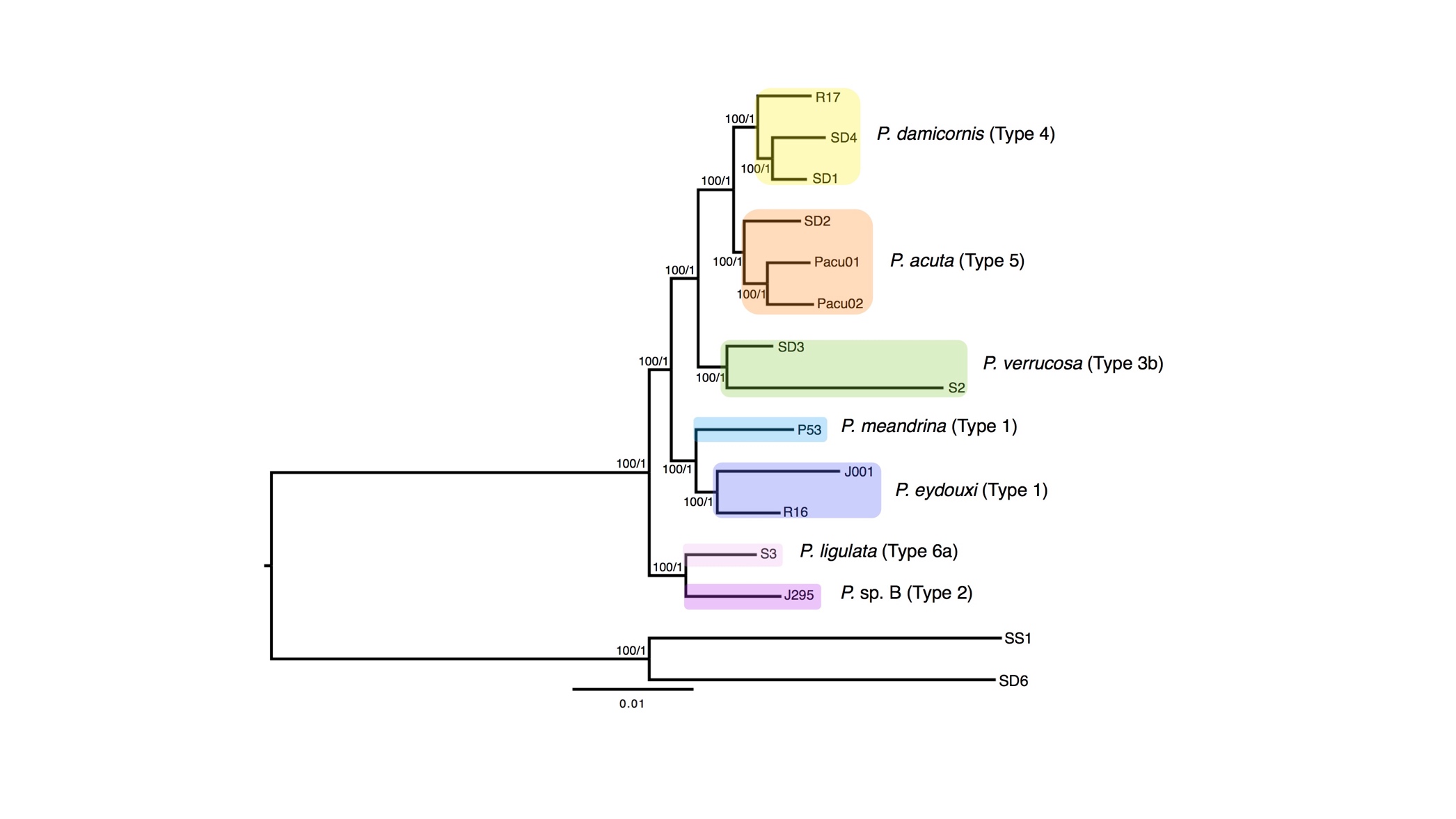


**Figure S1**. Maximum likelihood and Bayesian (ML/B) phylogenetic analysis of contigs that mapped to the *P. damicornis* transcriptome^23,24^ for 13 *Pocillopora* taxa (yellow: *Pocillopora damicornis*; orange: *P. acuta*; green: *P. verrucosa*; light blue: *P. meandrina*; dark blue: *P. eydouxi*; light purple: *P. ligulata*; dark purple: *P.* sp. B.) and two outgroups (*Stylophora pistillata* and (SD6) *Seriatopora hystrix* (SS1)).


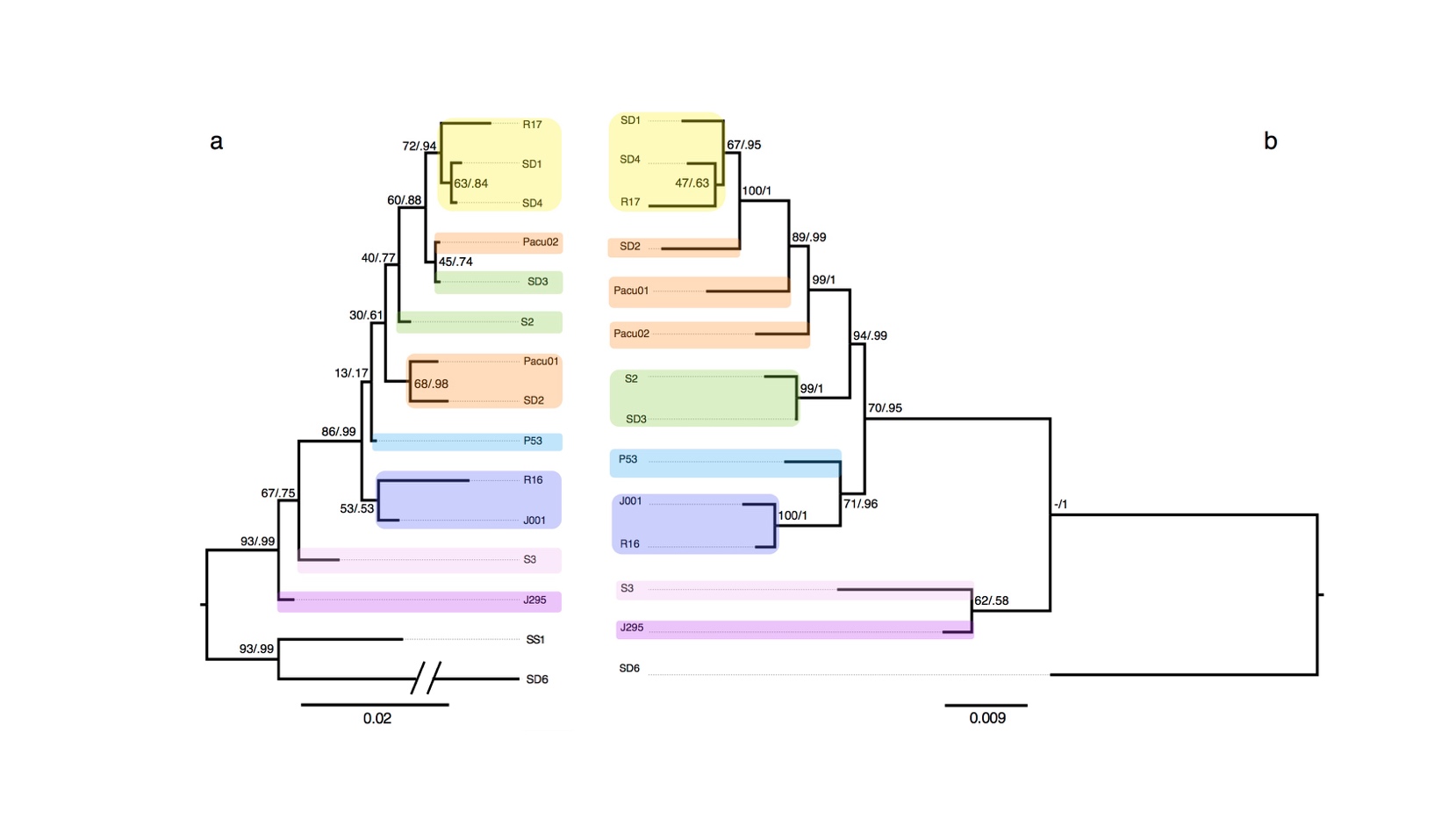


**Figure S2**. Maximum likelihood and Bayesian (ML/B) phylogenetic analysis of the **a)** ribosomal reference (partial sequence of ribosomal 18s, ITS1, 5.8s, ITS2, and 28s regions (1,399bp)) and **b)** histone reference (4,519bp) for *Pocillopora* taxa where yellow: *P. damicornis*; orange: *P. acuta*; green: *P. verrucosa*; light blue: *P. meandrina*; dark blue: *P. eydouxi*; light purple: *P. ligulata*; dark purple: *P.* sp. B. and two outgroups, *Stylophora pistillata* (SD6), *Seriatopora hystrix* (SS1).


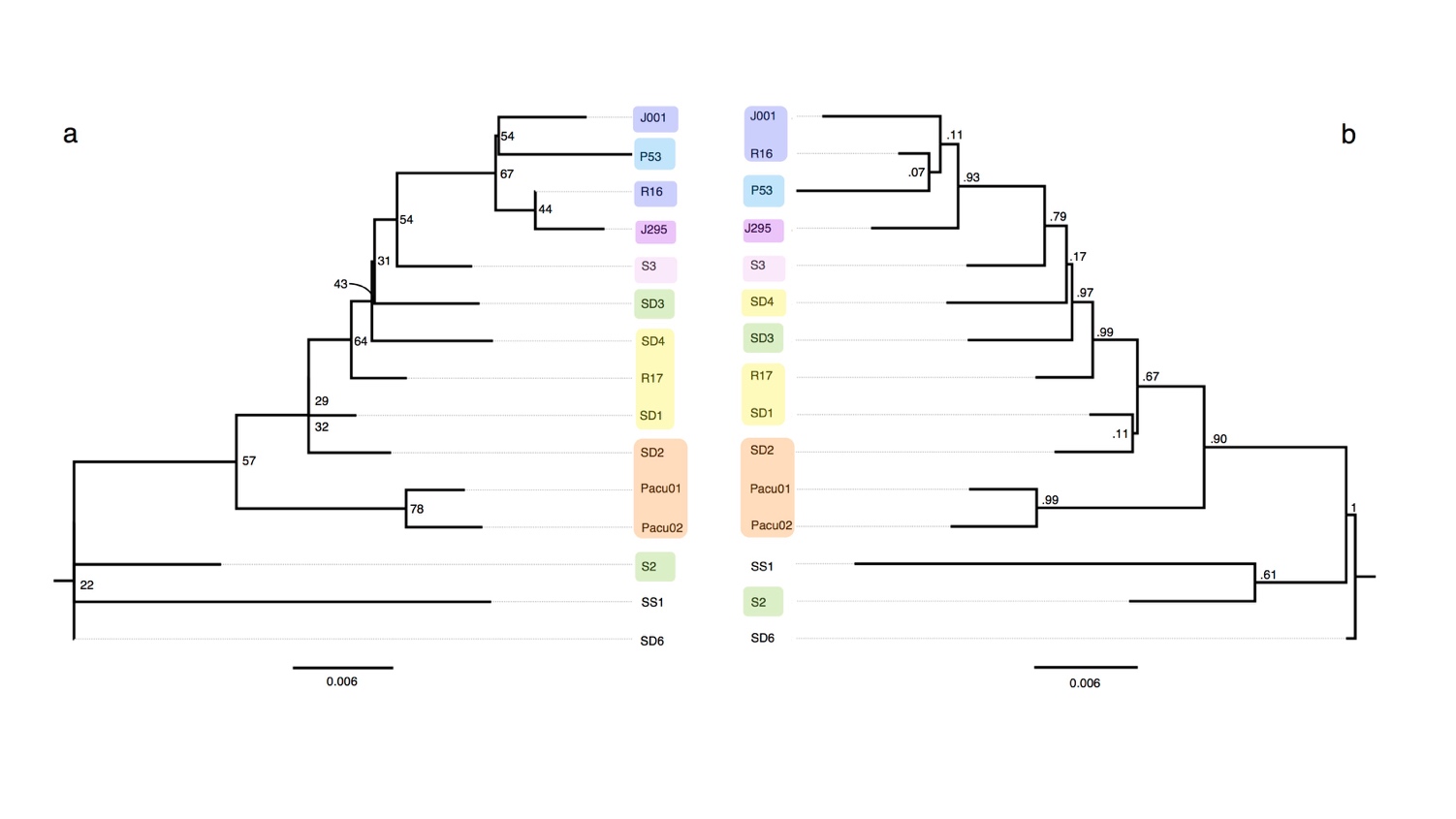


**Figure S3**. Phylogenetic trees from the concatenated *Symbiodinium kawagutii* and *S. minutum* genomes where **a)**: Maximum likelihood analysis; **b)**: Bayesian; yellow: *Pocillopora damicornis*; orange: *P. acuta*; green: *P. verrucosa*; light blue: *P. meandrina*; dark blue: *P. eydouxi*; light purple: *P. ligulata*; dark purple: *P.* sp. B. and two outgroups, *Stylophora pistillata* (SD6), *Seriatopora hystrix* (SS1).

**Supplementary Table S1**. Summary statistics for all libraries from pyRAD (Eaton 2014). Number of total non-homologous loci, number of non-homologous loci with
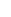
6x coverage depth, and the number of polymorphic sites in loci with
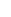
6x coverage depth.

|  | **Holobiont** | | | ***Pocillopora damicornis*** **transcriptome** | | | **Concatenated *S. kawagutii* and *S. minutum*** **genomes** | | |
| --- | --- | --- | --- | --- | --- | --- | --- | --- | --- |
| **Taxon** | **Loci** | **≥** **6 depth coverage** | **Polymorphic sites** | **Loci** | **≥** **6 depth coverage** | **Polymorphic sites** | **Loci** | **≥** **6 depth coverage** | **Polymorphic sites** |
| J001 | 195,855 | 25,418 | 345 | 4,754 | 537 | 3 | 518 | 20 | 0 |
| J295 | 179,037 | 94,656 | 8,483 | 5,521 | 2,782 | 253 | 2,396 | 119 | 3 |
| P53 | 238,160 | 94,659 | 3,121 | 8,595 | 3,134 | 112 | 424 | 67 | 0 |
| Pacu01 | 347,558 | 154,974 | 21,601 | 7,692 | 4,292 | 1,048 | 2,954 | 74 | 4 |
| Pacu02 | 358,824 | 96,300 | 8,616 | 9,735 | 3,511 | 569 | 1,914 | 117 | 5 |
| R16 | 293,708 | 93,998 | 11,222 | 7,937 | 2,663 | 406 | 1,563 | 87 | 2 |
| R17 | 531,983 | 188,589 | 9,640 | 7,792 | 4,311 | 413 | 9,899 | 615 | 25 |
| S2 | 1,221,076 | 98,362 | 600 | 53,295 | 4,638 | 80 | 8,252 | 246 | 6 |
| S3 | 823,513 | 334,262 | 32,163 | 16,284 | 7,222 | 1,450 | 30,291 | 20,079 | 46 |
| SD1 | 518,683 | 231,590 | 32,724 | 9,013 | 6,256 | 1,047 | 9,686 | 381 | 10 |
| SD2 | 398,574 | 186,409 | 37,824 | 11,277 | 5,294 | 1,295 | 3,525 | 134 | 4 |
| SD3 | 475,050 | 220,682 | 50,135 | 12,006 | 6,626 | 1,806 | 2,271 | 141 | 4 |
| SD4 | 292,579 | 140,951 | 9,659 | 5,404 | 3,046 | 318 | 3,398 | 409 | 11 |
| SD6 | 453,744 | 192,081 | 28,007 | 6,790 | 3,185 | 368 | 1,827 | 107 | 1 |
| SS1 | 265,786 | 69,932 | 2,790 | 2,593 | 649 | 29 | 1,459 | 53 | 1 |
|  |  |  |  |  |  |  |  |  |  |
| **Average** | 439,608.7 | 148,190.9 | 17,128.7 | 11,245.9 | 3,876.4 | 613.1 | 5,358.5 | 309.9 | 8.1 |

**Supplementary Table S2**. Sample collection information, average length of contigs in holobiont data sets, and the number of non-homologous contigs in the holobiont and transcriptomic datasets, and the number of contigs that mapped to the mt genome, rDNA, and histone data sets. Abbreviations: mean cov. = mean depth of coverage, ref. seq. = percent coverage of reference sequence.

|  |  |  |  |  | **mt genome** | | | **rDNA** | | | **histone** | | |  |
| --- | --- | --- | --- | --- | --- | --- | --- | --- | --- | --- | --- | --- | --- | --- |
| **ID** | **Location** | **avg. length** | **Holobiont** | **Transcriptome** | **contigs** | **mean cov.** | **ref. seq.** | **contigs** | **mean cov.** | **ref. seq.** | **contigs** | **mean cov.** | **ref. seq.** | **SRA #** |
| J001 | Gulf of California, Mexico | 157.7 | 543,032 | 13,098 | 1,043 | 5 | 81.8% | 300 | 10 | 80.3% | 298 | 4.7 | 73.8% | SAMN06928177 |
| J295 | Clipperton Island, France | 238.4 | 1,244,448 | 39,789 | 767 | 3.6 | 45.2% | 39,974 | 5,113.1 | 100.0% | 228 | 3.6 | 30.3% | SAMN06928180 |
| P53 | Kaneohe Bay, Oahu, Hawaii | 228.9 | 613,970 | 19,660 | 604 | 5.9 | 82.3% | 497 | 1.4 | 84.0% | 375 | 13 | 69.7% | SAMN06928176 |
| Pacu01 | Kaneohe Bay, Oahu, Hawaii | 121.7 | 1,519,983 | 53,401 | 2,189 | 11.5 | 42.8% | 3,356 | 271.7 | 100.0% | 1,233 | 20.7 | 69.9% | SAMN06928173 |
| Pacu02 | Kaneohe Bay, Oahu, Hawaii | 136.7 | 918,257 | 35,798 | 1,495 | 7.8 | 62.9% | 9,828 | 1,764.60 | 100.0% | 746 | 13 | 81.4% | SAMN06928172 |
| R16 | French Frigate Shoals, NWHI, Hawaii | 179.3 | 1,070,202 | 30,642 | 1,805 | 10.2 | 40.1% | 296 | 29.7 | 82.7% | 3,770 | 70.5 | 74.0% | SAMN06928178 |
| R17 | Pearl and Hermes Atoll, NWHI, Hawaii | 171.8 | 1,566,979 | 40,075 | 3,891 | 22.5 | 37.7% | 4,272 | 355 | 87.2% | 692 | 13.6 | 62.1% | SAMN06928168 |
| S2 | French Frigate Shoals, NWHI, Hawaii | 218.4 | 3,327,474 | 135,798 | 15,254 | 135.5 | 100.0% | 12,268 | 1,803.0 | 100.0% | 23,396 | 647.8 | 100.0% | SAMN06928174 |
| S3 | Midway Atoll, NWHI, Hawaii | 214.3 | 3,557,502 | 99,194 | 23,044 | 205.8 | 90.3% | 2,676 | 310.5 | 100.0% | 3,207 | 94.1 | 95.3% | SAMN06928179 |
| SD1 | Davies Reef, Australia | 172.9 | 2,609,953 | 81,056 | 4,815 | 27.6 | 44.0% | 6,963 | 738.5 | 86.7% | 4,518 | 90.5 | 59.6% | SAMN06928169 |
| SD2 | Davies Reef, Australia | 172.2 | 2,214,502 | 63,697 | 5,386 | 33.6 | 56.2% | 2,146 | 209.4 | 84.5% | 695 | 13.7 | 13.7% | SAMN06928171 |
| SD3 | Davies Reef, Australia | 173.2 | 2,674,722 | 81,191 | 7,613 | 57.7 | 89.6% | 755 | 61 | 85.6% | 13,533 | 296.7 | 96.1% | SAMN06928175 |
| SD4 | Davies Reef, Australia | 169 | 1,044,934 | 24,254 | 1,397 | 10.3 | 35.1% | 290 | 25.7 | 62.5% | 2,515 | 45.1 | 60.8% | SAMN06928170 |
| SD6 | Davies Reef, Australia | 170.4 | 2,639,615 | 34,654 | 5,741 | 43.3 | 81.7% | 909 | 90.4 | 80.6% | 5 | 0.1 | 4.5% | SAMN06928181 |
| SS1 | Davies Reef, Australia | 155.5 | 514,498 | 8,648 | 0 | 0 | 0.0% | 10,856 | 1,552.50 | 92.7% | 0 | 0 | 0.0% | SAMN06928182 |
|  |  |  |  |  |  |  |  |  |  |  |  |  |  |  |
|  | **Average** | 178.7 | 1,737,338 | 50,730 | 5,360.3 | 41.5 | 63.6% | 6,359.1 | 822.4 | 88% | 3,943.6 | 94.8 | 63.70% |  |

**18S ITS1 5.8S ITS2 28S Phylip Alignment (Accession number: AY722785)**

Pocillopora damicornis clone PEN400.1 18S ribosomal RNA gene, partial sequence; internal transcribed spacer 1, 5.8S ribosomal RNA gene, and internal transcribed spacer 2, complete sequence; and 28S ribosomal RNA gene, partial sequence.

15 1399

J001 ??????????????????????????????????????????????????????????????TCACCCTCTGCGACGTGCCGTTCCAAGCAACTTAGGCAGGGCCCCTCCGCCTAGAAAGTGCTTCTCGCAACTACAACTCGCCGATGCAGGCATCGGAGATTTCAAATTTGAGCTCTTCCCGCTTCACTCGCCGTTACTGGGGGAATCCTTGTTAGTTTCTTTTCCTCCGCTTATTAATATGCTTAAATTCAGCGGGTAGCCTTGCCTGATCTGAGGTCTGGAAGGCGATTCCTTTTTTCCTTTGAGATGGCGCCACCGCTACCCGGCGGCAGCAGAAAAAAGAATCGAATGGAGAAAGATTTGTTCCGTCAAAGCGATAGAGCCGTGGCCGKTTRGGGWWYMTTGTTCTATGAYC???????????????????????????????????????????????????????????????????????????????????????????????????????????????????????????????????????????????????????????????????????????????????????????????????????????????????????????????????????????ACACTCAGACAGACATGCTCCYGGTAKARCCNAAGAGCGCCATTTGCGTTCAAAGATTCGATGATTCACTGAATTCTGCAATTCACACTACKTATCGCAGCTGGCTGCGTTCTTCATCGATGCRYGAGCCWAGAGATMCACCGTCAAA-AGTTGTCAAACTTTTTTTTT-CTCTATAGATCCATTCTACAGGTATCTCTGTGCTCAGTTCTTTGAARCRAAT-CGT-C-TGTC-AAACGTCTG-CGGGGCAGGAGCCGACCGCGCCGCGGCACATCGAGTGCACGCACTA-GATAGACACGGAGASAGAGASA------------CACACACAACGAGAAACGTAGGCAGGCACCGACGCGGCGCACGAAGCGTCCTACGCCACACCGCACGGTCACGCTACGATCCCAMTGAGCAGTC----TCCATC-A------------AAAGTC----ACCTAGCACSGCAGTCCGAGCGACGCCTCGCGGCCCTCTCCTTTTAACGGAACCAGCCGACCCCTAATGATAATTTTTTCCACCGGTTCCCAGGGTAGAAGAACTCGTAGTACTAAAACTACGTATCTCTCTTTCGGTAATGATCCTTCCGCAGGTTCACCTACGGAAACCTTGTTACGACTTYTMCTTCCTCTAARTGATCAAGTTTGAACAACTTCCCGGCCTCCCACTCTCTGCACGAGACAGAGTGTCGGCGCCAGTCAGGAGGCCTCACTAAACCATTCAATCGGTAGT

J295 CCAAACAACCCGACTCTTCGAAAGCACATCGTGGGCGGCCGGCCCTGCCACAGACGGGGTTGTCACCCTCTGCGACGTGCCGTTCCAAGCAACTTAGGCAGGGCCCCTCCGCCTAGAAAGTGCTTCTCGCAACTACAACTCGCCGATGCAAGCATCGGAGATTTCAAATTTGAGCTCTTCCCGCTTCACTCGCCGTTACTGGGGGAATCCTTGTTAGTTTCTTTTCCTCCGCTTATTAATATGCTTAAATTCAGCGGGTAGCCTTGCCTGATCTGAGGTCTGGAAGGCGATTCCTTTTTTCCTTTGAGATGCCGCCACCGCTACCCGGCGGCAGCAGAAAAAAGAATCGAATGGAGAAAGATTTGTTCCGTCAAAGCGATAGAGCCGTGGCCGTTTAGGGTATCTTGTTCTATGATC?--??????????????????????GATCTTTCTCCCTGAATTTCAAGGGACGCGGTAAACCGACCGGTCGGGCCGAGCACCACCAGGGGTAGCAAGCRCACGACCGGTCATCTCGACCGCGACCCTCAACGCCGCACGAACCCGTTCACGGTGGGCGCGTCCCGGCCCCGTCCGCTACAGACGGGGACCAGGCGGACGCGCGCACGCGGATTCGCACGATGGGTGTTTGAATAGACACTCAGACAGACATGCTCCTGGGAGAACCCAAGAGCGCCATTTGCGTTCAAAGATTCGATGATTCACTGAATTCTGCAATTCACACTACTTATCGCAGCTGGCTGCGTTCTTCATCGATGCGTGAGCCTAGAGATCCACCGTCAAA-AGTTGTCAAACTTTTTTTTT-CTCTATNGATCCATTCTACAGGTATCTCTGTGCTCAGTTCTTTGAACCAAAT-CGT-C-TGTC-AAACGTCTG-CGGGGCAGGAGCCGACCGCGCCACGGCACATCGAGTGCACGCACTA-GATAGACACGGAGAGAAAGAGAGA----------CACACGCAAAGAGAAACGTAGGCAGRCACCGACGCGGCGCACGAAGCGTCCTACGCCACACCGCACGGTCACGCTACGATCCCACTGAGCAGTCAGTCTCCATCCATCTC----CACAAAAGTCAGTTAGCTAGCACGGCAGTCCGAGCGACGCCTCGCGGCCCTCTCCTTTTAACGGAACCAGCCGACCCCTAATGATAATTTTTTCCACTGGTTCCCAGGGTAGAAGAACTCGTAGTACTTAAACTACGTATCTCTCTTTCGGTAATGATCCTTCCGCAGGTTCACCTACGGAAACCTTGTTACGACTTTTACTTCCTCTAAATGATCAAGTTTGAACAACTTCCCGGCCTCCCACTCTCTGCACGAGACAGAGTGTCGGCGCCAGTCAGGAGGCCTCACTAAACCATTCAATCGGTAGT

P53 ??????????????????????????????????????????????????????????????????????????????GCCGTTCCAAGCAACTTAGGCAGGGCCCCTCCGCCTAGAAAGTGCTTCTCGCAACTACAACTCGCCGATGCAGGCATCGGAGATTTCAAATTTGAGCTCTTCCCGCTTCACTCGCCGTTACTGGGGGAATCCTTGTTAGTTTCTTTTCCTCCGCTTATTAATATGCTTAAATTCAGCGGGTAGCCTTGCCTGATCTGAGGTCTGGAAGGCGATTCCTTTTTTCCTTTGAGATGCCGCCACCGCTACCCGGCGGCAGCAGAAAAAAGAATCGAATGGAGAAAGATTTGWTCCGTCAAAGCGATAGAGCCGTGGCCGTTTAGGGTATCTTGTTCTATGATCCCCCGCGACACCGTGTCGCTTGGCGGATC????????????????????????????????????????????????????????????????????????????????????????????????????????????????????????????????????????????????????ACAGACGGGGACCAGGCGGACGCGCGCACGCGGATTCGCACGATGG-TGTTTGAATAGACACTCAGACAGACATGCTCCTGGGAGAACCCAAGAGCGCCATTTGCGTTCAAAGATTCGATGATTCACTGAATTCTGCAATTCACACTACTTATCGCAGCTGGCTGCGTTCTTCATCGATGCGTGAGCCTAGAGATCCACCGTCAAA-AGTTGTCAAACTTTTTTTT--CTCTATAGATCCATTCTACAGGTATCTCTGTGCTCAGTTCTTTGAAACAAAT-CGT-C-TGTC-AAACGTCTG-CGGGGCAGGAGCCGACCGCGCCGCGGCACATCGAGTGCACGCACTA-GATAGACACGGAGAGAGANNNNNNNN--------CNCACACAACGAGAAACGTAGGCAGGCACCGACGCGGCGCACGAAGCGTCCTACGCCACACCGCACGGTCACGCTACGATCCCACTGAGCAGTC----TCCATC-------------AAAAGTC----ACCTAGCACGGCAGTCCGAGCGACGCCTCGCGGCCCTCTCCTTTTAACGGAACCAGCCGACCCCTAATGATAATTTTTTCCACYGGTTCCCAGGGTAGAAGAACTCGTAGTACTAAAACTACGTATCTCTCTTTCGGTAATGATCCTTCCGCAGGTTCACCTACGGAAACCTTGTTACGACTTTTACTTCCTCTAAATGATCAAGTTTGAACAACTTCCCGGCCTCCCACTCTCTGCACGAGACAGAGTGTCGGCGCCAGTCAGGAGGCCTCACTAAACCATTCAATCGGTAGT

Pacu01 CCAAACAACCCGACTCTTCGAAAGCACATCGTGGGCGGCCGGCCCTGCCACAGACGGGGTTGTCACCCTCTGCGACGTGCCGTTCCAAGCAACTTAGGCAGGGCCCCTCCGCCTAGAAAGTGCTTCTCGCAACTACAACTCGCCGATGCAAGCATCGGAGATTTCAAATTTGAGCTCTTCCCGCTTCACTCGCCGTTACTGGGGGAATCCTTGTTAGTTTCTTTTCCTCCGCTTATTAATATGCTTAAATTCAGCGGGTAGCCTTGCCTGATCTGAGGTCTGGAAGGCGATTCCTTTTTTCCTTTGAGATGCCGCCACCGCTACCCGGCGGCAGCAGAAAAAAGAATCGAATGGAGAAAGATTTGTTCCGTCAAAGCGATAGAGCCGTGGCCGTTTAGGGTATCTTGTTCTATGATCCCCCGCGACACCGTGTCGCTTGGCGGATCTTTCTCCCTGAATTTCAAGGGACGCGGTAAACCGACCGGTCGGGCCGAG---CACCAAGGCT-----GCACACGACCGGTCATCTCGACCGCGACCCTCAACGCCGCACGAACCCGTTCACGGCGGGCGCGTCCCGGCCCCATCCGCTACAGACGGGGACCAGGCGGACGCGCGCACGCGGATTCGCACGATGGGTGTTTGAATAGACACTCAGACAGACATGCTCCTGGGAGAACCCAAGAGCGCCATTTGCGTTCAAAGATTCGATGATTCACTGAATTCTGCAATTCACACTACTTATCGCAGCTGGCTGCGTTCTTCATCGATGCGTGAGCCTAGAGATCCACCGTCAAA-AGTTGTCAAACTTTTTTTTTTCTCTATAGATCCATTTTACAGGTATCTCTGTGCTCAGTTCTTTGAACCAAAT-CGT-C-TGTC-AAACGTCTG-CGGGGCAGGAGCCGACCGCGCCGCGGCACATCGAGTGCACGCACTA-GATAGACACGGAGAGAGAGAAAAGGA--------CACACACAACGAGAAACGTAGGCAGGCACCGACGCGGCGCACGAAGCGTCCTACGCCACACCGCACGGTCACGCTACGATCCCACTGAGCAGTC----TCCATCCATCTC----CACCAAAGTC----ACCTAGCACGGCAGTCCGAGCGACGCCTCGCGGCCCTCTCCTTTTAACGGAACCAGCCGACCCCTAATGATAATTTTTTCCACTGGTTCCCAGGGTAGAAGAACTCGTAGTACTAAAACTACGTATCTCTCTTTCGGTAATGATCCTTCCGCAGGTTCACCTACGGAAACCTTGTTACGACTTTTACTTCCTCTAAATGATCAAGTTTGAACAACTTCCCGGCCTCCCACTCTCTGCACGAGACAGAGTGTCGGCGCCAGTCAGGAGGCCTCACTAAACCATTCAATCGGTAGT

Pacu02 CCAAACAACCCGACTCTTCGAAAGCACATCGTGGGCGGCCGGCCCTGCCACAGACGGGGTTGTCACCCTCTGCGACGTGCCGTTCCAAGCAACTTAGGCAGGGCCSMTCCGCCTAGAAAGTGCTTCTCGCAACTACAACTCGCCGATGCARGCATCGGAGATTTCAAATTTGAGCTCTTCCCGCTTCACTCGCCGTTACTGGGGGAATCCTTGTTAGTTTCTTTTCCTCCGCTTATTAATATGCTTAAATTCAGCGGGTAGCCTTGCCTGATCTGAGGTCTGGAAGGCGATTCCTTTTTTCCTTTGAGATGCCGCCACCGCTACCCGGCGGCAGCAGAAAAAAGAATCGAATGGAGAAAGATTTGTTCCGTCAAAGCGATAGAGCCGTGGCCGTTTAGGGTATCTTGTTCTATGATCCCCCGCGACACCGTGTCGCTTGGCKGATCTTTCTCCCTGAATTTCAAGGGACGCGGTAAACCGACCGGYCGGGCCGAGCAGCACCAGGGCTGGCTAGCRCACGACCGGTCATCTCGACCGCGACCCTCAACGCCGCACGAACCCGTTCACGGCGGGCGCGTCCCGGCCCCATCCGCTACAGACGGRGACCAGGCGGACGCGCGCACGCGGATTCGCACGATGGGTGTTTGAATAGACACTCAGACAGACATGCTCCTGGGAGAACCCAAGAGCGCCATTTGCGTTCAAAGATTCGATGATTCACTGAATTCTGCAATTCACACTACTTATCGCAGCTGGCTGCGTTCTTCATCGATGCGTGAGCCTAGAGATCCACCGTCAAA????????????????????????????AGATCCATTCTACAGGTATCTCTGTGCTCAGTTCTTTGAACCAAAT-CGT-C-TGTC-AAACGTCTG-CGGGGCAGGAGCCGACCGCGCCGCGGCACATCGAGTGCACGCACTA-GATAGACACGGAGAGAGACAGAGAGAGAGACACACACACACAACGAGAAACGTAGGCAGGCACCGACGCGGCGCACGAAGCGTCCTACGCCACACCGCACGGTCACGCTACGATCCCACTGAGCAGTC----TCCATCCATCTC----CACCAAAGTC----ACCTAGCACGGCAGTCCGAGCGACGCCTCGCGGCCCTCTCCTTTTAACGGAACCAGCCGACCCCTAATGATAATTTTTTCCACTGGTTCCCAGGGTAGAAGAACTCGTAGTACTAARACTACGTATCTCTCTTTCGGTAATGATCCTTCCGCAGGTTCACCTACGGAAACCTTGTTACGACTTCTCCTTCCTCTAAGTGATCAAGTTTGAACAACTTCCCGGCCTCCCACTCTCTGCACGAGACAGAGTGTCGGCGCCAGTCAGGAGGCCTCACTAAACCATTCAATCGGTAGT

R16 ???????????????????????????????????????????????????????????????????????????????????????????????AGGCAGGGCCCCTCCGCCTAGAAAGTGCTTCTCGCAACTACAACTCGCCGATGCAGGCATCGGAGATTTCAAATTTGAGCTCTTCCCGCTTCACTCGCCGTTACTGGGGGAATCCTTGTTAGTTTCTTTTCCTCCGCTTATTAATATGCTTAAATTCAGCGGGTAGCCTTGCCTGATCTGAGGTCTGGAAGGCGATTCCTTTTTTCCTTTGAGATGCCGCCACCGCTACCCGGCGGCAGCAGAAAAAAGAATCGAATGGAGAAAGATTTGTTCCGTCAAAGCGATAGAGCCGTGGCCGTTTGGGGTACATTGTTCTATGATCC????????????????????????GATCTTTCTCCCTGAATTTCAAGGGACGCGGTAAACCGACCGGTCGGGCCGAGCAGCACCAGGGCTGGCTAGCGCMCGACCGGTCATCTCGACCGCGACCC??????????????????????????????????????????????????????????????????????????????????????????????????TGTTTGAATAGACACTCAGACAGWCATGCTCCTGGGAGAACCCAAGAGCGCCATTTGCGTTCAAAGATTCGATGATTCACTGAATTCTGCAATTCACACTACTTATCGCAGCTGGCTGCGTTCTTCATCGATGCGTGAGCCTAGAGATCC----T-----A---------------------------GATCCATTCTACAGGTATCTCTGTGCTCAGTTCTTTGAAGCGAAT-CGT-C-TGTC-AAACGTCTG-CGGGGCAGGAGCCGACCGCGCCGCGGCACATCGAGTGCACGCACTA-GATAGACACGGAGAGAGAGAGA------------CACACACAACGAGAAACGTAGGCAGGCACCGACGCGGCGCACGAAGCGTCCTACGCCACACCGCACGGTCACGCTACGATCCCACTGCCCAMTGAGCAGTC-TCCATC---------AAAAGTC----ACCTAGCACGGCAGTCCGAGCGACGCCTCGCGGCCCTCTCCTTTTAACGGAACCAGCCGACCCCTAATGATAATTTTTTCCACCGGTTCCCAGGGTAGAAGAACTCGTAGTACTAAAACTACGTATCTCTCTTTCGGTAATGATCCTTCCGCAGGTTCACCTACGGAAACCTTGTTACGACTTCTCCTTCCTCTA---GATCAAGTTTGAACAACTTCCCGGCCTCCCACTCTCTGCACGAGACAGAGTGTCGGCGCCAGTCAGGAGGCCTCACTARAYCAYTCAATCGGTAGK

R17 ??????????????????????????????????????????????????????????????????????????????????????????AACTTAGGCAGGGCCCCTCCGCCTAGAAAGTGCTTCTCGCAACTACAACTCGCCGATGCAGGCATCGGAGATTTCAAATTTGAGCTCTTCCCGCTTCACTCGCCGTTACTGGGGGAATCCTTGTTAGTTTCTTTTCCTCCGCTTATTAATATGCTTAAATTCAGCGGGTAGCCTTGCCTGATCTGAGGTCTGGAAGGCGATTCCTTTTTTCCTTTGAGATGCCGCCACCGCTACCCGGCGGCAGCAGAAAAAAGAATCGAATGGAGAAAGATTTGTTCCGTCAAAGCGATAGAGCCGTGGCCGTTTAGGGTATCTTGTTCTATGATCCCCCGCGACACCGTGTCGCTTGGCGGATCTTTCTCCCTGAATTTCAAGGG--GCGGTAAACC??????????????????????????????????????????????????????????????????????????????????????????????????????GGCCCCATCCGCTACAGMYSRRKAYCAGGCGGACGCGCGCACGCGGATTCGCACGATGGGTGTTTGAATAGACACTCAGACAGACATGCTCCTGGGAGAACCCAAGAGCGCCATTTGCGTTCAAAGATTCGATGATTCACTGAATTCTGCAATTCACACTACTTATCGCAGCTGGCTGCGTTCTTCATCGATGCGTGAGCCTAGAGATC?????????????????????????????---??????AGATCCACTCTACAGGTATCTCTGTGCTCAGTTCTTTGAAACAAAT-CGT-C-TGTC-AAACGTCTG-CGGGGCAGGAGCCGACCGCGCCGCGGCACATCGAGTGCACGCACTA-GATAGACACGGAGAGAGACAGAGAGAGAAAAAGACACACACAACGAGAAACGTAGGCAGGCACCGACGCGGCGCACGAAGCGTCCTACGCCACACCGCACGGTCACGCTACGATCCCACTGAGCAGTCTCCATCCATCCATCNATCTCCACCAAAGTC----ACCTAGCACGGCAGTCCGAGCGACGCCTCGCGGCCCTCTCCTTTTAACGGAACCAGCCGACCCCTAATGATAATTTTTTCCACTGGTTCCCAGGGTAGAAGAACTCGTAGTACTAAAACTACGTATCTCTCTTTCGGTAATGATCCTTCCGCAGGTTCACCTACGGAAACCTTGTTACGACTTCTCCTTCCTCTAAGTGATAAGGTTCRWRMAACTTYCCGRCSYMMCRYYCWSWRSMYGAGACWGMGTGTCNGMNYCANTNANNNGNNCNNANNNRAYCAYTCAATCGGTAGK

S2 CCAAACAACCCGACTCTTCGAAAGCACATCGTGGGCGGCCGGCCCTGCCACAGACGGGGTTGTCACCCTCTGCGACGTGCCGTTCCAAGCAACTTAGGCAGGGCCCCTCCGCCTAGAAAGTGCTTCTCGCAACTACAACTCGCCGATGCAGGCATCGGAGATTTCAAATTTGAGCTCTTCCCGCTTCACTCGCCGTTACTGGGGGAATCCTTGTTAGTTTCTTTTCCTCCGCTTATTAATATGCTTAAATTCAGCGGGTAGCCTTGCCTGATCTGAGGTCTGGAAGGCGATTCCTTTTTTCCTTTGAGATGCCGCCACCGCTACCCGGCGGCAGCAGAAAAAAGAATCGAATGGAGAAAGATTTGTTCCGTCAAAGCGATAGAGCCGTGGCCGTTTAGGGTATCTTGTTCTATGATCCCCCGCGACACCGTGTCGCTTGGCGGATCTTTCTCCCTGAATTTCAAGGGACGCGGTAAACCGACCGGTCGGGCCGAGCAGCACCAGGGCTGGCTAGCGCACGACCGGTCATCTCGACCGCGACCCTCAACGCCGCACGAACCCGTTCACGGCGGGCGCGTCCCGGCCCCATCCGCTACAGACGGGGACCAGGCGGACGCGCGCACGCGGATTCGCACGATGGGTGTTTGAATAGACACTCAGACAGACATGCTCCTGGGAGAACCCAAGAGCGCCATTTGCGTTCAAAGATTCGATGATTCACTGAATTCTGCAATTCACACTACTTATCGCAGCTGGCTGCGTTCTTCATCGATGCGTGAGCCTAGAGATCCACCGTCAAAA-GTTGTCAAACTTTTTTTTT-CTCTATAGATCCATTCTACAGGTATCTCTGTGCTCAGTTCTTTGAAACAAAT-CGT-C-TGTC-AAACGTCTG-CGGGGCAGGAGCCGACCGCGCCGCGGCACATCGAGTGCACGCACTA-GATAGACACGGAGAGAGACAGAGAGACA------CACACACAACGAGAAACGTAGGCAGGCACCGACGCGGCGCACGAAGCGTCCTACGCCACACCGCACGGTCACGCTACGATCCCACTCAGCAGTC----TCCATCCATCTC----CACCAAAGTC----ACCTAGCACGGCAGTCCGAGCGACGCCTCGCGGCCCTCTCCTTTTAACGGAACCAGCCGACCCCTAATGATAATTTTTTCCACTGGTTCCCAGGGTAGAAGAACTCGTAGTACTAAAACTACGTATCTCTCTTTCGGTAATGATCCTTCCGCAGGTTCACCTACGGAAACCTTGTTACGACTTTTACTTCCTCTAAATGATCAAGTTTGAACAACTTCCCGGCCTCCCACTCTCTGCACGAGACAGAGTGTCGGCGCCAGTCAGGAGGCCTCACTAAACCATTCAATCGGTAGT

S3 CCAAACAACCCGACTCTTCGAAAGCACATCGTGGGCGGCCGGCCCTGCCACAGACGGGGTTGTCACCCTCTGCGACGTGCCGTTCCAAGCAACTTAGGCAGGGCCSCTCCGCCTAGAAAGTGCTTCTCGCAACTACAACTCGCCGAYGCAAGCRTCGGAGATTTCAAATTTGAGCTCTTCCCGCTTCACTCGCCGTTACTGRGGGAATCCTTGTTAGTTTCTTTTCCTCCGCTTATTAATATGCTTAAATTCAGCGGGTAGCCTTGCCTGATCTGAGGTCTGGAAGGCGATTCCTTTTTTCCTTTGAGATGCCGCCACCGCTACCCGGTGGCAGCAGAAAAAAGAATCGAATGGAGAAAGATTTGTTCCGTCAAAGCGATAGAGCCGTGGCCGTTTAGGGTATCTTGTTCTATGATCCCCCGCGACACCGTGTCGCTTGGCGGATCTTTCTCCCTGAATTTCAAGGGACGCGGTAAACCGACCGGTCGGGCCGAGCACCACCAGGGGTAGCTAGCGCACGACCGGTCATCTCGACCGCGACCCTCAACGCCGCACGAACCCGTTCACGGTGGGCGCGTCCCGGCCCCATCCGCTACAGACGGGGACCAGGCGAACGCGCGCACGCGGATTCGCACGATGGGTGTTTGAATAGACACTCAGACAGACATGCTCCTGGGAGAACCCAAGAGCGCCATTTGCGTTCAAAGATTCGATGATTCACTGAATTCTGCAATTCACACTACTTATCGCAGCTGGCTGCGTTCTTCATCGATGCGTGAGCCTAGAGATCCACCGTCAAAA-GTTGTCAAACTTTTTTTTT-CTCTATAGATCCATTCTACAGGTATCTCTGTGCTCAGTTCTTTGAACCAAAT-CGT-C-TGTC-AAACGTCTG-CGGGGCAGGAGCCGACCGCGCCGCGGCACATCGAGTGCACGCACTA-GATAGACACGGAGAGAAAGAGAGA----------CACACGCAAAGAGAAACGTAGGCAGGCACCGACGCGGCGCACGAAGCGTCCTACGCCACACCGCACGGTCACGCTACGATCCCAGTGAGCAGTCAGTCTCCATCCATCTC----CACAAAAGTCAGTTAGCTAGCACGGCAGTCCGAGCGACGCCTCGCGGCCCTCTCCTTTTAACGGAACCAGCCGACCCCTAATGATAATTTTTTCCACCGGTTCCCAGGGTAGAAGAACTCGTAGTACTAAAACTACGTATCTCTCTTTCGGTAATGATCCTTCCGCAGGTTCACCTACGGAAACCTTGTTACGACTTCTCCTTCCTCTAAGTGATCAAGTTTGAACAACTTCCCGGCCTCCCACTCTCTGCACGAGACAGAGTGTCGGCGCCAGTCAGGAGGCCTCACTAAACCATTCAATCGGTAGT

SD1 ??????????????????????????????????????????????GCCACAGACGGGGTTGTCACCCTCTGCGACGTGCCGTTCCAAGCAACTTAGGCAGGGCCCCTCCGCCTAGAAAGTGCTTCTCGCAACTACAACTCGCCGATGCAGGCATCGGAGATTTCAAATTTGAGCTCTTCCCGCTTCACTCGCCGTTACTGGGGGAATCCTTGTTAGTTTCTTTTCCTCCGCTTATTAATATGCTTAAATTCAGCGGGTAGCCTTGCCTGATCTGAGGTCTGGAAGGCGATTCCTTTTTTCCTTTGAGATGCCGCCACCGCTACCCGGCGGCAGCAGAAAAAAGAATCGAATGGAGAAAGATTTGTTCCGTCAAAGCGATAGAGCCGTGGCCGTTTAGGGTATCTTGTTCTATGATCCCCCGCGACACCGTGTCGCTTGGCGGATCTTTCTCCCTGAATTTCAAGGGMCGCGGTAAACCGACCGGTCGGGCCGAGCAGCACCAGGGCTGKCTAGCGCACGACCGGTCATCTCGACCGCGACCCTCMACGCCGC?????????????????????????????????????????????????????????????????????????????????????GGGTGTTTGAATAGACACTCAGACAGACATGCTCCTGGGAGAACCCAAGAGCGCCATTTGCGTTCAAAGATTCGATGATTCACTGAATTCTGCAATTCACACTACTTATCGCAGCTGGCTGCGTTCTTCATCGATGCGTGAGCCTAGAGATC?????????????????????????????---??????AGATCCACTCTACAGGTATCTCTGTGCTCAGTTCTTTGAAACAAAT-CGT-C-TGTC-AAACGTCTG-CGGGGCAGGAGCCGACCGCGCCGCGGCACATCGAGTGCACGCACTA-GATAGACACGGAGAGAGACAGAGAAAAAGA----CACACACAACGAGAAACGTAGGCAGGCACCGACGCGGCGCACGAAGCGTCCTACGCCACACCGCACGGTCACGCTACGATCCCACTGAGCAGTCTCCATCCATCCATCTC----CACCAAAGTC----ACCTAGCACGGCAGTCCGAGCGACGCCTCGCGGCCCTCTCCTTTTAACGGAACCAGCCGACCCCTAATGATAATTTTTTCCACTGGTTCCCAGGGTAGAAGAACTCGTAGTACTAAAACTACGTATCTCTCTTTCGGTAATGATCCTTCCGCAGGTTCACCTACGGAAACCTTGTTACGACTTCTCCTTCCTCTAAGTGATCAAGTTTGAACAACTTCCCGGCCTCCCACTCTCTGCACGAGACAGAGTGTCGGCGCCAGTCAGGAGGCCTCACTAAACCATTCAATCGGTAGT

SD2 CCAAACAACCCGACTCTTCGAAAGCACATCGTGGGCGGCCGGCCCTGCCACAGACGGGGNTGTCACC??CTGCGACGTGCCGTTCCAAGCAACTTAGGCAGGGCCCCTCCGCCTAGAAAGTGCTTCTCGCAACTACAACTCGCCGATGCAGGCATCGGAGATTTCAAATTTGAGCTMTTCCCGCTTCACTCGCCGTTACTGGGGGAATCCTTGTTAGTTTCTTTTCCTCCGCTTATTAATATGCTTAAATTCAGCGGGTAGCCTTGYCTGATCTGAGGTCTGGAAGGCGATTCCTTTTTTCCTTTGAGATGCCGCCACCGCTACCCGGCGGCAGCAGAAAAAAGAATCGAATGGAGAAAGATTTGTTCCGTCAAAGCGATAGAGCCGTGGCCGTTTAGGGTATCTTGTTCTATGATC???-????????????????????????????????????????????????????????????????????????????????????????????????????????????????????????????????????????????????????????????????????????????????????????????????????????????????????????????TGTTTGAATAGACACTCAGACAGACATGCTCCTGGGAGAACCCAAGAGCGCCATTTGCGTTCAAAGATTCGATGATTCACTGAATTCTGCAATTCACACTACTTATCGCAGCTGGCTGCGTTCTTCATCGATGCGTGAGCCTAGAGATCCACCGTCAAAA-GTTGTCAAACTTTTTTTTTNCTCTATAGATCCACTCTACAGGTATCTCTGTGCTCAGTTCTTTGAACCAAAC-CGT-C-TGTC-AAACGTCTG-CGGGGCAGGAGCCGACCGCGCCGCGGCACATCGAGTGCACGCACTA-GATAGACACGGAGAGAGAGAAAAAGA--------CGCACACAACGAGAAACGTAGGCAGGCACCGACGCGGCGCACGAAGCGTCCTACGCCACACCGCACGGTCACGCTACGATCCCACTGAACAGTC----TCCATCCATCTC----CACCAAAGTC----ACCTAGCACGGCAGTCCGAGCGACGCCTCGCGGCCCTCTCCTTTTAACGGAACCAGCCGACCCCTAATGATAATTTTTTCCACCGGTTCCCAGGGTAGAAGAACTCGTAGTACTAAAACTACGTATCTCTCTTTCGGTAATGATCCTTCCGCAGGTTCACCTACGGAAACCTTGTTACGACTTTTMCTTCCTCTAAATGATCAAGTTTGAACAACTTCCCGGCCTCCCACTCTCTGCACGAGACAGAGTGTCGGCGCCAGTCAGGAGGCCTCACTAAACCATTCAATCGGTAGT

SD3 CCAAACAACCCGAC?????????????????????????????????????????????????CACCCTCTGCGACGTGCCGTTCCAAGCAACTTAGGCAGGGCCCCTCCGCCTAGAAAGTGCTTCTCGCAACTMCAACTCGCCGATGCAGGCATCGGAGATTTCAAATTTGAGCTCTTCCCGCTTCACTCGCCGTTACTGGGGGAATCCTTGTTAGTTTCTTTTCCTCCGCTTATTAATATGCTTAAATTCAGCGGGTAGCCTTGCCTGATCTGAGGTCTGGAAGGCGATTCCTTTTTTCCTTTGAGATGCCGCCACCGCTACCCGGCGGCAGCAGAAAAAAGAATCGAATGGAGAAAGATTTGTTCCGTCAAAGCGATAGAGCCGTGGCCGTTTAGGGTATCTTGTTCTATGATCCCCCGCGACACCGTGTCGCTTGGCGGATC?????????????????????????????????????????????????????????????????-???????????????????????????????????????????????????????????????????????????????????????????????????????????????????????????????GGTGTTTGAATAGACACTCAGACAGACATGCTCCTGGGAGAACCCAAGAGCGCCATTTGCGTTCAAAGATTCGATGATTCACTGAATTCTGCAATTCACACTACTTATCGCAGCTGGCTGCGTTCTTCATCGATGCGTGAGCCTAGAGATCCACCGTCAAAA-GTTGTCAAACTTTTTTTTT-CTCTATAGATMCATTCTACAGGTATCTCTGTGCTCAGTTCTTTGAACCAAAT-CGT-C-TGTC-AAACGTCTG-CGGGGCAGGAGCCGACCGCGCCGCGGCACATCGAGTGCACGCACTA-GATAGACACGGAGAGAGAMARRGA----------CACACACAACGAGAAACGTAGGCAGGCACCGACGCGGCGCACGAAGCGTCCTACGCCACACCGCACGGTCACGCTACGATCCCACTGAGCAGTC----TCCATCCATCTCNNN-CACCAAAGTC----ASCTAGYACGGCAGTCCGAGCGACGCCTCGYGGCCCTCTCCTTTTAACGGAACCAGCCGACCCCTAATGATAATTTTTTCCACTGGTTCCCAGGGTAGAAGAACTCGTAGTACTAAAACTACGTATCTCTCTTTCGGTAATGATCCTTCCGCAGGTTCACCTACGGAAACCTTGTTACGACTTNTNCTTCCTCTAANTGATCAAGTTTGAACAACTTCCCGGCCTCCCACTCTCTGCACGAGACAGAGTGTCGGCGCCAGTCAGGAGGCCTCACTAAACCATTCAATCGGTAGT

SD4 ?????????????????????????????????????????????????????????????????????????????????????????????????????????????????????????????????????????????????????????????????????????????????????????????????????????????????????????????????????????????????????????????????????????????GATCTGAGGTCTGGAAGGCGATTCCTTTTTTCCTTTGAGATGCCGCCACCGCTACCCGGCGGCAGCAGAAAAAAGAATCGAATGGAGAAAGATTTGTTCCGTCAAAGCGATAGAGCCGTGGCCGTTTAGGGTATCTTGTTCTATGATC???-?????????--???????????????????????????????????????????????????????????????????????????????-??????????????????????????????????????????????????????????????????????????????????????????????????????????????????????????????????????????AGACACTCAGACAGACATGCTCCTGGGAGAACCCAAGAGCGCCATTTGCGTTCAAAGATTCGATGATTCACTGAATTCTGCAATTCACACTACTTATCGCAGCTGGCTGCGTTCTTCATCGATGCGTGAGCCTAGAGATCCACCGTCAAAA-GTTGTCAAACTTTTTTTTT-CTCTATAGATCCANTCTACAGGTATCTCTGTGCTCAGTTCTTTGAAACAAAT-CGT-C-TGTC-AAACGTCTG-CGGGGCAGGAGCCGACCGCGCCGCGGCACATCGAGTGCACGCACTA-GATAGACACGGAGAGAGACAGAGAAAGAGA--CACACACACAACGAGAAACGTAGGCAGGCACCGACGCGGCGCACGAAGCGTCCTACGCCACACCGCACGGTCACGCTACGATCCCACTGAGCAGTC----TCCATCCATCNNNNTCCACCAAAGTC----ACCTAGCACGGCAGTCCGAGCGACGCCTCGCGGCCCTCTCCTTTTAACGGAACCAGCCGACCCCTAATGATAATTTTTTCCACTGGTTCCCAGGGTAGAAGAACTCGTAGTACTAAAACTACGTATCTCTCTTTCGGTAATGATCCTTCCGCAGGTTCACCTACGGAAACCTTGTTACGACTTCTCCTTCCTCTAAGTGATCAAGTTTGAACAACTTCCCGGCCTCCCACTCTCTGCACGAGACAGAGTGTCGGCGCCAGTCAGGAGGCCTCACTARAYCAYTCAATCGGTAGK

SD6 CCAAACAACCCGACTCTTCGAAAGC?????????GCGGCCGGCCCTGCCACAGACGGGGTTGTCACCCTCTGCGACGTGCCGTTCCAAGCAACTTAGGCASGGCCCTTCCGCCTAGAAAGTGCTTCTCGCAACTACAACTCGCCGATGCAGGCATCGGAGATTTCAAATTTGAGCTCTTCCCGCTTCACTCGCCGTTACTGGGGGAATCCTTGTTAGTTTCTTTTCCTCCGCTTATTAATATGCTTAAATTCAGCGGGTAGCCTTGCCTGATC???????????????????????????????????????????????????????????????????????????????????????????????????????????????????????????????????????????????????-?????????--??????????????????????????????????????????????????????????????????????????????????????????????????????????????????????????????????????????????????????????????????????????????????????????????????????????????????????????AGACACTCAGACAGACATGCTCCTGGGGGAACCCAAGAGCGCCATTTGCGTTCAAAGATTCGATGATTCACTGAATTCTGCAATTCACACTACTTATCGCAGCTGGCTGCGTTCTTCATCGATGCGTGAGCCTAGAGATCCACCGTCAAAA-GTTGTCACAACTTTTT-T----CTTTATGTCAGAACATATTGTCTCTCTGTGCTCAGT-CTTTCCATCAAATGTGT-CACGTGAAAAC-ACCGTCGGGGCAGGAGCCCACCGCNNNNNNNNNNNNNNNNNNNNNNNNNNN-NNNNNNNNNNNNGA--------------------C--NCCTAAAGATTTTTTCAGTCG?????????????????????????????????????????????????????????????????????????????????????????????????????????????????????????????????????????????????????????????????????????????????????????????????????????????????????????????????????????????????????GATCNNNNNNNNNNNNNNNNNNNNNNNNNNNNNNNNNNNNNNNNNNNNNNNCTAAATGATCAAGTTTGAACAACTTCCCGGCCTCCGACTCTCTGCCGGAG-CAGAGTGTCGGCGCCAGTCAGGAGGCCTCACTAAACCATTCAATCGGTAGT

SS1 CCAAACAACCCGACTCTTCGAAAGCAC????????????????????????????????????????????????????????????????????????????????????????????????????????????????????????????GCATCGGAGATTTCAAATTTGAGCTCTTCCCGCTTCACTCGCCGTTACTNGGGGAATCCTTGTTAGTTTCTTTTCCTCCGCTTATTAATATGCTTAAATTCAGCGGGTAGCCTTGCCTGATCBGAGDTCTGGAAGGCGATTCCTTTTTTCCTTTGAGATGCCGCCACCGCTNCCCGGCGGCAGCAGAAAAAAGAATCGAATGGAGAAAGATTTGKTCCGTCAAAGCGATAGAGCCGTGGCCGTTTAGGGTATCTTGTTCTA??????????????????--??????????????????????????????????????????????????????????????????????????????????????????????????????????????????????????????????????????????????????????????????????????????????????????????????????????????????????????AGACACTCAGACAGACATGCTCCTGGGAGAACCCAAGAGCRCCATTTGCGTTCAAAGATTYGAYGATTCACTGAATTCTGCAATTCACACTACTTATYGCAGCTGGCTGCGTTCTTCATCGATGCGTGAGYCTRGAGATCCACCGTCAAA-AGTTGTCACACTTTTTT----CTCT------------TTCNNATCTCACTGTGCTCAGTCTTTCAAAACAAATGTGTGTGTGTCAAAAACACTGTCGGGGCAGGAGCCCACCGCKAAGCTRAGCWWAGMKWRSMYRMRCTAAGMTAAGCTAAGCKARRNWRAGMSA----------CACNCNCAANGAGAAACGTAGGCAGGCACCGACGCGGCGCACGAAGCGTCCTACGCCACACCGCACGGTCACGCTA?GATCCYACTGAGCAGTCAGTCTCCATCCATCTC----CACAAAAGTCAGTTAGCTAGCACGGCAGTCCGAGCGACKCCTCGCGGCCCTCTCCTTTTAACGGAACCAGCCGACCCCTAATGATAATTTTTTCCACTGGTTCCCAGGGTAGAAGAACTCGTAGTACTTAAACTACGTATCTCTCTTTCGGTAAKGATCCTTCCGCAGGTTCACCTACGGAAACCTTGTTACGACTTTTACTTCCTCTAAATGATCAAGTTTGAACAACTTCCCGGCCTCCCACTCTCTGCACGAGGCAGAGTGTCGGCGCCAGTCAGGAGGCCTCACTAAACCATTCAATCGGTAGT

***Pocillopora* Histone Phylip Alignment**

Contig with blast hits to coral histone proteins 2, 3, and 4.

14 4519

S2 AAGAAAGAAAGAAA-GAAAGAAAGAAAGAAAGAAAGAAAGAAAGAAAGAAAGAAAGAACAACATATGAACAATATATGACTTTCATGTATTCTTATCACAAAGAACAACAATGTTGGACATTTACTTTCATGATATGTGGTGGCTCCTAAAGGAGCCGTTTGTTTGTGAGGTCGGGCAGTTCACTTGCTGCTTGTATATTTGGTGACTGCTTTTGTACCCTCGCTTACTGCATGTTTTGCGAGTTCGCCCGGTAACAGTAACCTAATGGCGGTCTGGATTTCGCGAGAACTGATGGTGGACTTCTTGTTGTAGTGCGCGAGCCGCGAGGATTCGGTCGCGATGCGTTCAAAGACATCGTTGACAAACGAGTTCATGATACCCATGGCTTTGCTGGAGATACCGGTGTCGGGGTGCACCTGTTTCAACACTTTGTAGATGTAGATCGCATAGCTCTCCTTTCTCTTGCTTCTCTTCTTTTTCTTGCCAGGGTCTGTCGGGGCTGATTTAGCCTTGCCTGCTCTCTTCTCGCCTTTTCCGGCCGCGGGAACTTTTGGTGCCATGTCTAGAGAAATCTATCACCGTTCTCTCAAAAATAGACTGAGGTGAGTGACTAGACAGGAGAATTTACTCCCTATTTAAGGTCCATTGTTGAGGATCAATTAGTATGCCAACGAAAATTCTCTTTGAAGCGATTGGCTCAGATACAGCTGTGAAAGGAACAAAAGACTGAGAGGCAACAGAGGGCGGAATT-ATTTTGATTGGAGGGTTTCGATCCGTTAGCGCGCGCGAGCCGCGAGATAAGTACGGCGTCACGACTCGGAAGGCGTCACAATTTAGACAACAAAATCGTTGG-ATTTCAGTGTGTGTCCAAGCACGAGCTAGTTTGCGAGAGAGAAAACTAAAATGACCGGCCGAGGGAAAGGAAAAGCGTCGGGCACCAAGGC---AAAGAGCAGGTCCAGCCGAGCAGGTCTTCAGTTTCCAGTCGGCCGCATCCACCGACTCCTTCGCAAAGGAAACTATGCAGAGCGTGTCGGGGCGGGTGCTCCCGTGTACTTGGCCGCTGTGCTCGAGTATCTAAGTGCTGAAATTCTAGAGTTGGCCGGAAACGCCGCTCGTGACAACAAGAAGACCAGAATCATTCCCAGACACCTGCAGTTGGCCGTCCGAAACGACGAGGAATTGAACAAACTTCTAGCTGGTGTAACGATCGCCCAGGGCGGTGTTCTTCCCAACATACAGGCCGTGCTGCTACCCAAGAAGGCCGAGAAGAAGCACCATTGACAACATCAAACT-TCAAAACGGCTCCTTTAGGAGCCACCAAACCTCGAAAAAAGTCGTGATCAGTGAAAAATCCATTTACTCCAATTGACACTCGTACATGCACACACAAGCGCTCGCGTCTTTACTTTGTTATC-GATCAAAACAAATCGTTTCAGTTCACCTAGTGTCCATCGCTTACATTCCGCATTTTTGATCCCGAACTAGTTAAACAAATAAAAAGCAAGT-CAAATCAAACAGAAGGCAAGAAAGACAATTCAAATCAGCTTTGCTTTTT-TTGGAAACATTGCCGGCGACTTTGTTTT-CGTTCGTTTGGTTGGACGATGGTAATTTGGATTTGCAAAA-TGCCTCGACACATTTGCAGCCCGCTACTGTCTCTCTTTCAATTGTTGGAGCGTGGAATTTGCGACTTGCATAAACTGCCTTCCTACCTATCAAGACAGAGAAACGCATTTGAGTTTGAATGGTACGATCGCTGATCCCAAGTTTGCGTGGTTGTTGCAAATCCGCTTTAATCTTTTCTATTCCTTGCCATCCCCGTTCCAAAAAGGAACGAGGTACTTTTCTGCTTCCCAGGTGGGGCTAATTTTGCA-AAGCAAGACAAGCGACTCGAAATGTGTG-GTGTCGGCGTTCTCATAATGATCAGAACTAACGTACTTGGAGAACGGTGCGATCACGCGTGGTTCCATTGCGTGGAAACGTAATAGAATTTGAGTAGAACGTTCGCTGAACCCAGCTTTGCATGGCTTTTGAAACTCCAATTTCCTATTTCCTATTTCCTATTTCCTATTTCCTATTTCCTATTTCCTGCCATCCCAGTTCCCTGTTGGAACGAGAGACTATTTTACACTGCATTAACGCTAACACAAGGACTAGGAGAAACGTTGAGTGAACGCGATCACGCGTGGTTCCATTGCGTGGAAACGTAATAGAATTTGAGTAGAACGTTCGCTGAACCCAGCTTTGCRTGGCTTTTGAAACTCCAATTTCCTATTTCCTATTTCCTATTTCCTATTTCCTATTTCCTGCCATCCCAGTTCCCTGTTGGAACGAGAGACT-ATTTTACACTGCATTAACGCTAACACAAGGACTAGGAGAAACGTTGAGWGMAS--RATAATATCGCCCTTTGACTCGTTGTTCAAACACAGGGTTTGACCATCACTGTGGCTGATCACGACTTTCGCATGAGGTGGTGGCTCCTAAAGGAGCCGTTTATCAGTTCTCTGAGGTTTCCGTGATTCTCTACCCGCCAAAACCGTATAGGGTACGGCCTTGGCGTTTAAGGGCGTAGACCACATCCATTGCGGTAACGGTCTTCCGTTTGGCGTGTTCGGTGTAAGTCACAGCATCACGGATAACGTTTTCCAGGAAGACTTTGAGGACTCCGCGGGTCTCTTCGTAAATAAGCCCGGAAATACGCTTGACACCACCGCGGCGAGCCAGACGTCGGATTGCTGGCTTGGTGATTCCCTGGATGTTGTCTCGCAGAATCTTTCGATGGCGCTTGGCGCCCCCTTTTCCAAGCCCTTTACCTCCTTTGCCGCGTCCAGACATGATTTG--TTTTTCTTTCGAATGTTACGGATGCAATTGTTGTCGGCCCTGAACAAAACTTGAGATGTGTGGCTGGACCTCTCGGGAAACGTCACATTTCCAATTGACAAGAGAGCCAGGCCAAATGCCGACTTATGAATACCATTATCACTTATCTACACGGCCACGCTAAAGGATAGCGAAAATGACTCACAACCTTCAAAGTAATCCATGGTCAAATTCAGTCTCACTCACTCACTCACTCACTCACTCACTCACTCACTAATTAGTTTATTACTTTTTCGAGAGTACTCACTGAAGGCGAGCGACTGAAAACCGAGGGCAGTTTCGAGAAAAATTGCAGGTCAGAGTTTACTAAAATTTACTTTCCGCCTCGTCCTCGGAAAAAAAGAGTACCCGAACAAAGAAATTCACCGCAGCCAATCACAAGCAAACTTTGGATAACCAATCGGATCGTTGAGCGCTTGTTATAAAATCGGTTTTGTCACTGGAGATCGCGAGAAAAGCGTTAAATTTCTGAAG--ATTACTAGACTGAAGCGTCCAATCTTGTTGGCTATGGCTCGTACCAAGCAAACCGCCCGGAAATCAACAGGTGGAAAAGCTCCTCGTAAACAGTTAGCCACTAAGGCGGCCCGCAAGAGTGCTCCCGCTACGGGTGGCGTGAAGAAACCTCACCGTTACAGACCAGGCACAGTTGCACTCCGTGAAATCCGTCGTTATCAGAAGTCTACGGAGTTACTTATCCGCAAGTTGCCCTTTCAACGACTTGTGCGGGAGATCGCGCAGGATTTCAAAACCGATCTGCGATTCCAAAGTTCCGCTGTACTAGCGCTCCAAGAGGCTAGCGAAGCTTATTTGGTGGGTCTGTTCGAAGATACCAATCTGTGCGCCATCCACGCCAAACGCGTAACCATCATGCCGAAGGATATCCAGTTGGCTCGAAGGATTCGTGGCGAGCGGGCTTAGTACAGTCGCTTGGTCATCAAACAAACACTAACGGCTCCTTTAGGAGCCACCACTAACCTAAAGGTCAATGAGCAGCCCATCTCCTTTTACACGTCTTTAATTTTTCTGTGTGTGTTCCGTTTCGTTTTGTTGTGGTTGTTGTTTCATTTTCTTGG-TTTTTGTTACGTGAGTG-AAAAACTCGCATTATTGTCATGAAGCAACAGAGAAAATCAATGACCACGTCTCCAAAGGTGACCCGCCTCTGCGGAAAAA----TGTCGACGTAGAAAAAAAGATATCTTGTCAGAACATTCGAGCGATCGACTCTCGGTTTGATTGGCGACAAAAATTTGACAGAAAGTGAGCAGCGACGGAGGTCAACTGTTTTGATGCGTTTTCCGCGAGTTTGCTCGAACTAGACAAACATAAAGGTCACCATTTTGCGCTTTTAGCCGTTCGTATAATTTGTACCGACGTTACAATTCATTGCCGCTGTCTTTTCTTGTTCCTTACTWGGCAAAAAAAAAAAR--AAAAAAAAGAACAAGAACAAGAACAAGRACAAACAAAYAAACAAACAAACAAGAAACCAGAAAAACAAACAAGCACAGGAACAGCCTCTTTTTAAGCAGGCGCAATTACCTCAATCTCTACGTTATTACCAATATAACCAATTTCACAAATATCGTCAASWTTCAGACGGCTCAGGGAGAATATTTGCT

SD3 AAGAAAGAAAGAAA-GAAAGAAAGAAAGAAAGAAAGAAAGAAAGAAAGAAAGAAAGAACAACATATGAACAATATATGACTTTCATGTATTCTTATCACAAAGAACAACAATGTTGGACATTTACTTTCATGATATGTGGTGGCTCCTAAAGGAGCCGTTTGTTTGTGAGGTCGGGCAGTTCACTTGCTGCTTGTATATTTGGTGACTGCTTTTGTACCCTCGCTTACTGCATGTTTTGCGAGTTCGCCCGGTAACAGTAACCTAATGGCGGTCTGGATTTCGCGAGAACTGATGGTGGACTTCTTGTTGTAGTGCGCGAGCCGCGAGGATTCGGTCGCGATGCGTTCAAAGACATCGTTGACAAACGAGTTCATGATACCCATGGCTTTGCTGGAGATACCGGTGTCGGGGTGCACCTGTTTCAACACTTTGTAGATGTAGATCGCATAGCTCTCCTTTCTCTTGCTTCTCTTCTTTTTCTTGCCAGGGTCTGTCGGGGCTGATTTAGCCTTGCCTGCTCTCTTCTCGCCTTTTCCGGCCGCGGGAACTTTTGGTGCCATGTCTAGAGAAATCTATCACCGTTCTCTCAAAAATAGACTGAGGTGAGTGACTAGACAGGAGAATTTACTCCCTATTTAAGGTCCATTGTTGAGGATCAATTAGTATGCCAACGAAAATTCTCTTTGAAGCGATTGGCTCAGATACAGCTGTGAAAGGAACAAAAGACTGAGAGGCAACAGAGGGCGGAATT-ATTTTGATTGGAGGGTTTCGATCAGATCGGAAGAGCGWSSYGCGAGATAAGTACGGCGTCACGACTCGGAAGGCGTCACAATTTAGACAACAAAATCGTTGG-ATTTCAGTGTGTGTCCAAGCACGAGCTAGTTTGCGAGAGAGAAAACTAAAATGACCGGCCGAGGGAAAGGAAAAGCGTCGGGCACCAAGGC---AAAGAGCAGGTCCAGCCGAGCAGGTCTTCAGTTTCCAGWCGGCCGCATCCACCGACTCCTTC??????????????GCAGAGCGTGTCGGGGCGGGTGCTCCCGTGTACTTGGCCGCTGTGCTCGAGTATCTAAGTGCTGAAATTCTAGAGTTGGCCGGAAACGCCGCTCGTGACAACAAGAAGACCAGAATCATTCCCAGACACCTGCAGTTGGCCGTCCGAAACGACGAGGAATTGAACAAACTTCTAGCTGGTGTAACGATCGCCCAGGGCGGTGTTCTTCCCAACATACAGGCCGTGCTGCTACCCAAGAAGGCCGAGAAGAAGCACCATTGACAACATCAAACT-TCAAAACGGCTCCTTTAGGAGCCACCAAACCTCGAAAAAAGTCGTGATCAGTGAAAAATCCATTTACTCCAATTGACACTCGTACATGCACACACAAGCGCTCGCGTCTTTACTTTGTTATC-GATCAAAACAAATCGTTTCAGTTCACCTAGTGTCCATCGCTTACATTCCGCATTTTTGATCCCGAACTAGTTAAACAAATAAAAAGCAAGT-CAAATCAAACAGAAGGCAAGAAAGACAATTCAAATCAGCTTTGCTTTTT-TTGGAAACATTGCCGGCGACTTTGTTTT-CGTTCGTTTGGTTGGACGATGGTAATTTGGATTTGCAAAA-TGCCTCGACACATTTGCAGCCCGCTACTGTCTCTCTTTCAATTGTTGGAGCGTGGAATTTGCGACTTGCATAAACTGCCTTCCTACCTATCAAGACAGAGAAACGCATTTGAGTTTGAATGGTACGATCGCTGATCCCAAGTTTGCGTGGTTGTTGCAAATCCGCTTTAATCTTTTCTATTCCTTGCCATCCCCGTTCCAAAAAGGAACGAGGTACTTTTCTGCTTCCCAGGTGGGGCTAATTTTGCA-AAGCAAGACAAGCGACTCGAAATGTGTG-GTGTCGGCGTTCTCATAATGATCAGAACTAACGTACTTGGAGAACGGTGCGATCACGCGTGGTTCCATTGCGTGGAAACGTAATAGAATTTGAGTAGAACGTTCGCTGAACCCAGCTTTGCATGGCTTTTGAAACTCCAATTTCCTATTTCCTATTTCCTATTTCCTATTTCCTATTTCCTATTTCCTGCCATCCCAGTTCCCTGTTGGAACGAGAGACTATTTTACACTGCATTAACGCTAACACAAGGACTAGGAGAAACGTTGAGTGAACGCGATCACGCGTGGTTCCATTGCGTGGAAACGTAATAGAATTTGAGTAGAACGTTCGCTGAACCCAGCTTTGCGTGGCTTTTGAAACTCCAATTTCCTATTTCCTATTTCCTATTTCCTATTTCCTGCCAYCYSMCATCCCAGTTCCCTGTTGGAACGAGAGACT-ATTTTACACTGCATTAACGCTAACACAAGGACTAGGAGAAACGTTGAGAGCAG--AATAATATCGCCCTTTGACTCGTTGTTCAAACACAGGGTTTGACCATCACTGTGGCTGATCACGACTTTCGCATGAGGTGGTGGCTCCTAAAGGAGCCGTTTATCAGTTCTCTGAGGTTTCCGTGATTCTCTACCCGCCAAAACCGTATAGGGTACGGCCTTGGCGTTTAAGGGCGTAGACCACATCCATTGCGGTAACGGTCTTCCGTTTGGCGTGTTCGGTGTAAGTCACAGCATCACGGATAACGTTTTCCAGGAAGACTTTGAGGACTCCGCGGGTCTCTTCGTAAATAAGCCCGGAAATACGCTTGACACCACCGCGGCGAGCCAGACGTCGGATTGCTGGCTTGGTGATTCCCTGGATGTTGTCTCGCAGAATCTTTCGATGGCGCTTGGCGCCCCCTTTTCCAAGCCCTTTACCTCCTTTGCCGCGTCCAGACATGATTTG--TTTTTCTTTCGAATGTTACGGATGCAATT??????????????????????????????????CTGGACCTCTCGGGAAACGTCACATTTCCAATTGACAAGAGAGCCAGGCCAAATGCCGACTTATGAATACCATTATCACTTATCTACACGGCCACGCTAAAGGATAGCGAAAATGACTCACAACCTTCAAAGTAATCCATGGTCAAATTCAGTCTCACTCACTCACTCACTCACTCACTCACTCACTCACTAATTAGTTTATTACTTTTTCGAGAGTACTCACTGAAGGCGAGCGACTGAAAACCGAGGGCAGTTTCGAGAAAAATTGCAGGTCAGAGTTTACTAAAATTTACTTTCCGCCTCGTCCTCGGAAAAAAAGAGTACCCGAACAAAGAAATTCACCGCAGCCAATCACAAGCAAACTTTGGATAACCAATCGGATCGTTGAGCGCTTGTTATAAAATCGGTTTTGTCACTGGAGATCGCGAGAAAAGCGTTAAATTTCTGAAG--ATTACTAGACTGAAGCGTCCAATCTTGTTGGCTATGGCTCGTACCAAGCAAACCGCCCGGAAATCAACAGGTGGAAAAGCTCCTCGTAAACAGTTAGCCACTAAGGCGGCCCGCAAGAGTGCTCCCGCTACGGGTGGCGTGAAGAAACCTCACCGTTACAGACCAGGCACAGTTGCACTCCGTGAAATCCGTCGTTATCAGAAGTCTACGGAGTTACTTATCCGCAAGTTGCCCTTTCAACGACTTGTGCGGGAGATCGCGCAGGATTTCAAAACCGATCTGCGATTCCAAAGTTCCGCTGTACTAGCGCTCCAAGAGGCTAGCGAAGCTTATTTGGTGGGTCTGTTCGAAGATACCAATCTGTGCGCCATCCACGCCAAACGCGTAACCATCATGCCGAAGGATATCCAGTTGGCTCGAAGGATTCGTGGCGAGCGGGCTTAGTACAGTCGCTTGGTCATCAAACAAACACTAACGGCTCCTTTAGGAGCCACCACTAACCTAAAGGTCAATGAGCAGCCCATCTCCTTTTACACGTCTTTAATTTTTCTGTGTGTGTTCCGTTTCGTTTTGTTGTGGTTGTTGTTTCATTTTCTTGG-TTTTTGTTACGTGAGCG-AAAAACTCGCATTATTGTCATGAAGCAACAGAGAAAATCAATGACCACGTCTCCAAAGGTGACCCGCCTCTGCGGAAAAA----TGTCGACGTAGAAAAAAAGATATCTTGTCAGAACATTCGAGCGATCGACTCTCGGTTTGATTGGCGACAAAAATTTGACAGAAAGTGAGCAGCGACGGAGGTCAACTGTTTTGATGCGTTTTCCGCGAGTTTGCTCGAACTAGACAAACATAAAGGTCACCATTTTGCGCTTTTAGCCGTTCGTATAATTTGTACCGACGTTACAATTCATTGCCGCTGTCTTTTCTTGTTCCTTACTTGGCAAAAAAAAAAAA--AAAAAAAAGAACAAGAACAAGAACAAGGACAAACAAACAAACAAACAAACAAACAA?????????????????????????????????????????????????????????????????????????????????????????????????????????????????????????????????????

P53 AAGAAAGAAAGAAA-GAAAGAAAGAAAGAAAGAAAGAAAGAAAGAAAGAAAGAAAGAACAACA-ATGTTGGATAT??????????????????????????????CAACAATGTTGGACATTTACTTTCAGGACATGTGGTGGCTCCTA???????????????????????????????????TTGCTGCTTGTATATTTGGTGACTGCTTTTGTACCCTCGCTTACTGCATGTTTTGCGAGTTCGCCCGGTAACAGTAGCCTAATGGCGGTCTGGATTTCGCGAGAGCTGATGGTGGACTTCTTGTTGTAGTGCGCGAGCCGCGAGG????????????????????????????????????????????????????????????????????????????????????????????????????????????????GATCGCATAGCTCTCCTTTCTCTTGCTTCTCTTCTTTTTCTTGCCAGGGTCTGTCGGGGCTGATTTAGCCTTGCCGGCTCTCTTCTCGCCTTTTCCGGCCGCGGGAACTTTTGGTGCCATGTCTAGAGAAATCTATCACCGTTCTCTCAAAAATAGACTGAGGTGAGTGACTAGACAGGAGAATTTACTCCCTATTTAAGGTCCATTGTTGAGGATCAATTAGTATGCCAACGAAAATTCTCTTTGAAGCGATTGGCTCAGATACAGCTGTGAAAGGAACAAAAGACTGAGAGGCAACAGAGGGCGGAATT-ATTTTGATTGGAGGGTTTCGATCCGTTAGCGCGCGCGAGCCGCGAGATAAGTACGGCGTCACGACTCGKAACGCGTCACAATTTAGACGACAGAATCGTTGG-ATTTCAGTGTGTGTCCTAGCACGAGCTAGTTTGCGAGAGAGAAAACTAAAATGACCGGTCGAGGGAAAGGAAAAGCGTCAGGC????????---?????????????????????????????????????????????????????????????????????????????????????????????????????????????????????????????????????????????????????????????????????????????????????????????????????????????????????????????????????????????????????????????????????GATCGCCCAGGGCGGTGTTCTTCCCAACATACAGGCCGTGCTGCTACCCAAGAAGGCCGAGAAGAAGCACCATTGACAACATCAAACT-TCAAAACGGCTCCTTTAGGAGCCACCAAACCTCGAAAAAAGTCGTGATCAGTGAAAAATCCATTTACTCCAATTGACACTCGTACATGCACACACAAGCGCTCGCGTCTTTACTTTGTTATC-GATCAAAACAAATCGTTTCAGTTCACCTAGTGTCCATCGCTTACATTCCGCATTTTTGATCCCAAACTAGTTAAACAAATAAAAAGCAAGT-CAAATCAAACAGACGGAAAGAAAGACAATTCAAATCAGCTTTGCTTTTT-TTGGAAACATTGCCGGCGACTTTGTTTT-CGTTCGTTTGGTTGGACGATGGTAATTTGGATTTGCAAAA-TGCCTCGACACATTTGCAGCCCGCTACTGTCTCTCTTTCAATTTTTGGAGCGTGGAATTGGCGGCTTGCATAAACTGCCTTCCTACCTATCAAGACAGAGAAACGCATTTGAGTTTGAATGGTACGATCGCTGATCACAAGTTTGCGTGGTTGTTGCAAATCCGCTTTAATCTTTTCTATTCCTTGCCATCCCCGTTCCARTAAGGAACGAGGTACTTTTCTGCTTCCCAGGTGGGGCTAATTTTGCA-AAGCAAGACAAGCGACTCGAAATGTGTG-GTGTCGGCGTTCTCATAATGATCAGAACTAACGTACTTGGAGAACGGTGCGATCACGCGTGGTTCCATTGCGTGGAAACGTAATAGAATTTGAGTAGAACGTTCGCTGAACCCAGCTTTGCATGGCTTTTGAAACTCCAATTTCCTATTTCCYATTTCCYATTTCCTATTTCCTATTTCCTATTTCCTGCCATCCCAGTTCCC??????????????????????????????????????????????????????????????????????????????????????????????????????????????????????????????????????????????????????????????????????????????????????????????????????????????????????????-??????????????????????????????????????????????????????????????????????--????????????????????????????????ACAGGGTTTGACCATCACTGTGGC???????????????????????????GCTCCTAAAGGAGCCGTTTATCAGTTCTCTGAGGTTTCCGTGATTCTCTACCCGCCAAAACCGTATAGGGTACGGCCTTGGCGTTTAAGGGCGTAGACCACATCCATTGCGGTAACGGTCTTCCGTTTGGCGTGTTCGGTGTAAGTCACAGCATCACGGATAACGTTTTCCAGGAAGACTTTGAGGACTCCGCGGGTCTCTTCGTAAATAAGCCCGGAAATACGCTTGACACCACCGCGGCGAGCCAGACG??????????????????????????????--??????????????????????????????????????????????????????????????????????????????????????????????????????????????????????????????????????????????????????????????????????????????????????????????????????????????????????????????????????????????????????AAGGATAGCGAAAATGACTCACAACCTTCAAAGTAATCCATGGTCAAATTCAGTCTCACAGTCTCACTCACTCACTCACTCACTCACTCACTAATTAGGTTATTACTTTTTCCAGAGTACTCACTGAAGGCGAGCGACTGAAAACCGAGGGCAGTTTCGAGAAAAATTGCAGGTCAGAGTTTACTAAAATTTACTTTCCGCCTCGTCCTCGGAAAAAAAGAGTACCCGAACAAAGAAATTCACCGCAGCCAATCACAAGCAAACTTTGGATAACCAATCGGATCGTTGAGCGCTTGTTATAAAATAGGTTTTGTCACTGGAGATCGCGAGAAAAGCGTAAAATTTCTGAAG--ATTACTAGACTGAAGCGTCCAATCTTGTTGGCTATGGCTCGTACCAAGCAAACCGCCCGGAAATCAACAGGTGGAAAAGCTCCTCGTAAACAGTTAGCCACTAAGGCGGCCCGCAAGAGTGCTCCCGCTACGGGTGGCGTGAAGAAACCTCACCGTTACAGACCAGGCACAGTTGCACTCCGTGAAATCCGTCGTTATCAGAAGTCTACGGAGTTACTTATCCGCAAGTTGCCCTTTCAACGGCTTGTGCGGGAGATCGCGCAGGATTTCAAAACCGATCTGCGATTCCAAAGTTCCGCTGTACTAGCGCTCCAAGAGGCTAGCGAAGCTTACTTGGTGGGTCTGTTCGAAGATACCAATCTGTGCGCCATCCACGCCAAACGCGTAACCATCATGCCGAAGGATATCCAGTTGGCTCGAAGGATTCGTGGCGAGCGGGCTTAGTACAGTCGCTTGGTCATCAAACAAACACTAACGGCTCCTTTAGGAGCCACCACTAACYTAAAGGTCAATGAGCAGCCCATCTCCTTTTACACGTCTATAATTTTTCTGTGTGTGTTCCGTTTCGTTTTKTTGTGGTTGTTGTTTCATTTTCTTGGTTTTTTGTTACGTGAGTG-AAAAACTTGCATTATTGTCACGAAGCAACAGAGAAAATCGATGACCGCATCTCCAAAGGTGACCTGCCTCTGCGGAAAAA----TGTCGACGTAGAAAAAAAGATATCTTGTCAGAACATTCGAGCGATCGACTCTCGGTTTGATTCGCGACAAAAATTTGAC??????????????????????????????????????????????????????????????????????????????????????????????????????????????????????????????????????????????????????????????????????????????????????????????AGAAAA--------AACAAACAAACAAACAAACAAACAAAAA??????????????????????????????????????????????????????????????????????????????????????????????????????????????????????????????????????

R16 AAGAAAGAAAGAAA-GAAAGAAAG????----??????????????????????????????????????AATATATGACTTTCATGTATTCTTATCACAC??????????????????????????????????????????????????????????????????????????????????????????????????????????????TTTGTACCCTCGCTTACTGCATGTTTTGCGAGTTCGCCCGGTAACAGTAGCCTAATGGCGGTCTGGATTTCGCGAGAGCTGATGGTGGACTTCTTGTTGTAGTGCGCGAGCCGCGAGGATTCGGTCGCGATGCGTTCAAAGACATCGTTGACAAACGAGTTCATGATACCCATGGCTTTGCTGGAGATACCGGTGTCGGGGTGCACCTGTTTCAACACTTTGTAGATGTAGATCGCATAGCTCTCCTTTCTCTTGCTTCTCCTCTTTTTCTTGCCAGGGTCTGTCGGGGCTGATTTAGCCTTGCCGGCTCTCTTCTCGCCTTTTCCGGCCGCGGGAACTTTTGGTGCCATGTCTAGAGAAATCTATCACCGTTCTCTCAAAAATAGACTGAGGTGAGTGACTAGACAGGAGAATTTACTCCCTATTTAAGGTCCATTGTTGAGGATCAATTAGTATGCCAACGAAAATTCTCTTTGAAGCGATTGGCTCAGATACAGCTGTGAAAGGAACAAAAGACTGAGAGGCAACAGAGGGCGGAATT-ATTTTGATTGGAGGATTTCGATCGATCWSCKSGMGCGAGCMGCGAGATAAGTACGGCGTCACGACTCGGAACACGTCACAATTTAGACAACAAAATCGTTGG-ATTTCAGTGTGTGTCCTAGCACGAGCT---TTGCGAGAGAGAAAACTAAAATGACCGGTCGAGGGAAAGGAAAAGCGTCGGGCACCAAGGC---AAAGAGCAGGTCCAGCCGAGCAGGTCTTCAGTTTCC????????????????????????TCGCAAAGGAAACTATGCAGAGCGTGTCGGGGCGGGTGCTCCCGTGTACTTGGCCGCTGTGCTCGAGTATCTAAGTGCTGAAATTCTAGAGTTGGCCGGAAACGCCGCTCGTGACAACAAGAAGACCAGAATCATTCCCAGACACCTGCAGTTGGCCGTCCGAAACGACGAGGAGTTGAACAAACTTCTAGCT????????GATCGCCCAGGGCGGTGTTCTTCCCAACATACAGGCCGTGCTGCTACCCAAGAAGGCCGAGAAGAAGCACCATTGACAACATCAAACT-TCAAAACGGCTCCTTTAGGAGCCACCAAACCTCGAAAAAAGTCGTGATCAGTGAAAAATCCATTTACTCCAATTGACACTCGTACATGCACACACAAGCGCTCGCGTCTTTACTTTGTTATC-GATCAAAACAAATCGTTTCAGTTCACCTAGTGTCCATCGCTTACATTCCGCATTTTTGATCCCAAACTAGTTAAACAAATAAAAAGCAAGT-CAAATCAAACAGACGGAAAGAAAGACAATTCAAATCAGCTTTGCTTTTT-TTGGAAACATTGCCGGCGACTTTGTTTT-CGTTCGTTTGGTTGGACGATGGTAATTTGGATTTGCAA??-TGCCTCGACACATTTGCAGCCCGCTACTGTCTCTCTTTCAATTGTTGGAGCGTGGAATTTGCGGCTTGCATAAACTGCCTTCCTACCTATCAAGACAGAGAAACGCATTTGAGTTTGAATGGTACCATCGCTGATCACAAGTTTGCGTGGTTGTTGCAAATCCGCTTTAATCTTTTCTATTCCTTGCCATCCCCGTTCCAATAAGGAACGAGGTACTTTTCTGCTTCCCAGGTGGGGCTAATTTTGCA-AAGCAAGACAAGCGACTCGAAATGTGTG-GTGTCGGCGTTCTCATAATGATCAGAACTAACGTACTTGGAGAACGGTGCGATCACGCGTGGTTCCATTGCGTGGAAACGTAATAGAATTTGAGTAGAACGTTCGCTGAACCCAGCTTTGCATGGCTTTTGAAACTCCAATTTCCTATTTCCTATTTCCCATTTCCCATTTCCCATTTCCCATTTCCCATTNYCCCATTTC????????????????????????????????????????????????????????????????????????????????????????????TGCGTGGAAACGTAATAGAATTTGAGTAGAACGTTCGCTGAACCCAGCTTTGCATGGCTTTTGAAACTCCAATTTCCYATTTCCYRTTTCCYATTTCCCATTTCCYATTTCCYRYYATCCCAKTTCCCTGYWKCGACAAGCGACTCRRGTTACACTGCATTAACGCTAACACAAGGACTAGGAGAAACGTTGAGAGCAG--AATAATATCGCCCTTTGACTCGTTGTTCAAACACAGGGTTTGACCATCAC??GATCTGATCACGACTTTCGCATGAGGTGGTGGCTCCTAAAGGAGCCGTTTATCAGTTCTCTGAGGTTTCCGTGATTCTCTACCCGCCAAAACCGTATAGGGTACGGCCTTGGCGTTTAAGGGCGTAGACCACATCCATTGCGGTAACGGTCTTCCGTTTGGCGTGTTCGGTGTAAGTCACAGCATCACGGATAACGTTTTCCAGGAAGACTTTGAGGACTCCGCGGGTCTCTTCGTAAATAAGCCCGGAAATACGCTTGACACCACCGCGGCGAGCCAGACGTCGGATTGCTGGCTTGGTGATTCCCTGGAT??????????????????????????????????????????????????????????????????????????????????????????????????????????????????????????????????????????????????????????????????????????????????????????????????????????????????????????????????????????????????????????????????????????????????????????????????????????????????????????????????????????????????????????????????--??????????????????????????????????????????????????????????????????????????????????????????????????????????????????????????????????????????????????????????????????TAACCARWYSGATCGTTGAGCGCTTGTTATAAAATCGGTTTTGTCACTGGAGATCTCGAGAAAAGCGTAACATTTCTGAAG--ATTACTAGACTGAAGCGTCCAATCTTGTTGGCTATGGCTCGTACCAAGCAAACCGCCCGGAAATCAACAGGTGGAAAAGCTCCTCGTAAACAGTTAGCCA??????????CCGCAAGAGTGCTCCCGCTACGGGTGGCGTGAAGAAACCTCACCGTTACAGACCAGGCACAGTTGCACTCCGTGAAATCCGTCGTTATCAGAAGTCTACGGAGTTACTTATCCGCAAGTTGCCCTTTCAACGGCTTGTGCGGGAGATC?????????????????????????????????????????????????????????????????????????????????????????????????CCAATCTGTGCGCYATCCACGCCAAACGTGTAACCATCATGCCGAAGGATATCCAGTTGGCTCGAAGGATTCGTGGC???????????????????????GGTCATCAAACAAACACTAACGGCTCCTTTAGGAGCCACCACTAACCTAAAGGTCAATGAGCAGCCCATCTCCTTTTACACGTCTATAATTTTTCTGTGTGTGTTCCGTTTCGTTTTGTTGTGGTTGTTGTTTCATTTTCTTGGTTTTTTGTTACGTGAGTGAAAAAACTCGCATTATTGTCACGAAGCAACAGAGAAAATCGATGACCACATCTCCAAAGGTGACCTGCCTCTGCGGAAAAA----TGTCGACGTAGAAGAAAAGATATCTTGTCAGAACATTCGAGCGATCGACTCTCGGTTTGATTCGCGACAAAAATTTGACAGAAAGTGAGCAGCGACGGAGGTCGACTGTTTTGATGCGTTTTCCGCGAGTTTGTTTGAACTAGACAAACATAAAGGTCACCATTATGCGCTTTCAGCCGTTCGTATAA??????????????????????????????????????????????????????????????????????GATCAAGRWCAAGGACA--------AACAAACAAACAAACAAACAAACAAGAAACCAGAAAAACAAACAAGCACAGGAACAGCCTCTTTTTAAGCAGGCGCAATTACCTCAATC?????????????????????????????????????????????????????????????????????????

Pacu01 AAGAAAGAAAGAAA-GAAAGAAAGAAAG----AAAGAAAGAAAGAAAGAAAGAAAG?????????????????ATATGACTTTCATGTATTCTTATCACACCT?????????GTTGGACATTTACTTTCATGATATG????????????????????????????ATTAGGTCGGGCAGTTCACTTGCTGCTTGTATATTTGGTGACTGCTTTTGTACCCTCGCTTACTGCATGTTTTGCGAGTTCGCCCGGTAACAGTAACCTAATGGCGGTCTGGATTTCGCGAGAGCTGATGGTGGACTTCTTGTTGTAGTGYGCGAGCCGCGAGGATTCGGTCGCGATGCGTTCAAAGACATCGTTGACAAACGAGTTCATGATACCCATGGCTTTGCTGGAGATACCGGTGTCGGGGTGCACCTGTTTCGACACTTTGTAGATGTAGATCGCATAGCTCTCCTTTCTCTTGCTTCTCTTCTTTTTCTTGCCAGGGTCTGTCGGGGCTGATTTAACCTTGCCGGCTCTCTTCTCGCCTTTTCCGGCCGCGGGAACTTTTGGTGCCATGTCTAGAGAAATCTATCACCGTTCTCTCAAAAATAAACTGAGGTGAGTGACTAGACAGGAGAATTTACTCCCTATTTAAGGTCCATTGTTGAGGATCAATTAGTATGCCAACGAAAATTCTCTTTGAAGCGATTGGTTCAGATACAGCTGTGAAAGGAACAAAAGACTGAGAGGCAACAGAGGGCGGAATT-ATTTTGATTGGAGGGTTTCGATCCGTTAGCGCGCGCGAGCCGCGAGATAAGTACGGCGTCACGACTCGGAACGCGTCACAATTTAGACAACAAAATCGTTGGAATTTTAGTGTGTGTCCTAGCACGAGCTGGCTTGCGAGAGAGAAAACTAAAATGACCGGCCGAGGGAAAGGAAAAGCGTCGGGCACCAAGGCAAAAAAGAGCAGGTCCAGCCGAGCAGGTCTTCAGTTTCCAGTCGGCCGCATCCACCGACTCCTTCGCAAAGGAAACTATGCAGAGCGTGTCGGGGCGAGTGCTCCCGTATACTTGGCCGCTGTGCTCGAGTATCTAATTGCTGAAATTCTAGAGTTGGCCGGAAACGCCGCTCGTGACAACAAGAAGAGCAGAATCATTCCCAGACACCTGCAGTTGGCCGTCCGAAACGACGAGGAATTGAACAAACTTCTAGCTGGTGTAACGATCGCCCAGGGCGGTGTTCTTCCCAACATACAGGCCGTGCTGCTACCCAAGAAGGCCGAGAAGAAGCACCATTGACAACATCAAACT-TCAAAACGGCTCCTTTAGGAGCCACCAAACCTCGAAAAACGATCTGATCAGTGAAAAATCCATTTACTCCAATTGACACTCGTACATRCACACACAAGCGCTCGCGTCTTTACTTTSYKATCTGATCAAAACAAATCGTTTCAGTTCACCTAGTGTCCATCGCTTACATGCCGCATTTTTGATCAGATACTAGTTAAACAAATAAAAAGCAAGT-CAAATCAAACAGACGGAAAGAAAGACAATTCAAATCAGCTTTGCTTTTTTTTGGAAACATTGCCGGCGACTTTGTTTT-CGTTCGTTTGGTTGGACGATGGTAATTTGGATTTGCAAAA-TGCCTCGACACATTTGCAGCCCGCTACTGTCTCTCTTTCAATTGTTGGAGCGTGGAATTTGCGACTTGCATAAACTGCCTTCCTACCTATCAAGACAGAGAAACGCATTTGAGTTTGAATGGTACGATC???????????????GCGTGGTTGTTGCAAAYCCGCTTTAATCTTTTCTATTCCTTKSCATCCCCGTTCCAATAAGGAACGAGGTACTTTTCTGCTTCCTAGGT???????????????-????????????????????????????-?????????????????????????????????????????????????????????????ATTCCTTTGCGTGGAAMMGTAATAGAATTTGAGTRGAACGTTCGCTGAACCCAGCTTTGCATGGCTTTTGAAACTCCAATTTMCTAYTTCCTATTTCCTAT???????????????????????????????????????????????????????????????????????????????????????????????????????????????????????????????????????????????????????????????????????????????????????????????????????????????????????????????????????????????????????????????????-??????????????????????????????TTAACGCTAACACAAGGACTAGGAGAAACGTTGAGAGCAG--AAYAATATCGCCCTTTGACTCGTYGTTCAAACACAGGGTTTGACCATCACTGTGGCTGATCASRACTTTCGCATGAGGTGGTGGCTCCTAAAGGAGCCGTTTATCAATTCTCTGAGGTTTCCGTGATTCTCTACCCGCCAAAACCGTATAGGGTACGGCCTTGGCGTTTAAGGGCGTAGACCACATCCATTGCGGTGACGGTCTTCCGTTTGGCGTGTTCGGTGTAAGTCACAGCATCAC?GATAACGTTTTCCAGGAAGACTTTGAGGACTCCGCGGGTCTCTTCGTAAATAAGCCCGGAAATACGCTTGACACCACCG?GGCGAGCCAGACGTCGGATTGCTGGCTTGGTGATTCCCTGGATGTTGTCTCGCAGAATCTTTCGATGGCGCTTGGCGCCCCCTTTTCCAAGCCCTTTACCT????????????????????????????????????????????????????????????????????????????????????????????????????????????????????????????????????????????????????????????????????????????????????????????????????????????????????????????????????????????????????????????????????????????????????????????????????????--??????????????????????????????????????????????????????????????????????????????????????????????????????????????????????????????????????????????????????????????????????????????????????????????ATAAAATCGGTTTTGTCACTGGAGATCGGGATAAAAGCGTAAAATTTCTGAAG--ATTACTAGACTGAAGCGTCCAATCTCGTTGGCTATGGCTCGTACCAAGCAAACCGCCCGGAAATCAACAGGTGGAAAAGCTCCTCGTAAACAGCTAGCCACTAAGGCGGCCCGCAAGAGTGCTCCCGCTACGGGTGGCGTGAAGAAACCTCACCGTTACAGACCAGGCACAGTTGCACTCCGTGAAATCCGTCGTTATCAGAAGTCTACGGAGTTACTTATCCGCACGTTGCCCTTTCAACGGCTTGTGCGGGAGATCGCGCAGGWCTTCCGATCTGATCTGCGATTCCAAAGTTCCGCTGTACTAGCGCTCCAAGAGGCTAGCGAAGCTTATTTGGTGGGTCTGTTCGAAGATACCAATCTGTGCGCCATCCACGCCAAACGCGTAACCATCATGCCGAAGGATATCCAGTTAGCTCGAAGGATTCGTGGCGAGCGGGCTTAGTACAGTCGCTTGGTCATCAAACAAACAC???????????????????CACCACTAACCTAAAGGTCAATGAGCAGCCCATCTCCTTTTACACGTCTTTAATTTTTCTGTGTGTGTTCCGTTTCGTTTTGTTGT??????????????????????????????????????????????????????????????????????????????????????????????????????????????????????????????????????????????????????------???????????GATCGACTCTCGGTTTGATTGGCGACAAAAATTTGACAGAAAGTGAGCAGCGACGGAGGTCAACTGTTTTGATGCGTTTTCCGCGAGTTTGCTTGAACTAGACAAACATAAAGGTCACCATTTTGCGCTTTCAGCCGTTCGTAT??------???????????????????????????????????????????????????????????????AGAACAAGAACAAGGACA--AACAANNACAAACAAACAAACAAACAAACAAGAAACCAGAAAAACAAACAAGCACAGGAACAGCCTCTTTTTAAGCAGGCGCAATTACCTCAATCTCTAC????????????????????????????????????????????????????????????????????

Pacu02 AAGAAAGAAAGAAA-G??????????????????????????????????????????????????????AATATATGACTTTCATGTATTCTTATCAGAAACAACAACAATGTTGGACATTTRCTTTCATGATATGTGGTGGCTCCTGAAGGAGCCGTTTGTTTGTGAGGTCGGGCAGTTCACTTGCTGCTTGTATATTTGGTGACTGCTTTTGTACCCTCGCTTACTGCATGTTTTGCGAGTTCGCCCGGTAACAGTAACCTAATGGCGGTCTGGATTTCGCGAGAGCTGATGGTGGACTTCTTGTTGTAGTGCGCGAGCCGCGAGGATTCGGTCGCGATGCGTTCAAAGACATCGTTGACAAACGAGTTCATGATACCCATGGCTTTGCTGGAGATACCGGTGTCGGGGTGCACTTGTTTCAACACTTTGTAGATGTAGATCGCATAGCTCTCCTTTCTCTTGCTTCTCTTCTTTTTCTTGCCAGGGTCTGTCGGGGCTGATTTAGCCTTGCCGGCTCTCTTCTCGCCTTTTCCGGCCGCGGGAACTTTTGGTGCCATGTCTAGAGAAATCTATCACCGTTCTCTCAAAAATAAACTGAGGTGAGTGACTAGACAGGAGAATTTACTCCCTATTTAAGGTCCATTGTTGAGGATCAATTAGTATGCCAACGAAAATTCTCTTTGAAGCGATTGGCTCAGATACAGCTGTGAAAGGAACAAAAGACTGAGAGGCAACAGAGGGCGGAATT-ATTTTGATTGGAGGGTTTCGATCCGTTAGCGCGCGCGAGCCGCGAGATAAGTACGGCGTCACGACTCGGAACGCGTCACAATTTAGACAACAAAATCGTTGGAATTTYAGTGTGTGTCCTAGCACGAGCTRGYTTGCGAGAGAGAAAACTAAAATGACCGGCCGAGGGAAAGGAAAAGCGTCGGGCACCAAGGC---AAAGAGCAGGTCCAGCCGAGCAGGT?????????????TCGGCCGCATCCACCGACTCCTTCGCAAAGGAAACTATGCAGAGCGTGTCGGGGCGGGTGCTCCCGTRTACTTGGCCGCTGTGCTCGAGTATCTAAGTGCTGAAATTCTAGAGTTGGCCGGAAACGCCGCTCGTGACAACAAGAAGACCAGAATCATTCCCAGACACCTGCAGTTGGCCGTCCGAAACGACGAGGAATTGAACAAACTTCTAGCTGGTGTAACGATCGCCCAGGGCGGTGTTCTTCCYAACATACAGGCCGTGCTGCTACCCARGAAGGCCGAGAAGAAGCACCATTGACAACATCAAACT-TCAAAACGGCTCCTTTAGGAGCCACCAAACCTCGAAAAAAGTCCTGATCAGTGAAAAATCCATTTACTCCAATTGACAYTCGTACATGCACACACAAGCGCTCGCGTCTTTACTTTGTTATC-GATCAAAACAAATCGTTTCAGTTCACCTAGTGTCCATCGCTTACATGCCGCATTTTTGATCMCGAWCKRRWTAAACAAATAAAAAGCAAGT-CAAATCAAACAGACGGAAAGAAAGACAATTCAAATCAGCTTTGCTTTTTTTTGGAAACATTGCCGGCGACTTTGTTTT-CGTTCGTTTGGTTGGACGATGGTAATYTGGATTTGCAAAATTGCCTCGACACATTTGCAGCCCGCTACTGTCTCTCTTTCCATTGCTGGAGCGTGGAATTTGCGACTTGCATAAACTGCCTTCCTACCTATCAAGACAGAGAAACGCATTTGAGTTTGAATGGTACGATMGCTGATCASAAGTTTGCGTGGTTGTTGCAAATCCGCTTTAATCTTTTCTATTCCTTGCCATCCCCGTTCCAATAAGGAACGAGGTACTTTTCTGCTTCCCAGGTGGGGCTAATTTTGCA-AAGCAAGACAAGCGACTCGAAATGTGTG-GTGTCGGCGTTCTCATAATGATCAGAACTAACGTACTT??????????????????????????????TGCGTGGAAACGTAATAGAATTTGAGTAGAACGTTCGCTGAACCCAGCTTTGCATGGCTTTTGAAACTCCAATTTCCTATTTCCTATTTCCTATTTCCTATTTCCTATTTCCTATTTCCTGCCATCCCAGTTCCCTGTTGGAACGAGAGACTATTTTGCACACCA??????????????????????????????????????????????????????????????????????????????????????????????????????????????????????????????????????????????????????????????????????????????????????????????????GTCAAAGGGCG-ATATTACACTGCATTAACGCTAACACAAGGACTAGGAGAAACGTTGAGAGCAG--AATAATATCGCCCTTTGACTCGTTGTTCAAACACAGAGTTTGACCATCAC?????????????????TTCGCATGAGGTGGTGGCTCCTAAAGGAGCCGTTTATCAGTTCTCTGAGGTTTCCGTGATTCTCTACCCGCCAAAACCGTATAGGGTACGGCCTTGGCGTTTAAGGGCGTAGACCACATCCATTGCGGTAACGGTCTTCCGTTTGGCGTGTTCGGTGTAAGTCACAGCATCACGGATAACGTTTTCCAGGAAGACTTTGAGGACTCCGCGGGTCTCTTCGTAAATAAGCCCGGAAATACGCTTGACACC?????????????????????GATTGCTGGCTTGGTGATTCCCTGGATGTTGTCTCGCAGAATCTTTCGATGGCGCTTGGCGCCCCCTTTTCCAAGCCCTTTACCTCCTTTGCCGCGTCCAGACATGATTTG--TTTTTCTTTCGAATGTTACGGATG???????????????CTGAACAAAACTTGAGATGTGTGGCTGGACCTCTCGGGAAACGTCACATTTCCAATTGACAAGAGAGCCAGGCCAAATGCCGACTTATGAATACCATTATCACTTATCTACAC??????????????????????????????????????????????????????CTCACTCACTCTCACTCACTCACTCACTCACTCACTCACTCACTCACTCACTCGTTTATTACTTTTTCGAGAGTACTCACTGAAGGCGAGCGACTGAAAACCGAGGGCAGTTTCGAGAAAAATTGCAGGTCAGAGTTTACTAAAATTTACTTTCCGCCTMGTCCTCGGAAAAAAAGAGTACCCGAACAAAGAAATTCACCGCAGCCAATCACAAGCAAACTTTGGATAACCAATCGGATC????????????????????????????????CTGGAGATCGGGATAAAAGCGTAAAATTTCTGAAG--ATTACTAGACTGAAGCGTCCAATCTCGTTGGCTATGGCTCGTACCAAGCAAACCGCCCGGAAATCAACA???GGAAAAGCTCCTCGTAAACAGCTAGCCACTAAGGCGGCCCGCAAGAGTGCTCCCGCTACGGGTGGCGTGAAGAAACCTCACCGTTACAGACCAGGCACAGTTGCACTCCGTGAAATCCGTCGTTATCAGAAGTCTACGGAGTTACTTATCCGCACGTTGCCCTTTCAACGGCTTGTGCGGGAGATC??????????????????GATCTGCGATTCCAAAGTTCCGCTGTACTAGCGCTCCAAGAGGCTAGCGAAGCTTATYTGGTGGGTCTGTTCGAAGATACCAATCTGTGCGCCATCCACGCCAAACGCGTAACCATCATGCCGAAGGATATCCAGTTGGCTCGAAGGATTCGTGGCGAGCGGGCTTAGTACAGTCGCTTGGTCATCAAACAAACACTAACGGCTCCTTTAGGAGCCACCACTAACCTAAAGGTCAATGAGCAGCCCATCTCCTTTTACACGTCTTTAATTTTTCTGTGTGTGTTCCGTTTCGTTTTGTTGTGGTTGTTGTTTCATTTTCTTGG-TTTTTGTTACGTGAGTG-AAAAACTCGCATTAT?????????????????????????????????????????????????????????????????----??????????????????????????????????????????GATCGACTCTCGGTTTGATTGGCGACAAAAATTTGACAGAAAGTGAGCAGCGACGGAGGTCAACTGTTTTGATGMRTTTTCCGCGAGTTTGCTTGAACTAGACAAACATAAAGGTC?????????????????????????????????????????????????????????????????????????????????????????????????????????????????????????????????????????????????????????????????????????????????????????????????????????????????????????????????????????????????????????????????????????????????????????????--

R17 AARAAAGAAAGAAAGGAAAGAAAGAAAG----ARAGAAAGAAAGAAAGAAAGAA????????????????AATATATGACTTTCATGTATTCTTATTAC????????????????????????????????????????????????????????????????????????????????????????????????????????????????????????????????????????????????????????????????????????????????????????????????GATGGTGGACTTCTTGTTGTAGTGCGCGAGCCGCGAGGATTCGGTCGCGATGCGTTCAAAGACATCGTTGACAAACGAGTTCATGATACCCATGGCTTTGCTGGAGATACCGGTGTCGGGGTGCACCTGTTTCGACACTT??????????GATCGCATAGCTCTCCTTTCTCTTGCTTCTCTTCTTTTTCTTGCCAGGGTCTGTCGGGGCTGATTTAACCTTGCCGGCTCTCTTCTCGCCTTTTCCGGCCGCGGGAACTTTTGGTGCCATGTCTAGAGAAATCTATCACCGTTCTCTCAAAAATAAACTGAGGTGAATGACTAGACAGGAGAATTTACTCCCTATTTAAGGTCCATTGCTGAGGATCAATTAGTATGCAAACGAAAATTCTCTTTGAAGCGATTGGCTCAGATACAGCTGTGAAAGGAACAAAAGACTGAGAGGCAACAGAGGGCGGAATT-ATTTTGATTGGAGGGTTTCGATCAGATCGGAAGAGCGT----CGT???????????????????????????????????????????????????????????????????????????????????????????????????????????????????????????????????????????????????????????????????????????????????????????????CGGCCGCATCCACCGACTCCTTCGCAAAGGAAACTATGCAGAGCGTGTCGGGGCGAGTGCTCCCGTATACTTGGCCGCTGTGCTCGAGTATCTAAGTGCTGAAATTCT???????????GAAACGCCGCTCGTGACAACAAGAAGAGCAGAATCATTCCCAGACACCTGCAGTTGGCCGTCCGAAACGACGAGGAATTGAACAAACTTCTAGCTGGTATAACGATCGCCCAGGGCGGTGTTCTTCCCAACATACAGGCCGTGCTGCTACCCAAGAAGGCCGAGAAGAAGCACCATTGACAACATCAAACT-TCAAAACGGCTCCTTTAGGAGCCACCAAACCTCGAAAAAAGTCGTGATCAGTGAAAAATCCATTTACTCCAATTGACACTCGTACATGCACACACAAGCGCTCGCGTCTTTACTTTGTTATC-GATCAAAACAAATCGTTTCAGTTCACCTAGTGTCCATCGCTTACATGCCGCATTTTTGATCCCGAACTAGTTAAACAAATAAAAAGCAAGT-CAAATCAAACAGACGGAAAGAAAGACAATTCAAATCAGCTTTGCTTTTTTTTGGAAACATTGCCGGCGACTTTGTTTT-CGTTCGTTTGGTTGGACGATGGTAATTTGGATTTGCAAAA-TGCCTCGACACATTTGCAGCCCGCTACTGTCTCTCTTTCAATTGTTGGAGCGTGGAATTTGCGACTTGCATAAACTGCCTTCCTACCTATCAAGACAGAGAAACGCATTTGAGTTTGAATGGTACGATCGCTGATC????????GCGTGGTTGTTGCAAATCCGCTTTAATCTTTTCTATTCCTTGCCATCCCCGTTCYWATMAGGAACGAGRTACTTTTCTGCTTCCCAGGTGGGGCTAATTTTGCA-AAGCAAGACAAGCGACTCGAAATGTGTG-GTGTCGGCGTTCTCATAATGATCAGAACTAACGTACTTGGAGAACGGTGCGATC???TTCTATTCCTTTGCGTGGAACAGTAATAGAATTTGAGTAGAACGTTCGCTGAACCCAGCTTTGCATGGCTTTTGAAACTCCAATTTCCTATTTGCTATTTCCTGC???????????????????????????????????????????????????????????????????????????????????????????????????????????????????????????????????????????????????????????????????????????????????????????????????????????????????????????????????????????????????????????????????-??????????????????AGTTACACTGCATTAACGCTAACAMAAGGACTAGGAGAAACGTTGAGAGCAG--AATAATATCGCCCTTTGACTCGTTGTTCAAACACAGGGTTTGACCATCAC?????????????????TTCGCATGAGGTGGTGGCTCCTAAAGGAGCCGTTTATCAATTCTCTGAGGTTTCCGTGATTCTCTACCCGCCAAAACCGTATAAGGTACGGCCTTGGCGTTTAAGGGCGTAGACCACATCCATTGCGGTAACGGTCTTCCGTTTGGCGTGTTCGGTGTAAGTCACAGCATCACGGATAACGTTTTCCAGGAAGACTTTGAGGACTCCGCGGGTCTCTTCGTAAATAAGCCCGGAAATACGCTTGACACCACCGCGGCGAGCCAGACGTCGG??????????????????????????????????????????????????????????????????????????????????????????????????????????????--?????????????????????????????????????????????????????????????????????????????????????????????????????????????????????????????????????????????????????????????????????????????????????????????????????????????????????????TCACTCACTCACTCACTCACTCACTCACTCASYCAC???????????????????????????????????????????????????????????????????????????????????????????ACTAAAATTTACTTTCCGCCTCGTCCTCGGAAAAAAAGAGTACCCGAACAAAGAA???????????????????????????????????????????GATCGTTGAGCGCTTGTTATAAAATCGGTTTTGTCACAGGAGATCGGGATAAAAGCGTAAAATTTTTGAAG--ATTACTAGACTGAAGCGTCCAATCTCGTTGGCTATGGCTCGTACCAAGCAAACCGCCCGGAAATCAACAGGTGGAAAAGCTCCTCGTAAA??????????????????GCCCGCAAGAGTGCTCCCGCTACGGGTGGCGTGAAGAAACCTCACCGTTACAGACCAGGCACAGTTGCACTCCGTGAAATCCGTCGTTATCAGAAGTCTACGGAGTTACTTATCCGCACGTTGCCCTTTCAACGGCTTGTGCGGGAGATC????????????????????????????????????????????????????????????????????????????????????????????????????????????????TCCACGCCAAACGCGTAACCATCATGCCGAAGGATATACAGTTGGCTCGAAGGATTCGTGGCGAGCGGGCTTAGT??????????????????????????????????????????????CACCACTAACCTAAAGGTCAATGAGCAGCCCATCTCCTTTTACACGTCTTTAATTTTTCTGTGTGTGTTCCGTTTCGTTTTGTTGTGGTTGTTGTTTCATTTTCTTGG-TTTTTGTTACGTGAGTG----------------------------------------------------------------------------------------------AGAAAAAAAGATATCT?????????????????GATCGACTCTCGGTTTGATTSGCGACAAAAATTTGACAGAAAGTGAGCAGCGACGGAGGTCRACTGTTTTGATGCGTTTTCCGCGAGTTTGYTTGAACTAGACAAACATAAA??????????????????????????????????------??????????????????????????????????????????????AACAAAAAAAAAACACAAGAACAAGAACAAGAACARRAACAAGGACAAACAAACAAACAAACAAACAAGAAACCAGAAAAACAAACAAGCACAGGAACAGCCTCTTT??????????????????????????????????????????????????????????????????????????????????????????????????

SD1 KAGAAAGAAAGAAA-GAAAGAAAKAAAR----AAAGAAAGAAAGAAAGAAAGATAGATC???????TACAAATATATGACTTTCATGTATTCTTATCACAAACAACG????????????????????????????????????????????????????????????????????????????????????????ATATTTGCTGACTGCTTTTGTACCCTCGCTTACTGCATGTTTTGCGAGTTCGCCCGGTAACAGTAACCTAATGGCGGTCTGG????????????????????????????????????????????????????????????????TGCGTTCAAAGACATCGTTGACAAACGAGTTCATGATACCCATGGCTTTGCTGGAGATACCGGTGTCGGGGTGCACCTGTTTCAACACTTTGTAGATGTAGATCGCATAGCTCTCCTTTCTCTTGCTTCTCTTCTTTTTCTTGCCAGGGTCTGTCGGGGCTGATTTAACCTTGCCGGCTCTCTTCTCGCCTTTTCCGGCCGCGGGAACTTTTGGTGCCATGTCTAGAGAAATCTATCACCGTTCTCTCAAAAATAAACTGAGGTGAGTGACTAGACAGGAGAATTTACTCCCTATTTAAGGTCCATTGCTGAGGATCAATTAGTATGCAAACGAAAATTCTCTTTGAAGCGATTGGCTCAGATACAGCTGTGAAAGGAACAAAAGACTGAGAGGCAACAGAGGGCGGAATT-ATTTTGATTGGAGGGTTTCGATCAGATCGGAAGAGCGTCGTGCGAGATAAGTASGGCGTCACGACTCGGAACGCGTCACAATTTAGACAACAAAATCGTTGGAATTTTAGTGTGTGTC????????????????????????????????????????????????????????????????????????????---???????????????????????????????????????????????????????????????????????????????????????CGGGGCGAGTGCTCCCGTATACTTGGCCGCTGTGCTCGAGTATCTAAGTGCTGAAATTCTAGAGTTGGCCGGAAACGCCGCTCGTGACAACAAGAAGAGCAGAATCATTCCCAGACACCTGCAGTTGGCCGTCCGAAACGACGAGGAATTGAACAAACTTCTAGCT?????TCTGATCGCCCAGGGCGGTGTTCTTCCCAACATACAGGCCGTGCTGCTACCCAAGAAGGCCGAGAAGAAGCACCATTGACAACATCAAACT-TCAAAACGGCTCCTTTAGGAGCCACCAAACCTCGAAAAAAGTCCTGATCAGTGAAAAATCCATTTACTCCAATTGACACTCGTACATGCACACACAAGCGCTCGCGTCTTTACTTTGTTATC-GATCAAAACAAATCGTTTCAGTTCACCTAGTGTCCATCGCTTACATGCCGCATTTTTGATCCCGAACTAGTTAAACAAATAAAAAGCAAGT-CAAATCAAACAGACGGAAAGAAAGACAATTCAAATCAGCTTTGCTTTTTTTTGGAAACATTGCCGGCGACTTTGTTTT-CGTTCGTTTGGTTGGACGATGGTAATTTGGATTTGCAAAA-TGCCTCGACACATTTGCAGCCCGCTACTGTCTCTCTTTCAATTGTTGGAGCGTGGAATTTGCGACTTGCATAAACTGCCTTCCTACCTATCAAGACAGAGAAACGCATTTGAGTTTGAATGGTACGATCGCTGATC????????GCGTGGTTGTTGCAAATCCGCTTTAATCTTTTCTATTCCTTGCCATCCCCGTTCCAATAAGGAACGAGGTACTTTTCTGCTTCCCAGGTGGGGCTAATTTTGCA-AAGCAAGACAAGCGACTCGAAATGTGTG-GTGTCGGCGTTCTCATAATGATC?????????????????????????????????????????????TGCGTGGAAACGTAATAGAATTTGAGTAGAACGTTCGCTGAACCCAGCTTTGCATGGCTTTTGAAACTYCAATTTMCTAYTTCCTATTTYCTRYTTCCTATTTCCTATTTCCTATTTCCTGCC????????????????????????????????????????????????????????????????????????????????????????????????????????????????????????????????????????????????????????????????????????????????????????????????????????????????????????????????????????CCACGACAAGCGACTCRRGTTACACTGCATTAACGCTAACACAAGGACTAGGAGAAACGTTGAGAGCAG--AATAATATCGCCCTTTGACTCGTTGTTCAAACACAGGGTTTGACCATCACTGTGGCTGATCAGATC????????????????????????????????????????????????????????????????????????????????????????????????????????????????????????????????????????????????????????????????????????????????????????????????????????????????????????????????????????????????????????????????????????????????????????????????????????????????????????????????????????????????????????????????????????????????????????????????????????????????????????????????????????????????????????????????????????????????????????????????????????????????????????????????????????????????????????????????????????????????????????????????????????????????????????????????????????????????????????????????????????????--?????????????????????????????????????????????????????????????????????????????????????????????????????CGGAAAAAAAGAGTACCCGAACAAAGAAATTCACCGCAGCCAATCACAAGCAAACTTTGGATAACCAATCGGATC?????????????????????????????????????GATCGGGATAAAAGCGTAAAATTTTTGAAG--ATTACTAGACTGAAGCGTCCAATCTCGTTGGCTATGGCTCGTACCAAGCAAACCGCCCGGAAATCAACAGGTGGAAAAGCTCCTCGTAAACAGCTAGCCACTAAGGCGGCCCGCAAGAGTGCTCCCGCTACGGGTGGCGTGAAGAAACCTCACCGTTACAGACCAGGCACAGTTGCACTCCGTGAAATCCGTCGTTATCAGAAGTCTACGGAGTTACTTATCCGCACGTTGCCCTTTCAACGGCTTGTGCGGGAGATCRCGMMGSWYTTCMRAWCTGATCTGCGATTCCAAAGTTCCGCTCTACTAGCGCTCCAAGAGGCTAGCGAAGCTTATTTGGTGGGTCTGTTCGAAGATACCAATCTGTGCGCCATCCACGCCAAACGCGTAACCATCATGCCGAAGGATATCSAGTTGGCTCGAAGGATTCGTGGCGAGCGGGCTTAGTACAGTCG?????????????????????????????????AGGAGCCACCACTAACCTAAAGGTCAATGAGCAGCCCATCTCCTTTTACACGTCTTTAATTTTTCTGTGTGTGTTCCGTTTCGTTTTGTTGTGGTTGTTGTTTCATTTTCTTGG-TTTTTGTTACGTGAGTG-AGAAA???????????????????????????????????????????????????????????????????????????----???????????AAAAAAAGATATCTTGTCAGAACATTCGAGCGATC??????????????????????????ATCTGACAGAAAGTGAGCAGCGACGGAGGTCAACTGTTTTGATGCGTTTTCCGCGAGTTTGCTTGAACTAGACAAACATAAAGGTCACCATTTTGCGCTTTCAGCCGTG??TATAATTTGTACCGACGTTACAATTCATTGCCGCTGTCTTTTCTTGTTCCTTA???????????????????????????????????????????????????????????????????????????????????????????????????????????????????????????????????????????????????????????????????????????????????????????????????????????------

SD2 AAGAAAGAAAGAAA-GAAGGAAAGAAAG----AAAGAAAGAAAGAAAGAA????????????????????????TATGACTTTCATGTATTCTTATCA???????????????????????????????????????????????????????????????????????????????????????????????????????????????????????????????????????????????????????????????????????????????????????????????????????????????????????????????????????????????????????????????????????????????????????????????????????????????????????????????????????????????????????GATCGCATAGCTCTCCTTTCTCTTGCTTCTCTTCTTTTTCTTGCCAGGGTCTGTCGGGGCTGATTTAACCTTGCCGGCTCTCTTCTCGCCTTTTCCGGCCGCGGGAACTTTTGGTGCCATGTCTAGAGAAATCTATCACCGTTCTCTCAAAAATAAACTGAGGTGAGTGACTAGACAGGAGAATTTACTCCCTATTTAAGGTCCATTGTTKMKGATCAATTAGTATGCCAACGAAAATTCTCTTTGAAGCGATTGGCTCAGATACAGCTGTGAAAGGAACAAAAGACTGAGAGGCAACAGAGGGCGGAATT-ATTTTGATTGGAGGGTTTCGATCAGATCGGAAGAGCGTCGTGCGAGATAAGTACGGCGTCACGACTCGGAACGCGTCACAATTTAGACAACAAAATCGTTGGAATTTTAGTG??????????????????????????????????????????????????????????????????????????????????---??????????????????????????????????????????????????????????????????????????????????????????????????????????????????????????????????????????????????????????????GAAACGCCGCTCGTGACAACAAGAAGAGCAGAATCATTCCCAGACACCTGCAGTTGGCCGTCCGAAACGACGAGGAATTGAACAAACTTCTAGCT????????GATCGCCCAGGGCGGTGTTCTTCCCAACATACAGGCCGTGCTGCTACCCAAGAAGGCCGAGAAGAAGCACCATTGACAACATCAAACT-TCAAAACGGCTCCTTTAGGAGCCACCAAACCTCGAAAAA??????GATCAGTGAAAAATCCATTTACTCCAATTGACACTCGTACATGCACACACAAGCGCTCGCGTCTTTACTTTGTTATC-GATCRAWMCAAATCGTTTCAGTTCACCTAGTGTCCATCGCTTACATGCCGC??????GATCCCGAACTAGTTAAACAAATAAAAAGCAAGT-CAAATCAAACAGACGGAAAGAAAGACAATTCAAATCAGCTTTGCTTTTTTTTGGAAACATTGCCGGCGACTTTGTTTT-CGTTCGTTTGGTTGGACG??????????????TTGCAAAA-TGCCTCGACACATTTGCAGCCCGCTACTGTCTCTCTTTCAATTGTTGGAGCGTGGAATTTGCGACTTGCATAAACTGCCTTCCTACCTATCAAGACAGAGAAACGCATTTGAGTTTGAATGGTACGATCGCTGATCACAAGTTTGCGTGGTTGTTGCAAACCCGCTTTAATCTTTTCTATTCCTTGCCATCCCCGTTCCAATAAGGAACGAGGTACTTTTCTGCTTCCCAGGTGGGGCTAATTTTGCA-TAGCAAGACAAGCGACTCGAAATGTGTG-????????????????????????????????????????????????????????????????????TGCGTGGAAACGTAATAGAATTTGAGTAGAACGTTCGCTGAACCCAGCTTTGCATGGCTTTTGAAACTCCAATTTACTACTTTCTATTTCCTGCCA-------------------TTCCAGCC?????????????????????????????????????????????????????????????????????????????????????????????????????????????????????????????????????????????????????????????????????????????????????????????????????????????????????????????????????????CACGACAAGCGACTCRRGTTACACTGCATTAACGCTAACACAAGGACTAGGAGAAACGTTGAGAGCAG--AACAATATCGCCCTTTGACTCGTTGTTCAAACACAGGGTTTGACCATCAC????????????????????????????????????????????????????????????????????????????????????????????????????????????????????????????????????????????????????????????????????????????????????????????????????????????????????????????????????????????????????????????????????????????????????????????????????????????????????????????????????????????????????????????????????????????????????????????????????????????????????????????????????????????????????????????????????????????????????????????????????????????????????????????????????????????????????????????????????????????????????????????????????????????????????????????????????????????????????????????????????????????????????????????--?????????????????????????????????????????????????????????????????????????????????????????????????????????????????????????????????????????????????????????????????????????????????????????????????????????????????????GATCGGGATAAAAGCGTAAACTTTCTGAAG--ATTACTAGACTGAAGCGTCCAATCTCGTTGGCTATGGCTCGTACCAAGCAAACCGCCCGGAAATCAACAGG?????????????????????????????????????????????????????????TACGGGTGGCGTGAAGAAACCTCACCGTTACAGACCAGGCACAGTTGCACTCCGTGAAATCCGTCGTTATCAGAAGTCTACGGAGTTACTTATCCGCACGTTGCCCTTTCAACGGCTTGTGCGGGAGATCGCGCAGGA??????????GATCTGCGATTCCAAAGTTCAGCTGTACTAGCGCTCCAAGAGGCTAG???????????????????????TCGAAGATACCAATCTGTGCGCCATCCACGCCAAACGCGTAACCA????????????????????????????????????????????????????????????????????????????????????????????????????????????????????????????????????????????????????????????????????????????????????????????????????????????????????????????????????????????????????????????????????????????????????????????????????????????????????????????????????????????????------------------------------------------------------------------------------------------------------------------------------------------------------------------?------???????????????????????????????????????????????????????????????????????????????????????????????????????????????????????????????????????????????????????????????????????????????????????????????????????????????????????????????????????????????????????????

J001 AAGAAAKAAAGAA???????????????-?????????????????????????????????????????????????????????????????????????????????????????????????????TGACATGTGGTGGCTCCTAAAGGAGCCGTTTGTTTGTGAGGTCGGGCAGTTCACTTGCTGCTTGTATATTTGGTGACTGCTTTTGTACCCTCGCTTACTGCATGTTTTGCGAGTTCGCCCGGTAACAGTAGCCTAATGGCGGTCTGGATTTCGCG???????????????????????????????????????????????????????ATGCGTTCAAAGACATCGTTGACAAACGAGTTCATGATACCCATGGCTTTGCTGGAGATACCGGTGTCGGGGTGCACCTGTTTCAACACTTTGTAGATGTAGATCGCATAGCTCTCCTTTCTCTTGCTTCTCCTCTTTTTCTTGCCAGGGTCTGTCGGGGCTGATTTAGCCTTGCCGGCTCTCTTCTCGCCTTTTCCGGCCGCGGGAACTTTTGGTGCCATGTCTAGAGAAATCTATCACCGTTCTCTCAAAAATAGACTGAGGTGAGTGACTAGACAGGAGAATTTACTCCCTATTTAAGGTCCATTGTTGAGGATC???????ATGCCAACGAAAATTCTCTTTGAAGCGATTGGCTCAGATACAGCTGTGAAAGGAAMAAAAGACTGAGAGGCAACAGAGGGCGKAATT-ATTTTGATTGGAG??????GATCCGTTWGCGCGCGCGAGCCGCGAGATAAGTACGGCGTCACGACTCGGAACGYGTCACAATTTAGACAACAAAATCGTTGG-ATTTCAGTGTGTGTCCTAGCACGAGCTAGTTTGCGAGAGAGAAAACTAAAATGACCGGTCGAGGGAAAGGAAAAGCGTCGGGCACCAAGGC---AAAGAGCAGGTCCAGCCGAGCAGGTCTTCAGTTTCCAGTCGGCCGCA??????????????????????????????????????????????????????????????????????????????????????????????????????????????????????????????????????????????????????????????????????????????????????GAGGAGTTGAACAAACTTCTAGCTGGTGTAACGATCGCCCAGGGCGGTGTTCTTCCCAACATACAGGCCGTGCTGCTACCCAAGAAGGCCGAGAAGAAGCACCATTGACAACATCAAACT-TCAAAACGGCTCC??????????????AACCTCGAAAAAAGTCGTGATCAGTGAAAAATCCATTTACTCCAATTGACACTCGTACATGCACACACAAGCGCTCGCGTCTTTACTTTGTTATC-GATCAAAACAAATCGTTTCAGTTCACCTAGTGTCCATCGCTTACATTCCGC??????GATCCCAAACTAGTTAAACAAATAAAAAGCAAGT-CAAATCAAACAGACGGAAAGAAAGACAATTCAAATCAGCTTTGCTTTTT-TTGGAAACATTGCCGGCGACTTTGTTTT-CGTTCGTTTGGTTGGACGATGGTAATTTGG??????????-????????????????????????TACTGTCTCTCTTTCAATTGTTGGAGCGTGGAATTTGCGGCTTGCATAAACTGCCTTCCTACCTATCAAGACAGAGAAACGCATTTGAGTTTGAATGGTACCATCGCTGATAACAAGTTTGCGTGGTTGTTGCAAATCCGCTTTAATCTTTTCTATTCCTTGCCATCCCCGTTCCAATAAGGAACGAGGTACTTTTCTGCTTCCCAGGTGGGGCTAATTTTGCA-AAGCAAGACAAGCGACTCGAAATGTGTG-ATGTCGGCGTTCTCATAATGATC???????????????????AACGGTGCKATCACGCGTGGTTCCATTGCGTGGAAACGTAATAGAATTTGAGTAGAACGTTCGCTGAACCCAGCTTTGCATGGCTTTTGAAACTCCAATTTCCTATTTCCCATTTCCCATTTCCCATTTCCTATTTCCTATTTCCTATTTCCT???????????????????????????????????????????????????????????????????????????????????????????????????????????????????????????????????????????????????????????????????????????????????????????????TTTCCTATTTCCTATTTCCTGCCATCCCAGTTCCC-???????????????????????????????????????????????????????AAACGTTGAGAGCAG--AATAATATCGCCCTTTGACTCGTTGTTCAAACACAGGGTTTGACCATC?????????????????????????????????????????AAAGGAGCCGTTTATCAGTTCTCTGAGGTTTCCGTGATTCTCTACCCGCCAAA?????????????????????????????????????????????????????????GGTCTTCCGTTTGGCGTGTTCGGTGTAAGTCACAGCATCACGGATAACGTTTTCCAGGAAGACTTTGAGGACTCCGCGGGTCTCTTCGTAAATAAGCCCGGAAATACGCTTGACACCACCGCGGCGAGCCAGACGTCGGATTGCTGGCTTG?????????????????????????????????????????????????????????????GCCCTTTACCTCCTTTGCCGCGTCCAGACATGATTTG--TTTTTCTTTCGAATGTTACGGATGCAATTGTTGTCGGCCCTGAACAAAACTTGAG?????????????????????????????????????????????????????????????????????????????????????????????????????????TAAAGGCTAGCGAAAATGACTCACAACCTTCAAAGTAATCCATGGTCAAATTCAGTCTGACTCACTCACTC?CAGTCTSACTCACTCACTCACTAATTAGGTTATTACTTTTTCGAGAGTACTCACTGAAGGCGAGCGACTGAAAACCGAGGGCAGTTTCGAGAAAAATTGCAGGTCAGAGTTTACTAAAATTTACTTTCCGCCTCGTCCTCGGAAAAAAAGAGTACCCGAACAAAGAAATTCACCGCAGCCAATCACAAGCAAACTTTGGATAACCAATCGGATC?????????????????????????????????????GATCTCGAGAAAAGCGTAACATTTCTGAAG--ATTACTAGACTGAAGCGTCCAATCTTGTTGGCTATGGCTCGTACCAAGCAAACCGCCCGGAAATCAACAGGTGGAAAAGCTCCTCGTAAACAGTTAGCCACTAAGGCGGCCCGCAAGAGTGCTCCCGCTACGGGTGGCGTGAAGAAACCTCACCGTTACAGACCAGGCACAGTTGCACTCCGTGAAATCCGTCGTTATCAGAAGTCTACGGAGTTACTTATCCGCAAGTTGCCCTTTCAACGGCTTGTGCGGGAGATCGCGCAG????????????GCTCTGCGATTCCAAAGTTCCGCTGTACTAGCGCTCCAAGAGGCTA???????????????????????????????????????????????????????????????????????????????????ATCCAGTTGGCTCGAAGGATTCGTGGCGAGCGGGCTTAGTACAGTCGCTTGGTCATCAAACAAACACTAACGGCTCCTTTAGGAGCCACCACTAACCTAAAGGTCAATGAGCAGCCCATCTCCTTTTACACGTCTATAATTTTTCTGTGTGTGTTCCGTTTCGTTTTGTTGTGGTTGTTGTTTCATTTTCTTGGTTTTTTGTTACGTGAGTGAAAAAACTCGCATTATTGTCACGAAGCAACAGAGAAAATCGATGACCACATCTCCAAAGGTGACCTGCCTCTGCGGAAAAA----TGTCGACGTAGAAGAAAAGATATCTTGTCAGAACATTCGAGCGATCGACTCTCGGTTTGATTCGCGACAAAAATTTGACAGAAAGTGAGCAGCGACGGAGGTCGACTGTTTTGATGCGTTTTCCGCGAGTTTGTTTGAACTAGACAAACATAAAGGTCACCATTATGCGCTTTCAGCCGTTCGTATAATTTGTACCGACGTTACAATTCATTGCTGCTGTCTTTTCTTGTTCCTTACTTGGCAAAAAAAAAAAA??????????GATCAAGGACA--------AACAAACAAACAAACAAACAAACAAGAAACCAGAAAAACAAACAAGCACAGGAACAGCCTCTTTTTAAGCAGGCGCAATTACCTC?????????????????????????????????????????????????????????????????????????????

J295 AAGAAAGAAAGAAA-GAAAGAAAGAAAG----AAAGAAAGAAAGAAAGAAAGAAAG?????????????????????????????????????????????????????????????????????????????????????????????????????????????????????????????????????????????????????????????????????????????????????????????????????????????????????????????????????????????????????????????????????????????????????????????????????????????????????????????????????????????????????????????????????????????????????????????????GATCGCATAGCTCTCCTTTCTCTTGCTTCTCTTCTTTTTCTTGCCAGGGTCCGTCGGGGCTGATTTAGCCTTGCCGGCTCTCTTCTCGCCTTTTCCGGCCGCGGGAACTTTTGGTGCCATGTCTAGAAAAATCTATCACCGTTCTCTCAAAAATAGACTGAGGTGTGTGACTAGACAGGAGAATTTACTCCCTATTTAAGGTCCATTGTTGAGGATC??????TATGCCAACGAAAATTCTCTTTGAAGCCATTGGCTCAGATACAGCTGTGAAAGGAACAAAAGACTACGAGGCAACAGAGGGCGGAATTAATTTTGATTGGAGGGTTTCGATCAGATCGGAAGAG???????????????????????????----????????????????????????????????????????????????????????????????????????????????????????????????????????????????????????????????????????????????????????????????????????????????????????????????????????????????????????????????????????????????????????????????????????????????????????????????????????????????????????????????????????????????????????????????????????????????????????????????????????TATCGCCCAGGGCGGTGTTCTTCCCAACATACAGGCCGTGCTGCTACCCAAGAAGGCCGAGAAGAAGCACCATTGACAACATCAAACCATAAAAACGGCTCCTTTAGGAGCC???????????????????????????????????????????????????????????????????????????????????????????????????-?????????????????????????????????????????????????????????GATCCCAAACTAGTTAAAGAAATAAAAAGCAAATAAAAATCAAACAGACGGAATGAAAGACAATTCAAATCAGCTTTGGTTTTT-TTGGAAACATTGCCGGCGACTTTGTTTTCCTTTCGTTTGGTTGGACGATGGTAATTTGGATT???????-?????????????????CGCTCTTGTCTCTCTCTCTTTCAATTGTTGGAGTGTGGAATTTGCGAATTGCATAAACTGCTTTCCTACCTATCAAGACAGAGAAACGCATTTGAGTTTGAATGGTACGATCGCTGATC?????TTTGCGTGGTTGTTGCAAACCCGCTTTAATCTTTTCTATTCCTTGCCATCCCCGTTCCAATAAGGAACGAGGTACTTTTATTGGG?????????GGCTAATTTTGCATAAGCAAGACAAGCGACTCGAAATGTGT?-??????????????????????????????????????????????????TATCACGCGTGGTTCCATTGCGTGGAAACGTAATAGAATTTGAGTAGAACGTTCGCTGAACCCAGCTTTGCACGGCTTTTGAAACCCCAATTTACTACTTCCTATTTCCTGCC??????????????????????????????????????????????????????????????????????????????????????????????????????????????????????????????????TGCGTGGAAACGTAATAGAATTTGAGTA??????????????????????????????????????????????????????????????????????????????????????????????????????????????AGCGACY-GAGTTACACTGCAKTAACGCTAACACAAGGATTAGGAGAAACGTTGAGAGCAGAAAAAAATATCGCCCTTTGACTCGTTGTTCAAACACAGGGTTTGACCATCAC????????????????????????????????????????????????????????????????????????????????????????????????????????????????????????????????????????????????????????????????????????????????????????????????????????????????????????????????????????????????????????????????????????????????????????????????????????????????????????????????????????????????????????????????????????????????????????????????????????????????????????????????????????????????????????????????????????????????????????????????????????????????????????????????????????????????????????????????????????????????????????????????????????????????????????????????????????????????????????????????????????????????????????????--?????????????????????????????????????????????????????????????????????????????????????????????????????????????????????????????????????????????????????????????????????????????????????????????????????????????????????KATCGCGAGAAAAGCGTAAAATTTCKGAAGAAATTACGAGACTGAAGCSTCCAATCTTGTTAGCTATGGCTCGTACCAAGCAAACCGCCCGGAAATCAACAG????????????????????????????????????????????AAGAGTGCTCTCGCTACGGG-GGCGTGAAGAAACCTCACCGTTACAGACCAGGCACAGTTGCACTCCGTGAAATCCGTCGTTATCAGAAGTCTACGGAGTTACTTATCCGCAAGTTGCCCTTTCAACGGCTTGTGCGGGAGATC?????????????????????????????????????????????????????????????????????????????????????????????????????????????????????????????????????????????????????????????????????????????????????????????????????????????????????????????????????????????????????????????????????????????????????????????????????????????????????????????????????????????????????????????????????????????????????????????????????????????????????????????????????????????????????????????????????????????????????????????????????????????????????????????????????????????????????????????????????????????????????????????????????????????????????????????????????????????????????????????????????????????????????????????????????????????????????????????????????????????????????????????????????????????????????????????????????????????????????????????????????????????????????????????????????????????????????????????????????????----------------

S3 AAGAAAGAAAGAAANGAAAGAAAGAAAGAAAGAAAGAAAGAAAGAAAGAAAGAAAGAAAGATA?????????????????????????????????????????????????GTTGGACATTTACTTTCATGACATGTGGTGGCTCCTAAAGGAGCCGTTTGTTTGTGAGGTCGGGCAGTTCACTTGCTGCTTGTATATTTGGTGACTGCTTTTGTACCCTCGCTTACTGCATGTTTTGCGAGTTCGCCCGGTAACAGTAACCTAATGGCGGTCTGGATTTCGCGAGAGCTGATGGTGGATTTCTTGTTGTAGTGCGCGAGCCGCGAGGATTCGGTCGCGATGCGTTCAAAGACATCGTTGACAAACGAGTTCATGATACCCATGGCTTTGCTGGAGATACCGGTGTCGGGGTGCACCTGTTTCAAAACTTTGTAGATGTAGATCGCATAGCTCTCCTTTCTCTTGCTTCTCTTCTTTTTCTTGCCAGGGTCCGTCGGGGCTGATTTAGCCTTGCCGGCTCTCTTCTCGCCTTTTCCGGCCGCGGGAACTTTTGGTGCCATGTCTAGAAAAATCTATCACCGTCCTCTCAAAAATAGACTGAGGTGTGTGACTAGACAGGAGAATTTACTCTCTATTTAAGGTCCATTGTTGAGGATCAATTAGTATGCCGACGAAAATTCTCTTTGAAGCGATTGGCTCAGATACAGCTGTGAAAGGAACAAAAGACTAAGAGGCAACAGAGGGCGGAATT-ATTTTGATTGGAGGGTTTCGATCCGTTAGCGCGCGCGAGCCGCGAGAYAAGTACGGCGTCACGACTCGGAACGCGTCACAATTTGGACAACAAAATCGTTGG-ATTTCAGTGTGTGTCCTGGCACGAGCTAGTTTGCGAGAGAGAAAACTAAAATGACCGGCCGAGGGAAAGGAAAAGCGTCGGGCACCAAGGC---AAAGAGCAGGTCCAGCCGAGCAGGTCTTCAGTTTCCAGTCGGCCGCATCCACAGACTCCTTCGCAAAGGAAACTATGCAGAGCGTGTCGGGGCGGGTGCTCCCGTGTACTTGGCCGCTGTGCTCGAGTATCTAAGTGCTGAAATTCTAGAGTTGGCCGGAAACGCCGCTCGTGACAACAAGAAGACCAGAATCATTCCCAGACACCTGCAGTTGGCCGTCCGAAACGACGAGGAATTGAACAAACTTCTAGCTGGTGTAACGATCGCCCAGGGCGGTGTTCTTCCCAACATACAGGCCGTGCTGCTACCCAAGAAGGCCGAGARGAAGCACCATTGACAACATCAAACCATAAAAACGGCTCCTTTAGGAGCCACCAAACCTCGAAAAAAGTCGTGATCAGTGAAACATCCATTTACTCCAATTGACACTCGTACATGCACACACAAGCGCTCGCGTCTTTACTTTGTTGTC-GATCAAAACAAATCGTTTCAGTTCACCTAGTGTCCATCGCKTACATTCCGCATTTTTGATCCCAAACTAGTTAAAGAAATAAAAAGCAAATAAAAATCAAACAGACGGAATGAAAGACAATTCAAATCAGCTTYGGTTTTT-TTGGAAACATTGCCGGCGACTTTGTTTT-CGTTCGTTTGGTTGGACGATGGTAATTTGGATTTGCAAAA-TGCCTCGACACATTTGCAGSYCKCTNYTCTCTCTCTTTCAATTGTTGGAGTGTGGAATTTGCGAATTGCATAAACTGCTTTCCTACCTATCAAGACAGAGAAACGCATTTGAGTTTGAATGGTACGATSGCTGATCACAAGTTTGCGTGGTTGTTGCAAACCCGCTTTAATCTTTTCTATTCCTTGCCATCCCCGTTCCCATAAGGAACGAGGTACTTTTATTGGG?????TTGGGGCTAATTTTGCA-TAGCAAGACAAGCGACTCGAAATGTGTGTGTTRWTGAAATGTGTTAATGATCAGAACTAACGTACTTGGAGAACGGTGCGATCACGCGTGGTTCCATTGCGTGGAAACGTAATAGAATTTGAGTAGAACGTTCGCTGAACCCAGCTTTGCACGGCTTTTGAAACTCCAATTTACTACTTCCTATTTCCTGCCATCCCWKTACTACTTCCTATTTCCTGCCATCCCAGTTCCCTGTTGGAACGAGAGACTATTTKRCACTGCATTAACGCTAACACAAGGATTAGGAGAAACGTTGA????????GATCACGCGTGGTTCCATTGCGTGGAAACGTAATAGAATTTGAGTAGAACGTTCGCTGAACCCAGCTTTGCGTGGCTTTTGAAACTCCAATTTCCTATTTCCTATTTCCTATTTCCTAYTTCCTATTTCCTGCCATCCCAGTTCCCTGTTRSRACRAGCGACTSRRNTTACACTGCATTAACGCTAACACAAGGATTAGGAGAAACGTTGAGAGCAG--AAAAATATCGCCCTTTGACTCGTTGTTCAAACACAGGGTTTGACCATCACTGTGGCTGATCACGACTTTCGCATGAGGTGGTGGCTCCTAAAGGAGCCGTTTATCAGTTCTCTGAGGTTTCCGTGATTCTCTACCCGCCAAAACCGTATAGGGTACGGCCTTGGCGTTTAAGGGCGTAGACCACATCCATTGCGGTAACGGTCTTCCGTTTGGCGTGTTCGGTGTAAGTCACAGCATCACGGATAACGTTTTCCAGGAAGACTTTGAGGACTCCGCGGGTCTCTTCGTAAATGAGCCCGGAAATTCGCTTGACACCACCGCGGCGAGCCAGACGTCGGATTGCTGGCTTGGTGATTCCCTGGATGTTGTCTCGCAGAATCTTTCGATGGCGCTTGGCGCCTCCTYYTCCWWRYCCTTTACCTCCTTTGCCGCGTCCAGACATGATTTGTTTTTTTCTTTCGAATGTTACGGATGCAACTGTTGTCGGCCCTGAACAAAACTTGAGATGTGTGGCTGGACCTCTCGGGAAACGTCACATTTCCAATTGACAAGAAAGCCAGGCCAAATGCCGACTTATGAATACCATTATCACTTATCTACACGGCCACGCTAAAGGATAGCGAAAATGACTCACAACCTTCAAAGTAATCCATGGTCAAATTCAGTCTCACTCACTCACTCACTCACTCACTCACTCACTCACTMATTAGTTTATTACTTTTTCGAGAGTACTCACTGAAAGCGAGCGACTGAAAACCGAGGGCWGTTTTGAGAAAAGTTGCAGGTCAGAGTTTACTAAAATTTACTTTCCGCCTCATCCTCGGAAAAAAAAAAARMSSGAACAAAGAAACTCACCGCAGCCAATCACAAGCAAACTTTGGATAACCAATCGGATCGTTGAGCGCTTGTCATAAAATCGGTTTTGTCACAGGAGATCGCGAGAAAAGCGTAAAATTTCTGAAG--ATTACGAGACTGAAGCGTCCAATCTTGTTAGCTATGGCTCGTACCAAGCAAACCGCCCGGAAATCAACAGGTGGAAAAGCTCCTCGTAAACAGTTAGCCACTAAGGCGGCCCGCAAGAGTGCTCCCGCTACGGGTGGCGTGAAGAAACCTCACCGTTACAGACCAGGCACAGTTGCACTCCGTGAAATCCGTCGTTATCAGAAGTCTACGGAGTTACTTATCCGCAAGTTGCCCTTTCAACGGCTTGTGCGGGAGATCGCGCAGGATTTCAAAACCGATCTGCGATTCCAAAGTTCCGCTGTACTAGCGCTCCAAGAGGCTAGCGAAGCTTACTTGGTGGGTCTGTTCGAAGATACCAATCTGTGCGCCATCCACGCCAAACGCGTAACCATCATGCCGAAGGATATCCAGTTGGCTCGAAGGATTCGTGGCGAGCGGGCTTAGTACAGTCGCTTGGTCATCAAACAAACACTAACGGCTCCTTTAGGAGCCACCACTAACCTAAAGGTCAATGAGCAGCCCATCTCCTTTTACACGTCTTTAATTTTTCTGTGTGTGTTCTGTTTCGTTTTGTTGTGGTTGTTGTTTCATTTTCTTGG-TTTTTGTTACGTGAGTG-AAAAACTCGCATTATTGTCATGAAGCAACAGAGAAAATCAATGACCACACCTCCAAAGGTGACCTGCCTCTGCAGAGAAAAGNNTGTCGACGTAGAAAAAAAGSCGAGCGATC?????????GATCGATCGACTCTCGGTTTGATTCGCGACAAAAATTTGACAGAAAGTGAGCAGCGACGGAGGTCAACTGTTTTGATGCGTTTTCCGTGAGTTTGCTTGAACTAGACAAACATAAAGGTCACCATTTTGCGCTTTCAGCCGTTCGTATAATTTGTACCGACGTTTCAATTCATTGCCGCTGTCTTTTCTTGTTCCTTACTTGGCAAAAAAAAAAAGACAAAAACAAGAACAAGAACAAGAGCCTCAACAAACAAACAAACAAACAAACAA?????????????????????????????????????????????????????????????????????????????????????????????????????????????????????????????????????????

SD4 AAGAAAGAAAGATCGGA???????????-?????????????????????????????????????????????????????????????????????????????????????????????????????????????????????????????????????????????????GGCAGTTCAATTGCTGCTTGTATATTTGCTGACTGCTTTTGTACCCTCGCTTACTGCATGTTTTGCGAGTTCGCCCGGTAACAGT??????????????????????????????????????????????????????????????????????????????????????????????????????????????????????????CATGGCTTTGCTGGAGATACCGGTGTCGGGGTGCACCTGTTTCAACACTTTGTAGATGTAGATCGCATAGCTCTCCTTTCTCTTGCTTCTCTTCTTTTTCTTGCCAGGGTCTGTCGGGGCTGATTTAACCTTGCCGGCTCTCTTCTCGCCTTTTCCGGCCGCGGGAACTTTTGGTGCCATGTCTAGAGAAATCTATCACCGTTCTCTCAAAAATAAACTGAGGTGAGTGACTAGACAGGAGAATTTACTCCCTATTTAAGGTCCATTGSTGAGGATCAATTAGTATGCAAACGAAAATTCTCTTTGAAGCGATTGGCTCAGATACAGCTGTGAAAGGAACAAAAGACTGAGAGGCAACAGAGGGCGGAATT-ATTTTGATTGGAGGGTTTCGATCAGATCGGAAGAGCGTCGTGGGAAAG----???????????????????????????????????????????????????????????????????????????????????????????????????????????????????????????????????????????????????????????????????????????????????????????????????????????????????????????????????????????????????????????????????????????????????????????????????TAGAGTTGGCCGGAAACGCCGCTCGTGACAACAAGAAGAGCAGAATCATTCCCAGACACCTGCAGTTGGCCGTCCGAAACGACGAGGAATTGAACAAACTTCTAGCTGGTGTAACGATCGCCCAGGGCGGTGTTCTTCCCAACATACAGGCCGTGCTGCTACCCAAGAAGGCCGAGAAGAAGCACCATTGACAACATCAAACT-TCAAAACGGCTCCTTTAGGAGCCACCAAACCTCGAAAAAAGTCGTGATCAGTGAAAAATCCATTTACTCCAATTGACACTCGTACATGCACACACAAGCGCTCGCGTCTTTACTTTGTTATC-GATCAAAACAAATCGTTTCAGTTCACCTAGTGTCCATCGCTTACATGCCGCATTTTTGATCCCGAACTAGTTAAACAAATAAAAAGCAAGT-CAAATCAAACAGACGGAAAGAAAGACAATTCAAATCAGCTTTGCTTTTTTTTGGAAACATTGCCGGCGACTTTGTTTT-CGTTCGTTTGGTTGGACGATGGTAATTTGGATTTGCAAAA-TGCCTCGACACATTTGCAGCCCGCTACTGTCTCTCTTTCAATTGTTGGAGCGTGGAATTTGCGACTTGCATAAACTGCCTTCCTACCTATCAAGACAGAGAAACGCATTTGAGTTTGAATGGTACGATCGCTGATCACAAGTTTGCGTGGTTGTTGCAAATCCGCTTTAATCTTTTCTATTCCTTGCCATCCCCGTTCCAATARGGAACGAGGTACTTTTCTGCTTCCYAGGTGGGGCTAATTTTGCA-AAGCAAGACAAGCGACTCGAAATGTGTG-GTGTCGGCGTTCTCATAATGATC???????????????????????????GATCACGCGTGGTTCCATTGCGTGGAAACGTAATAGAATTTGAGTAGAACGTTCGCTGAACCCAGCTTTGCATGGCTTTTGAAACTCCAATTTCCTATTTCCTATTTCCTATTTCCT????????????????????????????????????????????????????????????????????????????????????????????????????????????GATCACGCGTGGTTCCATTGCGTGGAAACGTAATAGAATTTGAGTAGAACGTTCGCTGAACCCAGCTTTGCATGGCTTTTGAAACTCCAATTTCCTATTTCCTATTTCCTATTTCCTATTTCCTATTTCCTGCCATCCCARTTCCCTGYCACGACAAGCGACTCGRGTTACACTGCATTAACGCTAACAAAAGGACTAGGAGAAACGTTGAGAGCAG--AATAATATCGCCCTTTGACTCGTTGTTCAAACACAGGGTTTGACCATCACTGTGGCTGATCACGACTTTCGCATGAGGTGGTGGCTCCTAAAGGAGCCGTTTATCAATTCTCTGAGGTTTCCGTGATTCTCTACCCGCCAAAACCGTATAGGGTACGGCCTTGGCGTTTAAGGGCGTAGACCACATCCATTGCGGTAAC????????????????????????????????????????????????????????????????????????????????????????????????????????????CTTGACACCACCGCGGCGAGCCAGACGTCGGATTGCTGGCTTGGTGATTCCCTGGATGTTGTCTCGCAGAATCTTTCGATGGCGCTTGGCGCCCCCTTTTCCAAGCCCTTTACCTCCTTTGCCGCGTCCAGACATGATTTG--TTTTTCTTTCGAATGTTACGGATGCAATTGTTGTCGGCCCT?????????????????????????????????????????????????????????????????????????????????????????????????????????????????????????????????????????????????????????????????????????????????????????????????????????????????????AATTAGTTTATTACTTTTTCGAGAGTACTCACTGAAGGCGAGCGACTGAAAACCGAGGGCAGTTTCGAGAAAAATTGCAGGTCAGAGTTTACTAAAATTTACTTTCCGCC???????????????????????????????????????????????????????????????????????????????????????????????????????????????????????GATCGGGATAAAAGCGTAAAATTTTTGAAG--ATTACTAGACTGAAGCGTCCAATCTCGTTGGCTATGGCTCGTACCAAGCAAACCGCCCGGAAATCAACAGGTGGAAAAGCTCCTCGTAAACAGCTAGCC?????????????????GAGTGCTCCCGCTACGGGTGGCGTGAAGAAACCTCACCGTTACAGACCAGGCACAGTTGCACTCCGTGAAATCCGTCGTTATCAGAAGTCTACGGAGTTACTTATCCGCACGTTGCCCTTTCAACGGCTTGTGCGGGAGATCGCGCAGGATTTCAAAACTGATCTGCGATTCCAAAGTTCCGCTCTACTAGCGCTCCAAGAGGCTAGCGAAGCTTATTT???????????????????????????????????????????????????????????????????????????????????????????????????????????AGTACAGTCGCTTGGTCATCAAACAAACACTAACGGCTCCTTTAGGAGCCACCACTAACCTAAAGGTCAATGAGCAGCCCATCTCCTTTTACACGTCTTTAATTTTTCTGTGTGTGTTCCGTTTCGTTTTGTTGTGGTTGTTGTTTCATTTTCTTGG-TTTTTGTTACGTGAGTG----------------------------------------------------------------------------------------------AGAAAAAAAGAT??????????????????????????????????????????????????????????????????????????????????????????????????????????????????????????????????????????????????????????????????????????????????????????????????????????????????????????????????????????????????????????????????????????????????????????????????????????????????????????????????????????????????????????????????????????????????????????????????????????????????????????????----------

SD6 AAGAAAGAAAGAAA-RAAAGAAAGAAAGAAAGAAAGAAAGAAAGAAAGAAAGAAAGAAAG???????????????????????????????????????????????????????????????????????????????????????????????????????????????????????????????????????????????????????????????????????????????????????????????????????????????????????????????????????????????????????????????????????????????????????????????????????????????????????????????????????????????????????????????????????????????????????????????????????????????????????????????????????????????????????????????????????????????????????????????????????????????????????????????????????????????????????????????????????????????????????????????????????????????????????????????????????????????????????????????????????????????????????????????????????????????????????????????????????????????????????????????????????????????????----------????????????????????????????????????????????????????????????????????????????????????????????????????????????????????????????????????????????????????????????????????????????????????????????????????????????????????????????????????????????????????????????????????????????????????????????????????????TGACAACACGAAGGCCAGAGTCATTCCCAGACACCTGCAGTTGGCCGTGCGAAACGACGAAGAACTGAACAAACTTTTAGCTGGTGTAACGATC????????????????????????????????????????????????????????????????????????????????????????????????????????????????????????????????????????????????????????????????????????????????????????????????????????????????????????????????????????????????????????????????????????????????????????????????????????????????????????????????????????????????????????????????????????????????????????????????????????????????????????????????????????????????????????????????????????????????????????????????????????????????????????????????????????????????????????????????????????????????????????????????????????????????????????????????????????????????????????????????????????????????????????????-?????????????????????????????????????????????????????????????????????????????????????????????????????????????????????????????????????????????????????????????????????????????????????????????????????????????????????????????????????????????????????????????????????????????????????????????????????????????????????????????????????????????????????????????????????????????????????????????????????????????????????????????????????????????????????????????????????????????????????????????????????????????????????????????????????????--??????????????????????????????????????????????????????????????????????????????????????????????????????????????????????????????????????????????????????????????????????????????????????????????????????????????????????????????????????????????????????????????????????????????????????????????????????????????????????????????????????????????????--------------------------???????????????????????????????????????????????????????????????????????????????????????????????????????????????????????????????????????????????????????????????????????????????????????????????????????????????????????????????????????????????????????????????????????????????????????????????????????????????????????????????????????????????????????????????????????????????????????????????????????????????????????????????????????????????????????????????????????????????????????????????????????????????????????????????????????????????????????????????????????????????????????????????????????????????????????????????????????????????????????????????????????????????????????????????????????????????????????????????????????????????????????????????????????????????????????????????????????????????????????????????????????????????????????????????????????????????????????????????????????????????????????????????????????????????????????????????????????????????????????????????????????????????????????????????????????????????????????????????????????????????????????????????????????????????????????????????????????????????????????????????????????????????????????????????????????????????????????????????????????????????????????????????????????????????????????????????????????????????????????????????????????????????????????????????????????????????????????????????????????????????????????????????????????????????????????????????????????????????????????????????????????????????????????????????????????????????????????????????????????????????????????????????????????????????????????????????????????????????ACAAACAAACAAACAAACAAACAAACAAAACA??????????????????????????????????????????????????????????????????????????????????????????????????????????????????????????????????---

***Pocillopora* Mitochondrial Phylip Alignment**

Entire mitochondrial genomes of *S. pistillata* (accession number: EU400214; pistil), *S. hystrix* (accession number: NC_010244; hystri), *Seriatopora caliendrum* (Ehrenberg, 1834) (accession number: NC_010245; calien), *Pocillopora eydouxi* (accession number: NC_009798; Peydou), *P. damicornis* (accession number: NC_009797; Pdami), and *P. damicornis* (accession number: EU400213; Pdam), along with the mitochondrial consensus sequences generated per library.

19 17884

pistil TGTAAGAAAGACAAAGGGTTAGTCATTGGGTTCATGCCCCAAATAAGTGAGTTCGAATCTCTCTCTTACACAAAATAAAAATTATA-AAAAAAAAAGAGGATAACTAAGATAAACTAAAATTAGACGATTTTTTTCGAAATGGCGTTCAGTTGCTTATTGGTTGTTTCTCCAGAAAAGTGATTT---TTTTAATTTGAATTGAAACATCTTATTACTAAGTAAAAATCAAACGAGATTCCGAGAGTGGCGGAGAGTGAAATTGGAGTTGTGCTTGTGGTGGTCATAGATCTTGCTTAAACATTAGTTTATACTGATAATGAGAGTACTGTAAAGGAAAGTTGAAAGAGAGTTGAAAGGAATTTGAATTTTTTATTTTTGAAGTAGCTTTAAAAAAGCGTACCTTTTGTATAATGGGTAAAAGAGATTTATTTGGCATATTTAAAATGGAAAATTTTAAGTAATTTTTTTTTTAGTCAAATTTCCCGAAACCAAGTGATTTAATCATGAATTGTGTGAGCAAAAACTGGTGATTGTGGCAAAAATCTCAGAAAATTTGTGATTAGGGGTGAAAGGCTAATCGAACTTGGAAATAGCTGGTTTTCTGCGAAAACTATTTAAGTAGTGTTC-TTTTTTTTTTTATTTTAATTGTCATTTTAAAAAAAACAAAAATAAAAAAAAAGTAAACAAACAATAAATGAAAAGATTTTTTGTTAAAAGAGAAAGCTCAAATCAGAAGCTATTGTCATT-TTTTTTTTTTTAGTTTAAAAAAAAATTAAATTTAGACAATAAAAATGTAGGCTTGGAACCAGCCATCTTTTAAAAAGTACGTAGTTGTTTATTTAAAAATTTTAAATTTTTTTATAATTTTGATGGTTAAAATAAAACAGAAGCTCTGAAAATAATTATATATTTTGTAGCAGAATAAATTGTTTTTGATATTTGAGTTGATTTCAAAATCTTTTTTAGTTTGAAAAATTTTTT-TTAATACATCGTATTCAGTTGTAATAATTTTTACATAAGTAATCTAAATATTATTTTTTTTTTCTTAAGATTACCTTAGTCAAGGATAATACAGTTTCTAAAAAAGAAATTTTTTATGAAGATTTTTTATAAAAATATATAATATCGGGAAAAGAAAAAAAAAAGAACTGTTTTTCTAATTAAAGAAAGAAAAAAAAAAAGAAAAACATTTTTTTAAGGAACTCGGCAAAGTTAAACTTCGACTGTTTACCAAAAACATAGCTTTTTGATCTTTATAAAAGGTGAAACCTGCCCGATGGTTGTATCTTAATATGTTTGTCTCGCTTACTAATAAAGACAATTAAATGGCCGCGGTAACACTGACTGTGATAATGTAGCGTAATCAATTGTCAATTAATTGTTGACCGGTATGAATGGTACCCCGAAAGTTTTTCTGTCTTAAAAAAATATTCAATGAAATTAAATTTGTAGTGAAGATGCTACATTTTAATTGTTAGACGAGAAGTCCCCATGGAGCTTTACTGTTAGCTTATATATATAATTTTTTTTTTTTTATAAGCAAGACAGTTTTGTTGGGGCGACAGTTTTTTAAAAAGTAACGAAAATGAACTATGACGCATGTTTAACTTTGAAATTTTTTTAATTGATGAGACATTTTTGGTGTGTTTTTTGATCCGTTATTTTGAATGAAAAAAATAACGAAAACAAATAAAAGTTACCCTGGGGATAACAGCGCAATAACGTTTGAGAGTTAATTAACGACAGTGTTTGCGACCTCGATGTTGAATTGTAACGTCCTACGGTGTAGTCACTCGTAAGGGTTGGTTTGTTCATCCATTAAAGTTATACATGATTTGAGTTAAAAGCGTGGTGACACAGCTTGGTTTCTATCTACAATTAGAAAACAAAAATATGTTGTTTTTTCGTACGAAAGGATCAAAAATCAATAGTTCCCTTAATATAACTATTCTTTAAATTAGGATTGCTTCTAAAAATAAAAAGAAATATTTTTGTTTGTTGTTTTTATTAATGTATCTTTTAGTTTTATTTTTTCCTTTAATTGGAGCTGTTTTAACAGGTTGTTTTGGAAGAAAAATTGGAGAAAGAGGAGCGGGGATTTTAACTTCAAGTTGTTTAGTTTTTAGTTTGTCTTATTCTTTTTTAATCGCGATTGAAGTTTTATTTAATTCAACAACAACGTATTTGAAATTATGAAAATGATTTGATTCGGGATTGTTTGTTGTTTTTTTTGGTTTTCAATTTGATGGTTTAGTAGTTATTATGTTATTTGTTGTTTTTATTGTTTCTACTTTGGTTCATATTTTTTCTATTGCTTATATGCGGGGGGATCCTCATGTTCCTCGATTTATGACATATCTTTCTTTGTTTACTTTTTTAATGGTTTTATTGGTAACTAGTGATAATTTTCTTCAATTATTTATTGGGTGAGAAGGGGTTGGCCTTTGTTCTTATTTATTAATTAATTTTTGATTAACTCGATTAGAGGCAAATAGAGCTGCTATTAAAGCAATGTTAGTGAATAGAATTGGTGATATTGGGTTGCTTTTAGCAATGTTTTTACTTTGGGATCTTTTTGGATCTCTAGATTTTTCTACAATTTTTAATTCTATTTTTTTTTCTAATCAAATATTTTTTATTTGTTTATTTTTATTTTTTGGGGTTATGGGCAAATCTGCTCAATTAGGATTACATACTTGGTTACCGGATGCAATGGAAGGTTATTGGGCCTTTTAATTAAAAAATTAATTAAAAAAATTACTATACATTAAAAAAACAATTTATTAGTAAAATAATTGTTTATTAATGGACAATAAAATATTTATTTTAATGTCTAAGAGACTTTATGTGATTTACTTTTTTTTATTTGATTAAATCTATTATTATTATTATTCCTTTACTTATTGCTGTTGCATTTTTAACTTTAGCAGAACGAAAAATTTTAGGGTATATGCAAATAAGAAAAGGACCAAATGTTGTTGGAGGGGGGCTTCTTCAACCTTTTGCAGATGGGGTGAAATTATTTATTAAAGAAATGATTCTTCCCCATCAAGCAAATAAATTTGTTTATCTTTTGGCTCCAGTTATATCTTTTACATTAGCTTTCTTTGTTTGAGGTTTTCTTCCTTATGAGAAAGGAGTGAGTATTAGTGATTTTAAAATTAGTCTTTTATGAATTTTGGCTATTTCTTCTATTAGTGTTTATGCAATTTTAATGTCTGGATGAGGAAGTAAGTCTAAATATGCTTTTTTGGGTGCTATTCGGGCGGCGGCTCAAATGATTAGTTATGAGGTTTCAATTGGGCTAATTCTTATTTCGGTTTTATTATGTGTTGGTTCTTTAAGTGTTACTGAAATTGTTTTAGCGCAAAATAATGGTATTTGGTTTTTTTTTCCACTATTTCCTGTTACAATAATGTTTTTTGCCTCGATTTTAGCAGAGACTAATCGTGCTCCTTTTGATTTAACAGAAGGAGAGTCAGAGCTTGTTTCGGGATATAATGTTGAGTATGCTTCGATGTCTTTTGCTTTATTTTTTCTTGCTGAATATGCACATATTATTTTAATGAGTTGTTTAACAACGATTTTTTTTTTGGGAGGATGGCTTTCTCCGATTCCAGATTTTAAGGGTGGGGCTGGATGGTTTGGTTTTAAAGTTGTTTTTATTATTTTTTTTTTTATTTGAGTAAGGGCTTCATTTCCTCGAATTCGATATGATCAGCTTATGGCTTTGTTATGAAAAGGGTATTTGCCTTTAAGTTTAGGAATGGTCATTTTTGTGGCTAGTGTTCTCTTTGGGTTTAATGGCCCCCCTCCTATTTAAAATATTTTAATTAATTATTATGAAAAAAAAAAAGAACTAACAGATTTGCAAGTGCCATTACGAAAAAAAAATCCTATTTTATTTTTAATCAACGGATTTTTAGTTGATTTGGTTTCTCCTTCTAACATTACTTATTTATGAAATTTTGGATCTTTATTGGGATTGTGTTTGATTTTACAGATAGTCACTGGGTGTTTTTTGTCTATGCATTATTGTTCTGATGTTAATTTTGCTTTTGCTTCAATTGGTCATATCATGCGAGATGTTAATTATGGGTTTTTATTAAGATATCTTCATGCTAACGGTGCTTCTTTGTTTTTTTTTTGTCTTTATGTTTATATTGGTCGAAGTTTATATTATGGGGGGTATTTAAAATTTCATGTTTGAAGCATTGGAGTTGTTATTTTTTTATTAACAATGGCGATTGCCTTTATGGGCTATGTTTTGCCTTGGGGACAAATGTCTTTTTGAGGGGCAACTGTAATAACTAATTTATTGTCTGCTATTCCTTATTTTGGGATTGATATTGTTCAGTGAGTGTGAGGCGGGTTTAGTGTTTCAAATGCAACATTAAATCGGTTTTTTAGTTTACATTTTTTACTTCCTTTTATTTTAGTTTTTCTTGTTATTCTTCATTTAGTTTATTTACATGTTGATGGGTCTAATAATTCAACAGGATTAAATTCTTCAATTGACGGGGTTTCATTCCATACTTTTTATACATCAAAAGATTTTTTTGGTTTTTTTTTTCTTTTTTTTTTATTTTGTTTTTTTGTTTTTTTTTTGCCAAATTTATTAGGAGATGCTGAAAATTTTATTCAAGCGAATTCTTTGGTTACTCCGGTTCATATTCAACCAGAATGATATTTTTTATTTGCTTATGCARTCTTACGTTCTATACCAAATAAATTAGGTGGGGTTATTGCAATGTTTTGTAGTATTTTTATTTTATTTTTTTTATCTATTTTACATCAAAGTCTTTTAAAGGGGCTTTTTTTTCGTCCTTTAGGTCGAATTGCATTTTGGTTTTTAATTATTGATTTTGCTTTATTAACTTGAATTGGATCACAAGTTGTAGAAGAACCTTTTATTTTAATTGGTCAAATACTTTCTTTTTTTTATTTTTTTTATTTTTTAGTTTTAATACCAGTTTTGGGTATTATTGAAAATCAATTATTAAATAAAAATTAATGAAATTTTTGTTTTAGGGGGTTTTCTTTTGAGCTTCGTGGTTTTAAGTCTTCGTTATTTTCTTTTAAAACTTTCTCTTTTTTATTTATTATTATTTTTTTGTTTTTTTTTTTTTAAATTAGAATGGGGAAAGCTTTTGGTTTTAATTGGTTCTTTTGCTGTCTTTCTTTTATTTGGACCAAAAAAAGAAGAACAAACAGATGTGCCTATTTTAAGTTTAATTATTGTTTTTGGAGTTTTTTGTTTAATTTCTTCAAGTAATTGACTTTCTATTTATTTAACCATTGAACTTTTTACTCTTTGTTTTTTTATTTTGATTGCTCGAGGGTCTGGTTATAGTGTGGAAGCAGGATTAAAATATTTTATTTTGGGTGCTCTTTCTTCTGGTTTATTTTTATTTGGGTGTGCTTTATTATGTGGTATTGGGGCTAATATACACTTTTCTCATATAGAACTTCTTTTTAATTCAAAACAAGTTTTTTCTGCTGTTTCGATACCAATTGGGTATCTTTTAATTATTGTTGCTCTTTTTTTTAAATTATCAGTTGCTCCTTTTCATATGTGAGTTCCTGATGTTTATGAGGGGGCACCTACTAAAATTGTTCTATTATTGGCTATTGTTCCAAAAATAGGGTTTTTTTCTCTTATAATTTCAATTGGATTGCCTGTAAATTTTTTTTTTTTAGGGATTCTTTTTTCTTTGTTTGTTGGAGCTTTGGGTGCTTTAAATCAAACTAAAATAAAACGACTTTTGGCTTATAGTGGGATTGGTCATATGGGCTTTATTTTATGAGGTTTGGAAAATGGTTCTTTTGAAAGTTTACAAGCTAGTCTTGTTTATCTTTTTATATATATTGTGATGACAATTTGTGCTTTTTCTATTATATTAAGTTTTAATGTTTATAAAAATTTGCTCGTTGAATTTAGTGGACTTTCTCGATACTTACCTTTTTTTTCGATTACTTTAGGTGTTCTTTTTTTTTCTATTGCAGGAATTCCTCCTTTTGCTGGATTTTTTGGAAAATGATTTATTTTGTTATCTGGAATTCTTTCTAAATCATATTTTATTTTTTTTTTTGCTGTTTTTTGTTCTGTTATAGCTGGGGTTTATTATATTCGAATTATAAAAATACTTTTTTTTCAAAAAAATTCTTTTCTTTTAATTACAATTAAAGCTTTAAAAAAAGAATCTAAATTAAATTTTAAAAAAGTTTTTTTAATTGGTTTTTGTTTTTATTTTATTCTTTTTTTTTTTCTTTCTCCACATTTTCTTTTTTTTTTTTTTTCTCAAATAATTTTTGATTTATTTTAAAATGGAATTTTTTTCTTTTTTTTTTATTTTAGGGATAATTGGCTCAGGAGTAATGGTTGTTTCAGCGTTAAATCCTGTTCTTTCTATTTTTTGATTGGTTCTTGTTTTTATAAATTCTGCAGTTTTTTTCCTTTTACTAGGAATAGATTTTCTTGCTTTGATGTTTTTACTTATTTATGTTGGGGCAATAGCTATTTTATTTTTATTTGTTATTATGTTATTAAATCTAACTGACTATCCTCCGGTTTTAAAAAGAGAGGTTGATATGACAAATTATATACCAATTGGATTTATAATTGGGATTTTTTTTTTTTCAGAAATTGCTTCAAGTGGGTTATTTTTGGGTTCTTTTCAAATAGAGAATTGAGACCTTTCTTTTCCTTGATTTCTTATTTCTTATCATAATATTGAGGCCTTAGGGCAGATTTTATATGTTTCTTGTTTTTGTTTATTTCTTTTGGCCAGTTTTATTTTATTAGTTGCTATGATTGGTGTGATTGTATTAACTCAAGAAACAGAATTTTTAAGTAAAAAACAAGATCTTTTTTTTCAAATAAATAGATAATGAATAGCTCTTCTTATTTTGAACAATTTAACATAGTGTGAATGTTTGGTTTTACGAATTCAACAATAATGATGACTTTTGTAATTATTGTTGTTTTATTACTTTTTAAAGGAATTGAATTAATTCCAAAAAGATGGCAATCGGTTTATGAATATCTAGAAAATTATTTTTATTATATAACAGTGCAAAATTTAGGAAATGTGGGTTTAATATATTTTTCTTTTATTGTTTCACTTTTTGTTTTTTTAGTTTTTTTAAATATTTTAGGTTTATGTCCTTATGTCTTCACACCGACAACCCATATTGTTATTACGCTTGGATTTTCTTTTTCTATTGTTATTGGAGTTACTTTTTCTGGATTTTATAAATTTAAAAAAGACTTCTTTAGCATTTTAATGCCTAGCGGCGCTCCTTTAATTTTAGCGCCACTTTTAATTTTAATAGAAACAGTAAGTTATATTTCTCGAGCTGTTTCTTTGGGCATTCGTTTAGCAGCAAATCTTTCTGCCGGGCATCTTTTATTTGCAATTTTGGCAGGATTTGGTTTAGTTTTTAAATTAGCAATGTCTATAATGGTGTTTATTACGTTATTGGAGGTGGCCGTTGCGGTTATCCAAGCGTATGTGTTTTGTCTGCTGACATTAATTTATTTAGCAGACACAATTTTTTTACATTAATC-----------------ATGAGCGGGGTAGAAATTTTTTTATGTTCATTAATGGGTGTGTCGGTGTGTTTTAATTATGCGTTCATGATGGCTTGATACAGCCAGTCTTCTCAAAGGTGACTTCAGCATCAAATGGATCTTCCAGAGAATACTGAATACGTGCATCCTTGAACTTATTATAGTTATGTGCGCCGTCATGGTATTGTTCATAATGGTGGTG-----GTGA--------TC--------GGCCAA--------------------------------GA----------GATGTTATTTTTATCGATCTAGAAGGGCGACAAGTGCCCTTAAGAAGTCGGCGATTTTTTAGGGTGGCGGAGA-----ATGACGAG-AGTTTGTGTGATAATGGTAATCGTTTGTGTCATGACGAGGGTTTGTGTGATAATGATGATCGTTTGTGTGTTCATGGCGAGAGTTTGTGTGATGATGATAGTTTGTGTGATAATGACGAGTTTCAAGATGGGGTTTTGCATTTA-------AG-TAAAAGTGCAAGTGAGTCAGAAAGTAGAGGTAGTTTGAGG----GAAAGTG-TAAT-TAAG----GGATTAAGTGAGTCAGAAAGTAGAGGTAGTTTGAGGGAAAGTGTAA---TTAAGGGATTAAGT-GAGTCAGAAA-------GTAGAGGTAGTTTGAGGGAAAG----TGTAATTAAGGGATTAAGTGATTCAAGAAGCGAAGACGAAGCGTTAAGGGCAGTA---GAGGTAGAAA-------CACATTTAAGTGGTGGC-CAGTTAGCGCGTACAAATCAAGGTGAGGCAGTTGAATCGACTCTTAGTTCGATCCCTTTTGAGGTTGAAGGGATGATGGTTCCTGTGTGAAATCAATTTGACATGCTGTCTAACATATTGGGAGATAAGCCCCTGGTAAGCTGCATTTTTTTATCTTACCAGCTTTTAAAAATAACAAATCAAATGCCAAACCCATGGGTTTATAGGTGATTTATTTTGGTATATATTTCATTAATCTTTGTTTTTTTAATGGTTTAG----TT-----------------AACATGG--------TCT--------------------CTGG------------------------------------------CA------------------------------------------------------------AGCAGAA--------------------------------------------TTTGG--------------------------------------------------------------------------GTGG---------------GTTTA---GAATGAGA---GGGGCAT------GAGAGCCAAA--AAATTAATA-GAGT------TGAGAGAAAAAGTTTTAAAATGGTTCTTTGTATTTGAATTAGCTAAAAATCAATAAAATTTTTATTTAAAGAATT-TCTTTTGTGTTTGTTATTGTAATGGTTCTTATTTATTTAATTGTTATTATGGCATTAGTTAACATCATAGGGACAGCGAGGGAGAGAAGATGTGTTTTAAAAAAGCGGGCTTTAGAGTGGTCTTTGGCTTTATTGTTTAGTACTTTAGTTTTTTGGGGTGGATTTGACGGAGAAAGTCATTTTCAATTTTTTAGTTTAGTAGAATGAAATATATTTTCTACTTTAGATTGAGGCCCGATTGTTTTTGCAGTCGATGGTGTTTCTTTGGTTTTTTTATTTTTAACGACTTTTTTAATTCCAATTTGTATTTTAATTAGCCAAAAATCAATAAAATTTTTATTTAAAGAATTTCTTTTGTGCTTGTTTTTTTTAGAAGTTTTATTAATAGGTGTTTTTATAGTGTTTGATCTTCTTTTGTTTTATCTTTTTTTTGAGGGGATATTAATACCAATGTTTTTTTTAATTGGTATTTGAGGCTCTCGAGAAGAAAAGGTTCGTGCTTCTTTTTATTTTTTTTTTTTTACTTTTATAGGCTCTCTCTTTTT-TTTTTTATAATACTTTTTTTATATCAAAGGATTGGAACAACAGACTATTTTCTTTTACTTAATATTAAATTATTTTTAAATATTCAAAAATGAGCCTTAGTTGGGATTTTTCTTAGTTTTGCAGTGAAACTACCTCTTATTCCATTTCATATTTGATTGCCACAAGCACATGTTGAGGCTCCTGTTGCGGGATCTGTTATTTTGGCTGGAATTTTATTAAAATTAGGGGGTTATGGCCTTTTGCGTTTTTCTTGACCTCTTTTTCCGGGGGCTTCTTTATATTGGTCTCCAGTTATTGTTTTTTTTAGTGTTGTTGCTGTTGTTTATGGAGGCTTAATGACATGTCGTCAAATTGATTTTAAACGGCTTGTTGCTTACTCTTCTGTTGCTCATATGGGACTTGTGCCTTTGGGTCTTTTTACACATGTTATAGAGGGGTTAATTGGGGCTCTTTTTTTAATGTTGGCGCACGGATTTGTTAGCTCTGCTCTTTTTATTGGAGTAACTTTTTTGTATGATCGCCATCATACTCGTTTAATAAAATATTATCGGGGTTTGACTTTGACAATGCCTCTTTTTGTTATTACAATGTTAATTTTGTCTTTGGCGAACATGGGTTTTCCTCTTAGTTGTAATTTTGTTGGAGAATTTTTTTCTTTATTAGCAGTCTTTCAATATCATTATGGAGTTGGAATGTTTGTTATTTTAGGGGTTCTTTTTTCTGCAATTTATTCTCTTAGTCTTTTTAATCGTATTTCTTTTGGTGGAGGATCTAATTATTTACTTTTTAATAGAGACTTAAGTCGACGAGAGATTTTTGTAATTTTTCCTTTTCTTTTAATTATCTTGGGGGGGGGGATTGTGCCTTTTCCTATTATTGATTTAATTAAAAATAGTCTTGTTTTTAGCCCGGGGGGTTAG------------GTCCTCGGGTTTTAGGGAGAAAAAAATATTTAGTCTAGGTGATACATGCTAGTGTATGCGAACAGGTGAGGATAA-AATAAAAAAATTTTTTTTATTTGTTGTATTAAAAAAAGAAAAGTTTTTTAT-TCTTTTTTTGAAGATGCATAGTTTAACCACATTTTCACTGAAACAAAGGAAAACCTTTGGCAGCAGTAACAGGTTTTATGTAATATACGAAAGTAAGACATAGGTGGGAGAAAAAAAATCTGTCAAATATAAGTGCCAGCAGACGCGGTAAAACTTAAGGATTTA-TTTTTTAATAAAAAGCAAAAAGCGTGTTAAGGATTTTTAAAAAAAAAAAATAAATAGAATTTTTTT-TCGTAATTGTGAAATGTTGGAATGAAAAAAAGAATTTTTTATATGAAAATAATTTA------TTTTTTTTTTCTTAAACACGAAGGTTTTGGGAGCAAACAGGATTAGAGACCCTGGTAGTCTATACAGTAAAAGAATATCGCTTGAGTAGTACGGTCGCAAGATTGAAATTCAAAAGACTTGGCTGTTCGGTTGTTAATTAGAGGAGCGCGTCGCTTAATTCGATAATCCGCGAGAGACCTTACCGAAGTTTGAATTTATTAACAGGTGTTGCATGGCCGTCGTCAATTTCTGTTTGAAACAAAAAGGGGTTTAATCCTATCAAGGTTGAAGTCAGGTAATATGACCCTTATATTTCGGGGTTAGGCGCGCTACATTTCTCTCTTAGAAAAGGAGGGGTTCGGATTGTTTTTAAAATAAGATAATGAAGTTGTATTTAATAGTAATTGAAAAGTAGTGCGTTTCAATGAACGTGAAAACAATGTTAGTACAAATAGCCCGTCGCCAGCCCAGAAATAGGCTGTAAGTCGTAACATAGTAAGAGTGAGGGAACTGGCTCTTGATGCAGTTTCATTCGTATCATTTAGTTGAGCCTTCTCCATGGCCTTTTGTTGGAGCAATTGGCTCTTTTTTTATTACTGTTGGTGCAGTGGTTTTTTTTCATTATGGGTTTAGTTTTTTTTTATATTTGGGGCTTTTGGTTGTTGTTGGAGTTATGTTTGTTTGATGACAAGATGTTATACGAGAATCAACTTTTCAAGGTCACCATTCTTTAATTGTAAAACAAGGGATTAAATATGGTATGATTTTATTTATTCTTTCAGAAATTTTGTTTTTTTTTTCTTTTTTTTGGGCTTTTTTTCATAGTAGTTTAGCACCGGTTGTTGAACTTGGAGTCGTTTGGCCTCCACAAGGAATTGTTGCATTAAATCCCTTTTCTGTTCCTTTATTAAATACTGCTGTCTTATTAAGTTCTGGGGCAACGGTTACATGGGCGCATCATGCAATACTTTGTGGTTTAAAAAAAGAGGCTCAATTTGCTTTATTTTTAACTCTTTTTTTGGGTGTTATGTTTACGGGGTTGCAAGCATTTGAATATTATGAGGCGCCTTTTACTTTATCTGACTCTGTTTATGGGGCCACTTTTTTTGTTGCGACAGGGTTTCATGGACTACATGTTATAATTGGTACTACTTTTCTTTTTATTTGTTTTTTACGTTTACTCTCTAATCAATTTACTCGTCGTCAACATGTTGGGTTTGAGGCCGCGAGTTGATACTGGCACTTTGTCGATGTGGTTTGATTATTTTTATATTTATGTATTTATTGATGAGGCTCATAAAAACTTCTTTAATGCAAAATCTTTTTGTTTTTACTTTTTTTAGATATTTTATAAATGTTTTGGCTTGGTTATCTGTTCTCTGGTTTAATCATCTTTATTATTCGGATAGTCCAGAGACTTGACTTTTAGAGTTTCAAGATGTTGGGGATCCTATTGTAGAAGAAATTGTTTTTTTTCATGACCAAGTTATGTTTTTATTAATTATTATAGTTACTGTTGTTTTATGACTTTTTGTTGAAGCTTTTAAAAATAAGTTTTATGATCGTCATTTAATCGATGGTACATTTTTAGAAATTGTTTGAACGATTATTCCTGCTGTTATATTGATTTTTATTGCATTGCCCTCTCTTAAATTATTATATTTAATGGATGAGGTTATTTCTCCAGCTTTGACAATTAAAGTTATTGGGCATCAATGATATTGATCCTATGAATATTCTGATTATGAAGGGGATACGCTAGGGTTTGATTCTTATATGATTCCTACTTCGGATTTAGTTTCTGGAGAGAATCGTTTGTTAGAAGTTGATTATAAACTTTTAATTCCTATTCAAACACATATAAGATTTTTAGTTACTGGGGCAGATGTTTTACATTCTTTTGCAGTGCCTTCTTTAGGGTTAAAAATCGATGCGGTTCCGGGTCGTCTAAATCAAACTGGTGTTTTTATAAAACGAGCCGGGGTTTTTTTTGGACAATGTTCTGAAATTTGTGGGGCAAATCATTCTTTTATGCCAATTGTGATAAAGGGAGTTGGTTTAAATGAATACGTTCAATATTTAAATTATTTAAAATGTATTATAAATACTTAGTTATTATTATTATTTTATTTTTATTGGGGAGTTGGGGTATAATTTTAAATAGAGGACATTTTATTATTATGCTTGTTTCTATTGAATTAGTTTTATTATCAACTTTTTTTTTTTTTTTAATAAGCTCTAAAGAAATAGATCTTTTAATAGAACAAATTTTTATGATTATGGGATTAACTATTGCTGCGGCAGAATCTTCAATTGGTTTAGCTATTTTAGTTGCATATTATAGAATTCGAGGAACAATTGTTTTAAAATCTTTTAGTTCTTTGCGAGGATAATGATTAATCTTTTTATTTTTTTTTTTCTTATTTTTATTTTGGCGGGGTTATTTATATTTCTTTCTTTTTTGATAGGGGAAAAAATTCCAGATCGAGAAAAGGTTTCTGCTTATGAATGTGGTTTTGCTCCCTTTAATTTTTTAGGACGCCCTTTTTCAATACGTTTTTTTTTAATTGGTATTTTATTCTTGATTTTTGATTTAGAAATCTCTTTTTTTTTTCCCTGATGTGTTTTATATAATTCGACTGCTCCGTTTGGGTTTTGAACTATGATAGGTTTTTTTTTTGTATTAGTTTTGGGTTTGATATACGAATGAGTAATGGGGGGATTAGAATGAGAGTAAAAAGAGAGTAAAAGAAAAAGTCCTATCTATTATGAAAATAATAGAAATTTTATACCAACTCCGGTTTCTGCTTTAATTCATGCTGCAACGATGGTTACTGCAGGTGTTTTTTTATTAATTCGTGCTTCTCCTTTATTTGATGTTGTTCCTCTTATATTAATTATTATTTCAATCGTTGGGGTTTTAACCGTGTTTATTGCCGGCACAATTGGTTTAGTTCAAAATGATTTAAAAAAAATAATCGCTTATTCTACTTGTAGTCAATTAGGTTATATGGTTGTTGCTTGTGGGCTTTCTCATTATGCAATTAGTCTTTTTCATCTTATGAATCATGCTTTTTTTAAAGCTTTATTATTTTTGAGTGCAGGGTCTCTTATTCATGCGGTAATTGATGAACAAGACATAAGAAAAATGGGGGGTTTATTATCTTTTCTTCCTTTGACTTATGTTTTTTTTTTGATAGGCTCTTTTTCTTTAATGGGGTTTCCTTTTTTAACAGGATTTTATTCAAAAGATTTAATTTTAGAATTTGCTTTTGGACAATTTTATTTAATTTTTGTGTATTGATTAGGTTGTTTTTCTGTTTTATTAACAATTATATACTCTATTCGTTTAATTTATTTAGTTTTTTTATCCAATATTAATTTAAAACGAGCAAACATTTTTTTTCTTAAAGAAGGAGAATTTTTATTTTTAATTCCTTTGGGTATATTAACTTTAGGAAGTGTTTTTTGAGGCTATTTAAGTAAAGAAATAATTTGGTCTTTTCAAATAGATGTTTTTTCAATACTTTCTTTAAAAATAAAAATATTTCCAATTTTATTTTGTTTTATTGGACTTTTTGGAACGATATTTTTTTTTTTTTTTTTTTCTTCTCAAATTTTTGGTTATCCCCTTCAGGTGATCGGATCTTCTGTTTTTTCTCTTTATAATTTTTTTGGTTCTGCTTGACAAATAAATTTTTTTTTTAATTTTTTCTTTATAAAAAAAATATATAAAATAGGACATCTAATTACTAATTTAACAATTGATAAAGGTTTATTAGAGGTTGTTGGGCCTAGGGGTGTTGTTCAATTTTTTATTTTTCAAACTCAAAAGTTGAGTAGTTTACAATCGGGGTTAGTATTTAATTATGCTTTAGTCTTTTTTATTGGGATACTTTTTTTAATTTTTGCACTTTAATTTAAATTTTTGAAGGAGTTAAGTTAAAGTAAACTGTTGATCTTCAAAATCAACTATGTAGGTGCAAGTCCTACACTCTTTGAGTGCCTCAGTTAAAAGTAAGCTTTTATAAGATACAATATTGATGGAGTTTTTCGGTTTTATTTTTATTGTTAATTTTTTTTGAGATTGTTGTTTTTCCTTTAATAAAACGTAATTGATGGATAAGAAAGTTCTTAATGAAGTGCGATGGCGCCATCTTAACAAAAATTT--GGTTACAAAAAGAAATTTATAAAAAAA----AAAAAAAAGTATGGTGTAATATTAAA-----------ATTTTTG---AA---GGAGTAAAGTTAAAGTA-AACTGTTGA-TCTTCAAAATT---AAC-TATGTAGGTACAA---------GTCTTTGGGGCCTCTCCCCGGTTCAATCTAAA-AATGATACTC---TTAAAATATT-TAA----TTAA---CTTGATTGATTTATTATTATCGAAGTGAA-GAAGCGGGCATTTTAACTTTAAAAAAAA----TTGTTT----ATCTTTA-------GTTGGTAGATTAAAAAGGATGGGGTTGAGTAACATTTTAGCTCTAGGAGCGGTAATAATTTTAGTTA-TAGGAG-TCCCAAAAATTTTTATTCAATTT-GATT--TGGA--AGTGGGTTGTTTTGT---------AAGAATTATTTTT------TTATTGC---GT-----------GAATGTT---TTA----AAAAAA-------AATTGTTTGTTAA--TG-----CTGAGGGTCCGC-CCTG--TAAACGTGCTAGAGTTGAAGAAGA--ACCATCGTCAAATATGCCCGGGGATATTGAAAATGAAGTAGTAGGCGGAGCCGAAAATGAAGAGGTAGCCAACCCAGTG-AGTACCGGAGTCG----GAGAA-AC-CGAT--GGAG-ATTTG------GGTTAT---TCAGCTGAGGTTTGGGAAGTATTAAATGGATT--TCAGAAGATCAAGAAGACGAATTAGTAGAGATGGTAATTGGGTGAGCAAACAGTG-TAACTTTTTTAATTTGAATTGAAACGGTCGGAGAGTCAAACGAGGAGTTAGTAGATGTGCTAAGGGAGTGAGCAGAGGGTG----ACG----TGTCCGAAGATTCAAACGACGAATCAGA---------AGAT----GAC-GCGTCTGAAGATTCAAACGA-CGAATAA-GTAG--AGGCT--TTAATATT-------------TAAGAGGTGAGAGGG---GC----------------------------AGGGG---ATATTTTTTTTTAATTTTTAGATTTT-GTT-ATGAG-----CTTGGGGCCTCCCCCCGGCTCAATT----------TGTAGATGGTTTCTAGTTATTGAATTCGATGAATTTTTTCAACCAATCATAAAGATATTGGTAGTTTGTATTTAATTTTTGGTGGAGGTGCTGGTTTAATTGGAACGGCGTTTAGTATGCTTATACGACTAGAGCTTTCTGCGCCCGGAGCGATGTTAGGAGATGATCATCTTTATAATGTAATTGTTACAGCACATGCTTTTATTATGATTTTTTTTTTGGTTATGCCGGTTATGATTGGGGGGTTTGGTAATTGATTGGTCCCATTATATATTGGGGCGCCGGATATGGCGTTTCCCCGACTAAACAATATTAGTTTTTGACTTTTGCCCCCTGCGCTTTTTTTATTATTAGGCTCTGCTTTTATTGAACAAGGGGCGGGGACGGGATGAACAGTTTATCCTCCTCTTGCTAGTATTCAAGCACACTCTGGAGGTTCGGTTGATATGGTTATTTTTAGTCTTCATTTAGCTGGGGTTTCTTCTATTTTAGGTGCTATAAACTTTATTACTACAATTTTAAATATGCGAGCCCCGGGTGTGTCTTTTAATAAACTACCTTTATTTGTTTGATCTATTTTAATAACAGCTTTTTTATTACTTTTATCTTTACCTGTTTTAGCTGGTGCTATTACTATGTTGTTAACAGATAGAAACTTTAATACGACTTTTTTCGATCCAGCGGGTGGCGGGGACCCAATATTATTTCAGCATCTATTTTGATTCTTTGGGCATCCAGAAGTTTATATTTTAATTTTGCCTGGTTTTGGTATGATTTCTCAAATAATCCCGACTTTTGTTGCTAAAAAACAAGTTTTCGGGTACTTAGGAATGGTTTATGCCATGCTTTCTATTGGGCTTCTGGGATTTATTGTTTGAGCTCATCATATGTTTACTGTTGGGATGGATGTAGATACAAGAGCATATTTCACTGCTGCTACTATGATTATTGCTGTGCCAACTGGGATTAAAGTTTTTAGTTGGTTGGCAACTATTTATGGAGGTGTTCTTAGGTTAGAGACTCCAATGCTTTGAGCTATGGGGTTTGTTTTTTTATTTACAGTTGGTGGTTTAACTGGGGTTGTATTAGCAAATAGTTCTCTTGATATTGTTCTACATGATACATATTATGTAGTTGCGCATTTTCATTATGTTCTTTCTATGGGGGCTGTTTTTGCTATTTTTGGGGGATTTTACTATTGAATTGGAAAAATAAGTGGTTATTGTTATAATGAATTTTTTGGGAAAGTTCATTTTTGATTAATGTTTATCGGGGTTAATTTAACTTTTTTCCCTCAACATTTTTTAGGTTTAGCAGGATTTCCAAGACGATACTCGGATTATGCAGATGCTTTTTTGGGTTGAAATTTAATAAGCTCTTTAGGGTCTATTATTTCTATTTTGAGTGTTGTTTGGTTTTTATATATTGTTTTTGATCTTTTTGTTACAGAAGAAAAATTTTTGGGTTGAAAAGAAGGGTTTTCTTTAGAATGAATTCATTCTTCTCCCCCCTTATTTCATACTTATGAGGAGTTGCCTTTTGTACAAAAA-TAAATAATTTTTAGAATAGTGCCGGGTTTATATACCGGTTT

hystri TGTAAGAAAGACAAAGGGTTAGTCATTGGGTTCATGCCCCAAATAAGTGAGTTCGAATCTCTCTCTTACACAAAATAAAAATTAT-AAAAAAAAAGGAGGATAACTAAGATAAATTAAAATTAGACGATTTTTTTCGAAATGGCGTTCAGTTGCTTATTGGCTGTTTTTCCATAAAAGTGA---TTTTTTTAATTTGAATTGAAACATCTTATTACTAAGTAAAAATCAAACGAGATTCCGAGAGTGGCAGAGAGTGAAATTGGAGTTGTGCTTGTGGTGGTCATAGATCTTGCTTAAACATTAGTTTATACTGATAATGAGAGTACTGTAAGGGAAAGTTGAAAGAGAGTTGAAAGGAATTTGAATTTTTTATTTTTAAAGTAGCTTTAAAAAAGCGTACCTTTTGTATAATGGGTAAAAGAGATTTATTTGGCATATTTAAAATGGAAAATTTTAAGTAATT-TTTTTTTAGTTAAGTTCCCCGAAACCAAGTGATTTAATCATGAATTGTGTGAGCAAAAACTGGTGATTGTGGCAAAAATCTCAGAAAATTTGTGATTAGGGGTGAAAGGCTAATCGAACTTGGAAATAGCTGGTTTTCTGCGAAAACTATTTAAGTAGTGTTC-TTTTTGTTTTTATTTTAATTGTCATTTTAAAAAAAACAAAAATAAAAAAAAAGTAAACAAACAATAAATAAAAAGATTTTTTGTTAAAAGAGAAAGCTCAAATCAGAAGCTATTGTCATTTTTTTTTTCTTTAGTTTAAAAAAAAATTAAATTTAGACAATAAAAATGTAGGCTTGGAACCAGCCATCTTTTAAAAAGTACGTAGTTGTTTATTTAAAAATTTTAAATTTTTTTATAATTTTGATGGTTAAAATAAAACAGAAGCTCTGAAAATAATTATATATTTTGTAGCAGAATAAATTGTTTT---T--------------------------TAGTTTGAAAAATTTTTT-TTAATACATCGTATTCAATTGTAATAATTTTTACATAAGTAATCTAAATATTATT-TTTTTTTCTTAAGACTACCTTGGTCAAGGGTAATACAGTTTCTAAAAAAGAAATTTTTTATGAAGATTTTTTATA-AAATATATAATATCGGGAAAAG-AAAAAAAAAGAACTGTTTTTCTAATTAAAGAAAGAAA-AAAAAAAGAAAAACATTTTTTTAAGGAACTCGGCAAAGTTAAACTTCGACTGTTTACCAAAAACATAGCTTTTTGATCTTTATAAAAGGTGAAACCTGCCCGATGGTTGTATCTTAATGTGTTTGTCTCGCCTGCTAATAAAGACAATTAAATGGCCGCGGTAACACCGACTGTGATAATGTAGCGTAATCAATTGTCAATTAATTGTTGACCGGTATGAATGGTGCCCCGAAAGTTTTTCTGTCCTAAAAAAATATTCAATGAAATTAAATTTGTAGTGAAGATGCTACATTTTAATTGTTAGACGAGAAGTCCCCATGGAGCTTTACTGTAAGCTTATATATATAA-ATTTTTTTTTTTATAAGCAAGACAGTTTTGTTGGGGCGACAGTTTTTTAAAAAGTAACGAAAATGAACTATGACGCATGTTTAACTTTGAAATTTTTTTAATTGATGAGACATTTTTGGTGTGTTTTTTGATCCGTTATTTTGAATGAAAAAAATAACGAAAACAAATAAAAGTTACCCTGGGGATAACAGCGCAATAAYGTTTGAGAGTTAATTAACGACAGTGTTTGCGACCTCGATGTTGAATTGTAACGTCCTACGGTGTAGTCACTCGTAAGGGTTGGTTTGTTCATCCATTAAAGTTATACATGATTTGAGTTAAAAGCGTGGTGACACAGCTTGGTTTCTATCTACAATTAGAAAACAAAAATATGTTGTTTTTTCGTACGAAAGGATCAAAAATCAATAGTTCCCTTAATATAACTATTCTTTAAATTAGGATTGCTTCTAAAAATAAAAAGAAATATTTTTGTTTGTTGTTTTTATTAATGTATCTTTTAGTTTTATTTTTTCCTTTAATTGGAGCTGTTTTAACAGGTTGTTTTGGAAGAAAAATTGGAGAAAGAGGAGCGGGGATTTTAACTTCAAGTTGTTTAGTTTTTAGTTTGTCTTATTCTTTTTTAATCGCGATTGAAGTTTTATTTAATTCAACAACAACGTATTTGAAATTATGAAAATGATTTGATTCGGGATTATTTGTTGTTTTTTTTGGTTTTCAATTTGATGGTTTAGTAGTTATTATGTTATTTGTTGTTTTTATTGTTTCTACTTTGGTTCATATTTTTTCTATTGCTTATATGCGGGGGGATCCTCATGTTCCTCGATTTATGACATATCTCTCTTTGTTTACTTTTTTAATGGTTTTATTGGTAACTAGCGATAATTTTCTTCAATTATTTATTGGGTGAGAAGGGGTTGGCCTTTGTTCTTATTTATTAATTAATTTTTGATTAACTCGATTAGAGGCAAATAGAGCTGCTATTAAAGCAATGTTAGTGAATAGAATTGGTGATATTGGGTTGCTTTTAGCAATGTTTTTACTTTGGGATCTTTTTGGATCTCTAGATTTTTCTACAATTTTTAATTCTATTTTTTTTTCTAATCAAATATTTTTTATTTGTTTATTTTTATTTTTTGGGGTTATGGGAAAATCTGCTCAATTAGGATTACATACTTGGCTACCGGATGCAATGGAAGGTTATTGGGCCTTTTAATTAAAAAATTAATTAAAAAAATTACTATACATTAAAAAAACAATTTATTAGTAAAATAATTGTTTATTAATGGACAATAAAATATTTATTTTAATGTCTAAGAGACTTTATGTGATTTACTTTTTTTTATTTGATTAAATCTATTATTATTATTATTCCTTTACTTATTGCTGTTGCATTTTTAACTTTAGCAGAACGAAAAATTTTAGGGTATATGCAAATAAGAAAAGGGCCAAATGTTGTTGGAGGGGGGATCCTTCAACCTTTTGCAGATGGGGTGAAATTATTTATTAAAGAAATGATTCTTCCCCATCAAGCAAATAAATTTGTTTATCTTTTGGCTCCAGTTATATCTTTTACATTAGCTTTCTTTGTTTGAGGTTTTCTTCCTTATGAGAAAGGAGTGAGTATTAGTGATTTTAAAATTAGTCTTTTATGAATTTTGGCTATTTCTTCTATTAGTGTTTATGCAATTTTAATGTCTGGATGAGGAAGTAAGTCTAAATATGCTTTTTTGGGTGCTATTCGGGCGGCGGCTCAAATGATTAGTTATGAGGTTTCAATTGGGCTAATTCTTATTTCGGTTTTATTATGTGTTGGTTCTTTAAGTGTTACTGAAATTGTTTTAGCGCAAAATAATGGTATTTGGTTTTTTTTTCCACTATTTCCTGTTACAATAATGTTTTTTGCCTCGATTTTAGCAGAGACTAATCGTGCTCCTTTTGATTTAACAGAAGGAGAGTCGGAGCTTGTTTCGGGGTATAATGTTGAGTATGCCTCGATGTCTTTTGCTTTATTTTTTCTTGCTGAATATGCCCATATTATTTTAATGAGTTGTTTAACAACGATTTTTTTTTTGGGAGGATGGCTTTCTCCGATTTCAGATTTTAAGGGTGGGGCTGGATGGTTTGGTTTTAAAGTTGTTTTTATTATTTTTTTTTTTATTTGAGTAAGGGCTTCCTTTCCTCGAATTCGATATGATCAGCTTATGGCTTTGTTATGAAAAGGGTATTTGCCTTTAAGTTTAGGAATGGTCATTTTTGTGGCTAGTGTTCTCTTTGGGTGTAATGGCCCCCCTCCTATTTAAAATATTTTAATTAATTA--------------------------------AAGTGCCATTACGAAAACAAAATCCTATTTTATTTTTAATCAACGGATTTTTAGTTGATTTGGTTTCTCCTTCTAACATTACTTATTTATGAAATTTTGGATCTTTATTGGGATTGTGTTTGATTTTACAGATAGTCACTGGGTGTTTTTTGTCTATGCATTATTGTTCTGATGTTAATTTTGCTTTTGCTTCAATTGGTCATATCATGCGAGATGTTAATTATGGGTTTTTATTAAGATATCTTCATGCTAACGGTGCTTCTTTGTTTTTTTTTTGTCTTTATGTTCATATTGGTCGAAGTTTATATTATGGGGGGTATTTAAAATTTCATGTTTGAAGCATTGGAGTTGTTATTTTTTTATTAACAATGGCTATTGCTTTTATGGGCTATGTTTTGCCTTGGGGACAAATGTCTTTTTGAGGGGCAACTGTAATAACTAATTTATTGTCTGCTATTCCTTATTTTGGGATTGATATTGTTCAGTGAGTGTGAGGCGGGTTTAGTGTTTCTAATGCAACATTAAATCGGTTTTTTAGTTTACATTTTTTACTTCCTTTTATTTTAGTTTTTCTTGTTATTCTCCATTTAGTTTATTTACATGTTGATGGGTCTAATAATTCAACAGGATTAAATTCTTCAATTGACGGAGTTTCATTTCATACTTTTTATACATCAAAAGATTTTTTTGGTTTTTTTTTTCTTTTTTTTTTATTTTGTTTTTTTGTTTTTTTTTTGCCAAATTTATTAGGAGATGCTGAAAACTTTATTCAAGCGAATTCTTTGGTTACTCCGGTTCATATTCAACCAGAATGATATTTTTTATTTGCTTATGCAATATTACGTTCTATACCAAATAAATTAGGTGGGGTTATTGCAATGTTTTGTAGTATTTTTATTTTATTTTTTTTATCTATTTTACATCAAAGTCTTTTAAAGGGGCTTTTTTTTCGTCCTTTAGGTCGGATTGCATTTTGGTTTTTAATTATTGATTTTGCTTTATTAACTTGAATTGGATCACAAGTTGTAGAAGAACCTTTTATTTTAATTGGTCAAATACTCTCTTTTTTTTATTTTTTTTATTTTTTAGTTTTAATACCAGTTTTGGGAATTATTGAAAATCAATTATTAAATAAAAATTAATGAAATTTTTGTTTTAGGGGGTTTTCTTCTGAGCTTCGTCGTTTTAAGTCTTCGTTATTTTCTTTTAAAACTTTCTCTTTTTTATTTATTATTATTTTTTTGTTTTTTTTTTTTTAAATTAGAATGGGGAAAGCTTTTGGTTTTAATTGGTTCTTTTGCTGTCTTTCTTTTATTTGGACCAAAAAAAGAAGAACAAACAGATGTGCCCATTTTAAGTTTAATTATTGTTTTTGGAGTTTTTTGTTTAATTTCTTCAAGTAATTGACTTTCTATTTATTTAACCATTGAACTTTTTACTCTTTGTTTTTTTATTTTGATTGCTCGAGGGTCTGGTTATAGTGTGGAAGCAGGATTAAAATATTTTATTTTGGGTGCTCTTTCTTCTGGTTTATTTTTATTTGGGTGTGCTTTATTATGTGGTATTGGGGCTAATATACACTTTTCTCATATAGAACTTCTTTTTAATTCAAAACAAGTTTTTTCTGCTGTTTCGATACCAATTGGGTATCTTTTAATTATTGTTGCTCTTTTTTTTAAATTATCAGTTGCTCCTTTTCATATGTGAGTTCCTGATGTTTATGAGGGGGCACCTACTAAAATTGTTCTATTATTGGCTATTGTTCCAAAAATAGGGTTTTTTTCTCTTATAATTTCAATTGGATTGCCTGTAAATTTTTTTTTTTTAGGGATTCTTTTTTCTTTGTTTGTTGGAGCTTTGGGTGCTTTAAATCAAACTAAAATAAAACGACTTTTGGCTTATAGTGGGGTTGGCCATATGGGCTTTATTTTATGGGGTTTGGAAAATGGTTCTTTTGAAAGTTTACAAGCTAGTCTTGTTTATCTTTTTATATATATTGTGATGACAATTTGTGCTTTTTCTATTATATTAAGTTTTAATGTTTATAAAAATTTACTTGTTGAATTTAGTGGACTTTCTCGATACTTACCTTTTTTTTCGATTACTTTAGGTGTTCTTTTTTTTTCTATTGCAGGAATTCCTCCTTTTGCTGGATTTTTTGGAAAATGATTTATTTTGTTATCTGGAATTCTTTCTAAATCATATTTTATTTTTTTTTTTGCTGTTTTTTGTTCTGTTATAGCTGGGGTTTATTATATTCGAATTATAAAAATACTTTTTTTTCAAAAAAATTCTTTTCTTTTAATTACAATTAAAGCTTTAAAAAAAGAATCTAAATTAAATTTTAAAAAAGTTTTTTTAATTGGTTTTTGTTTTTATTTTATTCTTTTTTTTTTTCTTTCTCCACATTTTCTTTTTTTTTTTTTTTCTCAAATAATTTTTGATTTATTTTAAAATGGAATTTTTTTCTTTTTTTTTTATTTTAGGGATAATTGGCTCAGGAGTAATGGTTGTTTCAGCGTTAAATCCTGTTCTTTCTATTTTTTGATTGGTTCTTGTTTTTATAAATTCTGCAGTTTTTTTCCTTTTACTAGGAATAGATTTTCTTGCTTTGATGTTTTTACTTATTTATGTTGGGGCAATAGCTATTTTATTTTTATTTGTTATTATGTTATTAAATCTAACTGACTATCCCCCGGTTTTAAAAAGAGAGGTTGATATGACAAATTATATACCAATTGGATTTATAATTGGGATTTTTTTTTTTTCAGAAATTGCTTCAAGTGGATTATTTTTGGGTTCTTTTCAAATAGAGAATTGAGATCTTTCTTTTCCTTGATTTCTTATTTCTTATCATAATATTGAGGCCTTAGGGCAGATTTTATATGTGTCTGGTTTTTGTTTGCTTCTGCTGGCCAGTTTTATTTTATTAGTTGCTATGATTGGTGTGATTGTATTAACTCAAGAAACAGAATTTTTAAGTAAAAAACAAGATCTTTTTTTTCAAATAAACAGATAATGAATAGTTCTTCTTATTTTGAACAATTTAACATAGTGTGAATGTTTGGTTTTACGAATTCAACAATAATGATGACTTTTGTAATTATTGTTGTTTTATTACTTTTTAAAGGAATTGAATTAATTCCAAAAAGATGGCAATCGGTTTATGAATATCTAGAAAATTATTTTTATTATATAACAGTGCAAAATTTAGGAAATGTGGGTTTAATATATTTTTCTTTTATTGTTTCACTTTTTGTTTTTTTAGTTTTTTTAAATATTTTAGGGTTATGTCCTTACGTCTTTACACCGACAACGCATATCGTTGTTACTCTTGGGTTTTCTTTTTCTATTGTTATTGGAGTTACTTTTTCTGGATTTTATAAATTTAAAAAAGACTTCTTTAGCATTTTAATGCCTAGCGGTGCTCCTTTAATTTTAGCACCACTTTTAATTTTAATAGAAACAGTAAGTTATATTTCTCGGGGGGTTTCTTTGGGGATTCGTTTAGCAGCAAACCTTTCTGCCGGGCATCTTTTATTTGCAATTTTGGCAGGATTTGGTTTGGTTTTTAAACTAGCAATGTCTATAATGGTGTTTATTACGCTATTAGAGGTAGCCGTTGCGATAATACAGGCATATGTATTTTGTCTGCTCACATTAATTTATTTAGTAGACACAATTGTTCTACATTAAAAGTGATAAAATTAGAGATATGAGTGAATTAGAAATTATTTTTTATATAATATTTTTTGTGTCAGCAGGTTTCAATTGAGGTTTTTTTGTTATGTGGTATAATTCGTCTTCCCAAAGGTGGCTCCGGCATCAGATGGG-----TA----CTGATGATTGGGTGCATCCTCATAACTATATAAGTTATGTGCGCCGTCATGGTATTGC---------TGAGAGACAAGT------------------AGGGCGAGA--------GGGGTCAACCGAGGTAAT----------------GTATG---------TAGAGTCGGGGAGGC-----GACCACGAAAT--GGGGGTTTAG-------------------AAGATGGTGAGC--CAGAAAGTATTGAGAACTTGA---G--------AGAGTG--TGT-CGT------------------------------------------------TAGGGCATTACGTCA-----GTTAGAAAGTAGAGAGAAT---TTGAGAGAGGGTGAAACTAGCGCA-TTAAGTGAGTCA------GAAAGTAGAGAGAATTTGAGAGAGGGTGAAACTAGCGCATTAAGTGAGGCAGAAA---------G-TAGAGACAAT---------TTGAGGGA--GAGTGTAGCTAG---CGCATT-----AAGTGAGTCAGAAAGTAG-AGGCG-ATTTAAGGG--AGAGT-GT-----AACT--AGGGTATTACGTGAGTCAAGAAGCCAAGAC-GAAACAT---A----TGTATGTGATGGCAGGGT--------AAACAT--TGATGAGGCAGTTGAATCGACTCTTAGTTCGATCTCTTTTGAGGTTGGAGAGACGATGGTTCATGTGTGAAATCAATTTGACATGCTGTTTAACATATTGGCATGTAGGCCCCTGGTAGGCTGCATTTTTTTATCTTACCAGCTTTTAAAAATAGCAAATCAAATGCCAAACCCACGGGTTTATAGGTGATTTGTTTTGGTGTATATTTTATTAATCTTTGTTTTTTT-----TTTCCCT--------------------------------------TAG----TTATT------------------TTAGATTTA----------------TTTAACA--------------TGG----------------------TCTCTGGC--------------AAGCAGA-----------------------A-----------------------------------------TTTGGGT---GGGGCA-------------------------------------TTAGCCTTAAAG---G--------CTAG------------------------------AAAAGAGAGCAAAAA-A----TTTAATAGAGT----TGAGAAAAAAAGTTTTAAAATGGTTCTTTGTATTTAAATTAGCTAAAAATCAATAAAATTTTTATTTAAAGAGTTTTTTTTTGTGTTTGTTATTGTAATGGTTCTTATTTATTTAATTGTTATTATGGCATTAGTTAACATCATAGGGATAGGGAGGGAGAGAAGATGTGTTTTAAAAAAGCGGGCTTTAGAGTGGTCTTTGGCTTTATTGTTTAGTACTTTAGTTTTTTGGGGCGGATTTGACGGAGAAAGTCATTTTCAATTTTTTAGTTTAGTAGAATGAAATATATTTTCTACTTTAGATTGAGGCCCGATTGTTTTTGCAGTCGATGGTGTTTCTTTGGTTTTTTTACTTTTAACGACTTTTTTAATTCCAATTTGTATTTTAATTAGCCAAAAATCAATAAAATTTTTATTTAAAGAATTTCTTTTGTGCTTGTTTTTTTTAGAAGTTTTATTAATAGGTGTTTTTATAGTGTTTGATCTTCTTTTGTTTTATCTTTTTTTTGAGGGGATATTAATACCAATGTTTTTTTTAATTGGTATTTGAGGCTCTCGAGAAGAAAAGGTTCGTGCTTCTTTTTATT-TTTTTTTTTTACTTTTATAGGCTCTCTCTTTTTTTTTTTTATAATACTTTTTTTATATCAAAGGATTGGAACAACAGACTATTTTCTTTTACTTAATATTAAATTATTTTTAAATATTCAAAAATGAGCCTTAGTTGGGATTTTTCTTAGTTTTGCGGTGAAACTACCTCTTATTCCATTTCATATTTGATTGCCACAAGCACATGTTGAGGCTCCTGTTGCGGGCTCTGTTATTTTGGCTGGAATTTTATTAAAATTAGGGGGCTATGGCCTTTTCCGTTTTTCTTGACCTCTTTTTCCGGGGGGTTCTTTATATTGGTCTCCAGTTATTGTTTTTTTTAGTGTTGTTGCTGTTGTTTATGGAGGCTTAATGACATGTCGTCAAATTGATTTTAAACGGCTTGTTGCTTACTCTTCTGTTGCTCATATGGGACTTGTGCCTTTGGGTCTTTTTACACATGTTATAGAGGGGTTAATTGGGGCTCTTTTTTTAATGTTGGCGCACGGATTTGTTAGCTCTGCTCTTTTTATTGGAGTAACTTTTTTGTATGATCGCCATCATACTCGTTTAATAAAATATTATCGGGGTTTGACTTTGACAATGCCTCTTTTTGTTATTACAATGTTAATTTTGTCTTTGGCGAACATGGGTTTTCCTCTTAGTTGTAATTTTGTTGGAGAATTTTTTTCTTTATTAGCAGTCTTTCAATATCATTACGGAGTTGGAATGTTTGTTATTTTAGGGGTTCTTTTTTCTGCAATTTATTCTCTTAGTCTTTTTAATCGTATTTCTTTTGGTGGAGGATCTAATTATTTACTTTTTAATAGAGACTTAAGTCGACGAGAGATTTCTGTAATTTTTCCTTTTCTTTTAATTATCTTGGGGGGAGGGATTGTGCCTTTTCCTATTATTGATTTAATTAAAAATAGTCTTGTTTTTAGTCCGGGGGGTTAAGGCGGAGGATTTTTCCTCCAGTCTTAGGGAAAAAAAAATATTTAGTTTAGGTTATACATGCTAGTGTGTGCGAATGGGTGAGGATAAAAATAAAAAAAGTTTTTTT-TTTGTTGTATTAAAAAAAGAAAAGTTTTTTATTTTTTTTTTTGAAGATGCATGGTCTAACCACATTTTCCCTGAAACAAAAGAAAACCTTTGGCAGCAGTAACAAATTTTATGAAATATACGAAAGTAGGACATAGGTAAGAGAAAAAAAATCTGTCAAATAGAAGTGCCAGCAGACGCGGTAAGACTTAAGGATTTATTTTTTTTATAAAAAGCAAAAAGCGTGTTAAGGATT----AAAAAAAAAAATAAATAGAATTTTTTTG--GTAATTGTGAAATGTTAGAATGAAAAAAAGAATTTTTTATATGAAAATAATTTATTTTTTTTTTTTTTTTCTTAAAGACGAAGGTTTTGAGAGCAAACAGGATTAGAGACCCTGGTAGTCTATACAGTAAAAAAATATCGCTTGAGTAGTACGGTCGCAAGATTAAAATTCAAGAGACTTGGCTGTTCGGTTGTTAATTAGAGGAGCGCGCCGCTTAATTCGATAATCCGCGAGAGACCTTACCGAAGTTTGAATCTATAAACAGGTGTTGCATGGCCGTCGTCAATTTCTGTTTAAGACAAAGAGGGGTTTAATCCTATCAAGGTTGAAGTCAGGTATTATGACCCTTATATTTCGGGGTTAGGCGCGCTACATTTCTCTCTTAGAAAAGGAGGGGTTCGGATTGTCTTTAAAATAAGATAATGAAGTTGGATTTAATAGTAATTGAAAAGTAGTGCGTTTCAATGAACGTGAAAACAATGTTAGTACACATAGCCCGTCGCCAGCCCAGAAATAGGCTGTAAGTCGTAACATAGTAAGAGTGAGGGAACTGGCTCTTGATGCAGTTTCATTCGTATCATTTAGTTGAGCCTTCTCCATGGCCTTTCGTTGGAGCAATTGGCTCTTTTTTTATTACTGTTGGTGCAGTGGTTTTTTTTCATTATGGGTTTAGTTTTTTTTTATATTTGGGGCTTTTGGTTGTTGTTGGAGTAATGTTTGTTTGATGACAAGATGTTATACGAGAATCAACTTTTCAAGGTCACCATTCTTTAATTGTAAAACAAGGGATTAAATATGGTATGATTTTATTTATTCTTTCAGAAATTTTGTTTTTTTTTTCTTTTTTTTGGGCTTTTTTTCATAGTAGTTTAGCACCGGTTGTTGAACTTGGAGTCGTTTGGCCTCCACAAGGAATTGTTGCATTAAATCCCTTTTCTGTTCCTTTATTAAATACTGCTGTATTATTAAGTTCTGGGGCAACGGTTACATGGGCGCATCATGCAATACTTTGTGGTTTAAAAAAAGAGGCTCAATTTGCTTTATTTTTAACTCTTTTTTTGGGTGTTATGTTTACGGGGTTGCAAGCATTTGAATATTATGAGGCGCCTTTTACTTTATCTGACTCTGTTTATGGGGCCACTTTTTTTGTTGCGACAGGGTTTCATGGACTACATGTTATAATTGGTACTACTTTTCTTTTTATTTGTTTTTTACGTTTACTTTCTAATCAATTTACTCGTCGTCAACATGTTGGGTTTGAAGCCGCGAGTTGATATTGGCACTTTGTCGATGTGGTTTGATTATTTTTATATTTATGTATTTATTGATGAGGCTCATAAAAACTTCTTTAATGCAAAATCTTTTTGTTTT-------------T-A-T---AAATGTTTTGGCTTGGTTATGTGTCCTCTGGTTTAATCATCTTTATTATTCGGATAGTCCAGAGACTTGACTTTTAGAGTTTCAAGATGTTGGGGATCCTATTGTAGAAGAAATTGTTTTTTTTCATGACCAAGTTATGTTTTTATTAATTATTATAGTTACTGTTGTTTTATGACTTTTTGTTGAAGCTTTTAAAAATAAGTTTTATGATCGTCATTTAATCGATGGTACATTTTTAGAAATTGTTTGAACGATTATTCCTGCTGTTATATTGATTTTTATTGCATTGCCCTCTCTTAAATTATTATATTTAATGGATGAGGTTATTTCTCCAGCTTTGACAATTAAAGTTATTGGGCATCAATGATATTGATCCTATGAATATTCTGATTATGAAGGAGATACGCTAGGGTTTGATTCTTATATGATTCCTACTTCGGATTTAGTTTCTGGAGAGAATCGTTTGTTAGAAGTTGATTATAAACTTTTAATTCCTATTCAAACACATACAAGATTTTTAGTTACTGGGGCAGATGTTTTACATTCTTTTGCAGTTCCTTCTTTAGGGTTAAAAATCGATGCGGTTCCGGGTCGTCTAAATCAAACTGGTGTTTTTATAAAACGAGCCGGGGTTTTTTTTGGACAATGTTCTGAAATTTGTGGGGCAAATCATTCTTTTATGCCAATTGTGATAAAGGGAGTTGGTTTAAATGAATACGTTCAATATTTAAATTATTTAAAATGTATTATAAATACTTAGTTATTATTATTATTTTATTTTTATTGGGGAGTTGGGGTATAATTTTAAATAGAGGACATTTTATTATTATGCTTGTTTCTATTGAATTAGTTTTATTATCAACTTTTTTTTTTTTTTTAATAAGCTCTAAAGAAATAGATCTTTTAATAGAACAGATTTTTATGATTATGGGATTAACTATTGCTGCGGCAGAATCTTCAATTGGTTTAGCTATTTTAGTTGCATATTATAGAATTCGAGGAACAATTGTTTTAAAATCTTTTAGTTCTTTGCGAGGATAATGATTAATCTTTTTATTTTTTTTTTTCTTATTTTTATTTTGGCGGGGTTATTTATATTTCTTTCTTTTTTGATAGGGGAAAAAATTCCAGATCGAGAAAAGGTTTCTGCTTATGAATGTGGTTTTGCCCCCTTTAATTTTTTAGGGCGTCCTTTTTCAATACGTTTTTTTTTAATTGGTATTTTATTCTTGATTTTTGATTTAGAAATCTCTTTTTTTTTCCCCTGATGTGTTTTATATAATTCGACTGCTCCGTTTGGGTTTTGAACTATGATAGGTTTTTTTTTTGTATTAGTTTTGGGTTTGATATACGAATGAGTAATGGGGGGATTGGAATGAGAGTAAAAAGAGAGTAAAAGAAAAAGTCCTATCTATTATGAAAATAATAGAAATTTTATACCAACTCCGGTTTCTGCTTTAATTCATGCTGCAACGATGGTTACTGCAGGTGTTTTTTTATTAATTCGTGCTTCTCCTTTATTTGATGTTGTTCCTCTTATATTAATTATTATTTCAATCGTTGGGGTTTTAACCGTGTTTATTGCAGGCACAATTGGTTTAGTTCAAAATGATTTAAAAAAAATAATCGCTTATTCTACTTGTAGTCAATTAGGCTATATGGTTGTTGCTTGTGGGCTTTCTCATTATGCAATTAGTCTCTTTCATCTTATGAATCATGCTTTTTTCAAAGCTTTATTATTTTTGAGTGCTGGGTCTATTATTCATGCGGTAGTTGATGAACAAGACATAAGGAAAATGGGGGGTTTATTGTCTTTTCTTCCTTTGACTTATGTTTTTTTTTTGATAGGCTCTTTTTCTTTAATGGGGTTTCCTTTTTTAACAGGATTTTATTCAAAAGATTTAATTTTAGAATTTGCTTTTGGACAATTTTATTTAATTTTTGTGTATTGATTAGGTTGTTTTTCTGTTTTATTAACAATTACATACTCTATTCGTTTAATTTATTTAGTTTTTTTATCCAATATTAATTTAAAACGAGCAAACATTTTTTTTCTTAAAGAAGGAGAATTTTTATTTTTAATTCCTTTGGGTATATTAACTTTAGGAAGTGTTTTTTGAGGCTATTTAAGTAAAGAAATAATTTGGTCTTTTCAAATAGATGTTTTTTCAATACTTTCTTTAAAAATAAAAATATTTCCAATTTTATTTTGTTTTATTGGACTTTTTGGAACGATATTTTTTTTTTTTTTTTTTTCTTCTCAAATTTTTGGTTATCCCCTTCAGGTGGTCGGATCTTCTGTTTTTTCTCTTTATAATTTTTTTGGTTCTGCTTGACAAATAAATTTTTTTTTTAATTTTTTCTTTATAAAAAAAATAYATAAAATAGGACATTTAATTACTAATTTAACAATTGATAAAGGTTTATTAGAGGTTGTCGGGCCCAGGGGTGTTGTTCAATTTTTTATTTTTCAAACTCAAAAGTTGAGTAGTTTACAATCGGGGTTAGTATTTAATTATGCTTTAGTTTTTTTTCTTGGGATACTTTTTTTAATTTTTGCACTTTAATTAAAATTTTTGAAAGAGTTAAGTTAAAGTAAACTGTTGATCTTCAAAATCAACGATGTAGGTGCAAGTCCCACACTCTTTGAGTGCCTCAGTTAAAAGTAAGCTTTTATAAGATACAATATTGATGGGGTTTTTCGGTTTTATTTTTATTGTTAATTTTTTTTGAGATTGTTGTTTTTCCTTTAATAAAACGTAATTGATGGATAAGAAAGTTCTTAATGAAGTGCGATGGCGCCATCTTAACAAAAATTT--GGTTACAAAAAGAAATTTATAAAAAAA----AAAAAAAAGTATGGTGTAATATT---AAAATGTTTGTACTTTAACTTAATTTTTAATTGGTTTAG-CTATTTTAGTTGGATATTATAAAATGCAAAAAACAATTGTTTTAAAATCTTTTGATATTAAAAATTTATGGAAGAGTT-----A--------------------AGTT--------A-------A---AGTAAACTGTTGATCTTCAAAATCAACGATGTAG---------------------GTGCAAGTCCCACACTCTTTGAG--TGCCTCAAAAGTAT---TTAGCT--TTTGCA--AAAAATAGTATGTCTTGAGTTGATATGT--TCA-AAATTATGTGGTTGGGTAGAAGTTTA--------G--TTATAAGAGTTGCAATACTTTTTATTTTGGCATTAATAATAGTTATTTTTAGATTTAAAGTGAGTTATTTTATAATAATTGTTAGTATATTGTATAAGTGTTTAAAAAAAAATTTGTTCGTT--CATGCTGAGGGTCCGC-CCCG--TAAGCGTGCTAGAGTTAAAAAAAA--ACCATTGTGAAATATG----------------------------CCAAAAAAG--ATTAAAAATG---------AAGTAAA-----AGAGAGAGCCCAAAATAA--AGATTTGGGTTATCCAGCTGAGGTTTAAAAACCATTAAATTTACTTGAAT--TTAAAT--ACTTTACAAAAGAGCAAAAAGACAAATTAGTGGCGGTGTTAATTGAGTGAGCAAACAGTGTC---------------------G-CGGCCGGAGAGCCAAACGAGGGGTTATAAAATGTGTTAAT--AGG--G---TGGGCAGAAAGCG-AAGTGTCTGAAGATTTAAACGACGAATCTGAGGAGAACCCCGGAGAATAAGTCGG-CAGAGTA--GGACCCCGGGGAGTAAGTAG--AGGCAGGGTCATGGTCTCCTAG---CCCCAAGTCTTGAGATAGAGGGTTC-CAAGCCATAGAATTCG-ACATAAGGGGCGTTAGGGGCAAGCCCAT--GATGTCAAATCTTGTT------TCGGCCGCGGTGTACTAGCCCCAAATCTTGAGA---------TAAATGGTTTCTAGTCATTGAATTCGATGGATTTTTTCAACCAATCATAAAGATATCGGTAGTTTGTATCTAATTTTTGGTGGGGGTGCTGGTTTAATCGGGACGGCGTTTAGTATGCTTATACGACTCGAGCTTTCTGCGCCCGGAGCGATGTTAGGAGATGATCATCTTTATAATGTAATTGTTACAGCACATGCTTTTATTATGATTTTTTTTTTGGTTATGCCCGTTATGATTGGGGGGTTTGGTAATTGATTGGTCCCATTATATATTGGGGCGCCGGATATGGCGTTTCCCCGACTAAACAATATTAGTTTTTGACTTTTGCCCCCTGCGCTTTTTTTATTATTAGGCTCTGCTTTTATTGAACAAGGGGCGGGGACGGGGTGAACAGTTTATCCTCCTCTTGCTAGTATTCAAGCACACTCCGGAGGTTCGGTTGATATGGTTATTTTTAGTCTTCATTTAGCTGGGGTTTCTTCTATTTTAGGTGCTATAAACTTTATTACTACAATTTTAAATATGCGAGCCCCGGGTGTGTCTTTTAATAAACTACCTTTATTTGTTTGATCTATTTTAATAACAGCTTTTTTATTGCTTTTATCTTTACCTGTTTTAGCTGGTGCTATTACTATGTTGTTAACAGATAGAAACTTTAATACGACTTTTTTCGATCCAGCGGGTGGCGGGGACCCAATATTATTTCAGCATCTATTTTGATTCTTTGGGCATCCAGAAGTTTATATTTTAATTTTGCCTGGTTTTGGTATGATTTCTCAAATAATCCCGACTTTTGTTGCTAAAAAACAAGTTTTCGGGTATTTAGGAATGGTTTATGCCATGCTTTCTATTGGGCTTCTGGGATTTATTGTTTGAGCTCATCATATGTTTACTGTTGGGATGGATGTAGATACAAGAGCATATTTTACTGCTGCTACTATGATTATTGCTGTGCCAACTGGGATTAAAGTTTTTAGTTGGTTGGCAACTATTTATGGAGGTGTTCTTAGGTTAGAGACTCCAATGCTTTGAGCTATGGGGTTTGTTTTTTTATTCACAGTTGGTGGTTTAACTGGGGTTGTATTAGCAAATAGTTCTCTTGATATTGTTCTACATGATACATATTATGTAGTTGCGCATTTTCATTATGTTCTTTCTATGGGGGCTGTTTTTGCTATTTTTGGGGGATTTTACTATTGAATTGGAAAAATAAGTGGTTATTGTTATAATGAATTTTTTGGGAAAGTTCATTTTTGATTAATGTTTATCGGGGTTAATTTAACTTTTTTCCCTCAACATTTTTTAGGTTTAGCAGGATTTCCAAGACGATACTCGGATTATGCAGATGCTTTTTTGGGCTGAAATTTAATAAGCTCTTTAGGGTCTATTATTTCTATTTTGAGTGTTGTTTGGTTTTTATATATTGTTTTTGATCTTTTTGTTACAGAAGAAAAATTTTTGGGTTGAAAAGAAGGGTTTTCTTTAGAATGAATTCATTCTTCTCCCCCCTTATTTCATACTTATGAGGAGTTGCCTTTTGTACAAAAA-TAAATAATTTTTAGAATAGTGCCGGGTTTATGTACCGGTTT

calien TGTAAGAAAGACAAAGGGTTAGTCATTGGGTTCATGCCCCAAATAAGTGAGTTCGAATCTCTCTCTTACACAAAATAAAAATTATAAAAAAAAAAAGAAGATAACTAAGATAAATTAAAATTAGACGATTTTTTTCGAAATGGCGTTCAGTTGCTTATTGGCTGTTTCTCCAGAAAGGTGG---TTTTTTTAATTTGAATTGAAACATCTTATTACTAAGTAAAAATCAAACGAGATTCCGAGAGCGGCAGAGAGTGAAATTGGAGTTGTGCTTGTGGTGGTCATAGATCTTGCTTAAACATTAGTTTATACTGATAATGAGAGTACTGTAAGGGAAAGTTGAAAGAGAGTTGAAAGGAATTTGAATTTTTTATTTTTGAAGTAGCTTTAAAAAAGCGTACCTTTTGTATAATGGGTAAAAGAGATTTATTTGGCATATTTAAAATGGAAAATTTTAAGTAATT-TTTTTTTAGTTAAATTCCCCGAAACCAAGTGATTTAATCATGAATTGTGTGAGCAAAAGCTGGTGATTGTGGCAAAAATCTCAGAAAATTTGTGATTAGGGGTGAAAGGCTAATCGAACTTGGAAATAGCTGGTTTTCTGCGAAAACTATTTAAGTAGTGTTC-TTTTTGTTTTTATTTTAATTGTCATTTTAAAAAAAATAAAAATAAAAAAAAAGTAAACAAACAATAAATAAAAAGATTTTTTGTTAAAAGAGAAAGCTCAAATCCGAAGCTATTGTCA--TTTTTTTTCTTTAGTTTAAAAAAAAATTAAATTTAGACAATAAAAATGTAAGCTTGGAACCAGCCATCTTTTAAAAAGTACGTAGTTGTTTATTTAAAAATTTTAAATTTTTTTATAATTTTGATGGTTAAAATAAAACAGAAGCTTTGAAAATAATTATATATTTTGTAGCAGAGTAAATTGTTTT---T--------------------------TAGTTTGAAAAATTTTTT-TTAATACATCGTATTCAGTTGTAATAATTTTTACATAAGTAGTCTAAATATTATTTTTTTTTTCTTAAGACTACCTTGGTCAAGGGTAATACAGTTTCTAAAAAAGAAATTTTTTATGAAGATTTTTTATA-AAATATATAATATCGGGAAAAG-AAAAAAAAAGAACTGTTTTTCTAATTAAAGAAAGAAA-AAAAAAAGAAAAACATTTTTTTAAGGAACTCGGCAAAGCTAAACTTCGACTGTTTACCAAAAACATAGCTTTTTGATCTTTATAAAAGGTGAAACCTGCCCGATGGTTGTATCTTAATGTGTTTGTCTCGCCTGCTAATAAAGACAATTAAATGGCCGCGGTAACACTGACTGTGATAATGTAGCGTAATCAATTGTCAATTAATTGTTGACCGGTATGAATGGTGCCCCGAAAGTTTTTCTGTCTTAAAAAAATATTCAATGAAATTAAATTTGTAGTGAAGATGCTACATTTTAATTGYTAGACGAGAAGTCCCCATGGAGCTTTACTGTAAGCTTATATATATAA-ATTTTTTTTTTTATAAGCAAGACAGTTTTGTTGGGGCGACAGTTTTTTAAAAAGTAAYGAAAATGAACTATGACGCATGTTTAACTTTGAAATTTTTTTAATTGATGAGACATTTTTGGTGTGTTTTTTGATCCGTTATTTTGAATGAAAAAAATAACGAAAACAAATAAAAGTTACCCTGGGGATAACAGCGCAATAACGTTTGAGAGTTAATTAACGACAGTGTTTGCGACCTCGATGTTGAATTGTAACGTCCTACGGTGTAGTCACTCGTAAGGGTTGGTTTGTTCATCCATTAAAGTTATACATGATTTGAGTTAAAAGCGTGGTGACACAGCTTGGTTTCTATCTACAATTAGAAAACAAAAATATGTTGTTTTTTCGTACGAAAGGATCAAAAATCAATAGTTCCCTTAATATAACTATTCTTTAAATTAGGATTGCTTCTAAAAATAAAAAGAAATATTTTTGTTTGTTGTTTTTATTAATGTATCTTTTAGTTTTATTTTTTCCTTTAATTGGAGCTGTTTTAACAGGTTGTTTTGGAAGAAAAATTGGAGAAAGAGGAGCGGGGATTTTAACTTCAAGTTGTTTAGTTTTTAGTTTGTCTTATTCTTTTTTAATCGCGATTGAAGTTTTATTTAATTCAACAACAACGTATTTGAAATTATGAAAATGATTTGATTCGGGATTGTTTGTTGTTTTTTTTGGTTTTCAATTTGATGGTTTAGTAGTTATTATGTTATTTGTTGTTTTTATTGTTTCTACTTTGGTTCATATTTTTTCTATTGCTTATATGCGGGGGGATCCTCATGTTCCTCGATTTATGACATATCTCTCTTTGTTTACTTTTTTAATGGTTTTATTGGTAACTAGCGATAATTTTCTTCAATTATTTATTGGGTGAGAAGGGGTTGGCCTTTGTTCTTATTTATTAATTAATTTTTGATTAACTCGATTAGAGGCAAATAGAGCTGCTATTAAAGCAATGTTAGTGAATAGAATTGGTGATATTGGGTTGCTTTTAGCAATGTTTTTACTTTGGGATCTTTTTGGATCTCTAGATTTTTCTACAATTTTTAATTCTATTTTTTTTTCTAATCAAATATTTTTTATTTGTTTATTTTTATTTTTTGGGGTTATGGGAAAATCTGCTCAATTAGGATTACATACTTGGCTACCGGATGCAATGGAAGGTTATTGGGCCTTTTAATTAAAAAATTAATTAAAAAAATTACTATACATTAAAAAAACAATTTATTAGTAAAATAATTGTTTATTAATGGACAATAAAATATTTATTTTAATGTCTAAGAGACTTTATGTGATTTACTTTTTTTTATTTGATTAAATCTATTATTATTATTATTCCTTTACTTATTGCTGTTGCATTTTTAACTTTAGCAGAACGAAAAATTTTAGGGTATATGCAAATAAGAAAAGGGCCAAATGTTGTTGGAGGGGGGATCCTTCAACCTTTTGCAGATGGGGTGAAATTATTTATTAAAGAAATGATTCTTCCCCATCAAGCAAATAAATTTGTTTATCTTTTGGCTCCAGTTATATCTTTTACATTAGCTTTCTTTGTTTGAGGTTTTCTTCCTTATGAGAAAGGAGTGAGTATTAGTGATTTTAAAATTAGTCTTTTATGAATTTTGGCTATTTCTTCTATTAGTGTTTATGCAATTTTAATGTCTGGATGAGGAAGTAAGTCTAAATATGCTTTTTTGGGTGCTATTCGGGCGGCGGCTCAAATGATTAGTTATGAGGTTTCAATTGGGCTAATTCTTATTTCGGTTTTATTATGTGTTGGTTCTTTAAGTGTTACTGAAATTGTTTTAGCGCAAAATAATGGTATTTGGTTTTTTTTTCCACTATTTCCTGTTACAATAATGTTTTTTGCCTCGATTTTAGCAGAGACTAATCGTGCTCCTTTTGATTTAACAGAAGGAGAGTCGGAGCTTGTTTCGGGGTATAATGTTGAGTATGCCTCGATGTCTTTTGCTTTATTTTTTCTTGCTGAATATGCCCATATTATTTTAATGAGTTGTTTAACAACGATTTTTTTTTTGGGAGGATGGCTTTCTCCGATTTCAGATTTTAAGGGTGGGGCTGGATGGTTTGGTTTTAAAGTTGTTTTTATTATTTTTTTTTTTATTTGAGTAAGGGCTTCATTTCCTCGAATTCGATATGATCAGCTTATGGCTTTGTTATGAAAAGGGTATTTGCCTCTAAGTTTAGGAATGGTCATTTTTGTGGCTAGTGTTCTCTTTGGGTGTAATGGCCCCCCTCCTATTTAAAATATTTTAATTAATTA--------------------------------AAGTGCCATTACGAAAACAAAATCCTATTTTATTTTTAATCAACGGATTTTTAGTTGATTTGGTTTCTCCTTCTAACATTACTTATTTATGAAATTTTGGATCTTTATTGGGATTGTGTTTGATTTTACAGATAGTCACTGGGTGTTTTTTGTCTATGCATTATTGTTCTGATGTTAATTTTGCTTTTGCTTCAATTGGTCATATCATGCGAGATGTTAATTATGGGTTTTTATTAAGATATCTTCATGCTAACGGTGCTTCTTTGTTTTTTTTTTGTCTTTATGTTCATATTGGTCGAAGTTTATATTATGGGGGGTATTTAAAATTTCATGTTTGAAGCATTGGAGTTGTTATTTTTTTATTAACAATGGCTATTGCTTTTATGGGCTATGTTTTGCCTTGGGGACAAATGTCTTTTTGAGGGGCAACTGTAATAACTAATTTATTGTCTGCTATTCCTTATTTTGGGATTGATATTGTTCAGTGAGTGTGAGGCGGGTTTAGTGTTTCTAATGCAACATTAAATCGGTTTTTTAGTTTACATTTTTTACTTCCTTTTATTTTAGTTTTTCTTGTTATTCTCCATTTAGTTTATTTACATGTTGATGGGTCTAATAATTCAACAGGATTAAATTCTTCAATTGACGGAGTTTCATTTCATACTTTTTATACATCAAAAGATTTTTTTGGTTTTTTTTTTCTTTTTTTTTTATTTTGTTTTTTTGTTTTTTTTTTGCCAAATTTATTAGGAGATGCTGAAAACTTTATTCAAGCGAATTCTTTGGTTACTCCGGTTCATATTCAACCAGAATGATATTTTTTATTTGCTTATGCAATATTACGTTCTATACCAAATAAATTAGGTGGGGTTATTGCAATGTTTTGTAGTATTTTTATTTTATTTTTTTTATCTATTTTACATCAAAGTCTTTTAAAGGGGCTTTTTTTTCGTCCTTTAGGTCGGATTGCATTTTGGTTTTTAATTATTGATTTTGCTTTATTAACTTGAATTGGATCACAAGTTGTAGAAGAACCTTTTATTTTAATTGGTCAAATACTCTCTTTTTTTTATTTTTTTTATTTTTTAGTTTTAATACCAGTTTTGGGAATTATTGAAAATCAATTATTAAATAAAAATTAATGAAATTTTTGTTTTAGGGGGTTTTCTTCTGAGCTTCGTCGTTTTAAGTCTTCGTTATTTTCTTTTAAAACTTTCTCTTTTTTATTTATTATTATTTTTTTGTTTTTTTTTTTTAAAATTAGAATGGGGAAAGCTTTTGGTTTTAATTGGTTCTTTTGCTGTCTTTCTTTTATTTGGACCAAAAAAAGAAGAACAAACAGATGTGCCCATTTTAAGTTTAATTATTGTTTTTGGAGTTTTTTGTTTAATTTCTTCAAGTAATTGACTTTCTATTTATTTAACCATTGAACTTTTTACTCTTTGTTTTTTTATTTTGATTGCTCGAGGGTCTGGTTATAGTGTGGAAGCAGGATTAAAATATTTTATTTTGGGTGCTCTTTCTTCTGGTTTATTTTTATTTGGGTGTGCTTTATTATGTGGTATTGGGGCTAATATACACTTTTCTCATATAGAACTTCTTTTTAATTCAAAACAAGTTTTTTCTGCTGTTTCGATACCAATTGGGTATCTTTTAATTATTGTTGCTCTTTTTTTTAAATTATCAGTTGCTCCTTTTCATATGTGAGTTCCTGATGTTTATGAGGGGGCACCTACTAAAATTGTTCTATTATTGGCTATTGTTCCAAAAATAGGGTTTTTTTCTCTTATAATTTCAATTGGATTGCCTGTAAATTTTTTTTTTTTAGGGATTCTTTTTTCTTTGTTTGTTGGAGCTTTGGGTGCTTTAAATCAAACTAAAATAAAACGACTTTTGGCTTATAGTGGGGTTGGCCATATGGGCTTTATTTTATGGGGTTTGGAAAATGGTTCTTTTGAAAGTTTACAAGCTAGTCTTGTTTATCTTTTTATATATATTGTGATGACAATTTGTGCTTTTTCTATTATATTAAGTTTTAATGTTTATAAAAATTTACTTGTTGAATTTAGTGGACTTTCTCGATACTTACCTTTTTTTTCGATTACTTTAGGTGTTCTTTTTTTTTCTATTGCAGGAATTCCTCCTTTTGCTGGATTTTTTGGAAAATGATTTATTTTGTTATCTGGAATTCTTTCTAAATCATATTTTATTTTTTTTTTTGCTGTTTTTTGTTCTGTTATAGCTGGGGTTTATTATATTCGAATTATAAAAATACTTTTTTTTCAAAAAAATTCTTTTCTTTTAATTACAATTAAAGCTTTAAAAAAAGAATCTAAATTAAATTTTAAAAAAGTTTTTTTAATTGGTTTTTGTTTTTATTTTATTCTTTTTTTTTTTCTTTCTCCACATTTTCTTTTTTTTTTTTTTTCTCAAATAATTTTTGATTTATTTTAAAATGGAATTTTTTTCTTTTTTTTTTATTTTAGGGATAATTGGCTCAGGAGTAATGGTTGTTTCAGCGTTAAATCCTGTTCTTTCTATTTTTTGATTGGTTCTTGTTTTTATAAATTCTGCAGTTTTTTTCCTTTTACTAGGAATAGATTTTCTTGCTTTGATGTTTTTACTTATTTATGTTGGGGCAATAGCTATTTTATTTTTATTTGTTATTATGTTATTAAATCTAACTGACTATCCCCCGGTTTTAAAAAGAGAGGTTGATATGACAAATTATATACCAATTGGATTTATAATTGGGATTTTTTTTTTTTCAGAAATTGCTTCAAGTGGATTATTTTTGGGTTCTTTTCAAATAGAGAATTGAGATCTTTCTTTTCCTTGATTTCTTATTTCTTATCATAATATTGAGGCCTTAGGGCAGATTTTATATGTGTCTGGTTTTTGTTTGCTTCTCCTGGCCAGTTTTATTTTATTAGTTGCTATGATTGGTGTGATTGTATTAACTCAAGAAACAGAGTTTTTAAGTAAAAAACAAGATCTTTTTTTTCAAATAAACAGATAATGAATAGTTCTTCTTATTTTGAACAATTTAACATAGTGTGAATGTTTGGTTTTACGAATTCAACAATAATGATGACTTTTGTAATTATTGTTGTTTTATTACTTTTTAAAGGAATTGAATTAATTCCAAAAAGATGGCAATCGGTTTATGAATATCTAGAAAATTATTTTTATTATATAACAGTGCAAAATTTAGGAAATGTGGGTTTAATATATTTTTCTTTTATTGTTTCACTTTTTGTTTTTTTAGTTTTTTTAAATATTTTAGGGTTATGTCCTTACGTCTTTACACCGACAACGCATATCGTTGTTACTCTTGGGTTTTCTTTTTCTATTGTTATTGGAGTTACTTTTTCTGGATTTTATAAATTTAAAAAAGACTTCTTTAGCATTTTAATGCCTAGCGGCGCTCCTTTAATTTTAGCGCCACTTTTAATTTTAATAGAAACAGTAAGTTATATTTCTCGGGGGGTTTCTTTGGGGATTCGTTTAGCAGCAAACCTTTCTGCCGGGCATCTTTTATTTGCAATTTTGGCAGGATTTGGTTTAGTTTTTAAACTAGCAATGTCTATAATGGTGTTTATTACGCTATTGGAGATAGCTGTTGCGATAATACAGGCATATGTATTTTGTCTGCTCACATTAATTTATTTAGTTGACACAATTGTTTTACATTAAAAGAAAAAAAATTTGAGAAATGGGGGAATTAGAAATTATTTTTTATATAATATTTATGGTGTCATCAGGTTTAAATTGAGGGTTTGTTGTTATGTGGTATAATTCGTCCTCCCAAAGGTGGCTCCGGCATCAGATGGG-----TA----CTGATGGTTGGGTGCATCCTCATAACTATATAAGTTATGTGCGCCGTCATGGTATTGC---------TGAGAGACAAGT------------------TGGGCGAGA--------GGGGCCAACCGAGGCAA---T-------------GTATG---------TAGAGTCGGGGGGGC-----GGCCACGAAAT--TGGGGTGAAG-------------------AAGATAGTGAGT--CAGAAAGTATCGCGAATTTGA---G--------AGAGTG--TGT-AGA------------------------------------------------TAGGGCAATACGTCA-----GTTAGAAAGTAGAGAAAAT---TTGAGAGAGGGTGAAACTAGCGCA-TTAAGTGAGTCA------GAAAGTAGAGACAATTTGAGGGAGAGTGTAGCTAACGCATTAAGTGAGTCAGAAA---------G-TAGAGGCGA---------TTTAAGGGA--GAGTGTAACTAG---GGTATT-----ACGTGAGTCAAGAAGCAG-AGACG-A----------------AA-----CGCTC-CGGGGAGG----------AGA------GGCAGAAACAT---A----TGTATGTGATGGCAGGGT--------AAACAT--TGATGAGGCAGTTGAATCGACTCTTAGTTCGATCTCTTTTGAGGTTGGAGAGACGATGGTTCATGTGTGAAATCAATTTGACATGCTGTTTAACATATTGGCCTGTAGGCCCCTGGTAGGCTGCATTTTTTTATCTTACCAGCTTTTAAAAATAGCAAATCAAATGCCAAACCCACGGGTTTATAGGTGATTTGTTTTGGTATATATTTTATTAATCTTTGTTTTTTT-----TTTGCCT--------------------------------------TAG----TTATT------------------TTAGATTTA----------------TTTAACA--------------TGG----------------------TCTCTGGG--------------AAGCAGA-----------------------A-----------------------------------------TTTGGGT---GGGGCA-------------------------------------TTAGCCTTAAAG---G--------CTAG------------------------------AAAAGAGAGCAAAAA-A----TTTAATAGAGT----TGAGAAAAAAAGTTTTAAAATGGTTCTTTGTATTTAAATTAGCTAAAAATCAATAAAATTTTTATTTAAAGAA-TTTTTTTTGTGTTTGTTATTGTAATGGTTCTTATTTATTTAATTGTTATTATGGCATTAGTTAACATCATAGGGACAGCGAGGGAGAGAAGATGTGTTTTAAAAAAGCGGGCTTTAGAGTGGTCTTTGGCTTTATTGTTTAGTACTTTAGTTTTTTGGGGCGGATTTGACGGAGAAAGTCATTTTCAATTTTTTAGTTTAGTAGAATGAAATATATTTTCTACTTTAGATTGAGGCCCGATTGTTTTTGCAGTCGATGGTGTTTCTTTGGTTTTTTTACTTTTAACGACTTTTTTAATTCCAATTTGTATTTTAATTAGCCAAAAATCAATAAAATTTTTATTTAAAGAATTTCTTTTGTGCTTGTTTTTTTTAGAAGTTTTATTAATAGGTGTTTCTATAGTGTTTGATCTTCTTTTGTTTTATCTTTTTTTTGAGGGGATATTAATACCAATGTTTTTTTTAATTGGTATTTGAGGCTCTCGAGAAGAAAAGGTTCGTGCTTCTTTTTATTTTTTTTTTTTTACTTTTATAGGCTCTCTCTTTTT-TTTTTTATAATACTTTTTTTATACCAAAGGATTGGAACAACAGACTATTTTCTTTTACTTAATATTAAATTATTTTTAAATATTCAAAAATGAGCCTTAGTTGGGATTTTTCTTAATTTTGCGGTGAAACTACCTCTTATTCCATTTCATATTTGATTGCCACAAGCACATGTTGAGGCTCCTGTTGCGGGCTCTGTTATTTTGGCTGGAATTTTATTAAAATTAGGGGGCTATGGCCTTTTCCGTTTTTCTTGACCTCTTTTTCCGGGGGGTTCTTTATATTGGTCTCCAGTTATTGTTTTTTTTAGTGTTGTTGCTGTTGTTTATGGAGGCTTAATGACATGTCGTCAAATTGATTTTAAACGGCTTGTTGCTTACTCTTCTGTTGCTCATATGGGACTTGTGCCTTTGGGTCTTTTTACACATGTTATAGAGGGGTTAATTGGGGCTCTTTTTTTAATGTTGGCGCACGGATTTGTTAGCTCTGCTCTTTTTATTGGAGTAACTTTTTTGTATGATCGCCATCATACTCGTTTAATAAAATATTATCGGGGTTTGACTTTGACAATGCCTCTTTTTGTTATTACAATGTTAATTTTGTCTTTGGCGAACATGGGTTTTCCTCTTAGTTGTAATTTTGTTGGAGAATTTTTTTCTTTATTAGCAGTCTTTCAATATCATTACGGAGTTGGAATGTTTGTTATTTTAGGGGTTCTTTTTTCTGCAATTTATTCTCTTAGTCTTTTTAATCGTATTTCTTTTGGTGGAGGATCTAATTATTTACTTTTTAATAGAGACTTAAGTCGACGAGAGATTTCTGTAATTTTTCCTTTTCTTTTAATTATCTTGGGGGGGGGGATTGTGCCTTTTCCTATTATTGATTTAATTAAAAATAGTCTTGTTTTTAGTCCGGGGGGTTAGTCTGGGGGATTCGTCCTCCAGTCTTAGGGAAAAAAAAATATTTAGTTTAGGTTATACATGCTAGTGTGTGCGAATGGGTGAGGATAAAAATAAAAAAAAATTTTTT-TTTGTTGCATTAAAAAAAGAAAAGTTTTTTATTTTTTTTTTTGAAGATGCATGGTCTAACCACATTTTCCCTGAAACAAAAGAAAACCTTTGGCAGCAGTAACAAATTTTATGAAATATACGAAAGTTGGACATAGGTAAGAGAAAAAAAATCTGTCAAATAGAAGTGCCAGCAGACGCGGTAAGACTTAAGGGTTTATTTTTTTTATAAAAAGCAAAAAGCGTGTTAAGGATT----AAAAAAAAAAATAAATAGAATTTTTTTG--GTAATTGTGAAATGTTGGAATGAAAAAAAGAATTTTTTATATGAAAATAATTTATTTTTTTTTTTTTTTTCTTGAAGACGAAGGTTTTGAGAGCAAACAGGATTAGGGACCCTGGTAGTCTATACAGTAAAAGAATATCGCTTGAGTAGTACGGTCGCAAGATTAAAATTCAAGGGACTTGGCTGTTCGGTTGTTAATTAGAGGAGCGCGCCGCTTAATTCGATAATCCGCGAGAGACCTTACCGAAGTTTGAATTTATTAACAGGTGTTGCATGGCCGTCGTCAATTTCTGTTTAAGACAAAGAGGGGTTTAATCCTATCAAGGTTGAAGTCAGGTATTATGACCCTTATATTTCGGGGTTAGGCGCGCTACATTTCTCTCTTAGGAAAGGAGGGGTTCGGATTGTCTTTAAAATAAGATAATGAAGTTGGATTTAATAGTAATTGAAAAGTAGTGCGTTTCAATGAACGTGAAAACAATGTTAGTACACATAGCCCGTCGCCAGCCCAGAAATAGGCTGTAAGTCGTAACATAGTAAGAGTGAGGGAACTGGCTCTTGATGCAGTTTCATTCGTATCATTTAGTTGAGCCTTCTCCATGGCCTTTTGTTGGAGCAATTGGCTCTTTTTTTATTACTGTTGGTGCAGTGGTTTTTTTTCATTATGGGTTTAGTTTTTTTTTATATTTGGGGCTTTTGGTTGTTGTTGGAGTTATGTTTGTTTGATGACAAGATGTTATACGAGAATCAACTTTTCAAGGTCACCATTCTTTAATTGTAAAACAAGGGATTAAATATGGTATGATTTTATTTATTCTTTCAGAAATTTTGTTTTTTTTTTCTTTTTTTTGGGCTTTTTTTCATAGTAGTTTAGCACCGGTTGTTGAACTTGGAGTCGTTTGGCCTCCACAAGGAATTGTTGCATTAAATCCCTTTTCTGTTCCTTTATTAAATACTGCTGTATTATTAAGTTCCGGGGCAACGGTTACATGGACGCATCATGCAATACTTTGTGGTTTAAAAAAAGAGGCTCAATTTGCTTTATTTTTAACTCTTTTTTTGGGTGTTATGTTTACGGGGTTGCAAGCATTTGAATATTATGAGGCGCCTTTTACTTTATCTGACTCTGTTTATGGGGCCACTTTTTTTGTTGCGACAGGGTTCCATGGACTACATGTTATAATTGGTACTACTTTTCTTTTTATTTGTTTTTTACGTTTACTTTCTAATCAATTTACTCGTCGTCAACATGTTGGGTTTGAAGCCGCGAGTTGATATTGGCACTTTGTCGATGTGGTTTGATTATTTTTATATTTATGTATTTATTGATGAGGCTCATAAAAACTTCTTTAATGCAAAATCTTTTTGTTTT-------------T-AT----AAATGTTTTGGCTTGGTTATGTGTTCTCTGGTTTAATCATCTTTATTATTCGGATAGTCCAGAGACTTGACTTTTAGAGTTTCAAGATGTTGGGGATCCTATTGTAGAAGAAATTGTTTTTTTTCATGACCAAGTTATGTTTTTATTAATTATTATAGTTACTGTTGTTTTATGACTTTTTGTTGAAGCTTTTAAAAATAAGTTTTATGATCGTCATTTAATCGATGGTACATTTTTAGAAATTGTTTGAACGATTATTCCTGCTGTTATATTGATTTTTATTGCATTGCCCTCTCTTAAATTATTATATTTAATGGATGAGGTTATTYCTCCAGCTTTGACAATTAAAGTTATTGGGCATCAATGATATTGATCCTATGAATATTCTGATTATGAAGGAGATACGCTAGAGTTTGATTCTTATATGATTCCTACTTCGGATTTAGTTTCTGGAGAAAATCGTTTGTTAGAARTTGATTATAAACTTTTAATTCCTATTCAAACACATACAAGATTTTTAGTTACTGGGGCAGATGTTTTACATTCTTYTGCAGTTCCTTCTTTAGGGTTAAAAATCGATGCGGTTCCGGGTCGTCTAAATCAAACTGGTGTTTTTATAAAACGAGCCGGGGTTTTTTTTGGACAATGTTCTGAAATTTGTGGGGCAAATCATTCTTTTATGCCAATTGTGATAAAGGGAGTTGGTTTAAATGAATACGTTCAATATTTAAATTATTTAAAATGTATTATAAATACTTAGTTATTATTATTATTTTATTTTTATTGGGGAGTTGGGGTATAATTTTAAATAGAGGACATTTTATTATTATGCTTGTTTCTATTGAATTAGTTTTATTATCAACTTTTTTTTTTTTTTTAATAAGTTCTAAAGAAATAGATCTTTTAATAGAACAAATTTTTATGATTATGGGATTAACTATTGCTGCGGCAGAATCTTCAATTGGTTTAGCTATTTTAGTTGCATATTATAGAATTCGAGGAACAATTGTTTTAAAATCTTTTAGTTCTTTGCGAGGATAATGATTAATCTTTTTATTTTTTTTTTTCTTATTTTTATTTTGGCGGGGTTATTTATATTTCTTTCTTTTTTGATAGGGGAAAAAACTCCAGATCGAGAAAAGGTTTCTGCTTATGAATGTGGTTTTGCCCCCTTTAATTTTTTAGGGCGTCCTTTTTCAATACGTTTTTTTTTAATTGGTATTTTATTCTTGATTTTTGATTTAGAAATCTCTTTTTTTTTCCCCTGATGTGTTTTATATAATTCGACTGCTCCGTTTGGGTTTTGAACTATGATAGGTTTTTTTTTTGTATTAGTTTTGGGTTTGATATACGAATGAGTAATGGGGGGATTGGAATGAGAGTAAAAAGAGAGTAAAAGAAAAAGTCCTATCTATTATGAAAATAATAGAAATTTTATACCAACTCCGGTTTCTGCTTTAATTCATGCTGCAACGATGGTTACTGCAGGTGTTTTTTTATTAATTCGTGCTTCTCCTTTATTTGATGTTGTTCCTCTTATATTAATTGTTATTTCAATCGTTGGGGTTTTAACCGTGTTTATTGCAGGCACAATTGGTTTAGTTCAAAATGATTTAAAAAAAATAATCGCTTATTCTACTTGTAGTCAATTAGGCTATATGGTTGTTGCTTGTGGGCTTTCTCATTATGCAATTAGTCTTTTTCATCTTATGAATCATGCTTTTTTCAAAGCTTTATTATTTTTGAGTGCTGGGTCTCTTATTCATGCGGTAGTTGATGAACAAGACATAAGAAAAATGGGGGGTTTATTATCTTTTCTTCCTTTGACTTATGTTTTTTTTTTGATAGGCTCTTTTTCTTTAATGGGGTTTCCTTTTTTAACAGGATTTTATTCAAAAGATTTAATTTTAGAATTTGCTTTTGGACAATTTTATTTAATTTTTGTGTATTGATTAGGTTGTTTTTCTGTTTTATTAACAATTACATACTCTATTCGTTTAATTTATTTAGTTTTTTTATCCAATATTAATTTAAAACGAGCAAACATTTTTTTTCTTAAAGAAGGAGAATTTTTATTTTTAATTCCTTTGGGTATATTAACTTTAGGAAGTGTTTTTTGAGGCTATTTAAGTAAAGAAATAATTTGGTCTTTTCAAATAGATGTTTTTTCAATACTTTCTTTAAAAATAAAAATATTTCCAATTTTATTTTGTTTTATTGGACTTTTTGGAACGATATTTTTTTTTTTTTTTTTTTCTTCTCAAATTTTTGGTTATCCCCTTCAGGTGGTCAGATCTTCTGTTTTTTTTCTTTATAATTTTTTTGGTTCTGCTTGACAAATAAATTTTTTTTTTAATTTTTTCTTTATAAAAAAAATATATAAAATAGGACATCTAATTACTAATTTAACAATTGATAAAGGTTTATTAGAGGTTGTCGGGCCCAGGGGTGTTGTTCAATTTTTTATTTTTCAAACTCAAAAGTTGAGTAGTTTACAATCGGGGTTAGTATTTAATTATGCTTTAGTTTTTTTTCTTGGGATATTTTTTTTAATTTTTGTACTTTAATTAAAATTTTTGAAAGAGTTGAGTTAAAGTAAACTGTTGATCTTCAAAATCAACGATGTAGGTGCAAGTCCCACACTCTTTGAGTGCCTCAGTTAAAAGTAAGCTTTTATAAGATACAATATTGATGGGGTTTTTCGGTTTTATTTTTATTGTTAATTTTTTTTGAGATTGTTGTTTTTCCTTTAATAAAACGTAATTGATGGATAAGAAAGTTCTTAATGAAGTGCGATGGCGCCATCTTAACAAAAATTT--GGTTACAAAAAGAAATTTATAAAAAAA----AAAAAAAAGTATGGTGTAATATT---AAAATGTTTGTACTTTAACTTAATTTTTAATTGGTTTAG-CTATTTTAGTTAGATATTATAAAATGCAAAAAACAATTGTTTTAAAATCTTTTGATATTAAAAATTTCTGGAAGAGTTAAGTTAAAG-----TACTTTAATTAAAATTTTTGAAAGAGTTAAG-TTAAAGTAAACTGTTGATCTTCAAAATCAACGATGTAG---------------------GTGCAAGTCCCACACTCTTTGAG--TGCCTCAAAAGTAT---TTAGCT--TTTGCA--AAAAATAGTATGTCTTGAGTTGATATGT--TCA-AAATTATGTGGTTGGGTAAAAGTTTA--------G--TTATAAGAGTTGCAATACTTTTTATTTTG----------------------GATTTAAAGTGAGTTATTTTATA-------------------ATAAGTGTTTAAAAAAAAATTTGTTTGTT--CATGCTGAGGGTCCGC-CCCG--TAAGCGTGCTAGAGTTGAAGAAGA--ACCATTGTGAAATATG----------------------------CCGGAGAAG--ATTGAAAATG---------AAGTAGA-----AGGGGGAGCCGAAAATGA--AGATTTGGGTTATCTAGCA-----TTA----------AATTTACTTGAAT--TTGAAT--ACTTTACAGAAGAGCAAGAAGACGAATTAGTGGCGGTGTTAATTGAGTGAGCAAACAGTGTG---------------------G-CGGCCGGAGAGCCAAACGAGGGGTTATAAAATGTGTTAAT--AGG--G---TGGGCAGAAAGCG-AAGTGTCTGAAGATTCAAACGACGAATCAGAGGAGAACCCCGGGGAATAAGTCGG-CAGAGTA--GGACCCCGGGGAGTAAGTAG--AGGCAGGGTCATGGTCTCCTAG---CCCCAAGTCTTGAGATAGAGGGTTCT-AAGCCATAGAATTCG-ACATAAGGGGCGTTAGGGGCAAGCCCAT--GGTGTCAAATCTTGTT------TCGGCCGCGGTGTACTAGCCCCAAATCTTGAGA---------TAAATGGTTTCTAGTCATTGAATTCGATGGGTTTTTTCAACCAATCATAAAGATATCGGTAGTTTGTATCTAATTTTTGGTGGGGGTGCTGGTTTAATCGGGACGGCRTTTAGTATGCTTATACGACTCGAGCTTTCTGCGCCCGGAGCGATGTTAGGAGATGATCATCTTTATAATGTAATTGTTACAGCACATGCTTTTATTATGATTTTTTTTTTGGTTATGCCRGTTATGATTGGGGGGYTTGGTAATTGATTGGTCCCATTATATATTGGGGCGCCGGATATGGCGTTTCCCCGACTAAACAATATTAGTTTTTGACTYTTGCCCCCTGCGCTTTTTTTATTATTAGGCTCTGCTTTTATTGAACAAGGGGCGGGGACGGGGTGAACAGTTTATCCTCCTCTTGCTAGTATTCAAGCACACTCCGGAGGTTCGGTTGATATGGTTATTTTTAGTCTTCATTTAGCTGGGGTTTCTTCTATTTTAGGTGCTATAAACTTTATTACTACAATTTTAAATATGCGAGCCCCGGGTGTGTCTTTTAATAAACTACCTTTATTTGTTTGATCTATTTTAATAACAGCTTTTTTATTGCTTTTATCTTTACCTGTTTTAGCTGGTGCTATTACTATGTTGTTAACAGATAGAAACTTTAATACGACTTTTTTCGATCCAGCGGGTGGCGGGGACCCAATATTATTTCAGCATCTATTTTGATTCTTTGGGCATCCAGAAGTTTATATTTTAATTTTGCCTGGTTTTGGTATGATTTCTCAAATAATCCCGACTTTTGTTGCTAAAAAACAAGTTTTCGGGTATTTAGGAATGGTTTATGCCATGCTTTCTATTGGGCTTCTGGGATTTATTGTTTGAGCTCATCATATGTTTACTGTTGGGATGGATGTAGATACAAGAGCATATTTTACTGCTGCTACTATGRTTATTGCTGTGCCAACTGGGATTAAAGTTTTTAGTTGGTTGGCAACTATTTATGGAGGTGTTCTTAGGTTAGAGACTCCAATGCTTTGAGCTATGGGGTTTGTTTTTTTATTTACAGTTGGTGGTTTAACTGGGGTTGTATTAGCAAATAGTTCTCTTGATATTGTTCTACATGATACATATTATGTAGTTGCGCATTTTCATTATGTTCTTTCTATGGGGRCTGTTTTYGCTATTTTTGGGGGATTTTACTATTGAATTGGAAAAATAAGTGGTTATTGTTATAATGAATTTTTTGGGAAAGTTCATTTTTGATTAATGTTTATCGGGGTTAATTTAACTTTTTTCCCTCAACATTTTTTAGGTTTAGCAGGATTTCCAAGACGATACTCGGATTATGCAGATGCTTTTTTGGGCTGAAATTTAATAAGCTCTTTAGGGTCTATTATTTCTATTTTGAGTGTTGTTTGGTTTTTATATATTGTTTTTGATCTTTTTGTTACAGAAGAAAAATTTTTGGGTTGAAAAGAAGGGTTTTCTTTAGAATGAATTCATTCTTCTCCCCCCTTATTTCATACTTATGAGGAGTTGCCTTTTGTACAAAAA-TAAATAATTTTTAGAATAGTGCCGGGTTTATGTACCGGTTT

J295 ?????????????????????????????????????????????????????????????????????????????????????-???????????????????????????????????????????????????????????????????CTTATTGGTTGTTTCTTCAGAAAAGTGA--TTTTTTTTTATTTGAATTGAAACATCTTATTACTAAGTAAAAATCAAACGAGATTCCGAGAGTAGTGGAGAGTGAAATTGGAGTTGTGCTTGTGGTGGTCATA????????????????????????????????????????????????????????????????????????????????????????????????????????????????????????????????????????????????????????????????????????????????-?????????????????????????????????????????????????????????????????????????????????????????????????????????????????????????????????????????????????????????????????????-??????????????????????????????????????????????????????????????????????????????????????????????????????????????????????-??????????????????????????????????????????????????????????????????????????????????????????????????????????????????????????????????????????????????????????????????TATTTCGTAGCAGAATAAATTGTTTTCG????-?------------------?????????????????????????????????????TCAGTTGTAATAATTTTTACATAAGT?????????????-?????????????????????TTAGTCAAGGGTAATACAGTTTCTAAAAA??????TTTTTATGAAGAT??????????????????????????????????????????????????????????????????????????????????????????????????????????????????????????????????????????????????????????????????????????????????????????????????????????????????????????????????????????????????????????????????????????????????????????????????????????????????????????????????????????????????????????????????????????????????????????????????????????????????????????????????????????????????????????????????????????????????????????????????????????????????????????????????????-?????????????????????????????GATCCGTTATTTTGAATGAAAAAAATAACGAAAACAAATAAAAGTTACCCTGGGGATAACAGCGCAATAATGTTTGAGAGTTAATCAACGACAGTGTTTGCGACCTC????????????????????????GTGTAGTCACTCGTAAGGGTTGGTTTGTTCATCCATTAAAGTTATACATGATTTGAGTTAAAAGCGTGGTGACACAGCTTGGTTTCTATCTACAATTAGAAAACAAAAATATGTTGTTTTTTCGTACGAAAGGATCAAAAATCAATAGTTCTCTTAATATAACTATTCTTTAAATTAGGATTGTTTCTAAAAATAAAAAGAAATATTTTTGTTTGTTGTTTTTATTAATGTATCTTTTAGTTTTATTTTTTCCTTTAATTGGAGCTGTTTTAACAGGTTGTTTTGGAAGAAAAATC?????AAGAGGAGCG??????????????????????????????????????????????????????????????????????????????????????????????????ATGAAAATGATTTGATTCGGGGTTGTTTGTTGTTTTTTTTGGTTTTCAATTTGATGGTTTAGTAGTTATTATGTTATTTGTTGTTTTTATTGTTTCTACTTTGGTTCATATTTTTTCTATTGCTTATATG??????GATCCTCATATTCCTCGATTTATGACATATCTTTCTTTGTTTACTTTTTTAATGGTTTTATTGGTAACTAGCGATAATTTTCTTCAATTATTTATTGGGTGAGAAGGGGTTGGTCTTTGTTCTTATTTATTAATTAATTTTTGATTAACTCGATTAGAGGCAAATAGAGCTGCTATTAAAGCAATGTTAGTGAATAGAATTGGTGATATTGGGTTGCTTTTAGCAATGTTTTTACTTTGGGATM?????????????????????????????????????TATTTTTTTTTCTAATCAAATATTTTTTATTTGTTTATTTTTATTTTTTGGGGTTATGGGAAAATCTGCTCAATTAGGATTACAT????????????????????????????????????????????????????????????????????????????????????????????????????????????????TRGACAATAAAATATTTATTTTAATGTCTAAGAGACTTTATGTGATTTACTTTTTTTTATTTGATTAAATCTATTATTATTAT??????????????????????????????????????????????????????????????????????????????????????????????????????????????????????????????????????????????????????????????????????????????????????????????????????????????????????????????????????????????????????????????????????????????????????????????????????????????????????????????????????????????????????????????????????????????????????????????????????????????????????????????????????????????????????????????????????????????????????????????????????????????????????????????????????????????????????????????????????????????????????????????????????????????????????????????????????????????????????????????????????????????????????????????????????????????????????????????????????????????????????????????????????????????????????????????????????????????????????????????????????????????????????????GATCAGCTTATGGCTTTGTTATGAAAAGGGTATTTGCCTTTAAGTTTAGGAATGGTCATCTTTGTGGCTAGTGTT????????????????????????????????????????????????????ATGAAAAAAAAAAAGAACTAACAGATTTGCAAGTGCCATTACGAAAAAAAAATCCTATTTTATTTTTAATCAACGGATTTTTAGTTGATTTGGTTTCTCCTTCTAACATTACTTATTTATGAAATTTTGGATC???????????????????????????????????????????????????????????????????????????????????????????????????????????????????????????????????????????????????????????????????????????????????????????????????????????????????????????????????????????????????????????????????????????????????????????????????????????????????????????????????????????????????????????????????????????????????????????????????????????????????????????????????????????????????????????????????????????????????????????????????????????????????????????????????????AACAGGATTAAATTCTTCAATTGACGGAGTTTCATTCCATACTTTTTATACATCAAAAG????????????????????????TTTTTTTATTTTGTTTTTTT???????????????????????????????????????????????????????????????????????????????????????????????????????????????????????????????????????????????????????????????????????????????????????TTATCTATTTTACATCAAAGTCTTTTAAAGGGACTTTTTTTTCGTCCTTTAGGTCGAATTGCCTTTTGGTTTTTAATTATTGATTTTGCTTTATTAACTTGAATT???????????????????????????????????????????????????????????????????????????????????????????????????????AAAATCAATTATTAAATAAAAATTA???????????????????????????????????????????????????????????????????????????????????????????????????????????????TTTTTAAATTAGAATGGGGAAAGCTTTAAA???????????????????????????????????????????????????????????????????TATTTTAAGTTTAATTATTATTTTTGGAGTTTTTTGTTTAATTTCTTCAAGTAATTGACTTTCTATTTATTTAACCATTGAGCTTTTTACTCTTTG??????????????????????????????????????????????????????????????????????????????????????????????????????????????????????????????????????????????????????????????????????????????????????????????????????????????????????????????????????????????????????????????????????????????????????????????????????????????????????????????????????????????????????????????????????????????????????????????????????????????????????????????????????????????????????????????????????????????????????????????????????????????????????????????????????????????????????????????????????????????????????????????????????????????????????????????????????????????????????????????????AMCTTTTTTWWMRTTTACTTTAGGTGTTCTTTTTTTTTCTATTGCAGGAATTCCTCCTTTTGCTGGCTTTTTTGGAAAATGATTTATTTTGTTATCTGGAATTCTTTCTAAATCATATTTTATCTTTTTTTTTGCTGTTTTTTGTTCTGTTATAGCTGGGGTTTATTATATTCGAATTATAAAAATACTTTTTTTTCAAAAAAATTCTTTTCTTTTAATTACAATTAAAGTTTTAAAAAAAGAATCTAAATTAAATTTTAAAAAAGTTTTTTTAATTGGTTTTTGTTTTTATTTTATTCTTTTTTTTTTTCTTTCTCCACATTTTCTTTTTTTTTTTTTTTCTCAAATAATTTTTGATTTATTTTAAAATGGAATTTTTTTCTTTTTTTTTTATAATTSGRATAAT??????AGSWRTA?????????????????????????????????????????????????????????????????????????????????????????????????????????????????????????????????????????????????????????????????????????????????????????????????????????????????????????????TTATATACCAATTGGATTTATA??????????????????????????????????????????????????????????????AGAGAATTGAGACCTTTCTTTTCCTTGATTTCTTATTTCTTATCATAATATTGAGGCCTTAGGGCAGATTTTATATGTTTCTTGTTTTTGTTTATTTCTTTTGGCCAGTTTTATTTTATTAGTTGCTATGATGGGTGTGATTGTATTAACTCAAGAAACAGAATCTTTAAGTAAAAAACAAGATCTTTTTTTCCAAATAAATAGGTAATGAATAGTTCTTCTTATTTTGAACAATTTAACATAGTGTGATTGTTTGGTTTTACGAATTCAACAATAATGATGACTTTTGTAATTATTGTAGTTTTATTATTTTTTAAAGGAATTGAATTAATTCCAAAAAGATGGCAGTCGGTTTATGAATGTTTAGAAAATTATTTTTATTATATAACAGTGCAAAATTTAAGTAATGTGGGTTTAATATATTTTTCTTTTATTGTTTCCCTTTTTGTTTYTTTAGTTTTT?????????????????????????????????????????????????????????????????????????????????????????????????TTTCTGGATTTTATAAATTTAAAAAAGATTTTTTT?????????????????????????????????????????????????????????????????????????????????????????????????????????????????????????????????????????????????????????????????????????????????????????????????????????????????????????????????????????????????????????????????????????????????????????????????????????????????????????????????????????????????????????????????????????????????????????????????????????????????????????????????????????????????????????????????????????????????????????????????????????????????????????????????????????????????????????????????????????????????????????????????????????????????????????????????????????????????????????--????????---------------------------------------?????????????????---???????????????--???-???---------------------------------------------?????????????GATCA-----ATTTGA-AGATGTGGCTTT---TTTAG-GGGGGAG-TACAAGTACAATTACACCAGAAA----GTGTAAATAGGGGCGCTCCAAGTGGTAATATAAGAAATGTATTAAGTGATTCATTAA-TAGA-G-??-???????????????????????????--??-???????????????????----????????????????????GATG-A-------G--CCAGT-AT-----AAATCAAGGTGAGGCTTTAAG---AGAAAACAAGGTAGAAATTGGGG----TTTTAAGAGAAAACAAAGT--------AGAAAT--TGATGAGGTAGTTGGCTCGACTTTTAGTTCGATA???????????????????????????????????????????????????????????????????????????????????????????????????????????????????????AACG??????????????????????????????????????????????????????????????????????????????????????????????????????????????????????????????????????????????????????????????????????????????????????????????????????????????????????????????????????????????????????????????????????????????????????????????????????????????????????????????????????????????????????????????????????????????????????????????????????????????????????????????????????????????????????????????TCTTTAGGAGTTTAGAATGAGAGAAAAAGTTTTAA?????????????????????????????????????????????????????????????????????????????????????????????????????????????????????????????????????????????????????????????????????????????????????????????????????????????????????????????????????GAAAGTCATTTTCAATTTTTTAGTTTAGTAGAATGAAATATATTTTCTACTTTAGATTGA???????TTGTTTTTGCAGTTGATGGTGTTTCTTTGGTTTTTTTACTTTTAACGACTTTTTTAAWWCMMATTTK??????????????AAAAATCAATAAAATTCTTATTTAAAGAATTTCTTTTGTG???????????????????TTATTAATAGGTGTTTT?????????????????????????????????????????????ATTAATACCAATGTTTTT????????????????????????????????????????????????????????????????????????????????????????????????????????????????????????????????????CTATT???????????????????????????????????????????????????????????????????????????????????????????????????????????????????????????????????????????????????????????????????????????????????????????????????????????????????????????????????????????????????????????????????????????????????????????????????????????????????????????????????????????????????????????????????????????????????????????????????????????????????????????????????????????????????????????????????????????????????????????????????????????????????????????????????????????????????????????????????????????????????????????????????????????????????????????????????????????????????????????????????????????????????????TATTTTTTTCTGCCATTTATTCTCTTAGTCTTTTTAATCGTATTTCTTTTGGTGGAGGCTCTAATTATTTACTTTTTAATAGAGACTTAAGTCGACGAGAGATTTT???????????????????????????????????????????-------------------------??????????????????????????????????????????????????????????????????????????????????????????????????????????????????????????-?????????????????????????????????????????????????????????????????????????????????????????????????????????????????????????????????????????????????????????????????????????????????????????????????????????????????????????????????????????????????????????????????????????????????????????????????????????????????????????????????????????????????????------?????????????????????????????????????????????????????????????????????????????????????????????????????????????????????????????????????????????????????????????????????????????????????????????????????????????????????????????????????????????????????????????????????????????????????????????????????????????????????????????????????????????????????????????????????????????????????????????????????????????????????????????????????????????????????????????????????????????????????????????????????????????????????????????????????????????????????????????????????????????????????????????????????????????????????????????????????????????????????????????????????????????????????????????????????????????????????????????GACCATTCTTTAATTGTAAAACAAGGGATTAAATATGGTATGATTTTATT?????????????????????????????????TTTTTGGGCTTTTTTTCATAGTAGTTTAGC????????????????????????????????????????????????????????????????????????????????????????????????????????????????????????????????????????????????????????????????????????????????????????????????????????????????????????????????????????????????????????????????????????????????????????????????????????????????????????????????????????????????????????????????????????????????????????????????????????????????????????????????????????????????????????????????????????????????????????????????????????????????????????????????????????????????????????????????????????????????????????????????????????????????????????????????????GATCCTATTGTAGAAGAAATTGTTTTTTTTCATGACCAAGTTATGTTTTTATTAATTATTATAGTTACTGTTGTTTTATGACTTTTTGTTGAAGCTTTTAAAAATAAGTTTTATGATCGTCATTTAATCGATGGTACATTTTTAGAAATTGTTTGAACGATTATTCCTGCTGTTATATTGATTTTTATTGCATTGCCCTCTCTTAAATTATTATATTTAATGGATGAGGTTATTTCTCCAGCTTTGACAATTAAAGTTATTGGGCATCAATGATATTGATC??????????????????????????????????????????????????????????????????????????????????????????????????????????????????????????????????????????????????????????????????????????????????????????????????????????????????????????????????????????????????????????????????????????????????????????????????????????????????????????????????????????????????????????????????????????????????????????????TA??????????????????????????????????????????????????????????????????????????????????????????????????????????????????????????????????????????GATCTTTTAATAGAACAAATTTTTATGATTATGGGGTTAACTATTGCTGCTGCCGAATCTTCAATTGGTTTAGCTATTTTAGTTGCATATTATAGAATTCGAGGAACAATTGTTTTAAAATCTTTTAGTTCTTTGCGAGGATAATGATTAATCTTTTTATTTTTTTTTTTCTTATTTTTATTTTGGCGGGGTTATTTATATTTCTTTCTTTTTTGATAGGGGAAAAAATTCCAGATC???????????????????????????????????????????????ATAGATAGGACTTTTTCAATACGTTTTTTTTTAATTGGTATTTTATTCTTGATTTTTGATTTAGAAATTTCTTTTTTTTTTCCCTGATGTGTTTTATATAATTCGACTGCTCCGT??????????????????????TTTTTTTTTTGTATTAGTTTTGGGTTTGATATATGAATGAGTAATGGGGGGATTAGAATGAGAGTAAAAATAAAGTAAAAGAAAAAGTCCTATCTATTATGAAAATAATAGAAATTTTATACCAA??????????????????????????????????????????????????????????????????????????TTATTT????????????????????????????????????GTTGGGGTTTTAACCGTGTTTATTGCAGGCACAATTGGTTTAGTTCAAAATGATTTA????????????????????????????????????????????????????????????????????????????????????????????????????????????????????????????????????????????????????????????????????????????????????????????????????????????????????????????????????????????????????????????????????????????????????????????????????????WWWWKCTTTTGGACAATTTTATTTAATTTTTGTGTATTGATTAGGTTGTTTTTCTGTTTTATTGACAATTATATACTCTATTCGTTTAATTTATTTAGTTTTTTTATCCAATATTAATTTAAAACGAGCAAACATTTTTTT???????????????????????????A????????????????????????????????????????????????????????????????????????????????????????????????????????????????????????????????????????????????????????????TTTTTTTTTTTTTTTTTTTCTTCTCAAATTTTTGGTTATCCCCTTCAGGTGATC??????????????????????????????????????????????????????????????????????????????????????????????????????????????????????????????????????????????????????????????????????????????????????????????????????????????????????????????????????????????????????????????????????????????????????????????????????????????????????????????????????????????????????????????????????????????????????????????????????????????????????????????????????????????????????????????????????????????????????????????????????????????????????---------------?????????????????????????????????????????????????????????????????????????????????-????????--??????????????????????????-????????????????????????????????????????????????????????----------------------------------------------------------------------------------------------------------------------??????????????????????---??????????????---??????????????--?????WRRAARTRGAW???-?????TGA--AGAGATAGTAGGAG--CGCTTCCATCTTTA--------AGCTTCGTGGAGGGGTGAGATG----------------------------AAGAGGTGATAGCAGCGTTTCCATGTTCAGACT----------CCGAAGAGAGTATGGAAATTGATGGGGAAGA--ACTACCTTTGGATC????????????????????--????????????--????????????-------------------------------????????????????????????????????????????????????????????????????????????????-????????????????????????????????????--??---?--??????????????---??????????????????????????????????????-??????---------------------??????????????????????????????????????????--???--??????????????????????????-?????????????-???-?????-??????????????????????????????--???????????????????????????????-??????????????????????????????????????????????????????????????????????????????????????--????????????????????????????????????????-?????????????????????????????????????????????????????????????????????????????????????????????????????????????????????????????????????????????????????????????????????????????????????????GATCATCTTTATAATGTAATTGTTACAGCACATGCTTTTATTATGATTTTTTTTTTGGTTATGCCGGTCATGATTGGGGGATTTGGTAATTGATTAGTCCCATTATATATTGGG????????????????????????????????????????????????????????????????????????????????????????????????????????????????????????????????????????????????????????????????????????????????????????????????????????TTCTTCTATTTTAGGTGCTATTAACTTTATTACTACAATTTTTAATATGCGAGCCCCGGGTGTGTCTTTTAATAAACTACC???????????????TATTTTAATAACAGCTTTTTTATTACTTTTATCTTTACCTGTTTTAGCTGGTGCTATTACTATGTTGTTAACAGATAGAAACTTTAATACGACTTTTTTCGATC??????????????????????????????????????????????????????????????????????????????????????????????????????????????????????????????????????????????????????????????????????????????????????????????????????????????????????????????????????????????????????????????????????????????????????????????????????????????????????????????????????????????????????????????????????????????????????????????????????????????????????????????????????????????????????????????????????????????????????????????????????????????????????????????????????????????????????????????????????????????????????????????????????????????????????????????????????????????????????????????????????????????????????????????????????????????????????????????????????????????????????????????????????????????????????????????????GAAGAAAAATTTTTGGGTTGAAAAGAAGGATTTTCTTTAGAATG????????????????????????????????????????????????????????????????????????????????????????????????????

S3 TGTAAGAAAGACAAAGGGTTAGTCATTGGGTTCATGCCCCAACCAAGTGAGTTCGAATCTCTCTCTTACACAAAATAAAAATTAT-AAAAAAAAAAGAGGATAACTAAGATAAACTAAAATTAGACGATTTTTTTCGAAATGGCGTTCAGTTGCTTATTGGTTGTTTCTTCAGAAAAGTGA--TTTTTTTTTATTTGAATTGAAACATCTTATTACTAAGTAAAAATCAAACGAGATTCCGAGAGTAGTGGAGAGTGAAATTGGAGTTGTGCTTGTGGTGGTCATAGATCTTGCTTAAACATTAGTTTATACTGATAATGAAAGTACTGTAAAGGAAAGTTGAAAGAGAGTTGAAAGGAATTTGAATCTTTTATTTTTGAAGTAGCTTTAAAAAAGCGTACCTTTTGTATAATGGGTAAAAGAGATTTATTTGGCATATTTAAAATGGAAAATTTTAAGTAA-TTTTTTTTTAGTCAAATTTCCCGAAACCAAGTGATTTAATCATGAATTGTGTGAGCAAAAACTGGTGATTGTGGCAAAAATCTCAGAAAATTTGTGATTAGGGGTG???????????????????????????????????????????????????????????-??????????????????????????????????????????????????????????????????????????????????????????????????????????????????????-??????????????????????????????????????????????????????????????????????????????????????????????????????????????????????????????????????????????????????????????????????????????????????????????????-?------------------????????????????????????????????????????????????????????????????????????????-?????????????????????????????????????????????????????????????????????????????????????????????????????????????????????????????????????????????????????????????????????????????????????????????????????????????????????????????????????????????????????????????????????????????????????????????????????????CGGTAACACTGACTGTGATAATGTAGCGTAATCAATTGTCAATTAATTGTTGACCGGTATGAATGGTATCCCGAAAGTTTTTCTGTCTTAAAAAAATATTCAATGAAATTAAATCTGTAGTGAAGATGCTACATTTTAATTGTTAGACGAGAAGTCCCCATGGAGCTTTACTGTAAGCTTATATATATAATTTTTTTTTTTTTATAAGTAAGACAGTTTTGTTGGGGCGACAGTTTTTTAAAAAGTAACGAAAATGAGCTATGACGCATGTTTAACTTTGAAATTTTTTT-ATTGATGAGACATTTTTGGTGTGTTTTTTGATCCGTTATTTTGAATGAAAAAAATAACGAAAACAAATAAAAGTTACCCTGGGGATAACAGCGCAATAATGTTTGAGAGTTAATCAACGACAGTGTTTGCGACCTCGATGTTGAATTGTAACGTCCTACGGTGTAGTCACTCGTAAGGGTTGGTTTGTTCATCCATTAAAGTTATACATGATTTGAGTTAAAAGCGTGGTGACACAGCTTGGTTTCTATCTACAATTAGAAAACAAAAATATGTTGTTTTTTCGTACGAAAGGATCAAAAATCAATAGTTCTCTTAATATAACTATTCTTTAAATTAGGATTGTTTCTAAAAATAAAAAGAAATATTTTTGTTTGTTGTTTTTATTAATGTATCTTTTAGTTTTATTTTTTCCTTTAATTGGAGCTGTTTTAACAGGTTGTTTTGGAAGAAAAATCGGAGAAAGAGGAGCGGGGATTTTAACTTCAAGTTGTTTAGTTTTTAGTTTATCTTATTCTTTTTTAATCGCGATTGAAGTTTTATTTAATTCAACAACAACGTATTTGAAATTATGAAAATGATTTGATTCGGGGTTGTTTGTTGTTTTTTTTGGTTTTCAATTTGATGGTTTAGTAGTTATTATGTTATTTGTTGTTTTTATTGTTTCTACTTTGGTTCATATTTTTTCTATTGCTTATATGCGGGGAGATCCTCATATTCCTCGATTTATGACATATCTTTCTTTGTTTACTTTTTTAATGGTTTTATTGGTAACTAGCGATAATTTTCTTCAATTATTTATTGGGTGAGAAGGGGTTGGTCTTTGTTCTTATTTATTAATTAATTTTTGATTAACTCGATTAGAGGCAAATAGAGCTGCTATTAAAGCAATGTTAGTGAATAGAATTGGTGATATTGGGTTGCTTTTAGCAATGTTTTTACTTTGGGATCTTTTTGGATCTCTAGATTTTTCTACTATTTTTAATTCTATTTTTTTTTCTAATCAAATATTTTTTATTTGTTTATTTTTATTTTTTGGGGTTATGGGAAAATCTGCTCAATTAGGATTACATACTTGGTTACCGGATGCAATGGAAGGTTATTGGGCCTTTTAATTAAAAAATTAATTAAAAAAATTACTATACATTAAAAAAACAATTTGTTAGTAAAATATTTGTTTATTAATGGACAATAAAATATTTATTTTAATGTCTAAGAGACTTTATGTGATTTACTTTTTTTTATTTGATTAAATCTATTATTATTATTATTCCTTTACTTATTGCTGTTG?????????????????????????????????????????????????????????????????????????????????????????????????????????????????????????????????????????????????????????????????????????????????????????????????????????????????????????????????????????ATTAGTGATTTTAAAATTAGTCTTTTATGAATTTTGGCTATTTCTTCTATTAGTGTTTATGCAATTTTAATGTCTGGATGAGGAAGTAAGTCTAAATATGCTTTTTTGGGTGCTATTCGGGCGGCGGCTCAAATGATTAGTTATGAGGTTTCAATTG????????????????????????????????????????????????????????GTTTTAGCGCAAAATAATGGTATTTGGTTTTTTTTTCCACTATTTCCTGTTACAATAATGTTTTTTGCTTCGATTTTAGCAGAGACCAATCGTGCTCCTTTTGATTTAACAGAAGGAGAGTCAGAGCTTGTTTCGGGATATAATGTTGAGTATGCTTCGATGTCTTTTGCTTTATTTTTTCTTGCTGAATATGCCCATATTATTTTAATGAGTTGTTTAACAACGATTTTTTTTGGGGGAGGATGGCTTTCTCCGATTCCAGATTTTAAGGGTGGGGCGGGCTGGTTTGGTTTTAAAGTTGTTTTTATTATTTTTTTTTTTATTTGAGTAAGGGCTTCATTTCCTCGAATTCGATATGATCAGCTTATGGCTTTGTTATGAAAAGGGTATTTGCCTTTAAGTTTAGGAATGGTCATCTTTGTGGCTAGTGTTCTCTTTGGGTTTAATGGTCCCCCTCCTATTTAAAATATTTTAATTAATTATTATGAAAAAAAAAAAGAACTAACAGATTTGCAAGTGCCATTACGAAAAAAAAATCCTATTTTATTTTTAATCAACGGATTTTTAGTTGATTTGGTTTCTCCTTCTAACATTACTTATTTATGAAATTTTGGATCTTTATTGGGATTGTGTTTGATTTTACAGATAGTGACTGGGTGTTTTTTGTCTATGCACTATTGTTCTGATGTTAATTTTGCTTTTGCTTCAATTGGTCATATTATGCGAGATGTTAATTATGGGTTTTTATTAAGATATCTTCATGCTAACGGTGCTTCTTTGTTTTTTTTTTGTCTTTATGTTCATATTGGTCGAAGTTTATATTATGGGGGGTATTTAAAATTTCATGTTTGAAGCATTGGAGTTGTTATTTTTTTATTAACAATGGCTATTGCCTTTATGGGCTATGTTTTGCCTTGAGGACAAATGTCTTTTTGAGGGGCAACTGTAATAACGAATTTATTATCTG?????????ATTTTGGGATTGATATTGTTCAGTGAGTGTGAGGCGGGTTTAGTGTTTCTAATGCAACATTAAATCGGTTTTTTAGTTTACATTTTTTACTTCCTTTTATTTTAGTTTTTCTTGTT?????????????????????????????????????????????????????????????????????????????????????????????ACATCAAAAGATTTTTTTGGTTTTTTTTTTCTTTTTTTTTTATTTTGTTTTTTTGTTTTTTTTTTGCCTAATTTATTAGGAGATGCTGAAAATTTTATTCAAGCGAATTCTTTGGTTACTCCGGTTCATATTCAACCAGAATGATATTTTTTATTTGCTTATGCAATATTACGTTCTATACCAAATAAATTAGGTGGGGTTATTGCAATGTTTTGTAGTATTTTTATTTTATTTTTTTTATCTATTTTACATCAAAGTCTTTTAAAGGGACTTTTTTTTCGTCCTTTAGGTCGAATTGCCTTTTGGTTTTTAATTATTGATTTTGCTTTATTAACTTGAATTGGATCACAAGTTGTAGAAGAACCTTTTATTTTAATTGGTCAAATACTTTCTTTTTTTTATTTTTTTTATTTTTTAGTTTTAATACCAGTTTTGGGTATTATTGAAAATCAATTATTAAATAAAAATTAATGAAATTTTTGTTTTAGGCGGTTTTCTTTT???????????????????????????????????????????????TTTTTTATTTTTTWTTTTTTTTTTTTTTTTTTTTTTTTAAATTAGAATGGGGAAAGCTTTTGGTTTTAATTGGTTCTTTTGCTGTCTTTCTTTTATTTGGACCAAAAAAAGAAGAACAAACAGATGTC???????????????????????????????????????????????????????????????????????????????????????????????????????????????????????????????????????????????????????????????????????????????????????????????????????????????????????????????????????????????????????????????????????????????????????????????????????????????????TAATTATTGTTGCTCTTTTTTTTAAATTATCAGTTGCTCCTTTTCATATGTGAGTTCCTGATGTTTATGAGGGGGGACCTACTAAAATTATTCTATTATTGGCTATTGTTCCAAAAATAGGGTTTTTTTCTCTTATAATTTCAATTGGATT?????????????????????????????????????????????????????????????????????????????????????????????????????????????????????????ATTTTATGAGGTTTGGAAAATGGTTCTTTTGAAAGTTTACAAGCTAGTCTTGTTTATCTTTTTATATATATTGTGATGACAATTTGTGCTTTTTCTATTATATTAAGTTTTAATGTTTATAAAAAT????????????????????????????????????????????????????????????????????????????????------------------TGGCTTTTTTGGAAAATGATTTATTTTGTTATCTGGAATTCTTTCTAAATCATATTTTATCTTTTTTTTTGCTGTTTTTTGTTCTGTTATAGCTGGGGTTTATTATATTCGAATTATAAAAATACTTTTTTTTC???????????????????????????????????????????????????????????????????????????????????????????????????????????????????????????????????????????????????????????????????????????????????????????????????????????????????????????????????????????????????????YYYTYTYWWTKKWYTSMTTGGTTCTTGTTTTTATAAATTCTGCCGTTTTTTTCCTTTTACTAGGAATAGATTTTCTTGCTTTGATGTTTTTACTTATTTATGTTGGGGCAATAGCTATTTTATTTTTATTTGTTATTATGTTATTAAATCTAACTGACTACCCTCCTGTTTTAAAAAGAGAGGTTGATATGACAAATTATATACCAATTGGATTTATAATTGGGATTTTTTTTTTTTCAGAAATTGCTTCAAGTGGATTACTTTTGGGTTCTTTTCAAATAGAGAATTGAGACCTTTCTTTTCCTTGATTTCTTATTTCTTATCATAATATTGAGGCCTTAGGGCAGATTTTATATGTTTCTTGTTTTTGTTTATTTCTTTTGGCCAGTTTTATTTTATTAGTTGCTATGATGGGTGTGATTGTATTAACTCAAGAAACAGAATCTTTAAGTAAAAAACAAGATCTTTTTTTCCAAATAAATAGGTAATGAATAGTTCTTCTTATTTTGAACAATTTAACATAGTGTGATTGTTTGGTTTTACGAATTCAACAATAATGATGACTTTTGTAATTATTGTAGTTTTATTATTTTTTAAAGGAATTGAATTAATTCCAAAAAGATGGCAGTCGGTTTATGAATGTCTAGAAAATTATTTTTATTATATAACAGTGCAAAATTTAAGTAATGTGGGTTTAATATATTTTTCTTTTATTGTTTCCCTTTTTGTTTTTTTAGTTTTTTTAAATATTTTAGGTTTATGTCCTTATGTTTTTACTCCGACAACTCATATTATTGTTACTCTTGGGTTTTCTTTTTCTATTGTTATTGGAGTAACTTTTTCTGGATTTTATAAATTTAAAAAAGATTTTTTTAGTATTTTAATGCCTAGTGGGGCTCCTTTAATCTTGGCACCACTTTTAATTTTAATAGAAA??????????????????????????????????????????????????????????????????????????????????????????????????????????????????????????????????????????????????????????????????????????????????????????????????????????????????????????????????????-----------------?????????????????????????ATGCACATTTTTAGGTATATCGTTAACGGTGAATTATGGATTTGGTATTGGGTGATATGGTAGTTCTTCTCAAGCCTGGTTAAGGCACCAAATGGGC---ATAGACGGTGCAGATATCGTCCATCCTTGAGCTTATTTTAATTATGTGCGCGAGCATTATATTGCTCGTAGTGTTGGGGTACAAGTGAATCTAGATTCAACTGAAGGACCAAATGATGTTGGGGTACAAGTGAATCCAGAGTCTGAACAGTCTGTGGATGTTGTTTTTATAGAATTGGAAGGGCGAAGGGTCCCCGTAAG--GGGGGTGA------------------ATCTACATGTTGAGCGGCGT-GGTTATTTGGATAATCG---GTCTAATCAAGAAGG--TGT-TGA---------------------------------------------GAATAGTTTAGGAGATCA-----ATTTGA-AGATGTGGCTTT---TTTAG-GGGGGAG-TACAAGTACAATTACACCAGAAA----GTGTAAATAGGGGCGCTCCAAGTGGTAATATAAGAAATGTATTAAGTGATTCATTAA-TAGA-G-AG-GAGAGGCCAAGACCAAACTTTAAGTGA--AA-TAAAGGTAGAAACACCTCC----AGGTGCTTCAAGGGGTGGCCGATG-A-------G--CCAGT-AT-----AAATCAAGGTGAGGCTTTAAG---AGAAAACAAGGTAGAAATTGGGG----TTTTAAGAGAAAACAAAGT--------AGAAAT--TGATGAGGTAGTTGGCTCGACTTTTAGTTCGATCCCCTTAGAGGTTGAAGGGATGATGGCTCATGTGTGAAATCAATTTGACATGGTGTTTAACATATTGGCCGATAAGCCCCTGGTAGGTTGCATTTTTTTTTCTTACCAGCTTCTAAGAATAACGAATCAAATGCCAAACCCACGGGTTTATAGGTGATTTGCTTTGGTGTATCTTTTATTTATTTTTGTTCTTTTAATGGTTTAGCTGTTTGTACGTAGTAGGATAAAAAAGTGGGACATTTTTCTAGGAGTTTATTTGTATGCGTCTGGGGGGATTAGAATAAGAGTAAAAAAAAACTCTTTAAAAGTTTACAAAAAAAGTGGGGTGTTTTTTTTGGAGTTTTTTTTTATG???????????????????????????AAAAAAAAAACTCTTTAAGAGTTTACAAAAAAAGTGGGGTGTTTTTTTTG????????????????????????????????????GA???????????????????????????????????????????????????????????????????????????????????????????????????????????????????????????????????????????????????????????????????????????????????????????????????????????????????????????????????????????ATTGTTATTATGGCATTAGTTAACATCATAGGGACAGCGAGGGAGAGAAAATGTGTTTTAAAAAAGCGGGCTTTAGAGTGGTCTTTGGCTTTATTGTTTAGTACTTTAGTTTTTTGGGGTGGATTTGACGGAGAAAGTCATTTTCAATTTTTTAGTTTAGTAGAATGAAATATATTTTCTACTTTAGATTGAGGCCCGATTGTTTTTGCAGTTGATGGTGTTTCTTTGGTTTTTTTACTTTTAACGACTTTTTTAATTCCAATTTGTATTTTAATTAGCCAAAAATCAATAAAATTCTTATTTAAAGAATTTCTTTTGTGCTTGTTTTTTTTAGAATTTTTATTAATAGGTGTTTTCATAGTGTTTGATCTTCTTTTGTTTTATCTTTTTTTTGAGGGGATATTAATACCAATGTTTTTTTTAATTGGTATTTGAGGCTCTCGAGAAGAAAAGGTTCGTGCTTCTTTTTATTTTTTTTTTTTTACTTTTATAGGCTCTCTCTTTTTCTTTTTTATAATACTTTTTTTATATCAAAGGATTGGAACAACAGACTATTTTCTTTTACTTAATATTAAATTATTTTTAAATATTCAAAAATGAGCCTTAGTCGGGGTTTTTCTTAGTTTTGCGGTGAAACTACCTCTTATTCCATTTCATATTTGATTGCCACAAGCACATGTTGAGGCTCCTGTTGCGGGCTCTGTTATTTTGGCTGGAATTTTATTAAAATTAGGGGGTTATGGCCTTTTGCGTTTTTCTTGACCTCTTTTTCCGGGGGCTTCTTTATATTGGTCTCCAGTTATTGTTTTTTTTAGTGTTGTTGCTGTTGTTTATGGAGGTTTAATGACATGTCGTCAAATTGATTTTAAACGGCTTGTTGCTTACTCTTCTGTTGCTCATATGGGACTTGTGCCTTTGGGTCTTTTTACACATGTTATAGAGGGGTTAATTGGGGCTCTTTTTTTAATGTTGGCGCACGGATTTGTTAGCTCTGCTCTTTTTATTGGAGTAACTTTTTTGTATGATCGCCATCATACTCGTTTAATAAAATATTATCGGGGTTTGACTTTGACAATGCCTCTTTTTGTTATTACAATGTTAATTTTGTCTTTGGCGAACATGGGTTTTCCTCTTAGTTGTAATTTTGTTGGAGAGTTTTTTTCTTTATTAGCAGTCTTTCAATATCATTATGGAGTTGGAATGTTTGTTATTTTAGGGGTTCTTTTTTCTGCCATTTATTCTCTTAGTCTTTTTAATCGTATTTCTTTTGGTGGAGGCTCTAATTATTTACTTTTTAATAGAGACTTAAGTCGACGAGAGATTTTTGTAATTTTTCCTTTTCTTTTAATTATCTTTTTGGGGGGGATTGTGCCTTTTCCTATTATTGATTTAATTAAAAATAGTCTTGTTTTTAGCCCGGTTAGTTAG-------------TCCTGGGGTTTTAGGGAGAAAAAAATATTTAGTCTAGGTTATACATGCTAGTGTATGCGAACAGGTGAGGATAA-AATAAAAAAATTTTTTTTATTTGTTGTATTAAAAAAAGAAAAGTTTTTTA-TTCTTTTTTTGAAGATGCATAGTT????????????????????????AGAAAACCTTTGGCAGCAGTAATGAATTTTATGTAATATACGAAAGTAAGACATAGGTAGGAGAAAAAAAATCTGTCAAAT-TAAGTGCCAGCCGACGCGGTAAGACTTAAGGATTTA-TTTTTTAATAAAAAGCAAAAAGCGTGTTAAGGATT????????????????????????????????--??????????????????????????????????????????????????????------????????????????????AAGGTTTGGGGAGCAAATAGGATTAGGTACCCTGGTAGTCTATACAGTAAAATAATATCGCTTGAGTAGTATGGTCGCAAGATTGAAATTCAAAAGACTTGGCTGTTC-ATTGTTTATTAGAGGAGCGCGTCGCTTAATTCGATTATCCGCGAGAGACCTTACCGAAGTTTGAATTTATTAACAGGTGTTGCATGGCCGTCGTCAATTTCTGTTTGAGACAAAAAGGGGTTT??????????????????????????????????????????????GGTTAGGCGCGCTACATTTCTCTCTT?????????????????????????????????AGATAATGAAGTTGAATTTAATAGTAATTGAAAAGTAGTGCGTTTCAATGAATGTTATTGCAATGTTAGTACAAATAGTCCGTCGCCAGCTTAGAAATAGGCTGTAAGTCGTAACATAGTAAGAGTGAGGGAACTGGCTCTTGATGCAGTTTCATTCGTATCATTTAGTTG?????????????????????????????????????TTTTTTTATTACTGTTGGTGCAGTGGTTTTTTTTCATTATGGGTTTAGTTTTTTTTTATATTTGGGGCTTTTGGTTGTTGTTGGAGTTATGTTTGTTTGATGACAAGATGTTATACGAGAATCAACTTTTCAAGGTC?????????????????????????????????????????????????????????????????????????????????????????????????????????????????????????????????????????????????????????????????????????????????????????????????????????????????????????????????????????????????????????????????????????????MAATTTGCTTTATTTTTAACTCTTTTTTTGGGTGTTATGTTTACGGGGTTGCAAGCATTTGAATATTATGAGGCGCCTTTTACTTTATCTGACTCTGTTTATGGGGCCACTTTTTTTGTTGCGACAGGGTTTCATGGACTACATGTTATAATTGGTACGACTTTTCTTTTTATTTGTTTTTTACGTTTACTCTCTAATCAATTTACTCGTCGTCAACATGTTGGGTTTGAAGCCGCGAGTTGATACTGGCACTTTGTCGATGTGGTTTGATTATTTTTATATTTATGTATTTATTGATGAGGCTCATAAAAATTTCTTTAATGCAAAATCTTTTTGTTTTTACTTTTTTTAGATATTTTATAAATGTTTTGGCTGGGTTGTGTGTTCTCTGGTTTAATCATCTTTATTATTCGGATAGTCCAGAGACTTGACTTTTAGAGTTTCAAGATGTTGGGGATCCTATTGTAGAAGAAATTGTTTTTTTTCATGACCAAGTTATGTTTTTATTAATTATTATAGTTACTGTTGTTTTATGACTTTTTGTTGAAGCTTTTAAAAATAAGTTTTATGATCGTCATTTAATCGATGGTACATTTTTAGAAATTGTTTGAACGATTATTCCTGCTGTTATATTGATTTTTATTGCATTGCCCTCTCTTAAATTATTATATTTAATGGATGAGGTTATTTCTCCAGCTTTGACAATTAAAGTTATTGGGCATCAATGATATTGATCCTATGAATATTCTGATTATGAAGGGGATACGTTAGGGTTTGATTCTTATATGATTCCGACTTCGGATTTAGTTTCTGGAGAGAATCGTTTGTTAGAGGTTGATTATAAACTTTTAATCCCTATTCAAACACATATAAGATTTTTAGTTACTGGGGCAGATGTTTTACATTCTTTTGCAGTGCCTTCTTTAGGGCTAAAAATTGATGCGGTTCCGGGTCGTCTAAATCAAACTGGTGTTTTTATAAAACGAGCCGGGGTTTTTTTTGGACAATGTTCTGAAATTTGTGGGGCAAATCATTCTTTTATGCCCATTGTGATAAAGGGAGTTGGTTTAAATGAATACGTTCAATATTTAAATTATTTAAAATGTATTATAAATACTTAGTTATTATTATTATTTTATTTTTATTGGGGAGTTGGGGTATAATTTTAAATAGAGGACATTTTATTATTATGCTTGTTTCTATTGAATTAGTTTTATTATCAACTTTTTTTTTTTTTTTAATAAGTTCTAAAGAAATAGATCTTTTAATAGAACAAATTTTTATGATTATGGGGTTAACTATTGCTGCTGCCGAATCTTCAATTGGTTTAGCTATTTTAGTTGCATATTATAGAATTCGAGGAACAATTGTTTTAAAATCTTTTAGTTCTTTGCGAGGATAATGATTAATCTTTTTATTTTTTTTTTTCTTATTTTTATTTTGGCGGGGTTATTTATATTTCTTTCTTTTTTGATAGGGGAAAAAATTCCAGATCGAGAAAAGGTTTCTGCTTATGAGTGTGGTTTTGCTCCCTTTAATTTTTTAGGACGCCCTTTTTCAATACGTTTTTTTTTAATTGGTATTTTATTCTTGATTTTTGATTTAGAAATTTCTTTTTTTTTTCCCTGATGTGTTTTATATAATTCGACTGCTCCGTTTGGGTTTTGAACTATGATAGGTTTTTTTTTTGTATTAGTTTTGGGTTTGATATATGAATGAGTAATGGGGGGATTAGAATGAGAGTAAAAATAAAGTAAAAGAAAAAGTCCTATCTATTATGAAAATAATAGAAATTTTATACCAACTCCGGTTTCTGCTTTAATTCATGCTGCAAC???????????????????????????????????????????TTATTTGATGTTGTTCCTCTTATATTAATTATTATTTCAATCGTTGGGGTTTTAACCGTGTTTATTGCAGGCACAATTGGTTTAGTTCAAAATGATTTAAAAAAAATAATCGCTTATTCTACTTGTAGTCAATTAGGTTATATGGTTGTTGCTTGTGGGCTTTCTCATTATGCAATTAGTCTTTTTCATCTTATGAATCATGCTTTTTTCAAAGCTTTATTATTTTTGAGTGCAGGGTCTCTTATTCATGCGGTAATTGATGAACAAGACATAAGAAAAATGGGGGGTTTATTATCTTTTCTTCCTTTGACTTATGTTTTTTTTCTTATAGGCTCTTTTTCTTTAATGGGGTTTCCTTTTTTAACAGGATTTTATTCAAAAGATTTAATTTTAGAATTTGCTTTTGGACAATT????????????????????GATTAGGTTGTTTTTCTGTTTTATTGACAATTA?ATACTCTATTCGTTTAATTTATTTAGTTTTTTTATCCAATATTAATTTAAAACGAGCAAACATTTTTTTTCTTAAAGAAGGAGAATTTTTATTTTTAATTCCTTTGGGCATATTAACTTTAGGAAGTGTTTTTTGGGGCTATTTAAGTAAAGAAATAATTTGGTCTTTTCAAATAGATGTTTTTTCAATACTTTCTTTAAAAATAAAAATATTTCCAATTTTATTTTGTTTTATTGGACTTTTTGGGACGATATTTTTTTTTTTTTTTTTTTCTTCTCAAATTTTTGGTTATCCCCTTCAGGTGATCGGATCTTCTGTTTTTTCTCTTTATAATTTTTTTGGTTCTGCTTGACAAATAAATTTTTTTTTTAATTTTTTCTTTATAAAAAAAATATATAAAATAGGACATCTTATTACTAATTTAACAATTGACAAAGGTTTATTAGAGGTTGTTGGGCCTAGGGGTGTTGTTCAATTTTTTATTTTTCAAACTCAAAAGTTGAGTAGTATACAATCGGGGTTGGTATTTAATTATGCTTTAGTTTTTTTTCTTGGGATACTTTTTTTAATTTTTGCACTTTAATTAAAATTTTTGAAGGGGTTAAGTTAAGCAAAACAGTTGACTTTCAAAGTTAATTATGTAGGTGGAAATCTTACACTCTTTGGATGCCACAGTTAGAGGTAGGTACTTATAATATACAATATTGGTGGGGGTTCTCGGTTTTATTTTTCTTGTTAATTTTTTTAGAAGTTATAATCTTTCCTTTTGTAAAGCGTGGTTGATGAATAAGACAGTTCTTATTGGGGTG---------------AACCTTTTTTTTAGGGTGAAAAAAAAATTATGGAAAAAGATTTCCATGAGAATTGTCGCAAAATGTTTAAAAAAAGCCTTT-TTTTTGCT--AGTGGGATTTTGTTTAAAATATTTTT-TTGAGTTTTCTTACTTAAAAACATCTTTTTGTTTAATGA-TTTTTTTTTTATTTAT-------------------------------------AAAATTTTGT------------TCTAAGAAGATTC----------------------------------------------GGGCTCGTTTAGCGATC?????---??????GATC-TTT---GGGGATGGTGT--------AATGGAAGTAGATGAG-CCAAATGA--AGAGATAGTAGGAG--CGCTTCCATCTTTA--------AGCTTCGTGGAGGGGTGAGATG----------------------------AAGAGGTGATAGCAGCGTTTCCATGTTCAGACT----------CCGAAGAGAGTATGGAAATTGATGGGGAAGA--ACTACCTTTGGATCCCCTCCCGATTAGTGGAGCT--ATTAGAGGAGAA--AACATAGTGGCA-------------------------------CCCGTACCAGCCATGGGAGATGCGGGTCTTTCAGTTCATTTTTGGCGTGCACTCAAGAACACTCCATTTGAATTAT-TGGCAGAAGTTTATCGGTCAGGTGAGGAGGGAGAAG--TT---T--ATTCGTCAGATGAG---GAAGACGAGTTAGTAGAGGTGTTACTTCAGTACGGAAA-AGAGAT---------------------AACATCAAAAGAGTCAGAGGACAAATTTTTGAGTGGGCTAAT--AGT--GGAAATGGTGGAAAACGTAATTGTCT-AAGATAAATACCA-GAA-CAGTT-AGTAAACAGATTATTGAGTGGGTCAGTGGA--CAAAGTAGGGGATAACGTGGTTAGGTAAAAT-ATAGTGTCGGCGGCAACGGAAGTGGGGAGGGGGAGAGCTCTCTTGCTCTCTAATTTGTAGTTTTTGAGTGGTTTGTATTATTTTTT--TTTTTAATTTTTTCTTTATGAGTCTGTCTTGGTAGAGGAG-GCGATTTTTTTAGAAGCCCCGTCTAAATGCTTTCTAGTTATTGAACTCGATGGGTGTTTTCTACAAATCATAAGGACATTGGTACTTTGTATTTAATCTTCGGAGCAGGAGCTGGTTTAATTGGAACTGCTTTTAGTATGCTTATACGATTGGAGCTTTCTGCGCCGGGGGCGATGTTAGGAGATGATCATCTTTATAATGTAATTGTTACAGCACATGCTTTTATTATGATTTTTTTTTTGGTTATGCCGGTCATGATTGGGGGATTTGGTAATTGATTAGTCCCATTATATATTGGGGCGCCGGATATGGCGTTTCCTCGATTAAACAATATTAGTTTTTGACTTTTGCCTCCTGCGCTTTTTTTATTATTAGGCTCTGCTTTTATTGAACAAGGGGCGGGGACGGGGTGAACAGTTTATCCTCCTCTTTCTAGTATTCAAGCACACTCCGGAGGTTCTGTTGATATGGTTATTTTTAGTCTTCATTTAGCTGGGGTTTCTTCTATTTTAGGTGCTATTAACTTTATTACTACAATTTTTAATATGCGAGCCCCGGGTGTGTCTTTTAATAAACTACCTTTATTTGTTTGATCTATTTTAATAACAGCTTTTTTATTACTTTTATCTTTACCTGTTTTAGCTGGTGCTATTACTATGTTGTTAACAGATAGAAACTTTAATACGACTTTTTTCGATCCAGCGGGTGGCGGGGACCCAATATTATTTCAGCATTTATTTTGATTCTTTGGGCATCCAGAAGTTTATATTTTAATTTTGCCTGGTTTTGGTATGATTTCTCAAATAATCCCGACTTTTGTTGCTAAAAAACAAGTTTTTGGGTATTTAGGAATGGTTTATGCCATGCTTTCTATTGGGCTTCTTGGATTTATTGTTTGAGCTCATCATATGTTTACTGTTGGGATGGATGTAGATACAAGAGCATATTTTACTGCTGCTACTATGATTATTGCTGTGCCAACTGGGATTAAAGTTTTTAGTTGGTTGGCAACTATTTATGGAGGTGTTCTTAGGTTAGAGACTCCAATGCTTTGAGCTATGGGGTTTGTTTTTTTATTTACAGTTGGTGGTTTAACTGGGGTTGTGTTAGCAAATAGTTCTCTTGATATTGTTCTACATGATACATATTATGTAGTTGCGCATTTTCATTATGTTCTTTCTATGGGGGCTGTTTTTGCTATTTTTGGGGGGTTTTACTATTGAATTGGCAAAATAAGTGGTTATTGTTATAATGAATTTTTTGGGAAAGTTCATTTTTGATTAATGTTTATCGGGGTTAATCTAACTTTTTTCCCTCAACATTTTTTAGGTTTAGCAGGATTTCCAAGACGATATTCGGATTATGCAGATGCTTTTTTGGGTTGAAATTTAATAAGTTCTTTAGGGTCTATTATTTCTATTTTGAGTGTTGTTTGGTTTTTATATATTGTTTTTGATCTTTTTGTTACAGAAGAAAAATTTTTGGGTTGAAAAGAAGGATTTTCTTTAGAATGAATTCATTCTTCTCCCCCCTTATTTCATACTTATGAGGAGTTGCCCTTTGTACAAAAAGTAAATAATTTTTAGAATAGTGCCGGGTTTATATACCGGTTT

S2 TGTAAGAAAGACAAAGGGTTAGTCATTGGGTTCATGCCCCAACCAAGTGAGTTCGAATCTCTCTCTTACACAAAATAAAAATTAT-AAAAAAAAAAGAGGATAACTAAGATAAACTAAAATTAGACGATTTTTTTCGAAATGGCGTTCAGTTGCTTATTGGTTGTTTCTTCAGAAAAGTGATTTTTTTTTTTATTTGAATTGAAACATCTTATTACTAAGTAAAAATCAAACGAGATTCCGAGAGTAGTGGAGAGTGAAATTGGAGTTGTGCTTGTGGTGGTCATAGATCTTGCTTAAACATTAGTTTATACTGATAATGAAAGTACTGTAAAGGAAAGTTGAAAGAGAGTTGAAAGGAATTTGAATCTTTTATTTTTGAAGTAGCTTTAAAAAAGCGTACCTTTTGTATAATGGGTAAAAGAGATTTATTTGGCATATTTAAAATGGAAAATTTTAAGTAA-TTTTTTTTTAGTCAAATTTCCCGAAACCAAGTGATTTAATCATGAATTGTGTGAGCAAAAACTGGTGATTGTGGCAAAAATCTCAGAAAATTTGTGATTAGGGGTGAAAGGCTAATCGAACTTGGAAATAGCTGGTTTTCTGCGAAAACTATTTAAGTAGTGTTC-TTTTTTTTTTTATTTTAATTGTCATTTTAAAAAAAACAAAAATAAAAAAAAAGTAAACAAACAATAAATGAAAAGATTTTTTGTTAAAAGAGAAAGCTCAAATCAGAAGCTATTGTCA-TTTTTTTTTTTTTAGTTTAAAAAAAAATTAAATTTAGACAATAAAAATGTAGGCTTGGAACCAGCCATCTTTTAAAAAGTACGTAGTTGTTTATTTAAAAATTTTAAGTTTTTTTATAATTTTGATGGTTAAAATAAAACAGAAGCTCTGAAAATAATTATATATTTCGTAGCAGAATAAATTGTTTTCGATAT-C------------------TCCCTAGTTTGAAATTTTTTTTCTTAATACATCGTGTTCAGTTGTAATAATTTTTACATAAGTAATCTAAATATTA-TTTTTTTTTCTTAAGATTACCTTAGTCAAGGGTAATACAGTTTCTAAAAAAGAAATTTTTTATGAAGATTTTTTATAAAAATATATAATATCGGGAAAAGAAAAAAAAAAGAACTGTTTTTCTAATTAAAGAAAGAAAAAAAAAAAGAAAAACATTTTTTTAAGGAACTCGGCAAAGTTAAACTTCGACTGTTTACCAAAAACATAGCTTTTTGATTTTTATAAAAGGTGAAACCTGCCCGATGGTTGAATCTTAATATGTTTGTTCCGCTTACTAATAAAGACAATTAAATGGCCGCGGTAACACTGACTGTGATAATGTAGCGTAATCAATTGTCAATTAATTGTTGACCGGTATGAATGGTATCCCGAAAGTTTTTCTGTCTTAAAAAAATATTCAATGAAATTAAATCTGTAGTGAAGATGCTACATTTTAATTGTTAGACGAGAAGTCCCCATGGAGCTTTACTGTAAGCTTATATATATAATTTTTTTTTTTTTATAAGTAAGACAGTTTTGTTGGGGCGACAGTTTTTTAAAAAGTAACGAAAATGAGCTATGACGCATGTTTAACTTTGAAATTTTTTT-ATTGATGAGACATTTTTGGTGTGTTTTTTGATCCGTTATTTTGAATGAAAAAAATAACGAAAACAAATAAAAGTTACCCTGGGGATAACAGCGCAATAATGTTTGAGAGTTAATCAACGACAGTGTTTGCGACCTCGATGTTGAATTGTAACGTCCTACGGTGTAGTCACTCGTAAGGGTTGGTTTGTTCATCCATTAAAGTTATACATGATTTGAGTTAAAAGCGTGGTGACACAGCTTGGTTTCTATCTACAATTAGAAAACAAAAATATGTTGTTTTTTCGTACGAAAGGATCAAAAATCAATAGTTCTCTTAATATAACTATTCTTTAAATTAGGATTGTTTCTAAAAATAAAAAGAAATATTTTTGTTTGTTGTTTTTATTAATGTATCTTTTAGTTTTATTTTTTCCTTTAATTGGAGCTGTTTTAACAGGTTGTTTTGGAAGAAAAATCGGAGAAAGAGGAGCGGGGATTTTAACTTCAAGTTGTTTAGTTTTTAGTTTATCTTATTCTTTTTTAATCGCGATTGAAGTTTTATTTAATTCAACAACAACGTATTTGAAATTATGAAAATGATTTGATTCGGGGTTGTTTGTTGTTTTTTTTGGTTTTCAATTTGATGGTTTAGTAGTTATTATGTTATTTGTTGTTTTTATTGTTTCTACTTTGGTTCATATTTTTTCTATTGCTTATATGCGGGGAGATCCTCATATTCCTCGATTTATGACATATCTTTCTTTGTTTACTTTTTTAATGGTTTTATTGGTAACTAGCGATAATTTTCTTCAATTATTTATTGGGTGAGAAGGGGTTGGTCTTTGTTCTTATTTATTAATTAATTTTTGATTAACTCGATTAGAGGCAAATAGAGCTGCTATTAAAGCAATGTTAGTGAATAGAATTGGTGATATTGGGTTGCTTTTAGCAATGTTTTTACTTTGGGATCTTTTTGGATCTCTAGATTTTTCTACTATTTTTAATTCTATTTTTTTTTCTAATCAAATATTTTTTATTTGTTTATTTTTATTTTTTGGGGTTATGGGAAAATCTGCTCAATTAGGATTACATACTTGGTTACCGGATGCAATGGAAGGTTATTGGGCCTTTTAATTAAAAAATTAATTAAAAAAATTACTATACATTAAAAAAACAATTTGTTAGTAAAATATTTGTTTATTAATGGACAATAAAATATTTATTTTAATGTCTAAGAGACTTTATGTGATTTACTTTTTTTTATTTGATTAAATCTATTATTATTATTATTCCTTTACTTATTGCTGTTGCATTTTTAACTTTAGCAGAACGAAAAATTTTAGGGTATATGCAAATAAGAAAAGGGCCAAATGTTGTTGGGGGAGGGCTTCTTCAACCTTTTGCAGATGGGGTGAAATTGTTTATTAAAGAAATGATTCTTCCCCATCAAGCAAATAAATTTGTTTATCTTTTGGCTCCAGTTATATCTTTTACATTAGCTTTCTTTGTTTGAGGTTTTCTTCCTTATGAGAAAGGAGTGAGTATTAGTGATTTTAAAATTAGTCTTTTATGAATTTTGGCTATTTCTTCTATTAGTGTTTATGCAATTTTAATGTCTGGATGAGGAAGTAAGTCTAAATATGCTTTTTTGGGTGCTATTCGGGCGGCGGCTCAAATGATTAGTTATGAGGTTTCAATTGGGCTAATTCTTATTTCGGTTTTATTATGTGTTGGTTCTTTAAGTGTTACTGAAATTGTTTTAGCGCAAAATAATGGTATTTGGTTTTTTTTTCCACTATTTCCTGTTACAATAATGTTTTTTGCTTCGATTTTAGCAGAGACCAATCGTGCTCCTTTTGATTTAACAGAAGGAGAGTCAGAGCTTGTTTCGGGATATAATGTTGAGTATGCTTCGATGTCTTTTGCTTTATTTTTTCTTGCTGAATATGCCCATATTATTTTAATGAGTTGTTTAACAACGATTTTTTTTGGGGGAGGATGGCTTTCTCCGATTCCAGATTTTAAGGGTGGGGCGGGCTGGTTTGGTTTTAAAGTTGTTTTTATTATTTTTTTTTTTATTTGAGTAAGGGCTTCATTTCCTCGAATTCGATATGATCAGCTTATGGCTTTGTTATGAAAAGGGTATTTGCCTTTAAGTTTAGGAATGGTCATCTTTGTGGCTAGTGTTCTCTTTGGGTTTAATGGTCCCCCTCCTATTTAAAATATTTTAATTAATTATTATGAAAAAAAAAAAGAACTAACAGATTTGCAAGTGCCATTACGAAAAAAAAATCCTATTTTATTTTTAATCAACGGATTTTTAGTTGATTTGGTTTCTCCTTCTAACATTACTTATTTATGAAATTTTGGATCTTTATTGGGATTGTGTTTGATTTTACAGATAGTGACTGGGTGTTTTTTGTCTATGCACTATTGTTCTGATGTTAATTTTGCTTTTGCTTCAATTGGTCATATTATGCGAGATGTTAATTATGGGTTTTTATTAAGATATCTTCATGCTAACGGTGCTTCTTTGTTTTTTTTTTGTCTTTATGTTCATATTGGTCGAAGTTTATATTATGGGGGGTATTTAAAATTTCATGTTTGAAGCATTGGAGTTGTTATTTTTTTATTAACAATGGCTATTGCCTTTATGGGCTATGTTTTGCCTTGAGGACAAATGTCTTTTTGAGGGGCAACTGTAATAACGAATTTATTATCTGCTATTCCTTATTTTGGGATTGATATTGTTCAGTGAGTGTGAGGCGGGTTTAGTGTTTCTAATGCAACATTAAATCGGTTTTTTAGTTTACATTTTTTACTTCCTTTTATTTTAGTTTTTCTTGTTATTCTTCATTTAGTTTATTTACATGTTGATGGGTCTAATAATTCAACAGGATTAAATTCTTCAATTGACGGAGTTTCATTCCATACTTTTTATACATCAAAAGATTTTTTTGGTTTTTTTTTTCTTTTTTTTTTATTTTGTTTTTTTGTTTTTTTTTTGCCTAATTTATTAGGAGATGCTGAAAATTTTATTCAAGCGAATTCTTTGGTTACTCCGGTTCATATTCAACCAGAATGATATTTTTTATTTGCTTATGCAATATTACGTTCTATACCAAATAAATTAGGTGGGGTTATTGCAATGTTTTGTAGTATTTTTATTTTATTTTTTTTATCTATTTTACATCAAAGTCTTTTAAAGGGACTTTTTTTTCGTCCTTTAGGTCGAATTGCCTTTTGGTTTTTAATTATTGATTTTGCTTTATTAACTTGAATTGGATCACAAGTTGTAGAAGAACCTTTTATTTTAATTGGTCAAATACTTTCTTTTTTTTATTTTTTTTATTTTTTAGTTTTAATACCAGTTTTGGGTATTATTGAAAATCAATTATTAAATAAAAATTAATGAAATTTTTGTTTTAGGCGGTTTTCTTTTGAGCTTCGTGGTTTTAAGTCTTCGTTATTTTCTTTTAAAACTTTCTCTTTTTTATTTATTATTATTTTTTTTTTTTTTTTTTTTTAAATTAGAATGGGGAAAGCTTTTGGTTTTAATTGGTTCTTTTGCTGTCTTTCTTTTATTTGGACCAAAAAAAGAAGAACAAACAGATGTCCCTATTTTAAGTTTAATTATTATTTTTGGAGTTTTTTGTTTAATTTCTTCAAGTAATTGACTTTCTATTTATTTAACCATTGAGCTTTTTACTCTTTGTTTTTTTATTTTGATTGCTCGAGGGTCTGGTTATAGTGTGGAAGCAGGATTAAAATATTTTATTTTGGGTGCTCTTTCTTCTGGTTTATTTTTATTTGGGTGTGCTTTATTATGTGGTATTGGGGCGAATATACACTTTTCTCATATAGAACTTCTTTTTAATTCAAAACAAATTTTTTCTGCTGTTTCGATACCAATTGGGTATCTTTTAATTATTGTTGCTCTTTTTTTTAAATTATCAGTTGCTCCTTTTCATATGTGAGTTCCTGATGTTTATGAGGGGGGACCTACTAAAATTATTCTATTATTGGCTATTGTTCCAAAAATAGGGTTTTTTTCTCTTATAATTTCAATTGGATTGCCTGTAAATTTTTTTTTTTTAGGGATTCTTTTTTCTTTGTTTGTTGGAGCTTTGGGTGCTTTAAATCAAACTAAAATAAAACGACTTTTGGCTTATAGTGGGATTGGTCATATGGGCTTTATTTTATGAGGTTTGGAAAATGGTTCTTTTGAAAGTTTACAAGCTAGTCTTGTTTATCTTTTTATATATATTGTGATGACAATTTGTGCTTTTTCTATTATATTAAGTTTTAATGTTTATAAAAATTTACTTGTTGAATTTAGTGGACTTTCTCGATACTTACCTTTTTTTTCGATTACTTTAGGTGTTCTTTTTTTTTCTATTGCAGGAATTCCTCCTTTTGCTGGCTTTTTTGGAAAATGATTTATTTTGTTATCTGGAATTCTTTCTAAATCATATTTTATCTTTTTTTTTGCTGTTTTTTGTTCTGTTATAGCTGGGGTTTATTATATTCGAATTATAAAAATACTTTTTTTTCAAAAAAATTCTTTTCTTTTAATTACAATTAAAGTTTTAAAAAAAGAATCTAAATTAAATTTTAAAAAAGTTTTTTTAATTGGTTTTTGTTTTTATTTTATTCTTTTTTTTTTTCTTTCTCCACATTTTCTTTTTTTTTTTTTTTCTCAAATAATTTTTGATTTATTTTAAAATGGAATTTTTTTCTTTTTTTTTTATTTTGGGGATAATTGGCTCAGGAGTAATGGTTGTTTCAGCGTTAAATCCTGTTCTTTCTATTTTTTGATTGGTTCTTGTTTTTATAAATTCTGCCGTTTTTTTCCTTTTACTAGGAATAGATTTTCTTGCTTTGATGTTTTTACTTATTTATGTTGGGGCAATAGCTATTTTATTTTTATTTGTTATTATGTTATTAAATCTAACTGACTACCCTCCTGTTTTAAAAAGAGAGGTTGATATGACAAATTATATACCAATTGGATTTATAATTGGGATTTTTTTTTTTTCAGAAATTGCTTCAAGTGGATTACTTTTGGGTTCTTTTCAAATAGAGAATTGAGACCTTTCTTTTCCTTGATTTCTTATTTCTTATCATAATATTGAGGCCTTAGGGCAGATTTTATATGTTTCTTGTTTTTGTTTATTTCTTTTGGCCAGTTTTATTTTATTAGTTGCTATGATGGGTGTGATTGTATTAACTCAAGAAACAGAATCTTTAAGTAAAAAACAAGATCTTTTTTTCCAAATAAATAGGTAATGAACAGTTCTTCTTATTTTGAACAATTTAACATAGTGTGGTTGTTTGGTTTTACGAATTCAACAATAATGATGACTTTTGTAATTATTGTAGTTTTATTATTTTTTAAAGGAATTGAATTAATTCCAAAAAGATGGCAGTCGGTTTATGAATGTCTAGAAAATTATTTTTATTATATAACAGTGCAAAATTTAAGTAATGTGGGTTTAATATATTTTTCTTTTATTGTTTCCCTTTTTGTTTTTTTAGTTTTTTTAAATATTTTAGGTTTATGTCCTTATGTTTTTACTCCGACAACTCATATTATTGTTACTCTTGGGTTTTCTTTTTCTATTGTTATTGGAGTAACTTTTTCTGGATTTTATAAATTTAAAAAAGATTTTTTTAGTATTTTAATGCCTAGTGGGGCTCCTTTAATCTTGGCACCACTTTTAATTTTAATAGAAACAGTAAGTTATATTTCTAGGGCTGTTTCTTTGGGGATTCGTTTAGCAGCAAATCTTTCTGCCGGGCATCTTTTATTTGCAATTTTGGCAGGATTTGGTCTGGTTTTTAAATTTGCAATGTTTATAATGGTGTTTATTACGCTATTAGAGGTGGCAGTTGCAATAATACAGGCCTATGTGTTTTGTCTGTTGGTACAAATTTATTTAACAGATACAATTTTTTTACATTAA-----------------AAATGACCGCCGTAGAAATTTTTTTATGCACAATTATAGGGATGTCGCTAACTGTGAATTATGGATTTGGTATTGGGTGATATAGTAGTTCTTCTCAAGCCTGGTTAAGACACCAAATGGGC---ATAGACGGTGTAGATACGGTCCATCCTTGAGCTTATTTTAATTATGTGCGCCAGCATTCTATTGCTCGTAATGTTGGGGTACAAGTGAATCTAGATTCAACTGAAGGACCAAATGATGTTGGGGTACAAGTGAATCAAGAGTCTGAACAGTCTGTGGATGTTGTTTTTATAGAATTGGAAGGGCGAAGGGTCCCCGTAAG--GGGGGTGA------------------ATCTACATGTTGAGCGGCGT-GGTTATTTGGATAATCG---GTCTAATCAAGAAGG--TGT-TGA---------------------------------------------GAATAGTTTAGGAGATCA-----ATTTGA-AGATGTGGCTTT---TTTAG-GGGGGAG-TACAAGTACAATTACACCAGAAA----GTGTAAATAGGGGCGCTCCAAGTGGTAATATAAGAAATGTATTAAGTGATTCATTAA-TAGA-G-AG-GAGAGGCCAAGACCAAACTTTAAGTGA--AA-TAAAGGTAGAAACACCTCC----AGGTGCTTCAAGGGGTGGCCGATG-A-------G--CCAGT-AT-----AAATCAAGGTGAGGCTTTAAG---AGAAAACAAGGTAGAAATTGGGG----TTTTAAGAGAAAACAAAGT--------AGAAAT--TGATGAGGTAGTTGGCTCGACTTTTAGTTCGATCCCCTTAGAGGTTGAAGGGATGATGGCTCATGTGTGAAATCAATTTGACATGGTGTTTAACATATTGGCCGATAAGCCCCTGGTAGGTTGCATTTTTTTTTCTTACCAGCTTCTAAGAATAACGAATCAAATGCCAAACCCACGGGTTTATAGGTGATTTGCTTTGGTGTATCTTTTATTTATTTTTGTTCTTTTAATGGTTTAGCTGTTTGTACGTAGTAGGATAAAAAAGTGGGACATTTTTCTAGGAGTTTATTTGTATGCGTCTGGGGGGATTAGAATAAGAGTAAAAAAAAACTCTTTAAAAGTTTACAAAAAAAGTGGGGTGTTTTTTTTGGAGTTTTTTTTTATGGGTCCGGGAGGATTTTAAGGAGAGGAGAAAAAAAAAACTCTTTAAGAGTTTACAAAAAAAGTGGGGTGTTTTTTTTGGAGTTTTTTTTTATGGGTCCGGGGGGATTTTAAGGAGAGGAG-AAAAAAAAACTCTTCCGGAGTTTAGAATAAAAGTGGGATATTTTTCTAGGAGTTTATTTGTATGCGTCTGGGGGGATTAGAATGAGAGTAAAAATAAATCTTTAGGAGTTTAGAATGAGAGAAAAAGTTTTAAAATGGTTCTTCGTATTTGAATTAGTTAAAAATCAATAAAATTTTTATTTAAAGAATTTTTTTTTGTGTTTGTTATTGTAATGGTTCTTATTTATTTAATTGTTATTATGGCATTAGTTAACATCATAGGGACAGCGAGGGAGAGAAAATGTGTTTTAAAAAAGCGGGCTTTAGAGTGGTCTTTGGCTTTATTGTTTAGTACTTTAGTTTTTTGGGGTGGATTTGACGGAGAAAGTCATTTTCAATTTTTTAGTTTAGTAGAATGAAATATATTTTCTACTTTAGATTGAGGCCCGATTGTTTTTGCAGTTGATGGTGTTTCTTTGGTTTTTTTACTTTTAACGACTTTTTTAATTCCAATTTGTATTTTAATTAGCCAAAAATCAATAAAATTCTTATTTAAAGAATTTCTTTTGTGCTTGTTTTTTTTAGAATTTTTATTAATAGGTGTTTTCATAGTGTTTGATCTTCTTTTGTTTTATCTTTTTTTTGAGGGGATATTAATACCAATGTTTTTTTTAATTGGTATTTGAGGCTCTCGAGAAGAAAAGGTTCGTGCTTCTTTTTATTTTTTTTTTTTTACTTTTATAGGCTCTCTCTTTTTCTTTTTTATAATACTTTTTTTATATCAAAGGATTGGAACAACAGACTATTTTCTTTTACTTAATATTAAATTATTTTTAAATATTCAAAAATGAGCCTTAGTCGGGGTTTTTCTTAGTTTTGCGGTGAAACTACCTCTTATTCCATTTCATATTTGATTGCCACAAGCACATGTTGAGGCTCCTGTTGCGGGCTCTGTTATTTTGGCTGGAATTTTATTAAAATTAGGGGGTTATGGCCTTTTGCGTTTTTCTTGACCTCTTTTTCCGGGGGCTTCTTTATATTGGTCTCCAGTTATTGTTTTTTTTAGTGTTGTTGCTGTTGTTTATGGAGGTTTAATGACATGTCGTCAAATTGATTTTAAACGGCTTGTTGCTTACTCTTCTGTTGCTCATATGGGACTTGTGCCTTTGGGTCTTTTTACACATGTTATAGAGGGGTTAATTGGGGCTCTTTTTTTAATGTTGGCGCACGGATTTGTTAGCTCTGCTCTTTTTATTGGAGTAACTTTTTTGTATGATCGCCATCATACTCGTTTAATAAAATATTATCGGGGTTTGACTTTGACAATGCCTCTTTTTGTTATTACAATGTTAATTTTGTCTTTGGCGAACATGGGTTTTCCTCTTAGTTGTAATTTTGTTGGAGAGTTTTTTTCTTTATTAGCAGTCTTTCAATATCATTATGGAGTTGGAATGTTTGTTATTTTAGGGGTTCTTTTTTCTGCCATTTATTCTCTTAGTCTTTTTAATCGTATTTCTTTTGGTGGAGGCTCTAATTATTTACTTTTTAATAGAGACTTAAGTCGACGAGAGATTTTTGTAATTTTTCCTTTTCTTTTAATTATCTTTTTGGGGGGGATTGTGCCTTTTCCTATTATTGATTTAATTAAAAATAGTCTTGTTTTTAGCCCGGTTAGTTAG-------------TCCTGGGGTTTTAGGGATAAAAAAATATTTAGTCTAGGTTATACATGCTAGTGTATGCGAACAGGTGAGGATAA-AATAAAAAAATTTTTTTTATTTGTTGTATTAAAAAAAGAAAAGTTTTTTA-TTCTTTTTTTGAAGATGCATAGTTTAACCACATTTTCACTGAAACAAAAGAAAACCCTTGGCAGCAGTAATGAATTTTATGTAATATACGAAAGTAAGACATAGGTAGGATAAAAAAAATCTGTCAAAT-TAAGTGCCAGCCGACGCGGTAAGACTTAAGGATTTA-TTTTTTAATAAAAAGCAAAAAGCGTGTTAAGGATTTTTTAAAAAAAAAAATAAATAGAATTTTTTTC--GTAATTGTAATATGTTAAAATGAAAAAAAGAATTTTTTATATGAAGATAATTTA------TTTTTTTTTTCTTAAATACGAAGGTTTGGGGAGCAAATAGGATTAGGTACCCTGGTAGTCTATACAGTAAAATAATATCGCTTGAGTAGTACGGTCGCAAGATTGAAATTCAAAAGACTTGGCTGTTC-ATTGTTTATTAGAGGAGCGCGTCGCTTAATTCGATTATCCGCGAGAGACCTTACCGAAGTTTGAATTTATTAACAGGTGTTGCATGGCCGTCGTCAATTTCTGTTTGAGACAAAAAGGGGTTTAATCCTATCAAGGTTGAAGTCCGGTATTATGACCCTTATATTTCGGGGTTAGGCGCGCTACATTTCTCTCTTAGAAAAGGAGGGGTTCGGATTGTTTTTAAAATAAGATAATGAAGTTGAATTTAATAGTAATTGAAAAGTAGTGCGTTTCAATGAATGTTATTGCAATGTTAGTACAAATAGTCCGTCGCCAGCTTAGAAATAGGCTGTAAGTCGTAACATAGTAAGAGTGAGGGAACTGGCTCTTGATGCAGTTTCATTCGTATCATTTAGTTGAGCCTTCTCCATGGCCTTTTGTTGGAGCAATTGGCTCTTTTTTTATTACTGTTGGTGCAGTGGTTTTTTTTCATTATGGGTTTAGTTTTTTTTTATATTTGGGGCTTTTGGTTGTTGTTGGAGTTATGTTTGTTTGATGACAAGATGTTATACGAGAATCAACTTTTCAAGGTCACCATTCTTTAATTGTAAAACAAGGGATTAAATATGGTATGATTTTATTTATTCTTTCAGAAATTTTGTTTTTTTTTTCTTTTTTTTGGGCTTTTTTTCATAGTAGTTTAGCACCGGTTGTTGAACTTGGAGTCGTTTGGCCTCCACAAGGAATTGTTGCATTAAATCCCTTTTCTGTTCCTTTATTAAATACTGCTGTATTATTAAGTTCTGGGGCAACGGTTACATGGGCGCATCATGCAATACTTTGTGGTTTAAAAAAAGAGGCTCAATTTGCTTTATTTTTAACTCTTTTTTTGGGTGTTATGTTTACGGGGTTGCAAGCATTTGAATATTATGAGGCGCCTTTTACTTTATCTGACTCTGTTTATGGGGCCACTTTTTTTGTTGCGACAGGGTTTCATGGACTACATGTTATAATTGGTACGACTTTTCTTTTTATTTGTTTTTTACGTTTACTCTCTAATCAATTTACTCGTCGTCAACATGTTGGGTTTGAAGCCGCGAGTTGATACTGGCACTTTGTCGATGTGGTTTGATTATTTTTATATTTATGTATTTATTGATGAGGCTCATAAAAATTTCTTTAATGCAAAATCTTTTTGTTTTTACTTTTTTTAGATATTTTATAAATGTTTTGGCTGGGTTGTGTGTTCTCTGGTTTAATCATCTTTATTATTCGGATAGTCCAGAGACTTGACTTTTAGAGTTTCAAGATGTTGGGGATCCTATTGTAGAAGAAATTGTTTTTTTTCATGACCAAGTTATGTTTTTATTAATTATTATAGTTACTGTTGTTTTATGACTTTTTGTTGAAGCTTTTAAAAATAAGTTTTATGATCGTCATTTAATCGATGGTACATTTTTAGAAATTGTTTGAACGATTATTCCTGCTGTTATATTGATTTTTATTGCATTGCCCTCTCTTAAATTATTATATTTAATGGATGAGGTTATTTCTCCAGCTTTGACAATTAAAGTTATTGGGCATCAATGATATTGATCCTATGAATATTCTGATTATGAAGGGGATACGTTAGGGTTTGATTCTTATATGATTCCGACTTCGGATTTAGTTTCTGGAGAGAATCGTTTGTTAGAGGTTGATTATAAACTTTTAATCCCTATTCAAACACATATAAGATTTTTAGTTACTGGGGCAGATGTTTTACATTCTTTTGCAGTGCCTTCTTTAGGGCTAAAAATTGATGCGGTTCCGGGTCGTCTAAATCAAACTGGTGTTTTTATAAAACGAGCCGGGGTTTTTTTTGGACAATGTTCTGAAATTTGTGGGGCAAATCATTCTTTTATGCCCATTGTGATAAAGGGAGTTGGTTTAAATGAATACGTTCAATATTTAAATTATTTAAAATGTATTATAAATACTTAGTTATTATTATTATTTTATTTTTATTGGGGAGTTGGGGTATAATTTTAAATAGAGGACATTTTATTATTATGCTTGTTTCTATTGAATTAGTTTTATTATCAACTTTTTTTTTTTTTTTAATAAGTTCTAAAGAAATAGATCTTTTAATAGAACAAATTTTTATGATTATGGGGTTAACTATTGCTGCTGCCGAATCTTCAATTGGTTTAGCTATTTTAGTTGCATATTATAGAATTCGAGGAACAATTGTTTTAAAATCTTTTAGTTCTTTGCGAGGATAATGATTAATCTTTTTATTTTTTTTTTTCTTATTTTTATTTTGGCGGGGTTATTTATATTTCTTTCTTTTTTGATAGGGGAAAAAATTCCAGATCGAGAAAAGGTTTCTGCTTATGAGTGTGGTTTTGCTCCCTTTAATTTTTTAGGACGCCCTTTTTCAATACGTTTTTTTTTAATTGGTATTTTATTCTTGATTTTTGATTTAGAAATTTCTTTTTTTTTTCCCTGATGTGTTTTATATAATTCGACTGCTCCGTTTGGGTTTTGAACTATGATAGGTTTTTTTTTTGTATTAGTTTTGGGTTTGATATATGAATGAGTAATGGGGGGATTAGAATGAGAGTAAAAATAAAGTAAAAGAAAAAGTCCTATCTATTATGAAAATAATAGAAATTTTATACCAACTCCGGTTTCTGCTTTAATTCATGCTGCAACGATGGTTACTGCAGGTGTTTTTTTATTAATTCGTGCTTCTCCTTTATTTGATGTTGTTCCTCTTATATTAATTATTATTTCAATCGTTGGGGTTTTAACCGTGTTTATTGCAGGCACAATTGGTTTAGTTCAAAATGATTTAAAAAAAATAATCGCTTATTCTACTTGTAGTCAATTAGGTTATATGGTTGTTGCTTGTGGGCTTTCTCATTATGCAATTAGTCTTTTTCATCTTATGAATCATGCTTTTTTCAAAGCTTTATTATTTTTGAGTGCAGGGTCTCTTATTCATGCGGTAATTGATGAACAAGACATAAGAAAAATGGGGGGTTTATTATCTTTTCTTCCTTTGACTTATGTTTTTTTTCTTATAGGCTCTTTTTCTTTAATGGGGTTTCCTTTTTTAACAGGATTTTATTCAAAAGATTTAATTTTAGAATTTGCTTTTGGACAATTTTATTTAATTTTTGTGTATTGATTAGGTTGTTTTTCTGTTTTATTGACAATTATATACTCTATTCGTTTAATTTATTTAGTTTTTTTATCCAATATTAATTTAAAACGAGCAAACATTTTTTTTCTTAAAGAAGGAGAATTTTTATTTTTAATTCCTTTGGGCATATTAACTTTAGGAAGTGTTTTTTGGGGCTATTTAAGTAAAGAAATAATTTGGTCTTTTCAAATAGATGTTTTTTCAATACTTTCTTTAAAAATAAAAATATTTCCAATTTTATTTTGTTTTATTGGACTTTTTGGGACGATATTTTTTTTTTTTTTTTTTTCTTCTCAAATTTTTGGTTATCCCCTTCAGGTGATCGGATCTTCTGTTTTTTCTCTTTATAATTTTTTTGGTTCTGCTTGACAAATAAATTTTTTTTTTAATTTTTTCTTTATAAAAAAAATATATAAAATAGGACATCTTATTACTAATTTAACAATTGACAAAGGTTTATTAGAGGTTGTTGGGCCTAGGGGTGTTGTTCAATTTTTTATTTTTCAAACTCAAAAGTTGAGTAGTATACAATCGGGGTTGGTATTTAATTATGCTTTAGTTTTTTTTCTTGGGATACTTTTTTTAATTTTTGCACTTTAATTAAAATTTTTGAAGGGGTTAAGTTAAGCAAAACAGTTGACTTTCAAAGTTAATTATGTAGGTGGAAATCTTACACTCTTTGGATGCCACAGTTAGAGGTAGGTACTTATAATATACAATATTGGTGGGGGTTCTCGGTTTTATTTTTCTTGTTAATTTTTTTAGAAGTTATAATCTTTCCTTTTGTAAAGCGTGGTTGATGAATAAGACAGTTCTTATTGGGGTG---------------AACCTTTTTTTTAGGGTGAAAAAAAAATTATGGAAAAAGATTTCCATGAGAACTGTCGCAAAATGTTTAAAAAAAGCCTTT-TTTTTGCT--AGTGGGATTTTGTTTAAAATATTTTT-TTGAGTTTTCTTACTTAAAAACATCTTTTTGTTTAATGA-TTTTTTTTTTTTTTAT-------------------------------------AAAATTTTGT------------TCTAAGAAGATTC----------------------------------------------GGGCTCGTTTAGCGATCTTTTC---ACCGTTGATC-TTT---GGGGATGGTGTGGA--AAAAATGGAWGW------G-CCAGATGA--AGAGATAGTAGGAG--CGTTTCCATCTTTA--------AGCTTCGTGGAGGGGTGAGATG----------------------------AAGAGGTGATAGCAGCGTTTCCATGTTCAGACT----------CCGAAGAAAGTATGGAAATTGATGGGGAAGA--ACTACCTTTGGAGCCCCTCCCGATTAGTGGAGCT--ATTAGAGGAGAA--AACATAGTGGCA-------------------------------CCCGTACCAGTCATGGGAGATGCGGGTCTTTCAGTTCATTTTTGGCGTGCACTCAAGAACACTCCATTTGAATTAT-TGGCAGAAGTTTATCGGTCAGGTGAGGAGGGAGAAG--TT---T--ATTCGTCAGATGAG---GAAGACGAGTTAGTAGAGGTGTTACTTCAGTACGGAAA-AGAGAT---------------------AACATCAAAAGAGTCAGAGGACAAATTTTTGAGTGGGCTAAT--AGT--GGAAATGGTGGAAAACGTAATTGTCT-AAGATAAATACCA-GAA-CAGTT-AGTAAACAGATTATTGAGTGGGTCAGTGGA--CAAAGTAGGGGATAACGTGGTTGGGTAAAAT-ATAGTGTCGGCGGCAACGGAAGTGGGGAGGGGGAGAGCTCTCTTGCTCTCTAATTTGTAGTTTTTGAGTGGTTTGTATTATTTTTT--TTTTTAATTTTTTCTTTATGAGTCTGTCTTGGTAGAGGAG-GCGGTTTTTTTAGAAGCCCCGTCTAAATGCTTTCTAGTTATTGAACTCGATGGGTGTTTTCTACAAATCATAAGGACATTGGTACTTTGTATTTAATCTTCGGAGCAGGAGCTGGTTTAATTGGAACTGCTTTTAGTATGCTTATACGATTGGAGCTTTCTGCGCCGGGGGCGATGTTAGGAGATGATCATCTTTATAATGTAATTGTTACAGCACATGCTTTTATTATGATTTTTTTTTTGGTTATGCCGGTCATGATTGGGGGATTTGGTAATTGATTAGTCCCATTATATATTGGGGCGCCGGATATGGCGTTTCCTCGATTAAACAATATTAGTTTTTGACTTTTGCCTCCTGCGCTTTTTTTATTATTAGGCTCTGCTTTTATTGAACAAGGGGCGGGGACGGGGTGAACAGTTTATCCTCCTCTTTCTAGTATTCAAGCACACTCCGGAGGTTCTGTTGATATGGTTATTTTTAGTCTTCATTTAGCTGGGGTTTCTTCTATTTTAGGTGCTATTAACTTTATTACTACAATTTTTAATATGCGAGCCCCGGGTGTGTCTTTTAATAAACTACCTTTATTTGTTTGATCTATTTTAATAACAGCTTTTTTATTACTTTTATCTTTACCTGTTTTAGCTGGTGCTATTACTATGTTGTTAACAGATAGAAACTTTAATACGACTTTTTTCGATCCAGCGGGTGGCGGGGACCCAATATTATTTCAGCATTTATTTTGATTCTTTGGGCATCCAGAAGTTTATATTTTAATTTTGCCTGGTTTTGGTATGATTTCTCAAATAATCCCGACTTTTGTTGCTAAAAAACAAGTTTTTGGGTATTTAGGAATGGTTTATGCCATGCTTTCTATTGGGCTTCTTGGATTTATTGTTTGAGCTCATCATATGTTTACTGTTGGGATGGATGTAGATACAAGAGCATATTTTACTGCTGCTACTATGATTATTGCTGTGCCAACTGGGATTAAAGTTTTTAGTTGGTTGGCAACTATTTATGGAGGTGTTCTTAGGTTAGAGACTCCAATGCTTTGAGCTATGGGGTTTGTTTTTTTATTTACAGTTGGTGGTTTAACTGGGGTTGTGTTAGCAAATAGTTCTCTTGATATTGTTCTACATGATACATATTATGTAGTTGCGCATTTTCATTATGTTCTTTCTATGGGGGCTGTTTTTGCTATTTTTGCGGGGTTTTACTATTGAATTGGCAAAATAAGTGGTTATTGTTATAATGAATTTTTTGGGAAAGTTCATTTTTGATTAATGTTTATCGGGGTTAATCTAACTTTTTTCCCTCAACATTTTTTAGGTTTAGCAGGATTTCCAAGACGATATTCGGATTATGCAGATGCTTTTTTGGGTTGAAATTTAATAAGTTCTTTAGGGTCTATTATTTCTATTTTGAGTGTTGTTTGGTTTTTATATATTGTTTTTGATCTTTTTGTTACAGAAGAAAAATTTTTGGGTTGAAAAGAAGGATTTTCTTTAGAATGAATTCATTCTTCTCCCCCCTTATTTCATACTTATGAGGAGTTGCCCTTTGTACAAAAAGTAAATAATTTTTAGAATAGTGCCGGGTTTATATACCGGTTT

SD3 TGTAAGAAAGACAAAGGGTTAGTCATTGGGTTCATGCCCCAACCAAGTGAGTTCGAATCTCTCTCTTACACAAAATAAAAATTAT-AAAAAAAAAAGAGGATAACTAAGATAAACTAAAATTAGACGATTTTTTTCGAAATGGCGTTCAGTTGCTTATTGGTTGTTTCTTCAGAAAAGTGATTTTTTTTTTTATTTGAATTGAAACATCTTATTACTAAGTAAAAATCAAACGAGATTCCGAGAGTAGTGGAGAGTGAAATTGGAGTTGTGCTTGTGGTGGTCATAGATCTTGCTTAAACATTAGTTTATACTGATAATGAAAGTACTGTAAAGGAAAGTTGAAAGAGAGTTGAAAGGAATTTGAATCTTTTATTTTTGAAGTAGCTTTAAAAAAGCGTACCTTTTGTATAATGGGTAAAAGAGATTTATTTGGCATATTTAAAATGGAAAATTTTAAGTAA-TTTTTTTTTAGTCAAATTTCCCGAAACCAAGTGATTTAATCATGAATTGTGTGAGCAAAAACTGGTGATTGTGGCAAAAATCTCAGAAAATTTGTGATTAGGGGTGAAAGGCTAATCGAACTTGGAAATAGCTGGTTTTCTGCGAAAACTATTTAAGTAGTGTTC-TTTTTTTTTTTATTTT??????????????????????????????????????????????????????????????????????????????????????????????????????-??????????????????????????????????????????AAAAATGTAGGCTTGGAACCAGCCATCTTTTAAAAAGTACGTAG????????????????????????????????????????????????????????????????????????????????????????????????????????????-?------------------????????????????????????????????????????????????????????????????????????????-???????????????????????????????????????????????????????????????????????????????????????????????????????????????????GTTTTTCTAATTAAAGAAAGAAAAAAAAAAAGAAAAACATTTTTTTAAGGAACTCGGCAAAGTTAAACTTCGACTGTTTACCAAAAACATAGCTTTTTGATTTTT??????????????????????????????????????????????????????????????????????????????????????????????????????????????????GTCAATTAATTGTTGACCGGTATGAATGGTATCCCGAAAGTTTTTCTGTCTTAAAAAAATATTCAATGAAATTAAATCTGTAGTGAAGATGCTACATTTTAATTGTTAGACGAGAAGTCCCCATGGAGCTTTACTGTAAGCTTATATATATAATTTTTTTTTTTTTATAAGTAAGACAGTTTTGTTGGGGCGACAGTTTTTTAAAAAGTAACGAAAATGAGCTATGACGCATGTTTAACTTTGAAATTTTTTT-ATTGATGAGACATTTTTGGTGTGTTTTTTGATCCGTTATTTTGAATGAAAAAAATAACGAAAACAAATAAAAGTTACCCTGGGGATAACAGCGCAATAATGTTTGAGAGTTAATCAACGACAGTGTTTGCGACCTCGATGTTGAATTGTAACGTCCTACGGTGTAGTCACTCGTAAGGGTTGGTTTGTTCATCCATTAAAGTTATACATGATTTGAGTTAAAAGCGTGGTGACACAGCTTGGTTTCTATCTACAATTAGAAAACAAAAATATGTTGTTTTTTCGTACGAAAGGATCAAAAATCAATAGTTCTCTTAATATAACTATTCTTTAAATTAGGATTGTTTCTAAAAATAAAAAGAAATATTTTTGTTTGTTGTTTTTATTAATGTATCTTTTAGTTTTATTTTTTCCTTTAATTGGAGCTGTTTTAACAGGTTGTTTTGGAAGAAAAATCGGAGAAAGAGGAGCGGGGATTTTAACTTCAAGTTGTTTAGTTTTTAGTTTATCTTATTCTTTTTTAATCGCGATTGAAGTTTTATTTAATTCAACAACAACGTATTTGAAATTATGAAAATGATTTGATTCGGGGTTGTTTGTTGTTTTTTTTGGTTTTCAATTTGATGGTTTAGTAGTTATTATGTTATTTGTTGTTTTTATTGTTTCTACTTTGGTTCATATTTTTTCTATTGCTTATATGCGGGGAGATCCTCATATTCCTCGATTTATGACATATCTTTCTTTGTTTACTTTTTTAATGGTTTTATTGGTAACTAGCGATAATTTTCTTCAATTATTTATTGGGTGAGAAGGGGTTGGTCTTTGTTCTTATTTATTAATTAATTTTTGATTAACTCGATTAGAGGCAAATAGAGCTGCTATTAAAGCAATGTTAGTGAATAGAATTGGTGATATTGGGTTGCTTTTAGCAATGTTTTTACTTTGGGATCTTTTTGGATCTCTAGATTTTTCTACTATTTTTAATTCTATTTTTTTTTCTAATCAAATATTTTTTATTTGTTTATTTTTATTTTTTGGGGTTATGGGAAAATCTGCTCAATTAGGATTACATACTTGGTTACCGGATGCAATGGAAGGTTATTGGGCCTTTTAATTAAAAAATTAATTAAAAAAATTACTATACATTAAAAAAACAATTTGTTAGTAAAATATTTGTTTATTAATGGACAATAAAATATTTATTTTAATGTCTAAGAGACTTTATGTGATTTACTTTTTTTTATTTGATTAAATCTATTATTATTATTATTCCTTTACTTATTGCTGTTGCATTTTTAACTTT????????????????????????????????????????????????????????????????????TTCAACCTTTTGCAGATG???????????????????????????????????????????AAATAAATTTGTTTATCTTTTKGCTCCAGTTATATCTTTTACATTAGCTTTCTTTGTTTGAGGTTTTCTTCCTTATGAGAAAGGAGTGAGTATTAGTGATTTTAAAATTAGTCTTTTATGAATTTTGGCTATTTCTTCTATTAGTGTTTATGCAATTTTAATGTCTGGATGAGGAAGTAAGTCTAAATATGCTTTTTTGGGTGCTATTCGGGCGGCGGCTCAAATGATTAGTTATGAGGTTTCAATTGGGCTAATTCTTATTTCGGTTTTATTATGTGTTGGTTCTTTAAGTGTTACTGAAATTGTTTTAGCGCAAAATAATGGTATTTGGTTTTTTTTTCCACTATTTCCTGTTACAATAATGTTTTTTGCTTCGATTTTAGCAGAGACCAATCGTGCTCCTTTTGATTTAACAGAAGGAGAGTCAGAGCTTGTTTCGGGATATAATGTTGAGTATGCTTCGATGTCTTTTGCTTTATTTTTTCTTGCTGAATATGCCCATATTATTTTAATGAGTTGTTTAACAACGATTTTTTTTGGGGGAGGATGGCTTTCTCCGATTCCAGATTTTAAGGGTGGGGCGGGCTGGTTTGGTTTTAAAGTTGTTTTTATTATTTTTTTTTTTATTTGAGTAAGGGCTTCATTTCCTCGAATTCGATATGATCAGCTTATGGCTTTGTTATGAAAAGGGTATTTGCCTTTAAGTTTAGGAATGGTCATCTTTGTGGCTAGTGTTCTCTTTGGGTTTAATGGTCCCCCTCCTATTTAAAATATTTTAATTAATTATTATGAAAAAAAAAAAGAACTAACAGATTTGCAAGTGCCATTACGAAAAAAAAATCCTATTTTATTTTTAATCAACGGATTTTTAGTTGATTTGGTTTCTCCTTCTAACATTACTTATTTATGAAATTTTGGATCTTTATTGGGATTGTGTTTGATTTTACAGATAGTGACTGGGTGTTTTTTGTCTATGCACTATTGTTCTGATGTTAATTTTGCTTTTGCTTCAATTGGTCATATTATGCGAGATGTTAATTATGGGTTTTTATTAAGATATCTTCATGCTAACGGTGCTTCTTTGTTTTTTTTTTGTCTTTATGTTCATATTGGTCGAAGTTTATATTATGGGGGGTATTTAAAATTTCATGTTTGAAGCATTGGAGTTGTTATTTTTTTATTAACAATGGCTATTGCCTTTATGGGCTATGTTTTGCCTTGAGGACAAATGTCTTTTTGAGGGGCAACTGTAATAACGAATTTATTAT????????????????????????????????CAGTGAGTGTGAGGCG?????AGTGTTTCTAATGCAACATTAAATCGGTTTTTTAGTTTACATTTTTTACTTCCTTTTATTTTAGTTTTTCTTGTTATTCTTCATTTAGTTTATTTACATGTTGATGGGTCTAATAATTCAACAGGATTAAATTCTTCAATTGACGGAGTTTCATTCCATACTTTTTATACATCAAAAGATTTTTTTGGTTTTTTTTTTCTTTTTTTTTTATTTTGTTTTTTTGTTTTTTTTTTGCCTAATTTATTAGGAGATGCTGAAAATTTTATTCAAGCGAATTCTTTGGTTACTCCGGTTCATATTCAACCAGAATGATATTTTTTATTTGCTTATGCAATATTACGTTCTATACCAAATAAATTAGGTGGGGTTATTGCAATGTTTTGTAGTATTTTTATTTTATTTTTTTTATCTATTTTACATCAAAGTCTTTTAAAGGGACTTTTTTTTCGTCCTTTAGGTCGAATTGCCTTTTGGTTTTTAATTATTGATTTTGCTTTATTAACTTGAATTGGATCACAAGTTGTAGAAGAACCTTTTATTTTAATTGGTCAAATACTTTCTTTTTTTTATTTTTTTTATTTTTTAGTTTTAATACCAGTTTTGGGTATTATTGAAAATCAATTATTAAATAAAAATTAATGAAATTTTTGTTTTAGGCGGTTTTCTTTTGAGCTTCGTGGTTTTAAGTCTTCGTTATTTTCTTTTAAAACTTTCTCTTTTTTATT???????WTTTTTTTTTTTTTTTTTTTTTAAATTAGA?????????????????????????????????????????????TTTATTTGGACCAAAAAAAGAAGAACAAACAGATGTCCCTATTTTAAGTTTAATTATTATTTTTGGAGTTTTTTGTTTAATTTCTTCAAGTAATTGACTTTCTATTTATTTAACCATTGAGCTTTTTACTCTTTGTTTTTTTATTTTGATTGCTCGAGGGTCTGGTTATAGTGTGGAAGCAGGATTAAAATATTTTATTTTGGGTGCTCTTTCTTCTGGTTTATTTTTATTTGGGTGTGCTTTATTATGTGGTATTGGGGCG??????????????????????????????????????????????????????????????????????????????????????????????????????????????????????????????????????????????????????????????????????????????????????????????????????????????????????????????????????????????????????????????????????????????????????????????????????????????????????????????????????????????????????????????????????????????????????TGGTTCTTTTGAAAGTTTACAAGCTAGTCTTGTTTAT???????????????????????????????????????????????????????????????????????????????????????????????????????????????????????????????????????????????????????????????????????????????????????????????????????????????????????????????????????????????????????????????????????????????????????????????????????????????????????????CTTTTAATTACAATTAAAGTTTTAAAAAAAGAATCTAAATTAAATTTTAAAAAAGTTTTTTTAATTGGTTTTTGTTTTTATTTTATTCTTTTTTTTTTTCTTTCTCCACATTTTCTTTTTTTTTTTTTTTCTCAAATAATTTTTGATTTATTTTAAAATGGAATTTTTTTCTTTTTTTTTTATTTTGGGGATAATTGGCTCAGGAGTAATGGTTGTTTCAGCGTTAAA?????????????????????????????????????CTCCATTCTGCCGTTTTTTTCCTTTTACTAGGAATAGATTTTCTTGCTTTGATGTTTTTACTTATTTATGTTGGGGCAATAGCTATTTTATTTTTATTTGT????????????????????????????????????????????????????????????????????????????GGATTTATAATTGGGATTTTTTTTTTTTCAGAAATTGCTTCAAGTGGATTACTTTTGGGTTCTTTTCAAATAGAGAATTGAGACCTTTCTTTTCCTTGATTTCTTATTTCTTATCATAATATTGAGGCCTTAGGGCAGATTTTATATGTTTCTTGTTTTTGTTTATTTCTTTTGGCCAGTTTTATTTTATTAGTTGCTATGATGGGTGTGATTGTATTAACTCAAGAAACAGAATCTTTAAGTAAAAAACAAGATCTTTTTTTCCAAATAAATAGGTAATGAACAGTTCTTCTTATTTTGAACAATTTAACATAGTGTGGTTGTTTGGTTTTACGAATTCAACAATAATGATGACTTTTGTAATTATTGTAGTTTTATTATTTTTTAAAGGAATTGAATTAATTCCAAAAAGATGGCAGTCGGTTTATGAATGTCTAGAAAATTATTTTTATTATATAACAGTGCAAAATTTAAGTAATGTGGGTTTAATATATTTTTCTTTTATTGTTTCCCTTTTTGTTTTTTTAGTTTTTTTAAATATTTTAGGTTTATGTCCTTATGTTTTTACTCCGACAACTCATATTATTGTTACTCTTGGGTTTTCTTTTTCTATTGTTATTGGAGTAACTTTTTCTGGATTTTATAAATTTAAAAAAGATTTTTTTAGTATTTTAATGCCT??????????????????????????????????????????????????????????????????????????????????????????????????????????????????????????????????????????????????????????????????????????????????????????????????????????????????????????????????????????????????????????????????????TTTTTTTACATTAA-----------------AAATGACCGCCGTAGAAATTTTTTTAT??????????AGGGATGTCGCTAACTGTGAATTATGGATTTGGTATTGGGTGATATAGTAGTTCTTCTCAAGCCTGGTTAAGACACCAAATGGGC---ATAGACGGTGTAGATACGGTCCATCCTTGAGCTTATTTTAATTATGTGCGCCAGCATTCTATTGCTCGTAATGTTGGGGTACAAGTGAATCTAGATTCAACTGAAGGACCAAATGATGTTGGGGTACAAGTGAATCAAGAGTCTGAACAGTCTGTGGATGTTGTTTTTATAGAATTGGAAGGGCGAAGGGTCCCCGTAAG--GGGGGTGA------------------ATCTACATGTTGAGCGGCGT-GGTTATTTGGATAATCG---GTCTAATCAAGAAGG--TGT-TGA---------------------------------------------GAATAGTTTAGGAGATCA-----ATTTGA-AGATGTGGCTTT---TTTAG-GGGGGAG-TACAAGTACAATTACACCAGAAA----GTGTAAATAGGGGCGCTCCAAGTGGTAATATAAGAAATGTATTAAGTGATTCATTAA-TAGA-G-AG-GAGAGGCCAAGACCAAACTTTAAGTGA--AA-TAAAGGTAGAAACACCTCC----AGGTGCTTCAAGGGGTGGCCGATG-A-------G--CCAGT-AT-----AAATCAAGGTGAGGCTTTAAG---AGAAAACAAGGTAGAAATTGGGG----TTTTAAGAGAAAACAAAGT--------AGAAAT--TGATGAGGTAGTTGGCTCGACTTTTAGTTCGATCCCCTTAGAGGTTGAAGGGATGATGGCTCATGTGTGAAATCAATTTGACATGGTGTTTAACATATTGGCCGATAAGCCCCTGGTAGGTTGCATTTTTTTTTCTTACCAGCTTCTAAGAATAACGAATCAAATGCCAAACCCACGGGTTTATAGGTGATTTGCTTTGGTGTATCTTTTATTTATTTTTGTTCTTTTAATGGTTTAGCTGTTTGTACGTAGTAGGATAAAAAAGTGGGACATTTTTCTAGGAGTTTATTTGTATGCGTCTGGGGGGATTAGAATAAGAGTAAAAAAAAACTCTTTAAAAGTTTACA???????????????????????????????????????????????????????????????????????????????????????????????????????????????????????????????????????????????????????????????????????????????????????????????????????????????????????????????????????????????????????????????????????????????????????????????TTAAAATGGTTCTTCGTATTTGAATTAGTTAAAAATCAATAAAATTTTTATTTAAAGAATTTTTTTTTGTGTTTGTTATTGTAATGGTTCTTATTTATTTAAT?????????????????????????ATAGGGACAGCGAGGGAGAGAAAATGTGTTTTAAAAAAGCGGGCTTTAGAGTGGTCTTTGGCTTTATTGTTTAGTACTTTAGTTTTTTGGGGTGGATTTGACGGAGAAAGTCATTTTCAATTTTTTAGTTTAGTAGAATGAAATATATTTTCTACTTTAGATTGAGGCCCGATTGTTTTTGCAGTTGATGGTGTTTCTTTGGTTTTTTTACTTTTAACGACTTTTTTAATTCCAATTTGTATTTTAATTAGCCAAAAATCAATAAAATTCTTATTTAAAGAATTTCTTTTGTGCTTGTTTTTTTTAGAATTTTTATTAATAGGTGTTTTCATAGTGTTTGATCTTCTTTTGTTTTATCTTTTTTTTGAGGGGATATTAATACCAATGTTTTTTTTAATTGGTATTTGAGGCTCTCGAGAAGAAAAGGTTCGTGCTTCTTTTTATTTTTTTTTTTTTACTTTTATAGGCTCTCTCTTTTTCTTTTTTATAATACTTTTTTTATATCAAAGGATTGGAACAACAGACTATTTTCTTTTACTTAATATTAAATTATTTTTAAATATTCAAAAATGAGCCTTAGTCGGGGTTTTTCTTAGTTTTGCGGTGAAACTACCTCT????????????????????????????????????????????????????????????????????????????????????ATTAGGGGGTTATGGCCTTTTGCGTTTTTCTTGACCTCTTTTTCCGGGGGCTTCTTTATATTGGTCTCCAGTTATTGTTTTTTTTAGTGTTGTTGCTGTTGTTTATGGAGGTTTAATGACATGTCGTCAAATTGATTTTAAACGGCTTGTTGCTTACTCTTCTGTTGCTCATATGGGACTTGTGCCTTTGGGTCTTTTTACACATGTTATAGAGGGGTTAATTGGGGCTCTTTTTTTAATGTTGGCGCACGGATTTGTTAGCTCTGCTCTTTTTATTGGAGTAACTTTTTTGTATGATCGCCATCATACTCGTTTAATAAAATATTATCGGGGTTTGACTTTGACAATGCCTCTTTTTGTTATTACAATGTTAATTTTGTCTTTGGCGAACATGGGTTTTCCTCTTAGTTGTAATTTTGTTGGAGAGTTTTTTTCTTTATTAGCAGTCTTTCAATATCATTATGGAGTTGGAATGTTTGTTATTTTAGGGGTTCTTTTTTCTGCCATTTATTCTCTTAGTCTTTTTAATCGTATTTCTTTTGGTGGAGGCTCTAATTATTTACTTTTTAATAGAGACTTAAGTCGACGAGAGATTTTTGTAATTTTTCCTTTTCTTTTAATTATCTTTTTGGGGGGGATTGTGCCTTTTCCTATTATTGATTTAATTAAAAATAGTCTTGTTT?????????????????-------------??????????????????????????????????????????????????????????????????????????-??????????????????????????????????????????????????-?????????????????????????????????????????AAACAAAAGAAAACCCTT??????????????????????????????????????????????????????????????????????-????????????????????????????????????-???????????????????????????????????????????????????????????????????--??????????????????????????????????????????????????????------????????????????????????????????????????????????????????????????????????????????????????????????????????????????????????????????-????????????????????????????????????????????????????????????????????????????????????????????????????????????????????????????????????????????????????????????????????????????????????????????????????????????????????????????????????????????????????????????????TGAAAAGTAGTGCGTTTCAATGAATGTTATTGCAATGTTAGTACAAATAGTC??????????????????????????????GTAACATAGTAAGAGTGAGGGAACTGGCTCTTGATGCAGTTTCATTCGTATCATTTAGTTGAGCCTTCTCCATGGCCTTTTGTTGGAGCAATTGGCTCTTTTTTTATTACTGTTGGTGCAGTGGTTTTTTTTCATTATGGGTTTAGTTTTTTTTTATATTTGGGGCTTTTGGTTGTTGTTGG????????????????????AAGATGTTATACGAGAATCAACTTTTCAAGGTCACCATTC??????????????????????????????????????????????CTTTCAGAAATTTTGTTTTTTTTTTCTTTTTTTTGGGCTTTTTTTCATAGTAGTTTAGCACCGGTTGTTGAACTTGGAGTCGTTTGGCCTCCACAAGGAATTGTTGCATTAAATCCCTTTTCTGTTCCTTTATTAAATACTGCTGT???ATTAAGTTCTGGGGCAACGGTTACATGGGCGCATCATGCAATACTTTGTGGTTTAAAAAAAGAGGCTCA???????????????????????????????????????????????????????????????????????????????????????????????????????????????????????????????????????????????????????????????????????TTATTTGTTTTTTACGTTTACTCTCTAATCA???????????????????????????????????????????????????????GTCGATGTGGTTTGATTATTTTT??????????????????????????CATAAAAATTTCTTTAATGCAAAATCTTTTTGTTTTTACTTTTTTTAGATATTTTATAAATGTTTTGGCTGGGTTGTGTGTTCTCTGGTTTAATCATCTTTATTATTCGGATAGTCCAGAGACTTGACTTTTAGAGTTTCAAGATGTTGGGGATCCTATTGTAGAAGAAATTGTTTTTTTTCATGACCAAGTTATGTTTTTATTAATTATTATAGTTACTGTTGTTTTATGACTTTTTGTTGAAGCTTTTAAAAATAAGTTTTATGATCGTCATTTAATCGATGGTACATTTTTAGAAATTGTTTGAACGATTATTCCTGCTGTTATATTGATTTTTATTGCATTGCCCTCTCTTAAATTATTATATTTAATGGATGAGGTTATTTCTCCAGCTTTGACAATTAAAGTTATTGGGCATCAATGATATTGATCCTATGAATATTCTGATTATGAAGGGGATACGTTAGGGTTTGATTCTTATATGATTCCGACTTCGGATTTAGTTTCTGGAGAGAATCGTTTGTTAGAGGTTGATTATAAACTTTTAATCCCTATTCAAACACATATAAGATTTTTAGTTACTGGGGCAGATGTTTTACATTCTTTTGCAGTGCCTTCTTTAGGGCTAAAAATTGAT???GTTCCGGGTCGTCTAAATCAAACTGGTGTTTTTATAAAACGAGCCGGGGTTTTTTTTGGACAATGTTCTGAAATTTGTGGGGCAAATCATTCTTTTATGCCCATTGTGATAAAGGGAGTTGGTTTAAATGAATACGTTCAATATTTAAATTATTTAAAATGTATTATAAATACTTAGTTATTATTATTATTTTATTTTTATTGGGGAGTTGGGGTATAATTTTAAATAGAGGACATTTTATTATTATGCTTGTTTCTATTGAATTAGTTTTATTATCAACTTTTTTTTTTTTTTTAATAAGTTCTAAAGAAATAGATCTTTTAATAGAACAAATTTTTATGATTATGGGGTTAACTATTGCTGCTGCCGAATCTTCAATTGGTTTAGCTATTTTAGTTGCATATTATAGAATTCGAGGAACAATTGTTTTAAAATCTTTTAGTTCTTTGCGAGGATAATGATTAATCTTTTTATTTTTTTTTTTCTTATTTTTATTTTGGCGGGGTTATTTATATTTCTTTCTTTTTTGATAGGGGAAAAAATTCCAGATCGAGAAAAGGTTTCTGCTTATGAGTGTGGTTTTGCTCCCTTTAATTTTTTAGGACGCCCTTTTTCAATACGTTTTTTTTTAATTGGTATTTTATTCTTGATTTTTGATTTAGAAATTTCTTTTTTTTTTCCCTGATGTGTTTTATATAATTCGACTGCTCCGTTTGGGTTTTGAACTATGATAGGTTTTTTTTTTGTATTAGTTTTGGGTTTGATATATGAATGAGTAATGGGGGGATTAGAATGAGAGTAAAAATAAAGTAAAAGAAAAAGTCCTATCTATTATGAAAATAATAGAAATTTTATACCAACTCCGGTTTCTGCTTTAATTCATGCTGCAACGATGGTTACTGCAGGTGTTTTTTTATTAATTCGTGCTTCTCCTTTATTTGATGTTGTTCCTCTT?????????????????????????????????????????????????????????????????????????????????????????????TATTCTACTTGTAGTCAATTAGGTTATATGGTTGTTGCTTGTGGGCTTTCTCATTATGCAATTAGTCTTTTTCATCTTATGAATCATGCTTTTTTCAAAGCTTTATTATTTTTGAGTGCAGGGTCTCTTATTCATGCGGTAATTGATGAACAAGACATAAGAAAAATGGGGGGTTTATTATCTTTTCTTCCTTTGACTTATGTTTTTTTTCTTATAGGCTCTTTTTCTTTAAT?????TTCCTTTTTTAACAGGATTTTATTCAAAAGATTTAATTTTAGAATTTGCTTTTGGACAATTTTATTTAATTTTTGTGTATTGATTAGGTTGTTTTTCTGTTT?????????????????????????????????????????????????????????????????????????????????TTCTTAAAGAAGGAGAATTTTTATTTTTAATTCCTTTGGGCATATTAACTTTAGGAAGTGTTTTTTGGGGCTATTTAAGTAAAGAAATAATTTGGTCTTTTCAAATAGATGTTTTTTCAATACTTTCTTTAAAAATAAAAATATTTCCAATTTTATTTTGTTTTATTGGACTTTTTGGGACGATATTTTTTTTTTTTTTTTTTTCTTCTCAAATTTTTGGTTATCCCCTTCAGGTGATCGGATCTTCTGTTTTTTCTCTTTATAATTTTTTTGGTTCTGCTTGACAAATAAATTTTTTTTTTAATTTTTTCTTTATAAAAAAAATATATAAAATAGGACATCTTATTACTAATTTAACAATTGACAAAGGTTTATTAGAGGTTGTTGGGCCTAGGGGTGTTGTTCAATTTTTTATTTTTCAAACTCAAAAGTTGAGTAGTATACAATCGGGGTTGGTATTTAATTATGCTTTAGTTTTTTTTCTTGGGATACTTTTTTTAATTTTTGC????????????????????????????????????????????????????????????????????????????????????????????????CAGTTAGAGGTAGGTACTTATAATATACAATATTGGTGGGGGTTCTCGGTTTTATTTTTCTTGTTAATTTTTTTAGAAGTTATAATCTTTCCTTTTGTAAAGCGTGGTTGATGAATAAGACAGTTCTTATTGGGGTG---------------AACCTTTTTTTTAGGGTGAAAAAAAAATTATGGAAAAAGATTTCCATGAGAACTGTCGCAAAATGTTTAAAAAAAGCCTTT-TTTTTGCT--AGTGGGATTTTGTTTAAAATATTTTT-TTGAGTTTTCTTACTTAAAAACATCTTTTTGTTTAATGA-TTTTTTTTTTTTTTAT-------------------------------------AAAATTTTGT------------TCTAAGAAGATTC----------------------------------------------GGGCTCGTTTAGCGATC?????---????-------???---??????????????--???????????????????-?CAGATGA--AGAGATAGTAGGAG--CGTTTCCATCTTTA--------AGCTTCGTGGAGGGGTGAGATG----------------------------AAGAGGTGATAGCAGCGTTTCCATGTTCAGACT----------CCGAAGAAAGTATGGAAATTGATGGGGAAGA--ACTACCTTTGGAGCCCCTCCCGATTAGTGGAGCT--AT??????????--?????AGTGGCA-------------------------------CCCGTACCAGTCATGGGAGATGCGGGTCTTTCAGTTCATTTTTGGCGTGCACTCAAGAACACTCCATTTGAATTAT-TGGCAGAAGTTTATCGGTCAGGTGAGGAGGGAGAAG--TT---T--ATTYGTCA??????---??????????????????????????????????????-??????---------------------??????????????????????????????????????????--???--??????????????????????????-?????????????-???-?????-??????????????????????????????--???????????????????????????????-???????????????????????????????????????????????????????TGTAGTTTTTGAGTGGTTTGTATTATTTTTT--TTTTTAATTTTTTCTTTATGAGTCTGTCTTGGTAGAGGAG-GCGGTTTTTTTAGAAGCCCCGTCTAAATGCTTTCTAGTTATTGAACTCGATGGGTGTTTTCTACAAATCATAAGGACATTGGTACTTTGTATTTAATCTTCGGAGCAGGAGCTGGTTTAATTGGAACTGCTTTTAGTATGCTTATACGATTGGAGCTTTCTGCGCCGGGGGCGATGTTAGGAGATGATCATCTTTATAATGTAATTGTTACAGCACATGCTTTTATTATGATTTTTTTTTTGGTTATGCCGGTCATGATTGGGGGATTTGGTAATTGATTAGTCCCATTATATATTGGGGCGCCGGATATGGCGTTTCCTCGATTAAACAATATTAGTTTTTGACTTTTGCCTCCTGCGCTTTTTTTATTATTAGGCTCTGCTTTTATTGAACAAGGGGCGGGGACGGGGTGAACAGTTTATCCTCCTCTTTCTAGTATTCAAGCACACTCCGGAGGTTCTGTTGATATGGTTATTTTTAGTCTTCATTTAGCTGGGGTTTCTTCTATTTTAGGTGCTATTAACTTTATTACTACAATTTTTAATATGCGAGCCCCGGGTGTGTCTTTTAATAAACTACCTTTATTTATCTGATCTATTTTAATAACAGCTTTTTTATTACTTTTATCTTTACCTGTTTTAGCTGGTGCTATTACTATGTTGTTAACAGATAGAAACTTTAATACGACTTTTTTCGATCAGATGGGTGGCGGGGACCCAATATTATTTCAGCATTTATTTTGATTCTTTGGGCATCCAGAAGTTTATATTTTAATTTTGCCTGGTTTTGGTATGATTTCTCAAATAATCCCGACTTTTGTTGCTAAAAAACAAGTTTTTGGGTATTTAGGAATGGTTTATGCCATGCTTTCTATTGGGCTTCTTGGATTTATTGTTTGAGCTCATCATATGTTTACTGTTGGGATGGATGTAGATACAAGAGCATATTTTACTGCTGCTACTATGATTATTGCTGTGCCAACTGGGATTAAAGTTTTTAGTTGGTTGGCAACTATTTATGGAGGTGTTCTTAGGT???????????????????????ATGGGGTTTGTTTTTTTATTTACAGTTGGTGGTTTAACTGGGGTTGTGTTAGCAAATAGTTCTCTTGATATTGTTCTACATGATACATATTATGTAGTTGCGCATTTTCATT?????????????????????????????ATTTTTGCGGGGTTTTACTATTGAATTGGCAAAATAAGTGGTTATTGTTATAATGAATTTTTTGGGAAAGTTCATTTTTGATTAATGTTTATCGGGGTTAATCTAACTTTTTTCCCTCAACATTTTTTAGGTTTAGCAGGATTTCCAAGACGATATTCGGATTATGCAGATGCTTTTTTGGGTTGAAATTTAATAAGTTCTTTAGGGTCTATTATTTCTATTTTGAGTGTTGTTTGGTTTTTATATATTGTTTTTGATCTTTTTGTTACAGAAGAAAAATTTTTGGGTTGAAAAGAAGGATTTTCTTTAGAATGAATTCATTCTTCTCCCCCCTTATTTCATACTTATGAGGAGTTGCCCTTTGTACAAAAAGTAAATAATTTTTAGAATAGTGCCGGGTTTATATACCGGTTT

J001 TGTAAGAAAGACAAAG?????GTCATTGGGTTCATGCCCCAACCAAGTGAGTTCGAATCTCTCTCTTACACAAAATAAAAATTAT-AAAAAAAAAAGAGGATAACTAAGATAAACTAAAATTAGACGATTTTTTTCGAAATGGCGTTCAGTTGCTTATTGGTTGTTTCTTCAGAAAAGTGA-TTTTTTTTTTATTTGAATTGAAACATCTTATTACTAAGTAAAAATCAAACGAGATTCCGAGAGTAGTGGAGAGTGAAATTGGAGTTGTGCTTGTGGTGGTCATAGATC?TGCTTAAACATTAGTTTATACTGATAATGAAAGTACTGTAAAGGA?????????????????????????????????????????????????????????????????????????????????????????????????????????????????AAATTTTAAGTAA-TTTTTTTTTAGTCAAATTTCCCGAAACCAAGTGATTTAATCATGAATTGTGTGAGCAAAAACTGGTGATTGTGGCAAAAATCTCAGAAAATTTGTGAK???????????????????????????????????????????????????????????????????-??????????????????????????????????????????????????????????????????????????????????????????????????????????????????????-??????????????????????????????????????????????????????????????????????????????????????????????????????????????????????????????????????????????????????????????????????????????????????????????????-?------------------????????????????????????????????????????????????????????????????????????????-??????????????????????????????????????????????????????????????????????????????????????????????????????????????????????????????????????????????????????????????????????????????????????????????????????????????????????????????????????????????????????????????????????????????????????????????????????????????????????????????????????????????????????????????????????????????????????????????????????????????AATGAAATTAAATCTGTAGTGAAGATGCTACATTTTAATTGTTAGACGAGAAGTCCCCATGGAGCTTTACTGTAAGCTTATATATATAATTTTTTTTTTTTTATAAGTAAGACAGTTTTGTTGGGGCGACAGTTTTTTAAAAAGTAACGAAAATGAGCTATGACGCATGTTTAACTTTGAAATTTTTTT-ATTGATGAGACATTTTTGGTGTGTTTTTTGATCCGTTATTTTGAATGAAAAAAATAACGAAAACAAATAAAAGTTACCCTGGGGATAACAGCGCAATAATGTTTGAGAGTTAATCAACGACAGTGTTTGCGACCTCGATGTTGAATTGTAACGTCCTACGGTGTAGTCACTCGTAAGGGTTGGTTTGTTCATCCATTAAAGTTATACATGATTTGAGTTAAAAGCGTGGTGACACAGCTTGGTTTCTATCTACAATTAGAAAACAAAAATATGTTGTTTTTTCGTACGAAAGGATCAAAAATCAATAGTTCTCTTAATATAACTATTCTTTAAATTAGGATTGTTTCTAAAAATAAAAAGAAATATTTTTGTTTGTTGTTTTTATTAATGTATCTTTTAGTTTTATTTTTTCCTTTAATTGGAGCTGTTTTAACAGGTTGTTTTGGAAGAAAAATCGGAGAAAGAGGAGCGGGGATTTTAACTTCAAGTTGTTTAGTTTTTAGTTTATCTTATTCTTTTTTAATCGCGATTGAAGTTTTATTTAATTCAACAACAACGTATTTGAAATTATGAAAATGATTTGATTCGGGGTTGTTTGTTGTTTTTTTTGGTTTTCAATTTGATGGTTTAGTAGTTATTATGTTATTTGTTGTTTTTATTGTTTCTACTTTGGTTCATATTTTTTCTATTGCTTATATG??????GATCCTCATATTCCTCGATTTATGACATATCTTTCTTTGTTTACTTTTTTAATGGTTTTATTGGTAACTAGCGATAATTTTCTTCAATTATTTATTGGGTGAGAAGGGGTTGGTCTTTGTTCTTATTTATTAATTAATTTTTGATTAACTCGATTAGAGGCAAATAGAGCTGCTATTAAAGCAATGTTAGTGAATAGAATTGGTGATATTGGGTTGCTTTTAGCAATGTTTTTACTTTGGGATC??????GAT??????????????????????????????????????????????????????????????????????????????GGGTTATGGGAAAATCTGCTCAATTAGGATTACATACTTGGTTACCGGATGCAATGGA???????????????????????????????????????????????????????????????????????????????????????????????????????????????????????????????????????????????????????????????????????????????????????????????????????????????????????????????????????????????????????????????????????????????????????????????????????????????????????????????????????????????????????????GTTTATCTTTTGGCTCCAGTTATATCTTTTACATTAGCT????????????????????????????????????????GTATTAGTGATTTTAAAATTAGTCTTTTATGAATTTTGGCTATTTCTTCTATTAGTGTTTATGCAATTTTAATGTCTGGATGA????????????????????????????????????????????????????????????????????????????????????????????????????????????????CTTTAAGTGTTACTGAAATTGTTTTAGCGCAAAATAATGGTATTTGGTTTTTTTTTCCACTATTTCCTGTTACAATAATGTTTTTTGCTTCGATTTTAGCAGAGACCAATCGTGCTCCTTTTGATTTAACAGAAGGAGAGTCAGAGCTTGTTTCGGGATATAATGTTGAGTATGCTTCGATGTCTTTTGCTTTATTTTTTCTTGCTGAATATGCCCATATTATTTTAATGAGTTGTTTAACAACGATTTTTTTTGGGGGAGGATGGCTTTCTCCGATTCCAGATTTTAAGGGTGGGGCGGGCTGGTTTGGTTTTAAAGTTGTTTTTATTATTTTTTTTTTTATTTGAGTAAGGGCTTCATTTCCTCGAATTCGATATGATCAGCTTATGGCTTTGTTATGAAAAGGGTATTTGCCTTTAAGTTTAGGAATGGTCATCTTTGTGGCTA????TCTCTTT?????????????????TCCTATTTAAAATATTTTAATTAATTATTATGAAAAAAAAAAAGAACTAACAGATTTGCAAGTGCCATTACGAAAAAAAAATCCTATTTTATTTTTAATCAACGGATTTTTAGTTGATTTGGTTTCTCCTTCTAACATTACTTATTTATGAAATTTTGGATCTTTATTGGGATTGTGTTTGATTTTACAGATAGTGACTGGGTGTTTTTTGTCTATGCACTATTGTTCTGATGTTAATTTTGCTTTTGCTTCAATTGGTCATATTATGCGAGATGTTAATTATGGGTTTTTATTAAGATATCTTCATGCTAACGGTGCTTCTTTGTTTTTTTTTTGTCTTTATGTTCATATTGGTCGAAGTTTATATTATGGGGGGTATTTAAAATTTCATGTTTGAAGCATTGGAGTTGTTATTTTTTTATTAACAATGGCTATTGCCTTTATGGGCTATGTTTTGCCTTGAGGACAAATGTCTTTTTGAGGGGCAACTGTAATAACGAATTTATTATCTGCTATTCCTTATTTTGGGATTGATATTGTTCAGT???????????????????????????????????????????????????????????????????????????????????????????????????????????????????????????????????????????????????????????????????????????????????????????????????ATTTTTTTGGTTTTTTTTTTCTTTTTTTTTTATTTTGTTTTTTTGTTTTTTTTTTGCCTAATTTATTAGGAGATGCTGAAAATTTTATTCAAGCGAATTCTTTGGTTACTCCGGTTCATATTCAACCAG??????AT??????????????????????????????????????????TTAGGT???????????????TTTTGTAGTATTTTTATTTTATTTTTTTTATCTATTTTACATCAAAGTCTTTTAAA????????TTTTTCGTCCTTTAGGTCGAATTGCCTTTTGGTTTTTA??????????????????????????????????????????????????????????????AATTGGTCAAATACTTTCTTTTTTTTATTTTTTTTATTTTTTAGTTTTAATACCAGTTTTGG???????????????????????????????????????????????????????????????????????????????????????????????????????????????????????????????????????????????????????????????????????????????????????????????????????????????????????????????????????????????????????????????????????????????????????????????????????????????????????????????TTGAGCTTTTTACTCTTTGTTTTTTTATTTTKATTGCTCGAGGGTCTGGTTATAGTGTGGAAGCAGGATTAAAATATTTTATTTTGGGTGCTCTTTCTTCTGGTTTATTTTTATTTGGGTGTGCTTTATTATGTGGTATTGGGGCGAATATACACTTTTCTCATATAGAACTTCTTTTTAATTCAAAACAAATTTTTTCTGCTGTTTCGATACCAATTGGGTATCTTTTAATTATTGTTGCTCTTTTTTTTAAATTATCAGTTGCTCCTTTTCATATG???????????????????????????????????????????????????????????????????????GTTTTTTTCTCTTATAATTTCAATTGGATTGCCTGTAAATTWTTKTT??????????????????????????????????????????????????????????????????????????????????????????????????????????????????????????????????????????????????????????????????????????????????????????????????????????????????????????????????????????????????????????????????????????????????????????????????????????????????????????????????????????????????????????????????????????????????????????????????????????????????????????????????????????????????????????????????????????????????????????????????????????????????????????????????????????????????????????????????????????????????????????????????????????????????????????????????????????????????????????????????????????????????????????????????????????????????????????????????????????????????????????TTCTTGTTTTTATAAATTCTGCCGTTTTTTTCCTTTTACTAGGAATAGATTTTCTTGCTTTGATGTTTTTACTTATTTATGTTGGGGCAATAGCTATTTTATTTTTATTTGTTATTATGTTATTAAATCTAACTGACTACCCTCCTGT???????????????????????????TTATATACCAATTGGATTTATAATTG??????????????????????????TCAAGTGGATTACTTTTG??????TTTCAAATAGAGAA???????????????TCCTTGATTTCTTATTTCTTATCATAATATTGAGGCCTTAGGGCAGATTTTATATGTTTCTTGTTTTTGTTTATTTCTTTTGGCCAGTTTTATTTTATTAGTTGCTATGATGGGTGTGATTGTATTAACTCAAGAAACAGAATCTTTAAGTAAAAAACAAGAT?????????????????????????????????????????????????????????????????????????????????????????????????????????????????????????????????????????????????????????????????????????????????????????????????????????????????????????????????????????????????????????????????????????????????????????????????????????????????????????????????????????????????????????????????????????????????????????????????????????????????????????????????????????????????????????????????????????????????????????????GTTATATTTCTAGGGCTGTTTCTTTGGGGATTCGTTTAGCAGCAAATCT??????????????????????????????????????????????????????????????????????????????????????????????????????????????????????TATGTGTTTTGTCTGTTGGTACAAATTTAT?????????????????????CATTAA-----------------AAATGACAGCCGTAGAAATTTTTTTATGCACAATTATAGGTATATCGTTAACAGTGAATTATGGATTTGGTATTGGGTGATATAGTAGCTCTTCTCAAGCCTGGTTAAGGCACCAAATGGGC---ATAGACGGTACAGATATGGTCCATCCTTGAGCTTATTTTAATTATGTGCGCCAGCATTCTATTGCTCGTAATGTTGGGGTACAAGTGAATCTAGATTCAACTGAAGGACCAAATGATGTTGGGGTACAAGTGAATCAAGAGTCTGAACAGTCTGTGGATGTTGTTTTTATAGAATTGGAAGGGCGAAGGGTCCCCGTAAG--GGGGGTGA------------------ATCTACATGTTGAGCGGCGT-GGTTATTTGGATAATCG---GTCTAATCAAGAAGG--TGT-TGA---------------------------------------------GAATAGTTTAGGAGATCA-----ATTTGA-AGATGTGGCTTT---TTTAG-GGGGGAG-TACAAGTACACTTACACCAGAAA----GTGTAAATAGGGGAGCTCCAAGTGGTAATATAAGAAATGTATTAAGTGATTCATTAA-TAGA-G-AG-GAGAGGCCAAGACCAAACTTTAAGTGA--AA-TAAAGGTAGAAACACCTCC----AGGTGCTTCAAGGGGTGGCCGATG-A-------G--CCAGT-AT-----AAATCAAGGTGAGGCTTTAAG---AGAAAACAAGGTAGAAATTGGGG----TTTTAAGAGAAAACAAAGT--------AGAAAT--TGATGAGGTAGTTGGCTCGACTTTTAGTTCGATCCCCTTAGAGGTTGAAGGGATGATGGCTCATGTGTGAAATCAATTTGACATGGTGTTTAACATATTGGCCGATAAGCCCCTGGTAGGTTGCATTTTTTTTTCTTACCAGCTTCTAAGAATAACG????????????????????GGTTTATAGGTGATTTGCTTTGGTGTATCTTTTATTTATTTTTGTTCTTTTAATGGTTTAGCTGTTTGTACGTAGTAGGATAAAAAAGTGGGACATTTTTCTAGGAGTTTATTTGTA???????????????????????????????????????????????????????????????????????????????????TTTTTATGGGTCCGGGAGGATTTTAAGGAGAGGAGAAAAAAAAA?????????????????????????????????????????????????????????????????????????????????????????????????????????????????????????????????????????KTATTTGTATGCGTCTGGGGGGA??????????????????????????????????????????????????????????????????????????????????????????????????????????????????????????????????????????????????????????????????????????????????????????????????????????????????????????????????????????KTCTTTGGCTTTATTGTTTAGTACTTTAGTTTTTTGGGGTGGATTTGACGGAGAAAGTCATTTTCAATTTTTTAGTTTAGTAGAATGAAATATATTTTCTACTTTAGATTGAGGCCCGATTGTTTTTGCAGTTGATGGTGTTTCTTTGGTTTTTTTACTTTTAACGACTTTTTTAATTCCAATTTGTATTTTAATTAGCCAAAAATCAATAAAATTCTTATTTAAAGAATTTCTTTTGTGCTTGTTTTTTTTAGAATTTTTATTAATAGGTGTTTTCATAGTGTTTGATCTTCTTTTGTTTTATCTTTTTTTTGAGGGGATATTAATACCAATGTTTTTTTTAATTGGTATTTGAGGCTCTCGAGAAGAAAAGGTTCGTGCTTCTTTTT??????????????????????????????????????????????????????????????????????????????????????????????????????????ATTATTTTTAAATATTC????????????????????????????????????????????????????????????TTCATATTTGATTGCCACAAGCACATGTTGAGGCTCCTGTTGCGGGCTCTGTTATTTTGGCTGGAATTTTATTAAAATTAGGGGGTTATGGCCTTTTGCGTTTTTCTTGACCTCTTTTTCCGGGGGCTTCTTTATATTGGTCTCCAGTTATTGTTTTTTTTAGTGTTGTTGCTGTTGTTTATGGAGGTTTAATGACATGTCGTCAAATTGATTTTAAACGGCTTGTTGCTTACTCTTCTGTTGCTCATATGGGACTTGTGCCTTTGGGTCTTTTTACACATGTTATAGAGGGGTTAATTGGGGCTCTTTTTTTAATGTTGGCGCACGGATTTGTTAGCTCTGCTCTTTTTATTGGAGTAACTTTTTTGTATGATCGCCATCATACTCGTTTAATAAAATATTATCGGGGTTTGACTTTGACAATGCCTCTTTTTGTTATTACAATGTTAATTTTGTCTTTGGCGAACATGGGTTTTCCTCTTAGTTGTAATTTTGTTGGAGAGTTTTTTTCTTTATTAGCAGTCTTTCAATATCATTATGGAGTTGGAATGTTTGTTATTTTAGK???????????????????????CTCTTAGTCTTTTTAATCGTATTTCTTTTGGTGGAGGCTCTAATTATTTACTTTTTAATAGAGACTTAAGTCGACGAGAGATTTTTGTAATTTTTCCTTTTCTTTTAATTATCT??????????????????????????????????????????????????????????????????????????-------------??????????????????????????????????????????????????????????????????????????-??????????????????????????????????????????????????-???????????????????????????????????????????????????????????????????????????????????????????????????????GGTAGGATAAAA??????????????-????????????????????????????????????-???????????????????????????????????????????????????????????????????--??????????????????????????????????????????????????????------???????????????????GAAGGTTTGGGGAGCAAAY??????????????????????????????????????????????????????????????????????????????????????????-??????????????????????????????????????????????????????????????????????????????????????????????????????????????????????????????????????????????????????????ACCCTTATATTTCGGGGTTAGGCGCGCTACATTTCTCTCTTAGAAAAGGAG???????????????????????????????????????????????????????????????????????????????????????????????????????????????????????????????????????????????????????????????????????????????????????????????????????????????????????????????????????????????????????????????????????????????????????????????????????????????????????????????????????????????????????????????????????????????????????????????????????????????????????TGATTTTATTTATTCTTTCAGAAATTTTGTTTTTTTTTTCTTTTTTTTGGGCTTTTTTTCATAGTAGTTTAGCACCG????????????????????????????????AGGAATTGTTGCATTAAATCCCTTTTCTGTTCCTTTATTAAATACTGCTGTATTATTAAGTTCTGGGGCAACGGTTACATGGGCGCATCATGCAATACTTTGTGGTTTAAAAAAAGAGGCTCAATTTGCTTTATTTTTAACTCTTT??TTGGGTGTTATGTTTACGGGGTTGCAAGCATTTGAATATTATGAGGC???????????????????????????????????????????????????????????????????????TTATAATTGGTACGACTTTTCTTTTTATTTGTTTTTTACGTTTACTCTCTAATCAATTTACTCGTCGTCAACATGTTGGGTTTGAAGCCGCGAGTTGATACTGGCACTTTGTCGATGTGGTTTGATTATTTTTATATTTATGTATTTATTGATGAGGCT????AAAATTTCTTTAATGCAAAATCTTTTTGTTTTTACTTTTTTTAGATATTTTATAAATGTTTTGGCTGGGTTGTGTGTTCTCTGGTTTAATCATCTTTATTATTCGGATAGTCCAGAGACTTGACTTTTAGAGTTTCAAGATGTTGGGGATC??????????????ATTGTTTTTTTTCATGACCAAGTTATGTTTTTATTAATTATTATAGTTACTGTTGTTTTATG??????????????????????????????????GATCGTCATTTAATCGATGGTACATTTTTAGAAATTGTTTGAACGATTATTCCTGCTGTTATATTGATTTTTATTGCATTGCCCTCTCTTAAATTATTATATTTAATGGATGAGGTTATTTCTCCAGCTTTGACAATTAAAGTTATTGGGCATCAATGATATTGATCCTATGAATATTCTGATTATGAAGGGGATACGTTAGGGTTTGATTCTTATATGATTCCGACTTCGGATTTAGTTTCTGGAGAGAATCGTTTGTTAGAGGTTGATTATAAACTTTTAATCCCTATTCAAACACATATA??????????????????????????????????????????????????????????TAAAAATTGATGCGGTTCCGGGTCGTCTAAATCAAACTGGTGTTTTTATAAAA?????????????????????????????TGAAATTTGTGGGGCAAATCATT??????TGCCCATTGTGATAAAGGGAGTTGGTTTAAATGAATACGTTCAATATTTAAATTATTTAAAATGTATTATAAATACTTAGTTATTATTATTATTTTATTTTTATTGGGGAGTTGGGGTAT????????????????????????????????????????????????????????????AACTTTTTTTTTTTTTTTAATAAGTTCTAAAGAAATAGATCTTTTAATAGAACAAATTTTTATGATTATGGGGTTAACTATTGCTGCTGCCGAATCTTCAATTGGTTTAGCTATTTTAGTTGCATATTATAGAATTCGAGGAACAATTGTTTTAAAATCTTTTAGTTCTTTGCGAGGATAATGATTAATCTTTTTATTTTTTTTTTTCTTATTTTTATTTTGGCGGGGTTATTTATATTTCTTTCTTTTTTGATAGGGGAAAAAATTCCAGATCGAGAAAAGGTTTCTGCTTATGAGTGTGGTTTTGCTCCCTTTAATTTTTTAGGGCGCCCTTTTTCAATACGTTTTTTTTTAATTGGTATTTTATTCTTGATTTTTGATTTAGAAATTTCTTTTTTTTTTCCCTGATGTGTTTTATATAATTCGACTGCTCCGTTTGGGTTTTGAACTATGATAGGTTTTTTTTTTGTATTAGTTTTGGGTTTGATATATGAATGAGTAATGGGGGGATTAGAATGAGAGTAAAAATAAAGTAAAAGAAAAAGTCCTATCTATTATGAAAATAATAGAAATTTTATACCAACTCCGGTTTCTGCTTTAATTCATGCTGCAACGATGGTTACTGCAGGTGTTTTTTTATTAATTCGTGCTTCTCCTTTATTTGATGTTGTTCCTCTTATATTAATTATTATTTCAATCGTTGGGGTTTTAAC??????????????????????????????????AATGATTTAAAAAAAATAATCGCT??????ACTTGTAGTCAATTAGGTTATATGGTTGTTGCTTGTGGGCTTTCTCATTATGCAATTAGTCTTTTTCATCTTATGAATCATGCTTTTTTCAAAGCTTTAT??????????????????????????????????????????????????????????????????????????????????????????????????????????????????????????????????????????????????????????????????????????????????????????????????????????????????????????????????????????????????????????????????????AATTTATT?????????????????????????????????????????????????????????????????????????????????????????????????????????????????????????????????????????????????????????????????????????????????????????????????????????????????????????????????????????TTTTTTTTTTTTTTTTCTTCTCAAATTTTTGGTTATCCCCTTCAGG?GATCGGATCTTCTGTTTTTTCTCTTTATAATTTTTTTGGTTCTGCTTGACAAATAAATTTTTTTTTTAATTTTTT????????????????????????????????????????????????????????????????TTAGAGGTTGT??????TAGGGGTGTTGTTCAATTTTTTATTTTTCAAACTCAAAAGTTG????????????????????????????ATTATGCTTTAGTTTTTTTTCTTGGGATACTTTTTTTAATTTTTGCACTTTAATTAAAATTTTTGAAGGGGTTAAGTTAAGCAAAACAGTTGACTTTCAAAGTTAATTATGTAGGTGGAAATCTTACACTCTTTGGATGCCAC?GTTAGAGGTAGGTACTTATAATATACAATATTGGTGGGGGTTCTCGGTTTTATTTTTCTTGTTAATTTTTTTAGAAGTTATAATCTTTCCTTTTGTAAAGCGTGGTTGATGAATAAGACAGTTCTTATTGGGGTG---------------A????????TTTAGGGTGAAAAAAAAATTATGGAAAAAGATTTCCATGAGAACTGTCGCAAAATGTTTAAAAAAAGCCTTT-TTTTTGCT--AGTGGGATTTTGTTTAAAATATTTTT-TTGAGTTTTCTTACTTTAAAACATCTTTTTGTTTAATGA-TTTTTTTTTTATTTAT-------------------------------------AAAATTTTGT------------TCTAAGAAGATTC----------------------------------------------GGGCTCGTTTAGCGATC?????---????-------???---??????????????--??????RRAARTRGAWGWR-SCAAATGA--AGAGATAGTAGGAG--CGCTTCCATCTTTA--------AGCTTCGTGGAGGGGTGAGATG----------------------------AAGAGGTGATAGCAGCGTTTCCATGTTCAGACT----------CCGAAGAGAGTATGGAAATTGATGGGGAAGA--ACTACCTTTGGATCCCCTCCCGATTAGTGGAGCT--ATTAGAGGAGAA--AACATAGTGGCA-------------------------------CCCGTACCAGCCATGGGAGATGCGGGTCTTTCAGTTCATTTTTGGCGTGCACTCAAGAACACTCCATTTGAATTAT-TGGCAGAAGTTTATCGGTCAGGTGAGCAGGGAGAAG--TT---T--ATTCGTCAGATGAG---GAAGACGAGTTAGTAG??????????????????????-??????---------------------?????CAAAAGAGTCAGAGGACAAATTTTTGAGTGGGCTAAT--AGT--GGAAATGGTGGAAAACGTAATTGTCT-AAGATAAATACCA-GAA-CAGTT-AGTAAACAGATTATTGAGTGGGT???????--???????GGGGATAACGTGGTTAGGTAAAAT-ATAGTGTCGGCGGCAACGGAAGTGGGGAGGGGGAGAGCTCTCTTGCTCTCTAATTTGTAGTTTTTGAGTGGTTTGTATTATTTTTT--TTTTTAATTTTTTCTTTATGAGTCTGTCTTGGTAGAGGAG-GCGATTTTTTTAGAAGCCCCGTCTAAATGCTTTCTAGTTATTGAACTCGATGGGTGTTTTCTACAAATCATAAGGACATTGGTACTTTGTATTTAATCTTCGGAGCAGGAGCTGGTTTAATTGGAACTGCTTTTAGTATGCTTATACGATTGGAGCTTTCTGCGCCGGGGGCGATGTTAGGAGATGATCATCTTTATAATGTAATTGTTACAGCACATGCTTTTATTATGATTTTTTTTTTGGTTATGCCGGTCATGATTGGGGGATTT???????????????????????????????????????????????????????????????????????????CTTTTGCCTCCTGCGCTTTTTTTATTATTAGGCTCTGCTTTTATTGAACAAGGGGCGGGGACGG????????GTTTATCCTCCTCTTTCTAGTA????????????????????TCTGTTGATATGGTTATTTTTAGTCTTCATTTAGCTGGGGTTTCTTCTATTTTAGGTGCTATTAACTTTATTACTACAATTTTTAATATGCGAGCCCCGGGTGTGTCTTTTAATAAACTACC????????????????????????????????????????????????????????????????????????????????????????????????????????????????????????????????????GGACCCAATATTATTTCAGCATTTATTTTGATTCTTTGGGCATCCAGAAGTTTATATTTTAATTTTGCCTGGTTTTGGTATGATTTCTCAAATA????????T?????????????AACAAGTTTTTGGGTATTTAGGAATGGTTTATGCCATGCTTTCTATTGGG?????????????????????????????TATGTTTACTGTTGGGATGGATGTAGATACAAGAGCATATTTTACTGCTGCTACTATG???????????????????????TAAAGTTTTTAGTTGGTTGGCAACTATTTATGG??????????????????????????????????????????????????????ATTTACAGTTGGTGGTTTAACTGGGGTTGTGTTAGCAAAT???TCTCTTGAT???GTTCTACATGATACATATTATGTAGTTGCGCATTTTCATTATGTTCTTTCTATGGGGGCTGTTTTTGCTATTTTTGCGGGGTTTTACTATTGAATTGGCAAAATAAGTGGTTATTGTTATAATGAATTTTTTGGGAAAGTTCATTTTTGATTAATGTTTATCGGGGTTAATCTAACTTTTTTCCCTCAACATTTTTTAGGTTTAGCAGGATTTCCAAGACGATATTCGGATTATGCAGATGCTTTTTTGGGTTGAAATTTAATAAGTTCTTTAGGGTCTATTATTTCTATTTTGAGTGTTGTTTGGTTTTTATATATT???????ATCTTTTTGTTACAGAAGAAAAATTTTTGGGTTGAAAAGAAGGATTTTCTTTAGAATGAATTCATTCTTCTCCCCCCTTATTTCATACTTATGAGGAGTTGCCCTTTGTACAAAAAGTAAATAATTTTTAGAATAGTGCCGGGTTTATATACCGGTTT

P53 TGTAAGAAAGACAAAGGGTTAGTCATTGGGTTCATGCCCCAACCAAGTGAGTTCGAATCTCTCTCTTACACAAAATAAAAATTAT-AAAAAAAAAAGAGGATAACTAAGATAAACTAAAATTAGACGATTTTTTTCGAAATGGCGTTCAGTTGCTTATTGGTTGTTTCTTCAGAAAAGTGA-TTTTTTTTTTATTTGAATTGAAACATCTTATTACTAAGTAAAAATCAAACGAGATTCCGAGAGTAGTGGAGAGTGAAATTGGAGTTGTGCTTGTGGTGGTCAT?GATCTTGCTTAAACATTAGTTTATACTGATAATGAAAGTACTGTAAAGGAAAGTTGAAAGAGAGTTGAAAGGAATTTGAATCTTTTATTTTTGAAGTAGCTTTAAAAAAGCGTACCTTTTGTATAATGGGTAAAAGAGATTTATTTGGCATATTTAAAATGGAAAATTTTAAGTAA-TTTTTTTTTAGTCAAATTTCCCGAAACCAAGTGATTTAATCATGAATTGTGTG??????????????????????????????????????????????AGGGGTGAAAGGCTAATCGAACTTGGAAATAGCTGGTTTTCTGCGAAAACTATTTAAGTAGTGTTC-TTTTTTTTTTTATTTTAATTGTCATTTTAAAAAAAACAAAAATAAAAAAAAAGTAAACAAACAATAAATGAAAAGATTTTTTGTTAAAAGAGAAAGCTCAAATCAGAAGCTATTGTCA-TTTTTTTTTTTTTAGTTTAAAAAAAAATTAAATTTAGACAATAAAAATGTAGGCTTGGAACCAGCCATCTTTTAAAAAGTACGTAGTTGTTTATTTAAAAATTTTAAGTTTTTTTATAATTTTGATGGTTAAAATAAAACAGAAGCTCTGAAAATAATTATATATTTCGTAGCAGAATAAATTGTTTTCGATAT-C------------------TCCCTAGTTTGAAATTTTTTTTCTTAATACATCGTGTTCAGTTGTAATAATTTTTACATAAGTAATCTAAATATTATTTTTTTTTTCTTAAGATTACCTTAGTCAAGGGTAATACAGTTTCTAAAAAAGAAATTTTTTATGAAGATTTTTTATAAAAATATATAATATCGGGAAAAG-AAAAAAAAAGAACTGTTTTTCTAATTAAAGAAAGAAAAAAAAAAAGAAAAACATTTTTTTAAGGAACTCGGCAAAGTTAAACTTCGACTGTTTACCAAAAACATAGCTTTTTGATTTTTATAAAAGGTGAAACCTGCCCGATGGTTGAATCTTAATATGTTTGTTCCGCTTACTAATAAAGACAATTAAATGGCCGCGGTAACACTGACTGTGATAATGTAGCGTAATCAATTGTCAATTAATTGTTGACCGGTATGAATGGTATCCCGAAAGTTTTTCTGTCTTAAAAAAATATTCAATGAAATTAAATCTGTAGTGAAGATGCTACATTTTAATTGTTAGACGAGAAGTCCCCATGGAGCTTTACTGTAAGCTTATATATATAATTTTTTTTTTTTTATAAGTAAGACAGTTTTGTTGGGGCGACAGTTTTTTAAAAAGTAACGAAAATGAGCTATGACGCATGTTTAACTTTGAAATTTTTTT-ATTGATGAGACATTTTTGGTGTGTTTTTTGATCCGTTATTTTGAATGAAAAAAATAACGAAAACAAATAAAAGTTACCCTGGGGATAACAGCGCAATAATGTTTGAGAGTTAATCAACGACAGTGTTTGCGACCTCGATGTTGAATTGTAACGTCCTACGGTGTAGTCACTCGTAAGGGTTGGTTTGTTCATCCATTAAAGTTATACATGATTTGAGTTAAAAGCGTGGTGACACAGCTTGGTTTCTATCTACAATTAGAAAACAAAAATATGTTGTTTTTTCGTACGAAAGGATCAAAAATCAATAGTTCTCTTAATATAACTATTCTTTAAATTAGGATTGTTTCTAAAAATAAAAAGAAATATTTTTGTTTGTTGTTTTTATTAATGTATCTTTTAGTTTTATTTTTTCCTTTAATTGGAGCTGTTTTAACAGGTTGTTTTGGAAGAAAAATCGGAGAAAGAGGAGCGGGGATTTTAACTTCAAGTTGTTTAGTTTTTAGTTTATCTTATTCTTTTTTAATCGCGATTGAAGTTTTATTTAATTCAACAACAACGTATTTGAAATTATGAAAATGATTTGATTCGGGGTTGTTTGTTGTTTTTTTTGGTTTTCAATTTGATGGTTTAGTAGTTATTATGTTATTTGTTGTTTTTATTGTTTCTACTTTGGTTCATATTTTTTCTATTGCTTATATGCGGGGAGATCCTCATATTCCTCGATTTATGACATATCTTTCTTTGTTTACTTTTTTAATGGTTTTATTGGTAACTAGCGATAATTTTCTTCAATTATTTATTGGGTGAGAAGGGGTTGGTCTTTGTTCTTATTTATTAATTAATTTTTGATTAACTCGATTAGAGGCAAATAGAGCTGCTATTAAAGCAATGTTAGTGAATAGAATTGGTGATATTGGGTTGCTTTTAGCAATGTTTTTACTTTGGGATCTTTTTGGATCTCTAGATTTTTCTACTATTTTTAATTCTATTTTTTTTTCTAATCAAATATTTTTTATTTGTTTATTTTTATTTTTTGGGGTTATGGGAAAATCTGCTCAATTAGGATTACATACTTGGTTACCGGATGCAATGGAAGGTTATTGGGCCTTTTAATTAAAAAATTAATTAAAAAAATTACTATACATTAAAAAAACAATTTGTTAGTAAAATATTTGTTTATTAATGGACAATAAAATATTTATTTTAATGTCTAAGAGACTTTATGTGATTTACTTTTTTTTATTTGATTAAATCTATTATTATTATTATTCCTTTACTTATTGCTGTTGCATTTTTAACTTTAGCAGAACGAAAAATTTTAGGGTATATGCAAATAAGAAAAGGGCCAAATGTTGTTGGGGGAGGGCTTCTTC????????????????????????????????AAAGAAATGATTCTTCCCCATCAAGCAAATAAATTTGTTTATCTTTTGGCTCCAGTTATATCTTTTACATTAGCTTTCTTTGTTTGAGGTTTTCTTCCTTATGAGAAAGGAGTGAGTATTAGTGATTTTAAAATTAGTCTTTTATGAATTTTGGCTATTTCTTCTATTAGTGTTTATGCAATTTTAATGTCTGGATGAGGAAGTAAGTCTAAATATGCTTTTTTGGGTGCTATTCGGGCGGCGGCTCAAATGATTAGTTATGAGGTTTCAATTGGGCTAATTCTTATTTCGGTTTTATTATGTGTTGGTTCTTTAAGTGTTACTGAAATTGTTTTAGCGCAAAATAATGGTATTTGGTTTTTTTTTCCACTATTTCCTGTTMCAATAATGTTTTTTGCTTC??????????????????????????????????????????????????????????????????????????????????GCTTCGATGTCTTTTGCTTTATTTTTTCTTGCTGAATATGCCCATATTATTTTAATGAGTTGTTTAACAACGATTTTTTTTGGGGGAGGATGGCTTTCTCCGATTCCAGATTTTAAGGGTGGGGCGGGCTGGTTTGGTTTTAAAGTTGTTTTTATTATTTTTTTTTTTATTTGAGTAAGGGCTTCATTTCCTCGAATTCGATATGATCAGCTTATGGCTTTGTTATGAAAAGGGTATTTGCCTTTAAGTTTAGGAATGGTCATCTTTGTGGCTAGTGTTCTCTTTGGGTTTAATGGTCCCCCTCCTATTTAAAATATTTTAATTAATTATTATGAAAAAAAAAAAGAACTAACAGATTTGCAAGTGCCATTACGAAAAAAAAATCCTATTTTATTTTTAATCAACGGATTTTTAGTTGATTTGGTTTCTCCTTCTAACATTACTTATTTATGAAATTTTGGATCW???????????????????????????????????????????????????????????????????????????????????????????????????????????????????????????????????????????????????????????????????????????????????????????????????????????????????????????????????????????????????????????????????????????????????????????????????????????????????????????????????????????????????????????????????????????????????????GTTCAGTGAGTGTGAGGCGGGTTTAGTGTTTCTAATGCAACATTAAATCGGTTTTTTAGTTTACATTTTTTACTTCCTTTTATTTTAGTTTTTCTTGTTATTCTTCATTTAGTTTATTTACATGTTGATGGGTCTAGTAATTCAACAGGATTAAATTCTTCAATTGACGGAGTTTCATTCCATACTTTTTATACATCAAAAGATTTTTTTGGTTTTTTTTTTCTTTTTTTTTTATTTTGTTTTTTTGTTTTTTTTTTGCCTAATTTATTAGGAGATGCTGAAAATTTTATTCAAGCGAATTCTTTGGTTACTCCGGTTCATATTCAACCAGAATGATATTTTTTATTTGCTTATGCAATATTACGTTCTATACCAAATAAATTAGGTGGGGTTATTGCAATGTTTTGTAGTATTTTTATTTTATTTTTTTTATCTATTTTACATCAAAGTCTTTTAAAGGGACTTTTTTTTCGTCCTTTAGGTCGAATTGCCTTTTGGTTTTTAATTATTGATTTTGCTTTATTAACTTGAATTGGATC????????????????????????TTTAATTGGTCAAATACTTTCTTTTTTTTATTTTTTTNATTTTTTRGTTTTAATACCAGTTTTGGGTATTATTGAAAATCAATTATTAAATAAAAATTAATGAAATTTTTGTTTTAGGCGGTTTTCTTTTGAGCTTCGTGGTTTTAAGTCTTCGTTATTTTCTTTTAAAACTTTCTCTTTTTTATTTATTATTATTTTTTTTTTTTTTTTTTTTTAAATTAGAATGGGGAAAGCTTTTGGTTTTAATTGGTTCTTTTGCTGTCTTTCTTTTATTTGGACCAAAAAAAGAAGAACAAAC?????????????????????????????????????????????????????????????????????????????????????????????????????????????????????????????????????????????????????????????????????????????????????????????????????????????????????????????????????????????????????????????????????????????????????????????????????????????????????????????????????????????????????????????????????????????????????????????????????????????????????????????????????GTTCCAAAAATAGGGTTTTTTTCTCTTATAATTTCAATTGGATTGCCTGTAAATTTTTTTTTTTTAGGGATTCTTTTTTCTTTGTTTGTTGGAGCTTTGGGTGCTTTAAATCAAACTAAAATAAAACGACTTTTGGCTTATAGTGGGATTGGTCATWTGGGCTTTATTTTATGAGGTTTGGAAAATGGTTCTTTTGAAA????????????????????????????????????????????????????????????????????????????????????????????????????????????????????????????????????????????????????????????????????????????????????????TTTTGCTGGCTTTTTTGGAAAATGATTTATTTTGTTATCTGGAATTCTTTCTAAATCATATTTTATCTTTTTTTTTGCTGTTTTTTGTTCTGTTATAGCTGGGGTTTATTATATTCGAATTATAAAAATACTTTTTTTTCAAAAAAATTCTTTTCTTTTAATTACAATTAAAGTYTTAAAAAAAGAATCTAAATTAAATTTTAAAAAAGTTTTTTTAATTGGTTTTTGTTTTTATTTTATTCTTTTTTTTTTT????????????????TTTTTTTTTTTTTTTCTCAAATAATTTTTGATTTATTTTAAAATGGAATTTTTTTCTTTTTTTTTTATTTTGGGGATAATTG??????????????????????????????????????????????????????TGGTTCTTGTTTTTATAAATTCTGCCGTTTTTTTCCTTTTACTAGGAATAGATTTTCTTGCTTTGATGTTTTTACTTATTTATGTTGGGGCAATAGCTATTTTATTTTTATTTGTTATTATGTTATTAAATCTAACTGACTACCCTCCTGTTTTAAAAAGAGAGGTTGATATGACAAATTATATACCAATTGGATTTATAATTGGGATTTTTTTTTTTTCAGAAATTGCTTCAAGTGGATTACTTTTGGGTTCTTTTCAAATAGAGAATTGAGACCTTTCTTTTCCTTGATTTCTTATTTCTTATCATAATATTGAGGCCTTAGGGCAGATTTTATATGTTTCTTGTTTTTGTTTATTTCTTTTGGCCAGTTTTATTTTATTAGTTGCTATGATGGGTGTGATTGTATTAACTCAAGAAACAGAATCTTTAAGTAAAAAACAAGATC?????????????????????????????????????????????????????????????????????????????????????????????????????????????????????????????????????????????????????????????????????????????????????????????????????????????????????????????????GGGTTTAATATATTTTTCTTTTATTGTTTCCCTTTTTGTTTTTTTAGTTTTTTTAAATATTTTAGGTTTATGTCCTTATGTTTTTACTCCGACAACTCATATTATTGTTACTCTTGGGTTTTCTTTTTCTATTGTTATTGGAGTAACTTTTTCTGGATTTTATAAATTTAAAAAAGATTTTTTTAGTATTTTAATG?????????????????????????????????????????????????????????????????????????????????????????????????????????????????????????????????????????????????????????????????????????????????????????????????????????????????????????????????GTGTTTTGTCTGTTGGTACAAATTTATTTAACTGATACAATTTTTTTACATTAA-----------------AAATGACAGCCGTAGAAATTTTTTTATGCACAATTATAGGTATATCGTTAACAGTGAATTATGGATTTGGTATTGGGTGATATAGTAGCTCTTCTCAAGCCTGGTTAAGGCACCAAATGGGC---ATAGACGGTACAGATATGGTCCATCCTTGAGCTTATTTTAATTATGTGCGCCAGCATTCTATTGCTCGTAATGTTGGGGTACAAGTGAATCTAGATTCAACTGAAGGACCAAATGATGTTGGGGTACAAGTGAATCA???????????????????????????????????????????????????????????????--????????------------------????????????????????-?????????????????---???????????????--???-???---------------------------------------------?????????????GATCA-----ATTTGA-AGATGTGGCTTT---TTTAG-GGGGGAG-TACAAGTACACTTACACCAGAAA----GTGTAAATAGGGGAGCTCCAAGTGGTAATATAAGAAATGTATTAAGTGATTCATTAA-TAGA-G-AG-GAGAGGCCAAGACCAAACTTTAAGTGA--AA-TAAAGGTAGAAACACCTCC----AGGTGCTTCAAGGGGTGGCCGATG-A-------G--CCAGT-AT-----AAATCAAGGTGAGGCTTTAAG---AGAAAACAAGGTAGAAATTGGGG----TTTTAAGAGAAAACAAAGT--------AGAAAT--TGATGAGGTAGTTGGCTCGACTTTTAGTTCGATCCCCTTAGAGGTTGAAGGGATGATGGCTCATGTGTGAAATCAATTTGACATGGTGTTTAACATATTGGCCGATAAGCCCCTGGTAGGTTGCATTTTTTTTTCTTACCAGCTTCTAAGAATAACGAATCAAATGCCAAACCCACGGGTTTATAGGTGATTTGCTTTGGTGTATCTTTTATTTATTTTTGTTCTTTTAATGGTTTAGCTGTTTGTACGTAGTAGGATAAAAAAGTGGGACATTTTTCTAGGAGTTTATTTGTATGCGTCTGGGGGGATTAGAATAAGAGTAAAAAA???????????????TTACAAAAAAAGTGGGGTGTTTTTTTTGGAGTTTTTTTTTATG???????????????????????????AAAAAAAAAACTCTTTAAGAGTTTACAAAAAAAGTGGGGTGTTTTTTTTGGAGTTTTTTTTTATG???????????????????????????????????????????????????????????????????????????????????????????????????????????????????????????????????????????????????????????????????????????????????????????????????????????????????????????????????????????????????????????????????????????????????????????????AGGGACAGCGAGGGAGAGAAAATGTGTTTTAAAAAAGCGGGCTTTAGAGTGGTCTTTGGCTTTATTGTTTAGTACTTTAGTTTTTTGGGGTGGATTTGACGGAGAAAGTCATTTTCAATTTTTTAGTTTAGTAGAATGAAATATATTTTCTACTTTAGATTGAGGCCCGATTGTTTTTGCAGTTGATGGTGTTTCTTTGGTTTTTTTACTTTTAACGACTTTTTTAATTCCAATTT?????????????????????CAATAAAATTCTTATTTAAAGAATTTCTTTTGTGCTTGTTTTTTTTAGAATTTTTATTAATAGGTGTTTTCATAGTGTTTGATCTTCTTTTGTTTTATCTTTTTTTTGAGGGGATATTAATACCAATGTTTTTTTTAATTGGTATTTGAGGCTCTCGAGAAGAAAAGGTTCGTGCTTCTTTTTATTTTTTTTTTTTTACTTTTATAGGCTCTCTCTTTTTCTTTTTTATAATACTTTTTTTATATCAAAGGATTGGAACAACAGACTATTTTCTTTTACTTAATATTAAATTATTTTTAAATATTCAAAAATGAGCCTTAGTCGGGGTTTTTCTTAGTTTTGCGGTGAAACTACCTCTTATTC?????????????????????????????????????????GTTGCGGGCTCTGTTATTTTGGCTGGAATTTTATTAAAATTAGGGGGTTATGGCCTTTTGCGTTTTTCTTGACCTCTTTTTCCGGGGGCTTCTTTATATTGGTCTCCAGTTATTGTTTTTTTTAGTGTTGTTGCTGTTGTTTATGGAGGTTTAATGACATGTCGTCAAATTGATTTTAAACGGCTTGTTGCTTACTCTTCTGTTGCTCATATGGGACTTGTGCCTTTGGGTCTTTTTACACATGTTATAGAGGGGTTAATTGGGGCTCTTTTTTTAATGTTGGCGCACGGATTTGTTAGCTCTGCTCTTTTTATTGGAGTAACTTTTTTGTATGATCGCCATCATACTCGTTTAATAAAATATTATCGGGGTTTGACTTTGACAATGCCTCTTTTTGTTATTACAATGTTAATTTTGTCTTTGGCGAACATGGGTTTTCCTCTTAGTTGTAATTTTGTTGGAGAGTTTTTTTCTTTATTAGCAGTCTTTCAATATCATTATGGAGTTGGAATGTTTGTTATTTTAGGGGTTCTTTTTTCTGCCATTTATTCTCTTAGTCTTTTTAATCGTATTTCTTTTGGTGGAGGCTCTAATTATTTACTTTTTAATAGAGACTTAAGTCGACGAGAGATTTTTGTAATTTTTCCTTTTCTTTTAATTATCTTTTTGGGGGGGATTGTGCCTTTTCCTATTATTGATTTAATTAAAAATAGTCTTGTTTTTAGCCCGGTTAGTTAG-------------TCCTGGGGTTTTAGGGATAAAAAAATATTTAGTCTAGGTTATACATGCTAGTGTATGCGAACAGGTGAGGATAA-AATAAAAAAATTTTTTTTATTTGTTGTATTAAAAAAAGAAAAGTTTTTTA-TTCTTTTTTTGAAGATGCATAGTTTAACCACATTTTCACTGAAACAAAAGAAAACCCTTGGCAGCAGTAATGAATTTTATGTAATATACGAAAGTAAGACATAGGTAGGATAAAAAAAATCTGTCAAAT-TAAGTGCCAGCCGACGCGGTAAGACTTAAGGATTTA-TTTTTTAATAAAAAGCAAAAAGCGTGTTAAGGATTTTTTAAAAAAAAAAATAAATAGAATTTTTTTC--GTAATTGTAATATGTTAAAATGAAAAAAAGAATTTTTTATATGAAGATAATTTA------TTTTTTTTTTCTTAAATACGAAGGTTTGGGGAGCAAATAGGATTAGGTACCCTGGTAGTCTATACAGTAAAATAATATCGCTTGAGTAGTACGGTCGCAAGATTGAAATTCAAAAGACTTGGCTGTTC-ATTGTTTATTAGAGGAGCGCGTCGCTTAATTCGATTATCCGCGAGAGACCTTACCG??????????????????????????????????????????????????????????????????????????????????????????????????????????????????????????????????????CTCTTAGAAAAGGAGGGGTTCGGATTGTTTTTAAAATAAGATAATGAAGTTGAATTTAATAGTAATTGAAAAGTAGTGCGTTTCAATGAATGTTATTGCAATGTTAGTACAAATAGTCCGTCGCCAGCTTAGAAATAGGCTGTAAGTCGTAACATAGTAAGAGTGAGGGAACTGGCTCTTGATGCAGTTTCATTCGTATCATTTAGTTGAGCCTTCTCC????????????????????????????????????????????????????????????????????????????????????ATATTTGGGGCTTTTGGTTGTTGTTGGAGTTATGTTTGTTTGATGACAAGATGTTATACGAGAATCAACTTTTCAAGGTCACCATTCTTTAATTGTAAAACAAGGGATTAAATATGGTATGATTTTATTTATTCTTTCAGAAATTTTGTTTTTTTTTTCTTTTTTTTGGGCTTTTTTTCATAGTAGTTTAGCACCGGTTGTTGAACTTGGAGTCGTTTGGCCTCCACAAGGAATTGTTGCATTAAATCCCTTTTCTGTTCYTTTATTAAATACTGCTGTATTATTAAGTTCTGGGGCAACGGTTACATGGGCGCATCATGCAATACTTTGTGGTTTAAAAAAAGAGGCTCAATTTGCTTTATTTTTAACTCTTTTTTTGGGTGTTATGTTTACGGGGTTGCAAGCATTTGAATATTATGAGGCGCCTTTTACTTTATCTGACTCTGTTTATGGGGCCACTTTTTTTGTTGCGACAGGGTTTCATGGACTACATGTTATAATTGGTACGACTTTTCTTTTTATTTGTTTTTTACGTTTACTCTCTAATCAATTTACTCGTCGTCAACATGTTGGGTTTGAAGCCGCGAGTTGATACTGGCACTTTGTCGATGTGGTTTGATTATTTTTATATTTATGTATTTATTGATGAGGCTCATAAAAATTTCTTTAATGCAAAATCTTTTTGTTTTTACTTTTTTTAGATATTTTATAAATGTTTTGGCTGGGTTGTGTGTTCTCTGGTTTAATCATCTTTATTATTCGGATAGTCCAGAGACTTGACTTTTAGAGTTTCAAGATGTTGGGGATCCTATTGTAGAAGAAATTGTTTTTTTTCATGACCAAGTTATGTTTTTATTAATTATTATAGTTACTGTTGTTTTATGACTTTTTGTTGAAGCTTTTAAAAATAAGTTTTATGATCGTCATTTAATCGATGGTACATTTTTAGAAATTGTTTGAACGATTATTCCTGCTGTTATATTGATTTTTATTGCATTGCCCTCTCTTAAATTATTATATTTAATGGATGAGGTTATTTCTCCAGCTTTGACAATTAAAGTTATTGGGCATCAATGATATTGATCCTATGAATATTCTGATTATGAAGGGGATACGTTAGGGTTTGATTCTTATATGATTCCGACTTCGGATTTAGTTTCTGGAGAGAATCGTTTGTTAGAGGTTGATTATAAACTTTTA??????????????????????????????????????????????????????????????????????????????????????????????????????????????????????????????????????????????????????????????????????????????????????????????????????GTGATAAAGGGAGTTGGTTTAAATGAATACGTTMAATATTTAAATTATTTAAAATGTATTATAAATACTTAGTTATTATTATTATTTTATTTTTATTGGGGAGTTGGGGTATAATTTTAAATAGAGGACATTTTATTATTATGCTTGTTTCTATTGAATTAGTTTTATTATCAACTTTTTTTTTTTTTTTAATAAGTTCTAAAGAAAT?GATCTTTTAATAGAACAAATTTTTATGATTATGGGGTTAACTATTGCTGCTGCCGAATCTTCAATTGGTTTAGCTATTTTAGTTGCATATTATAGAATTCGAGGAACAATTGTTTTAAAATCTTTTAGTTCTTTGCGAGGATAATGATTAATCTTTTTATTTTTTTTTTTCTTATTTTTATTTTGGCGGGGTTATTTATATTTCTTTCTTTTTTGATAGGGGAAAAAATTCCAGATCGAGAAAAGGTTTCTGCTTATGAGTGTGGTTTTGCTCCCTTTAATTTTTTAGGGCGCCCTTTTTCAATACGTTTTTTTTTAATTGGTATTTTATTCTTGATTTTTGATTTAGAAATTTCTTTTTTTTTTCCCTGATGTGTTTTATATAATTCGACTGCTCCGTTTGGGTTTTGAACTATGATAGGTTTTTTTTTTGTATTAGTTTTGGGTTTGATATATG?????????????????????????????????????????????????????????????????????????????????????????????????????????????????????????????????????????????????????????????????????????????????????????????????????????????????????????????????????????????????????????????????????????????????????????????????????????????????????????????????????????????????????????????????????????????????????????????????????????????????????????????????????????????????????????????GGGGGGTTTATTATCTTTTCTTCCTTTGACTTATGTTTTTTTTCTTATAGGCTCTTTTTCTTTAATGGGGTTTCCTTTTTTAACAGGATTTTATTCAAAAGATTTAATTTTAGAATTTGCTTTTGGACAATTTTATTTAATTTTTGTGTATTGATTAGGTTGTTTTTCTGTTTTATTGACAATTATATACTCTATTCGTTTAATTTATTTAGTTTTTTTATCCAATATTAATTTAAAACGAGCAAACATTTTTTTTCTTAAAGAAGGAGAATTTTTATTTTTAATTCCTTTGGGCATATTAACTTTAGGAAGTGTTTTTTGGGGCTATTTAAGTAAAGAAATAATTTGGTCTTTTCAAATAGATGTTTTTTCAATACTTTCTTTAAAAATAAAAATATTTCCAATTTTATTTTGTTTTATTGGACTTTTTGGGACGATATTTTTTTTTTTTTTTTTTNCTTCTCAAATTTTTGGTTATCCCCTTCAGGTGATC????????????????????????????????????????????????????????????????????????????????????????????????????????????????????????????????????????????????????????????????????????????????????????????????????????????????????????????????????????????????????????????????????????????????????????????????????????????????????????????????????????????????????????????????????????????????????????????ACTTATAATATACAATATTGGTGGGGGTTCTCGGTTTTATTTTTCTTGTTAATTTTTTTAGAAGTTATAATCTTTCCTTTTGTAAAGCGTGGTTGATGAATAAGACAGTTCTTATTGGGGTG---------------AACCTTTTTTTTAGGGTGAAAAAAAAATTATGGAAAAAGATTTCCATGAGAACTGTCGCAAAATGTTTAAAAAAAGCCTTT-TTTTTGCT--AGTGGGATTTTGTTTAAAATATTTTT-TTGAGTTTTCTTACTTWAAAACATCTTTTTGTTTAATGA-TTTTTTTTTTATTTAT-------------------------------------AAAATTTTGT------------YCTAAGAAGATTC----------------------------------------------GGGCTCGTTTAGCGATC?????---????-------???---??????????????--?????????AGTAGATGAG-CCAAATGA--AGAGATAGTAGGAG--CGCTTCCATCTTTA--------AGCTTCGTGGAGGGGTGAGATG----------------------------AAGAGGTGATAGCAGCGTTTCCATGTTCAGACT----------CCGAAGAGAGTATGGAAATTGATGGGGAAGA--ACTACCTTTGGATCCCCTCCCGATTAGTGGAGCT--ATTAGAGGAGAA--AACATAGTGGCA-------------------------------CCCGTACCAGCCATGGGAGATGCGGGTCTTTCAGTTCATTTTTGGCGTGCACTCAAGAACACTCCATTTGAATTAT-TGGCAGAAGTTTATCGGTCAGGTGAGGAGGGAGAAG--TT---T--ATTCGTCAGATGAG---GAAGACGAGTTAGTAGAGGTGTTACTTCAGTACGGAAA-AGAGAT---------------------AACATCAAAAGAGTCAGAGGACAAATTTTTGAGTGGGCTAAT--AGT--GGAAATGGTGGAAAACGTAATTGTCT-?????????????-???-?????-??????????????????????????????--???????????????????????????????-???????????????????????????????????????????????CTCTAATTTGTAGTTTTTGAGTGGTTTGTATTATTTTTT--TTTTTAATTTTTTCTTTATGAGTCTGTCTTGGTAGAGGAG-GCGATTTTTTTAGAAGCCCCGTCTAAATGCTTTCTAGTTATTGAACTCGATGGGTGTTTTCTACAAATCATAAGGACATTGGTACTTTGTATTTAATCTTCGGAGCAGGAGCTGGTTTAATTGGAACTGCTTTTAGTATGCTTATACGATTGGAGCTTTCTGCGCCGGGGGCGATGTTAGGAGATGATCATCTTTATAATGTAATTGTTACAGCACATGCTTTTATTATGATTTTTTTTTTGGTTATGCCGGTCATGATTGGGGGATTTGGTAATTGATTAGTCCCATTATATATTGGGGCGCCGGATATGGCGTTTCCTCGATTAAACAATATTAGTTTTTGACTTTTGCCTCCTGCGCTTTTTTTATTATTAGGCTCTGCTTTTATTGAACAAGGGGCGGGGACGGGGTGAACAGTTTATCCTCCTCTTTCTAGTATTCAAGCACACTCCGGAGGTTCTGTTGATATGGTTATTTTTAGTCTTCATTTAGCTGGGGTTTCTTCTATTTTAGGTGCTATTAACTTTATTACTACAATTTTTAATATGCGAGCCCCGGGTGTGTCTTTTAATAAACTACCTTTATTTGTTTGATCTATTTTAATAACAGCTTTTTTATTACTTTTATCTTTACCT????????????????????????????????????AGAAACTTTAATACGACTTTTTTCGATC?????????????????????????????CAGCATTTATTTTGATTCTTTGGGCATCCAGAAGTTTATATTTTAATTTTGCCTGGTTTTGGTATGATTTCTCAAATAATCCCGACTTTTGTTGCTAAAAAACAAGTTTTTGGGTATTTAGGAATGGTTTATGCCATGCTTTCTATTGGGCTTCTTGGATTTATTGTTTGAGCTCATCATATGTTTACTGTTGGGATGGATGTAGATACAAGAGCATATTTTACTGCTGCTACTATGATTATTGCTGTGCCAACTGGGATTAAAGTTTTTAGTTGGTTGGCAACTATTTATGGAGGTGTTCTTAGGTTAGAGACTCCAATGCTTTGAGCTATGGGGTTTGTTTTTTTATTTACAGTTGGTGGTTTAACTGGGGTTGTGTTAGCAAATAGTTCTCTTGATATTGTTCTACATGATACWTATTATGTAGTTGCGCATTTTCATTATGTTCTTTCTATGGGGGCTGTTTTTGCTATTTTTGCGGGGTTTTACTATTGAATTGGCAAAATAAGTGGTTATTGTTATAATGAATTTTTTGGGAAAGTTCATTTTTGATTAATGTTTATCGGGGTTAATCTAACTTTTTTCCCTCAACATTTTTTAGGTTTAGCAGGATTTCCAAGACGATATTCGGATTATGCAGATGCTTTTTTGGGTTGAAATTTAATAAGTTCTTTAGGGTCTATTATTT?????????????????????????????????????????C????????????????????????????????????????????????????????ATTCATTCTTCTCCCCCCTTATTTCATACTTATGAGGAGTTGCCCTTTGTACAAAAAGTAAATAATTTTTAGAATAGTGCCGGGTTTATATACCGGTTT

R16 TGTAAGAAAGACAAAGGGTTAGTCATTGGGTTCATGCCCCAACCAAGTGAGTTYGAATCTCTCTCTTACAC??????????????-??????????????ATAAC?????????????????????????TTTTTCGAAATGGCGTTCAGTTGCTTATTGGTTGTTTCTTCAGAAAAGTGA-TTTTTTTTTTATTTGAATTGAAACATCTTATTACTAAGTAAAAATCAAACG?????????????????????????????????????????????????????GATCTTGCTTAAACATTAGTTTATACTGATAATGAAAGTACTGTAAAGGAAAGTTGAAAGAGAGTTGAAAGGAATTTGAATCTTTTATTTTTGAAGTAGCTTTAAAAAAGCGTACCTTTTGTATAATGGGTAAAAGAGATTTATTTGGCATATTTAAAATGGAAAATTTTAAGTAA-TTTTTTTTTAGTCAAATTTCYCGAWACCAAGTGATTTAATCATGAATTGTGTGAGCAAAAACTGGTGATT???????????????????????????????????????????????????????????????????????????????????????????????-??????????????????????????????????????????????????????????????????????????????????????????????????????????????????????-??????????????????????????????????????????????????????????????????????????????????????????????????????????????????????????????????????????????????????????????????????????????????????????????????-?------------------????????????????????????????????????????????????????????????????????????????-????????????????????????????????????????????????????????????????????????????????????????????????????????????????????????????????????????????????????????????????????????????????????????????????????????????????????????????????????????????????????????????????????????????????????????????????????????????????????????????????????????????????????????????????????????????????????????????????????????????????????????????????????????????????????????????????????CCCCATGGAGCTTTA?????????????????????????????????????????GACAGTTTTGTTGGGGCGACAGTTTTTTAAAAAGTAACGAAAA????????????????????????????????????-?????????????????????????????GATCCGTTATTTTGAATGAAAAAAATAACGAAAACAAATAAAAGTTACCCTGGGGATAACAGCGCAATAATGTTTGAGAGTTAATCAACGACAGTGTTTGCGACCTCGATGTTGAATTGTAACGTCCTACGGTGTAGTCACTCGTAAGGGTTGGTTTGTTCATCCATTAAAGTTATACATGATTTGAGTTAAAAGCGTGGTGACACAGCTTGGTTTCTATCTACAATTAGAAAACAAAAATATGTTGTTTTTTCGTACGAAAGGATCAAAAATCAATAGTTCTCTTAATATAACTATTCTTTAAATTAGGATTGTTTCTAAAAATAAAAAGAAATATTTTTGTTTGTTGTTTTTATTAATGTATCTTTTAGTTTTATTTTTTCCTTTAATTGGAGCTGTTTTAACAGGTTGTTTTGGAAGAAAAATCGGAGAAAGAGG???????????????????????????????????????????????????????????????????????????????TCMMCAACAACGTATTTGAAATTATGAAAATGATTTGATTCGGGGTTGTTTGTTGTTTTTTTTGGTTTTCAATTTGATGGTTTAGTAGTTATTATGTTATTTGTTGTTTTTATTGTTTCTACTTTGGTTCATATTTTTTCTATTGCTTATATGCGGGGAGATCCTCATATTCCTCGATTTATGACATATCTTTCTTTGTTTACTTTTTTAATGGTTTTATTGGTAACTAGCGATAATTTTCTTCAATTATTTATTGGGTGAGAAGGGGTTGGTCTTTGTTCTTATTTATTAATTAATTTTTGATTAACTCGATTAGAGGCAAATAGAGCTGCTATTAAAGCAATGTTAGTGAATAGAATTGGTGATATTGGGTTGCTTTTAGCAATGTTTTTACTTTGGGATC??????????????????????????????????????????????????????????????????????????????????????????????????????????????????????????????????????????????????????????????????????????????????????????????????????????????????????????????????????????????????????????????????????????????????????????????????????????????????????????????????????????????????????????????????????????????????????????????????????????????????????????????????????????????????????????????????????????????????????????????????????????????????????????????????????????????????????????????????????????????????????????????????????????????????????????????????????????????????????????????????????????????????????????????????????????????????????????????????????????????????????????????????????????????????????????????????????????????????????????????????????????????????????????????????GTTACAATAATGTTTTTTGCTTCGATTTTAGCAGAGACCAATCGTGCTCCTTTTGATTTAACAGAAGGAGAGTCAGAGCTTGTTTCGGGATATAATGTTGAGTATGCTTCGATGTCTTTTGCTTTATTTTTTCTTGCTGAATATGCCCATATTATTTTAATGAGTTGTTTAACAACGATTTTTTTTGGGGGAGGATGGCTTTCTCCGATTCCAGATTTTAAGGGTGGGGCGGGCTGGTTTGGTTTTAAAGTTGTTTTTATTATTTTTTTTTTTATTTGAGTAAGGGCTTCATTTCCTCGAATTCGATATGATCAGCTTATGGCTTTGTTATGAAAAGGGTATTTGCCTTTAAGTTTAGGAATGGTCATCTTTGTGGCTAGTGTTCTCTTTGGGTTTAATGGTCCCCCTCCTATTTAAAATATTTTAATTAATTATTATGAAAAAAAAAAAGAACTAACAGATTTGCAAGTGCCATTACGAAAAAAAAATCCTATTTTATTTTTAATCAACGGATTTTTAGTTGATTTGGTTTCTCCTTCTAACATTACTTATTTATGAAATTTTGGATCTTTATTGGGATTGTGTTTGATTTTACAGATAGTGACTGGGTGTTTTTTGTCTATGCACTATTGTTCTGATGTTAATTTTGCTTTTGCTTCAATTGGTCATATTATGCGAGATGTTAATTATGGGTTTTTATTAAGATATCTTCATGCTAACGGTGCTTCTTTGTTTTTTTTTTGTCTTTATGTTCATATT???????????????????????????????????????????????????????????????????????????????????????????????????????????????????????????????????????????????????????????????????????????????????????????????????????????????????????????????????????????????????????????????????????????????????????????????????????????????????????????????????????????????????????????????????????????????????????????????????????????????????????????????????????????????????????????????????????????????????????????????????????????????????????????????????????????????????????????????????????????????????????????????????????????????????????????????????????????????????????????????????????????????????????????????????????????????????????????????????????????????????????????????????????????????????????????????????????????????????????????????????????????????????????????????????????????????????????????????????????????????????????????????????????????????????????????????????????????????????????????????????????????????????????????????????????????????????????????????????????????????????????????????????????????????????????????????????????????????????????????????????????????????????????????????????????????????????????????????????????????????????????????????????????????????????????????????????????????????????????????????????????????????????????????????????????????????????????????????????????????????????????????????????????????????????????????????????????????????????????????????????????????????????????????????????????????????????????????????????????????????????????????????????????????????????????????????????????????????????????????????????????????????????????????????????????????????????????????????????????????????????????????????????????????????????????????????????????????????????????????????????????????????????????????????????????????????????????????????????????????????????????????????????????????????????????????????????????????????????????????????????????????????????????????????????????????????????????????????????????????????????????????????????????????????????????????????????????????????????????????????????????????????????????????????????????????????????????????????????????????????????????????????????????????????????????????????????????????????????????????????????????????????????????????????????????????????????????????????????????????????????????????????????????????????????????????????????????????????????????????????????????????????????????????????????????????????????????????????????????????????????????????????????????????????????????????????????????????????????????????????????????????????????????????????????????????????????????????????????????????????????????????????????????????????????????????????????????????????????????????????????????????????????????????????????????????????????????????????????????????????????????????????????????????????????????????????????????????????????????????????????????????????????????????????????????????????????????????????????????????????????????????????????????????????????????????????????????????????????????????????????????????????????????????????????????????????????????????????????????????????????????????????????????????????????????????????????????????????????????????????????????????????????????????????????????????????????????????????????????????????????????????????????????????????????????????????????????????????????????????????????????????????????????????????????????????-----------------??????????????????????????????????????????????????????????????????????????????????????????????????????????????????????????---????????????????????????????GAGCTTATTTTAATTATGTGCGCCAGCATTCTATTGCTCGTAATGTTGGGGTACAAGTGAATCTAGATTCAACTGAAGGACCAAATGATGTTGGGGTACAAGTGAATCAAGAGTCTGAACAGTCTGTGGATGTTGTTTTTATAGAATTGGAAGGGCGAAGGGTCCCCGTAAG--GGGGGTGA------------------ATCTACATGTTGAGCGGCGT-GGTTATTTGGATAATCG---GTCTAATCAAGAAGG--TGT-TGA---------------------------------------------GAATAGTTTAGGAGATCA-----ATTTGA-AGATGTGGCTTT---TTTAG-GGGGGAG-TACAAGTACACTTACACCAGAAA----GTGTAAATAGGGGAGCTCCAAGTGGTAATATAAGAAATGTATTAAGTGATTCATTAA-TAGA-G-AG-GAGAGGCCAAGACCAAACTTTAAGTGA--AA-TAAAGGTAGAAACACCTCC----AGGTGCTTCAAGGGGTGGCCGATG-A-------G--CCAGT-AT-----AAATCAAGGTGAGGCTTTAAG---AGAAAACAAGGTAGAAATTGGGG----TTTTAAGAGAAAACAAAGT--------AGAAAT--TGATGAGGTAGTTGGCTCGACTTTTAGTTCGATCCCCTTAGAGGTTGAAGGGATGATGGCTCATGTGTGAAATCAATTTGACATGGTGTTTAACATATTGGCCGATAAGCCCCTGGTAGGTTGCA-TTTTTTTTCTTACCAGCTTCTAAGAATAACGAATCAAATGCCAAACCCACGGGTT????????????????????????????????????????????????????????????????????????????????????????????????????????????????????????????????????????????????????????????????????????????????????????????????????????????????????????????????????????????????????????????????????????????????????????????????????????????????????????????????????????????????????????????????????????????????????????????????????????????????????????????????????????????????????????????????????????????????????????????????????????????????????????????????????????????????????????????????????????????????????????????????????????????????????????????????????????????????????????????????????????????????????????????????????????????????????????????????????????????????????????????????????????????????????????????????????????????????????????????????????????????????????????????????????????????????????????????????????????????????????????????????????????????????????????GATC??????????????????????????????????????????????????????TTC-------------------------------------------------------------------------------------------------------------------------------------------------------------------------------------------------------------------------------------------------------------------------------------------------------------ATTAGGGGGTTATGGCCTTTTGCGTTTTTCTTGACCTCTTTTTCCGGGGGCTTCTTTATATTGGTCTCCAGTTATTGTTTTTTTTAGTGTTGTTGCTGTTGTTTATGGAGGTTTAATGACATGTCGTCAAATTGATTTTAAACGGCTTGTTGCTTACTCTTCTGTTGCTCATATGGGACTTGTGCCTTTGGGTCTTTTTACACATGTTATAGAGGGGTTAATTGGGGCTCTTTTTTTAATGTTGGCGCACGGATTTGTTAGCTCTGCTCTTTTTATTGGAGTAACTTTTTTGTATGATCGCCATCATACTCGTTTAATAAAATATTATCGGGGTTTGACTTTGACAATGCCTCTTTTTGTTATTACAATGTTAATTTTGTCTTTGGCGAACATGGGTTTTCCTCTTAGTTGTAATTTTGTTGGAGAGTTTTT??????????????????????????????????????????????????????????????????TTCTGCC???????????????????????TCGTATTTCTTTTGGTGGAGGCTCTAATTATTTACTTTTTAATAGAGACTTAAGTCGACGAGAGATTTTTGTAATTTTTCCTTTTCTTTTAATTATCTTTTTGGGGGGGATT????????????????????????????????????????????????????????????-------------??????????????????????????????????????????????????????????????????????????-??????????????????????????????????????????????????-?????????????????????????????????????????????????????????????????????????????????????????????????????????????????????????????????-????????????????????????????????????-???????????????????????????????????????????????????????????????????--??????????????????????????????????????????????????????------????????????????????????????????????????????????????????????????????????????????????????????????????????????????????????????????-???????????????????????????????????????????????????????????????????????????????????????????????????????????????????????????????????????????????????????????????????????????????????????????????????????????????????????????????????????????????????????????????????????????????????????????????????????????????????????????????????????????????????????????????????????????????????????????????????????????????????????????????????????????????????????????????????????????????????????????????????????????TTATAATTGG------------------------------------------------TACGA-------------------------------------------------------------------------------------------------CTTTTCTTT---------------------------------------------------------------------------------------------------------------------------------------------------------------------------------------------------------------------------------------------------------------------------------------------------------------------------------------------------------------TTATTTG???????????????????????????????????????????????????????????????????????????????????????????????????????????????????????????????????????????????????????????????????????????TTTAGATATTTTATAAATGTTTTGGCTGGGTTGTGTGTTCTCTGGTTTAATCATCTTTATTATTCGGATAGTCCAGAGACTTGACTTTTAG------------------ATCCTATTGTAGAAGAAATTGTTTTTTTTCATGACCAAGTTATGTTTTTATTAATTATTATAGTTACTGTTGTTTTATGACTTTTTGTTGAAGCTTTTAAAAATAAGTTTTATGATCGTCATTTAATCGATGGTACATTTTTAGAAATTGTTTGAACGATTATTCCTGCTGTTATATTGATTTTTATTGCATTGCCCTCTCTTAAATTATTATATTTAATGGATGAGGTTATTTCTCCAGCTTTGACAATTAAAGTTATTGGGCATCAATGATATTGATCRR????????????????????????????????????????????????????????????????????????????????????????????????????????????????????????????????????????????????????????????????????????????????????????????????????????????????????????????????????????????????????????????????????????????????????????????????????????????????????????????????????????????????????????????????????????????????????????????????????????????????????????????????????????????????????????????????????????????????????????????????????????????????????????????????????????GATCTTTTAATAGAACAAATTTTTATGATTATGGGGTTAACTATTGCTGCTGCCGAATCTTCAATTGGTTTAGCTATTTTAGTTGCATATTATAGAATTCGAGGAACAATTGTTTTAAAATCTTTTAGTTCTTTGCGAGGATAATGATTAATCTTTTTATTTTTTTTTTTCTTATTTTTATTTTGGCGGGGTTATTTATATTTCTTTCTTTTTTGATAGGGGAAAAAATTCCAGATC???????????????????????????????????????????????????????????????????????????????????????????????????????????????????????????????????????????????????????????????????????????????????????????????????????????????????????????????????????????????????????????????????????????????????????????????????????????????????????????????????????????????????????????????????????????????????????????????????????????????????????????????????????????????????????????????????????????????????????????????????????????????????????????????????????????????????????????????????????????????????????????????????????????????????????????????????????????????????????????????????????????????????????????????????????????????????????????????????????????????????????????????????????????????????????????????????????????????????????????????????????????????????????????????????????????????????????????????????????????????????????????????????????????????????????????????????????????????????????????????????????????????????????????????????????????????????????????????????????????????????????????????????????????????????????????????????????????????????????????????????????????????????????????????????????????????????????????????????????????????????????????????????????????????????????????????????????????????????????????????????????????????????????????????????????????????????????????????????????????????????????????????????????????????????????????????????????????????????????????????????????????????????????????????????????????????????????????????????????????????????????????????????????????????????????????????????????????????????????????????????????????????????????????????????????????????????????????---------------?????????????????????????????????????????????????????????????????????????????????-????????--??????????????????????????-???????????????????????????????????????-????????????????-------------------------------------??????????-----------ATGGAAGTAGAT------------------------------------------------GAGC---------------------------------------------------------------------------CAAATGA--AGAGATAGTAGGAG--CGCTTCCATCTTTA--------AGCTTCGTGGAGGGGTGAGATG----------------------------AAGAGGTGATAGCAGCGTTTCCATGTTCAGACT----------CCGAAGAGAGTATGGAAATTGATGGGGAAGA--ACTACCTTTGGATCCCCTCCCGATTAGTGGAGCT--ATTAGAGGAGAA--AACATAGTGGCA-------------------------------CCCGTACCAGCCATGGGAGATGCGGGTCTTTCAGTTCATTTTTGGCGTGCACTCAAGAACACTCCATTTGAATTAT-TGGCAGAAGTTTATCGGTCAGGTGAGCAGGGAGAAG--TT---T--ATTCGTC???????---??????????????????????????????????????-??????---------------------??????????????????????????????????????????--???--??????????????????????????-?????????????-???-?????-??????????????????????????????--???????????????????????????????-???????????????????????????????????????CTCTTGCTCTCTAATTTGTAGTTTTTGAGTGGTTTGTATTATTTTTT--TTTTTAATTTTTTCTTTATGAGTCTGTCTTGGTAGAGGAG-GCGATTTTTTTAGAAGCCCCGTCTAAATGCTTTCT????????????CGATGGGTGTTTTCTACAAATCATAAGGACATTGGTACTTTGTATTTAATCTTCGGAGCAGGAGCTGGTTTAATTGGAACTGCTTTTAGTATGCTTATACGATTGGAGCTTTCTGCGCCGGGGGCGATGTTAGGAGATGATCATCTTTATAATGTAATTGTTACAGCACATGCTTTTATTATGATTTTTTTTTTGGTTATGCCGGTCATGATTGGGGGATTTGGTAATTGATTAGTCCCATTATATATTGGGGCGCCGGATATGGCGTTTCCTCGATTAAACAATATTAGTTTTTGACTTTTGCCTCCTGCGCTTTTTTTATTATTAGGCTCTGCTTTTATTGAACAAGGGGCGGGGACGGGGTGAACAGTTT?????CCTCTTTCTAGTATTCAAGCACACTCCGGAGGTTCTGTTGATATGGTTATTTTTAGTCTTCATTTAGCTGGGGTTTCTTCTATTTTAGGTGCTATTAACTTTATTACTACAATTTTTAATATGCGAGCCCCGGGTGTGTCTTTTAATAAACTACCTTTATTTGTTTGATCTATTTTAATAACAGCTTTTTTATTACTTTTATCTTTACCTGTTTTAGCTGGTGCTATTACTATGTTGTTAACAGATAGAAACTTTAATACGACTTTTTTCGATCGATCGGGTGGCGGGGACCCAATATTATTTCAGCATTTATTTTGATTCTTTGGGCATCCAGAAGTTTATATTTTAATTTTGCCTGGTTTTGGTATGATTTCTCAAATAATCCCGACTTTTGTTGCTAAAAAACAAGTTTTTGGGTATTTAGGAATGGTTTATGCCATGCTTTCTATTGGGCTTCTTGGATTTATTGTTTG?????????????????????????????????????????????????????????????????????????????????????????????????????????????????????????????????????????????????????????????????????????????????????????????????????????????????????????????????????????????????????????????????????????????????????????????????????????????????????????????????????????????????????????????????ATGAATTTTTTGGGAAAGTTCATTTTTGATTAATGTTTATCGGGGTTAATCTAACTTTTTTCCCTCAACATTTTTTAGGTTTAGCAGGATTTCCAAGACGATATTCGGATTATGCAGATGCTTTTTTGGGTTGAAATTTAATAAGTTCTTTAGGGTCTATTATTTCTATTKTGAKTGTTGTTTGGTTTTTATATATTGTTTTTGATC???????????????????????????????????????????????????????????????????????????????????????????????????????????????????????????????????????????????????ACCGGTTT

R17 ??????????????????TTAGTCATTGGGTTCATGCCCCAACCAAGTGAGTTCGAATCTCTCWCTTACACAAAATAAAAATTAT-AAAAAAAAAAGAGGATA?????????????AAAATTAGACGAKTT??????AAATGGCGTTCAGTTGCTTATTGGTTGTTTCTTCAGAAAAGTGA--TTTTTTTTTATTTGAATTGAAACATCTTATTACTAAGTAAAAATCAAACGAGATWCCGAGAGTAGTGGAGAGYGAAATTGGAGTTGTGCTTSTGGTGGTCATAGATCTTGCTTAAACATTAGTTTATACTGATAATGAAAGTACTGTAAAGGAAAGTTGAAAGAGAGTTGAAAGGAATTTGAATCTTTTATTTTTGAAGTAGCTTTAAAAAAGCGTACCTTTTGTATAATGGGTAAAAGAGATTTATTTGGCATATTTAAAATGGAAAATTTTAAGTAA-TTTTTTTTTAGTCAAATTTCCCGAAACCAAGTGATTTAATCA???????????????????????????????????????????????????????????????????????????????????????????????????????????????????????????-??????????????????????????????????????????????????????????????????????????????????????????????????????????????????????-??????????????????????????????????????????????????????????????????????????????????????????????????????????????????????????????????????????????????????????????????????????????????????????????????-?------------------????????????????????????????????????????????????????????????????????????????-????????????????????????????????????????????????????????????????????????????????????????????????????????????????????????????????????????????????????????????????????????????????????????????????????????????????????????????????????????????????????????????????????????????????????????????????????????????????????????????????????????????????????????????????GGTATGAATGGTATCCCGAAAGTTTTTCTGTCTTAAAAAAATATTCAATGAAATTAAATCTGTAGTGAAGATGCTACATTTTAATTGTTAGACGAGAAGTCCCCATGGAGCTTTACTGWAAGCTTATATATATAATTTTTTTTTTTTTATA?????GACAGTTTTGTTGGGGCGACAGTTTTTTAAAAAGTAACGAAAATGAGCTATGACGCATGTTTAACTTTGAAATTTTTTT-ATTGATGAGACATTTTTGGTGTGTTTTTTGATCCGTTATTTTGAATGAAAAAAATAACGAAAACAAATAAAAGTTACCCTGGGGATAACAGCGCAATAATGTTTGAGAGTTAATCAACGACAGTGTTTGCGACCTCGATGTTGAATTGTAACGTCCTACGGTGTAGTCACTCGTAAGGGTTGGTTTGTTCATCCATTAAAGTTATACATGATTTGAGTTAAAAGCGTGGTGACACAGCTTGGTTTCTATCTACAATTAGAAAACAAAAATATGTTGTTTTTTCGTACGAAAGGATCAAAAATCAATAGTTCTCTTAATATAACTATTCTTTAAATTAGGATTGTTTCTAAAAATAAAAAKAAATATTTTTGTTTGTTGTTTTTATTAATGTATCTTTTAGTTTTATTTTTT???????????????????????????????????????????????????????????????????????????????????????????????????????????????????????????????????TAATTCAACAACAACGTATTTGAAATTATGAAAATGATTTGATTCGGGGTTGTTTGTTGTTTTTTTTGGTTTTCAATTTGATGGTTTAGTAGTTATTATGTTATTTGTTGTTTTTATTGTTTCTACTTTGGTTCATATTTTTTCTATTGCTTATATGCGGGGAGATCCTCATATTCCTCGATTTATGACATATCTTTCTTTGTTTACTTTTTTAATGGTTTTATTGGTAACTAGCGATAATTTTCTTCAATTATTTATTGGGTGAGAAGGGGTTGGTCTTTGTTCTTATTTATTAATTAATTTTTGATTAACTCGATTAGAGGCAAATAGAGCTGCTATTAAAGCAATGTTAGTGAATAGAATTGGTGATATTGGGTTGCTTTTAGCAATGTTTTTACTTTGGGATCTTTTTGGATC????????????????????????????????????????????????????????????TTTATTTTTATTTTTTGGGGTTATGGGAAAATCTGCTCAATTAGGATT????????????????????????????????????????????????????????????????????????????????????????????????????????????????????????????????????????????????????????????????????????????????????????????????????????????????????????????????????????????????????????????????????????????????????????????????????????????????????????????????????????????????????????????????????????????????????????????????????????????????????????????????????????????????????????????????????????????????????????????????????????TTGGCTATTTCTTCTATTAGTGTTTATGCAATTTTAATGTCTGG??????????????????????????????????????????????????????????????????????????????????????????????????????????????????????????????????????????????????????????????????????????????????????????????????????????????????????????????????????????????????????????????????????????????????????????????????????????????????????????????????????????????????????????????????????????????????????????????????????????????????????????????????????TTGGTTKTAAAGTTGTTTTTATTATTTTTTTTTTTAK??????????????????????????????????GATCAGCTTATGGCTTTGTTATGAAAAGGGTATTTGCCTTTAAGTTTAGGAATGGTCATCTTTGTGGCTAGTGTTCTCTTTGGGTTTAATGGTCCCCCTCCTATTTAAAATATTTTAATTAATTATTATGAAAAAAAAAAAGAACTAACAGATTTGCAAGTGCCATTACGAAAAAAAAATCCTATTTTATTTTTAATCAACGGATTTTTAGTTGATTTGGTTTCTCCTTCTAACATTACTTATTTATGAAATTTTGGATC???????????????????????????????????????????????????????????????????????????????????????????????????????????????????????????????????????????????????????????????????????????????????????????????????????????????????????????????????????????????????????????????????????????????????????????????????????????????????????????????????????????????????????????????????????????????????????????????????????????????????????????????????????????????????????????????????????????????????????????????????????????????????????????????????????????????????????????????????????????????????????????????????????????????????????????????????????????????????????????????????????????????????????????????????????????????????????????????????????????????????????????????????????????????????????????????????????????????????????????????????TTTTATCTATTTTACATCAAAGTCTTTTAAAGGGACTTTTTTTTCGTCCTTTAGGTCGAATTGCCTTTTGGTTTTTAATTATTGATTTTGCTTTATTAAC???????KGATCACAAGTTGTAGAAGAACCTTTTATTTTAATTGGTCAAATACTTTCTTTTTTTTATTTTTTTTATTTTTTAGTTTTAATACCAGTTTTGGGTATTATTGAAAATCAATT??????????????????????????????????????????????????????????????????????????????????????????????????????????????????????????????????????????????????????????????????????????????????????????????????????????????????????????????????????????????????????????????????????????????????????????????????????????????????????????????????????????????????????????????????????????????????????????????????????????????????????????????????????????????????????????????????????????????????????????????????????????????????????????????????????????????????????????????????????????????????????????????????????????????????????????????????????????????????????????????????????????????????????????????????????????????????????????????????????????????????????????????????????????????????????????????????????????????????????????????????????????????????????????????????????????????????????????????????????????????????????????????????????????????????????????????????????????????????????????????????????????????????????????????????????????????????????????????????????????????????????????????????????????????????????????????????????????????????????????????????????????????????????????????????????????????????????????????????????????????????????????????????????????????????????????????????????????????????????????????????????????????????????????????????????????????????????????????????????????????????????????????????????????????????????????????????????????????????????????????????????????????????????????????????????????????????????????????????????????????????????????????????????????????????????????????????????????????????????????????????????????????????????????????????????????????????????????????????????????????????????????????????????????????????????????????????????????????????????????????TATGTTTCTTGTTTTTGTTTATTTCTTTTGGCCAGTTTTATTTTATTAGTYGCTATGATGGGTGTGATTGTATTAACTCAAGAAA???????????????????????????????????????????????????????????????????????????????????????????????????????????????????????????????????????????????????????????????????????????????????????????????????????????????????????????????????????????????????????????????????????????????????????????????????????????????????????????????????????????????????????????????????????????????????????????????????????????????????????????????????????????????????????????????????????????????????????????????????????????????????????????????????????????????????????????????????????????????????????????????????????????????????????????????????????????????????????????????????????????????????????????????????????????????????????????????????????????????????????-----------------??????????????????????????????????????????????????????????????????????????????????????????????????????????????????????????---????????????????????????????????????????????????????????????????????????????????????????????????????????????????????????????????????????????????????????????????????????????????????????????????????????--????????------------------????????????????????-?????????????????---???????????????--???-???---------------------------------------------?????????????GATCA-----ATTTGA-AGATGTGGCTTT---TTTAA-GAGAGAG-TACAAGTACAATTACACCAGAAA----GTGTAAATAGGGGCGCTCCAAGTGGTAATATAAGAAATGTATTAAGTGATTCATTAA-TAGA-G-AA-GAGAGGCCAAGACCAAACTTTAAGTGA--AA-TAAAGGTAGAAACACCTCC----AGGTGCTTCAAGGGGTGGCCGATG-A-------G--CCAGT-AT-----AAATCAAGGTGAGGCTTTAAG---AGAAAACAAGGTAGAAATTGGGG----TTTTAAGAGAAAACAAAGT--------AGAAAT--TGATGAGGTAGTTGGCTCGACTTTTAGTTCGATC???????????????????????????????????????????????????????????????????????????????????????????????????????????????????????????????????????????????????????????????????????????????????????????????????????????????????????????????????????????????????????????????????????????????????????????????????????????????????????????????????????????????????????????????????????????????????????????????????????????????????????????????????????????????????????????????????????????????????????????????????????????????????????????????????????????????????????????????????????????????????????????????????????????????????????????????????????????????????????????????????????????????????????????????????????????????????????????????????????????????????????????????????????????????????????????????????????????????????????????????????????????????????????????????????????????????????????????????????????????????????????????????????????????????????????????????????????????????????????????????????????????????????????????????????????????????????????????????????????????????????????????????????????????GATC?????????TTTATCTTTTTTTTGAGGGGATATTAATACCAATGTTTTTTTTA??????????????????????????????????????TTCTTTTTATTTTTTTTTTTTTACTTTTATAGG????????????????????????????????????????????????????????????????????????????????????????????????????????????????????????????????????????????????????????????????????????????????????????????????????????????????????????????????????????????????GGGTTATGGCCTTTTGCGTTTTTCTTGACCTCTTTTTCCGGGGGCTTCTTTATATTGGTCTCCAGTTATTGTTTTTTTTAGTGTTGTTGCTGTTGTTTATGGAGGTTTAATG??????????????????????????????GTTGCTTACTCTTCTGTTGCTCATATGGGACTTGTGCCTTTGGGTCTTTTTACACATGTTATAGAGGGGTTAATTGGGGCTCTTTTTTTAATGTTGGCGCACGGATTTGTTAGCTCTGCTCTTTTTATTGGAGTAACTTTTTTGTATGATCGCCATCATACTCGTTTAATAAAATATTATCGGGGTTTGACTTTGACAATGCCTCTTTTTGTTATTACAATGTTAATTTTGTCTTTGGCGAACATGGGTTTTCCTCTTAGTTGTAATTTTGTTGGAGAGTTTTTTTCTTTATTAGCAGTCTTTCAATATCATTATGGAGTTGGAATGTTTGTTATTTTAGGGGTTC??????????????????????????????????????????????????????????????????????????????????????????????????????????????????????????????????????????????????????????????????????????????????????????????????????????????-------------??????????????????????????????????????????????????????????????????????????-??????????????????????????????????????????????????-?????????????????????????????????????????????????????????????????????????????????????????????????????????????????????????????????-????????????????????????????????????-???????????????????????????????????????????????????????????????????--??????????????????????????????????????????????????????------????????????????????????????????????????????????????????????????????????????????????????????????????????????????????????????????-??????????????????????????????????????????????????????????????????????????????????????????????????????????????????????????????????????????????????????????????????????????????????????????????????????????????????????????????????????????????????????????????????????????????????????????????????????????????????????????????????????????????????????????????????????????????????????????????????????????????????????????????????????????????????????????????????????????????????????????????????????????????????????????????????????????????????????????????????????????????????????????????????????????????????????????????????????????????????????????????????????????????????????????????????????????????????????????????????????????????????????????????????????????????????????????????????????????????????????????????????????????????????????????????????????????????????????????????????????????????????????????????????????????????????????????????????????????????????????????????????????????????????????????????????????????????????????????????????????????????????????????????????????????????????????????????????????????????????????????????????????????????????????????????AAAATTTCTTTAATGCAAAATCTTTTTGTTTTTACTTTTTTTAGATATTTTATAAATGTTTTGGCTGGGTTGTGTGTTCTCTGGTTTAATCATCTTTATTATTCG?????????????????????????????????????????GGATCCTATTGTAGAAGAAATTGTTTTTTTTCATGACCAAGTTATGTTTTTATTAATTATTATAGTTACTGTTGTTTTATGACTTTTTGTTGAAGCTTTTAAAAATAAGTTTTATGATCGTCATTTAATCGATGGTACATTTTTAGAAATTGTTTGAACGATTATTCCTGCTGTTATATTGATTTTTATTGCATTGCCCTCTCTTAAATTATTATATTTAATGGATGAGGTTATTTCTCCAGCTTTGACAATTAAAGTTATTGGGCATCAATGATATTGATC??????????????????????????????????????????????????????????????????????????????????????????????????????????????????????????????????????????????????????????????????????????????????????????????????????????????????????????????????????????????????????????????????????????????????????????????????????????????????????????????????????????????????????????????????????????????????????????????????????????????????????????????????????????????????????????????????????????????????????????????????????????????????????????????????????????GATCTTTTAATAGAACAAATTTTTATGATTATGGGGTTAACTATTGCTGCTGCCGAATCTTCAATTGGTTTAGCTATTTTAGTTGCATATTATAGAATTCGAGGAACAATTGTTTTAAAATCTTTTAGTTCTTTGCGAGGATAATGATTAATCTTTTTATTTTTTTTTTTCTTATTTTTATTTTGGCGGGGTTATTTATATTTCTTTCTTTTTTGATAGGGGAAAAAATTCCAGATCGAGAAAAGGTTTCTGCTTATGAGTGTGGTTTTGCTCCCTTTAATTTTTTAGGACGCCCTTTTTCAATACGTTTTTTTTTAATTGGTATTTTATTCTTGATTTTTGATT???????????????????????????????????????ATTCGACTGCTCCGTTTGGGTTTTGAACTATGATAGGTTTTTTTTTTGTATTAGTTTTGGGTTTGATATATGAATGAGWAATGGGGGGATTAGA??????????????????????????????????????????????????????????????????????????????????????????????????????????????????????????????????????????????????????????????????????????????????????????????????????????????????????????????????????????????????????????????????????????????????????????????????????????????????????????????????????????????????????????????????????????????????????????????????????????????????????????????????????????????????????????????????????????????????????????????????????????????????????????????????????????????????????????????????????????????????????????????????????????????????????????????????????????????????????????????????????????????????????????????????????????????????????????????????????????????????????????????????????????????????????????????????GGTCTTTTCAAATAGATGTTTTTWCAATACT?????????????????????????????????????????????????????????????????TTTTTTTTTTTTTTTCTTCTCAAATTTTTGGTTATCCCCTTCAGGTGATC????????????????????????????????????????????????????????????????????????????????????????????????????????????????????????????????????????????????????????????????????????????????????????????????????????????????????????????????????????????????????????????????????????????????????????????????????????????????????????????????????????????????????????????????????????????????????????????????????????????????????????????????????????????????????????????????AATCTTTCCTTTYGTAAAGCGTGGT???TKMATAAGACAGTTCTTATTGGGGTG---------------AACCTTTTTTTTAGGGTGAAAAAAAAATTATGGAAAAAGATTTCCATGAGAACTGTCGCAAAATGTTTAAAAAAAGCCTTT-TTTTTGCT--AGTGGGATTTTGTTTAAAATATTTTT-TTGAGTTTTCTTACTTTAAAACATCTTTTTGTTTA????-??TTTTTTTTTTTTAT-------------------------------------AAAATTTGGT------------TCTAAGAAGATTC----------------------------------------------GGGCTCGTTTAGCGATC?????---????-------???---??????????????--?????????????GATGAG-CCARATGA--AGAGATAGTAGGAG--CGYTTCCATCTTTA--------AGCTTCGTGGAGGGGTGAGATG----------------------------AAGAGGTGATAGCAGCGTTTCSATGT???????----------???????????????????????????????--??????????????????????????????????--????????????--????????????-------------------------------????????????????????????????????????????????????????????????????????????????-????????????????????????????????????--??---?--??????????????---??????????????????????????????????????-??????---------------------??????????????????????????????????????????--???--??????????????????????????-?????????????-???-?????-??????????????????????????????--???????????????????????????????-??????????????????????????????????????????????????????????????????????????????????????--????????????????????????????????????????-?????????????????????????????????????????????????????????????????????????????????????????????????????????????????????????????????????????????????????????????????????????????????????????GATCATCTTTATAATGTAATTGTTACAGCACATGCTTTTATTATGATTTTTTTTTTGGTTATGCCGGTCATGATTGGGGGATTTGGTAATTGATTAGTCCCATTAWATATTGGGGCGCCGGATAT?????????????????????????????????????????????????????????????????????????????????????????????????????????????????????????????????????????????????????????????????????????????TTCATTTAGCTGGGGTTTCTTCTATTTYAGGTGCTATTAACTTTATTACTACAATTTTTAATATGCGAGCCCCGGGTGTGTCTTTTAATAAACTACCTTTATTTATCTGATCTATTTTAATAACAGCTTTTTTATTACTTTTATCTTTACCTGTTTTAGCTGGTGCTATTACTATGTTGTTAACAGATAGAAACTTTAATACGACTTTTTTCGATCAGAT??????????????????????????????????????????????????????????GTTTATATTTTAATTTTGCCTGGTTTTGGTATGATTTCTCAAATAATCCCGACTTTTGTTGCTAAAAAACAAGTTTTTGGGTATT?????ATGGTTTATGCCATGCTTTCTATTGGGCTTCTTGGATTTATTGTTTKAGCTCATCATATGT???????????????????????????????????????????????????????????????????????????????????????????????????????????????????????????????????????????????????????????????????????????????????????????????????????????????????????????????????????????????????????????????????????????????????????????????????????????????????????????????????????????????????????????????????????????????????????????????????????????TTTTTCCCTCAACATTTTTTAGGTTTAGCAGGATTTCCAAGACGATATTCGGATTATGCAGATGCTTTTTTGGGTTGAAATTTAATAAGTTCTTTAGGGTCTATTATTTCTATTTTGAGTGTTGTTTGGTTTTTATATATTGTTTTTGATC??????????????????????????????????????????????????????????????????????????????????????????????????????????ACAAAAAGTAAATAATTTTTAGAATAGTGCCGGGTTTATAT????????

SD2 TGTAAGAAAGACAAAGGGTTAGTCATTGGGTTCATGCCCCAACCAAGTGAGTTCGAATCTCTCTCTTACACAAAATAAAAATTAT-AAAAAAAAAAGAGGATAACTAAGATAAACTAAAATTAGACGATTTTTTTCGAAATGGCGTTCAGTTGCTTATTGGTTGTTTCTTCAGAAAAGTGA--TTTTTTTTTATTTGAATTGAAACATCTTATTACTAAGTAAAAATCAAACGAGATTCCGAGAGTAGTGGAGAGTGAAATTGGAGTTGTGCTTGTGGTGGTCATAGATCTTGCTTAAACATTAGTTTATACTGATAATGAAAGTACTGTAAAGGAAAGTTGAAAGAGAGTTGAAAGGAATTTGAATCTTTTATTTTTGAAGTAGCTTTAAAAAAGCGTACCTTTTGTATAATGGGTAAAAGAGATTTATTTGGCAT?????????????????????????-?????????????????????????????????????????????????????????????????????????????????????????????????????????????????????????????????????????????????????????????????????-??????????????????????????????????????????????????????????????????????????????????????????????????????????????????????-???????????????????????????????????????????????????????????????????????????????????????????????????????????????????????????????????????????????????????????????????????????GCAGAATAAATTGTTTTCGATAT-C------------------TCCCTAGTTTGAAATTTTTTTTCTTAATACATCGTGTTCAGTTGTAATAATTTTTACATAAGTAATCTAAATATTA-TTTTTT?????????????????????????????????????????????????????????????????????????????????????????????????????????????????????????????????????????????????????????????????????????????????????????????????????????????????????????????????????????????????????????????????????????????????????????????????????????????????????????????????????????????????????????????????????????????????????????????????????????????????????????AGTGAAGATGCTACATTTTAATTGTTAGACGAGAAGTCCCCATGGAGCTTTACTGTAAGCTTATATATATAATTTTTTTTTTTTTATAAGTAAGACAGTTTTGTTGGGGCGACAGTTTTTTAAAAAGTAACGAAAATGAGCTATGACGCATGTTTAACTTTGAAATTTTTTT-ATTGATGAGACATTTTTGGTGTGTTTTTTGATCCGTTATTTTGAATGAAAAAAATAACGAAAACAAATAAAAGTTACCCTGGGGATAACAGCGCAATAATGTTTGAGAGTTAATCAACGACAGTGTTTGCGACCTCGATGTTGAATTGTAACGTCCTACGGTGTAGTCACTCGTAAGGGTTGGTTTGTTCATCCATTAAAGTTATACATGATTTGAGTTAAAAGCGTGGTGACACAGCTTGGTTTCTATCTACAATTAGAAAACAAAAATATGTTGTTTTTTCGTACGAAAGGATCAAAAATCAATAGTTCTCTTAATATAACTATTCTTTAAATTAGGATTGTTTCTAAAAATAAAAAGAAATATTTTTGTTTGTTGTTTTTATTAATGTATCTTTTAGTTTTATTTTTTCCTTTAATTGGAGCTGTTTTAACAGGTTGTTTTGGAAGAAAAATCGGAGAAAGAGGAGCGGGGATTTTAACTTCAAGTTGTTTAGTTTTTAGTTTATCTTATTCTTTTTTAATCGCGATTGAAGTTTTATTTAATTCAACAACAACGTATTTGAAATTATGAAAATGATTTGATTCGGGGTTGTTTGTTGTTTTTTTTGGTTTTCAATTTGATGGTTTAGTAGTTATTATGTTATTTGTTGTTTTTATTGTTTCTACTTTGGTTCATATTTTTTCTATTGCTTATATGCGGGGAGATCCTCATATTCCTCGATTTATGACATATCTTTCTTTGTTTACTTTTTTAATGGTTTTATTGGTAACTAGCGATAATTTTCTTCAATTATTTATTGGGTGAGAAGGGGTTGGTCTTTGTTCTTATTTATTAATTAATTTTTGATTAACTCGATTAGAGGCAAATAGAGCTGCTATTAAAGCAATGTTAGTGAATAGAATTGGTGATATTGGGTTGCTTTTAGCAATGTTTTTACTTTGGGATCTTTTTGGATCTCTAGATTTTTCTACTATTTTTAATTCTATTTTTTTTTCTAATCAAATATTTTTTATTTGTTTATTTTTATTTTTTGGGGTTATGGGAAAATCTGCTCAATTAGGATTACATACTTGGTTACCGGATGCAATGGAAGGTTATTGGGCCTTTTAATTAAAAAATTAATTAAAAAAATTACTATACATTAAA?????????????????????????????????????????????????????????????????????????????????????????????????????????????????????????????????????????????????????????????????????????????????????????????????????????????????????????????????????????????????????????????????????????????????????????????????????????????????????????????????????????????????????????????????????????????????????????????????????????????????????????????????????????????????????????????????????????????????????????????????????????????????????????????????????????????????????????????????????????????????????????????????????????????????????????????????????????????????????????????????????GTTTTTTGCTTCGATTTTAGCAGAGACCAATCGTGCTCCTTTTGATTTAACAGAAGGAGAGTCAGAGCTTGTTTCGGGATATAATGTTGAGTATGCTTCGATGTCTTTTGCTTTATTTTTTCTTGCTGAATATGCCCATATTATTTTAATGAGTTGTTTAACAACGATTTTTTTTGGGGGAGGATGGCTTTCTCCGATTCCAGATTTTAAGGGTGGGGCGGGCTGGTTTGGTTTTAAAGTTGTTTTTATTATTTTTTTTTTTATTTGAGTAAGGGCTTCATTTCCTCGAATTCGATATGATCAGCTTATGGCTTTGTTATGAAAAGGGTATTTGCCTTTAAGTTTAGGAATGGTCATCTTTGTGGCTAGTGTTCTCTTTGGGTTTAATGGTCCCCCTCCTATTTAAAATATTTTAATTAATTATTATGAAAAAAAAAAAGAACTAACAGATTTGCAAGTGCCATTACGAAAAAAAAATCCTATTTTATTTTTAATCAACGGATTTTTAGTTGATTTGGTTTCTCCTTCTAACATTACTTATTTATGAAATTTTGGATCTTTATTGGGATTGTGTTTGATTTTACAGATAGTGACTGGGTGTTTTTTGTCTATGCACTATTGTTCTGATGTTAATTTTGCTTTTGCTTCAATTGGTCATATTATGCGAGATGTTAATTATGGGTTTTTATTAAGATATCTTCATGCTAACGGTGCTT???????????????????????????????????????????????????????????????????????????????????????????????????????????????????????????????????????????????????????????????????????????????????????????????????????????????????????????????????????????????????????????????????????????????????????????????????????????????????????????????????????????????????????????????????????????????????????????????????????????????????????????????????????????????????????????????????????????????????????????????????????????????????????????????????????????????????????????????????????????????????????????????ATATTACGTTCTATACCAAATAAATTAGGTGGGGTTATTGCAATGTTTTGTAGTATTTTTATTTTATTTTTTTTATCTATTTTACATCAAAGTCTTTTAAAGGGACTTTTTTTTCGTCCTTTAGGTCGAATTGCCTTTTGGTTTTTAATTATTGATTTTGCTTTATTAACTTGAATTGGATCACAAGTTGTAGAAGAACCTTTTATTTTAATTGGTCAAATACTTTCTTTTTTTTATTTTTTTTATTTTTTAGTTTTAATACCAGTTTTGGGTATTATTGAAAATCAATTATTAAATAAAAATTAATGAAATTTTTGTTTTAGGCGGTTTTCTTTTGAGCTTCGTGGTTTTAAGTCTTCGTTATTTTCTTT??????????????????????????????TTTTTTTTTTTTTTTTTTTTAAATTAGAAT?????????????????????????????????????????????????????????????????????????????????????????????????????????????????????????????????????????????????????????????????????????????????????????????????????????????????????????????????????????????????????????????????????????????????????????????????????????????????????????????????????????????????????????????????????????????????????????????????????????????????????????????????????????????????????????????????????????????????????????????????????????????????????????????????????????????????????????????????????????????????????????????????????????????????????????????????????????????????????????????????????????????????????????????????????????????????????????????????????????????????????????????????????????????????????????????????????????????????????????????????????????????????????????????????????????????????????????????????????????????????????????????????????????????????????????????????????????????????????????????????????????????????????????????????????????????????????????????????????????????????????????????????????????????????????????????????????????????????????????????????????????????????????????????????????????????????????????????????????????????????????????????????????????????????????TAATGGTTGTTTCAGCGTTAAATCCTGTTCTTTCTATTTTTTGATTGGTTCTTGTTTTTATAAATTCTGCCGTTTTTTTCCTTTTACTAGGAATAGATTTTCTTGCTT??????????????????????????????????????????????????????????????????????????????????????????????????????????????????????????????????????????????????????????????????????????????????????????????????????????????TGAGACCTTTCTTTTCCTTGATTT???ATTTCTTATCATAATATTGAGGCCTTAGGGCAGATTTTATATGTTTCTTGTTTTTGTTTATTTCTTTTGGCCAGTTTTATTTTATTAGTTGCTATGATGGGTGTGATTGTATTAACTCAAGAAACAGAATCTTTAAGTAAAAAACAAGATCTTTTTTTCCAAATAAATAGGTAATGAACAGTTCTTCTTATTTTGAACAATTTAACATAGTGTGATTGTTTGGTTTTACGAATTCAACAATAATGATGACTTTTGTAATTATTGTAGTTTTATTATTTTTTAAAGGAATTGAATTAATTCCAAAAAGATGGCAGTCGGTTTATGAATGTCTAGAAAATTATTTTTATTATATAACAGTGCAAAATTTAAGT????????????????????????????????????????????????????????????????????????????????????????????????????????????????????????????????????????????????????????????????????????????????????????????????????????????????????????????????????????????????????????????????????????????????????????????????????????????????????????????????????????????????????????????????????????????????????????????????????????????????????????????????????????????????????????????????????????????????????????????????-----------------??????????????????????????????????????????????????????????????????????????????????????????????????????????????????????????---???????????????????????????????????????????????????????????????????????????????????????????????????????????ACCAAATGATGTTGGGGTACAAGTGAATCAAGAGTCTGAACAGTCTGTGGATGTTGTTTTTATAGAATTGGAAGGGCGAAGGGTCCCCGTAAG--GGGGGTGA------------------ATCTACATGTTGAGCGGCGT-GGTTATTTGGATAATCG---GTCTAATCAAGAAGG--TGT-TGA---------------------------------------------GAATAGTTTAGGAGATCA-----ATTTGA-AGATGTGGCTTT---TTTAG-GGGGGAG-TACAAGTACACTTACACCAGAAA----GTGTAAATAGGGGCGCTCCAAGTGGTAATATAAGAAATGTATTAAGTGATTCATTAA-TAGA-G-AG-GAGAGGCCAAGACCAAACTTTAAGTGA--AA-TAAAGGTAGAAACACCTCC----AGGTGCTTCAAGGGGTGGCCGATG-A-------G--CCAGT-AT-----AAATCAAGGTGAGGCTTTAAG---AGAAAACAAGGTAGAAATTGGGG----TTTTAAGAGAAAACAAAGT--------AGAAAT--TGATGAGGTAGTTGGCTCGACTTTTAGTTCGATCCCCTTAGAGGTTGAAGGGATGATGGCTCATGTGTGAAATCAATTTGACATGGTGTTTAACATATTGGCTGATAAGCCCCTGGTAGGTTGCATTTTTTTTTCTTACCAGCTTCTAAGAATAACGAATCAAATGCCAAACCCACGGGTTTATAGGTGATTTGCTTTGGTGTATCTTTTATTTATTTTTGTTCTTTTAATGGTTTAGCTGTTTGTACGTAG????????????????????????????????????????????????????????????????????????????????????????????????????????????????????????????????????????????????????????????????????????????????????????????????????????????????????????????????????????????????????????????????????????????????????????????????????????????????????????????????????????????????????????????????????????????????????????????????????????????????TTTGAATTAGTTAAAAATCAATAAAATTTTTATTTAAAGAATTTTTTTTTGTGTTT????????????????????????????????????????????????????????????????????????????????????????????????????????GTGGTCTTTGGCTTTATTGTTTAGTACTTTAGTTTTTTGGGGTGGATTTGACGGAGAAAGTCATTTTCAATTTTTTAGTTTAGTAGAATGAAATATATTTTCTACTTTAGATTGAGGCCCGATTGTTTTTGCAGTTGATGGTGTTTCTTTGGTTTTTTTACTTTTAACGACTTTTTTAATTCCAATTTGTATTTTAATTAGCCAAAAATCAATAAAATTCTTATTTAAAGAATTTCTTTTGTGCTTGTTTTTTTTAGAATTTTTATTAATAGGTGTTTTCATAGTGTTTGATCTTCTTTTGTTTTATCTTTTTTTTGAGGGGATATTAATACCAATGTTTTTTTTAATTGGTATTTGAGGCTCTCGAGAAGAAAAGGTTCGTGCTTCTTTTTATTTTTTTTTTTTT???????TAGGCTC????????????????????????????????????????????????????????????????????????????????????????????????????????????????????????????????????????????????????????????????????????????????????????????????????????????????????????????????????????????????????????????????????????????????????????????????????????????????ATTGTTTTTTTTAGTGTTGTTGCTGTTGTTTATGGAGGTTTAATGACATGTCGTCAAATTGATTTTAAACGGCTTGTTGCTTACTCTTCTGTTGCTCATATGGGACTTGTGCCTTTGGGTCTTTTTACACATGTTATAGAGGGGTTAATTGGGGCTCTTTTTTTAATGTTGGCGCACGGATTTGTTAGCTCTGCTCTTTTTATTGGAGTAACTTTTTTGTATGATCGCCATCATACTCGTTTAATAAAATATTATCGGGGTTTGACTTTGACAATGCCTCTTTTTGTTATTACAATGTTAATTTTGTCTTTGGCGAACATGGGTTTTCCTCTTAGTTGTAATTTTGTTGGAGAGTTTTTTTCTTTATTAGCAGTCTTTCAATATCATTATGGAGTTGGAATGTTTGTTATTTTAGGGGTTCTTTTTTCTGCCATTT???????????????????????????????????????????????????????????????????????????????????????????????????????????????????????????????????????????????????????????????????????????????????????????????-------------??????????????????????????????????????????????????????????????????????????-??????????????????????????????????????????????????-?????????????????????????????????????????????????????????????????????????????????????????????????????????????????????????????????-????????????????????????????????????-???????????????????????????????????????????????????????????????????--??????????????????????????????????????????????????????------????????????????????????????????????????????????????????????????????????????????????????????????????????????????????????????????-?????????????????????????????????????????????????????????????????????????????????????????????????????????????????????????????????????????????????????????????????????????????????????????????????????????????????????????????????????????????????????????????????????????????????????????????????????????????????????????????????????????????????????????????????????????????????????????????????????????????????????????????????????????????????????????????????????????????????????????????????????????????????????????????????????????????????????????????????????????????????????????????????????????????????????????????????????????????????????????????????????????????????????????????????????????????????????????????????????????????????????????????????????????????????????????????????????????????????????????????????????????????????????????????????????????????????????????????????????????????????????????????????????????????????????????????????????????????????????????????????????????????????????????????????????????????????????????????????????????????????????????????????????????????????????????????????????????????????????????????????????????????????????????????????????????????????????????????????????????????????????????????????????????????????????????????????????????????????----------------------------------TCCTATTGTAGAAGAAATTGTTTTTTTTCATGACCAAGTTATGTTTTTATTAATTATTATAGTTACTGTTGTTTTATGACTTTTTGTTGAAGCTTTTAAAAATAAGTTTTATGATCGTCATTTAATCGATGGTACATTTTTAGAAATTGTTTGAACGATTATTCCTGCTGTTATATTGATTTTTATTGCATTGCCCTCTCTTAAATTATTATATTTAATGGATGAGGTTATTTCTCCAGCTTTGACAATTAAAGTTATTGGGCATCAATGATATTGATC??????????????????????????????????????????????????????????????????????????????????????????????????????????????????????????????????????????????????????????????????????????????????????????????????????????????????????????????????????????????????????????????????????????????????????????????????????????????????????????????????????????????????????????????????????????????????????????????????????????????????????????GGGGAGTTGGGGTATAATTTTAAATAGAGGACATTTTATTATTATGCTTG???????????????????????????????TTTTTTTTTTTTAATAAGTTCTAAAGAAATAGATCTTTTAATAGAACAAATTTTTATGATTATGGGGTTAACTATTGCTGCTGCCGAATCTTCAATTGGTTTAGCTATTTTAGTTGCATATTATAGAATTCGAGGAACAATTGTTTTAAAATCTTTTAGTTCTTTGCGAGGATAATGATTAATCTTTTTATTTTTTTTTTTCTTATTTTTATTTTGGCGGGGTTATTTATATTTCTTTCTTTTTTGATAGGGGAAAAAATTCCAGATCGAGAAAAGGTTTCTGCTTATGAGTGTGGTTTTGCTCCCTTTAATTTTTTAGGACGCCCTTTTTCAATACGTTTTTTTTTAATTGGTATTTTATTCTTGATTTTTGATTTAGAAATTTCTTTTTTTTTTCCCTGATGTGTTTTATATAATTCGACTGCTCCGTTTGGGTTTTGAACTATGATAGGTTTTTTTTTTGTATTAGTTTTGGGTTTGATATATGAATGAGTA???????????????????????????????????????????????????????????????????????????????????????????????????????????????????????????????????????????????????????????????????????????????????????????????????????????????????????????????????????????????????????????????????????????????????????????????????????????????????????????????????????????????????????????????????????????????????????????????????????????????????????????????????????????????????????????????????????????????????????????????????????????????????????????????????????????????????????????????????????ATTTGCTTTTGGACAATTTTATTTAATTTTTGTGTATTGATTAGGTTGTTTTTCTGTTTTATTGACAATTATATACTCTATTCGTTTAATTTATTTAGTTTTTTTATCCAATATTAATTTAAAACGAGCAAACATTTTTTTTCTTAAAGAAGGAGAATTTTTATTTTTAATTCCTTTGGGCATATTAACTTTAGGAAGTGTTTTTTGGGGCTATTTAAGTAAAGAAATAATTTGGTCTTTTCAAATAGATGTTTTTTCAATACTTTCTTTAAAAATAAAAATATTTCCAATTTTATTTTGTTTTATTGGACTTTTTGGGACGATATTTTTTTTTTTTTTTTTTTCTTCTCAAATTTTTGGTTATCCCCTTCAGGTGATCGGATCTTCTGTTTTTTCTCTTTATAATTTTTTTGGTTCTGCTTGACAAATAAATTTTTTTTTTAATTTTTTCTTTATAAAAAAAATATATAAAATAGGACATCTTATTACTA???????????????????????????????????????????????????????????????????????????????????????????????????????????????????????????????????????????????????????TTTTGCACTTTAATTAAAATTTTTGAAGGGGTTAAGTTAAGCAAAACAGTTGACTTTCAAAGTTAATTATGTAGGTGGAAATCTTACACTCTTTGGATGCCACAGTTAGAGG????????????????????????????????????????????????????????????TTTTAGAAGTTATAATCTTTCCTTTTGTAAAGCGTGGTTGATGAATAAGACAGTTCTTATTGGGGTG---------------AACCTTTTTTTTAGGGTGAAAAAAAAATTATGGAAAAAGATTTCCATGAGAACTGTCGCAAAATGTTTAAAAAAAGCCTTT-TTTTTGCT--AGTGGGATTTTGTTTAAAATATTTTT-TTGAGTTTTCTTACTTTAAAACATCTTTTTGTTTAATGA-TTTTTTTTTTTTTTAT-------------------------------------AAAATTTTGT------------TCTAAGAAGATTC----------------------------------------------GGGCTCGTTTAGCGATC?????---??????GATC-TTT---GGGGATGGTGTGGA--AAAAATGGAAGTAGATG-------ATGA--AGAGATAGTAGGAG--CGTTTCCATCTTTA--------AGCTTCGTGGAGGGGTGAGATG----------------------------AAGAGGTGATAGCAGCGTTTCCATGTTCAGACT----------CCGAAGAGAGTATGGAAATTGATGGGGAAGA--ACTACCTTTGGATCCCCTCCCGATTAGTGGAGCT--ATTAGAGGAGAA--AACATAGTGGCA-------------------------------CCCGTACCAGTCATGGGAGATGCGGGTCTTTCAGTTCATTTTTGGCGTGCACTCAAGAACACTCCATTTGAATTAT-TGGCAGAAGTTTATCGGTCAGGTGAGGAGGGAGAAG--TT---T--??????????????---??????????????????????????????????????-??????---------------------??????????????????????????????????????????--???--??????????????????????????-?????????????-???-?????-??????????????????????????????--???????????????????????????????-??????????????????????????????????????????????????????????????????????????????????????--??????????????????????????????GGTAGAGGAG-GCGGTTTTTTTAGAAGCCCCGTCTAAATGCTTTCTGGTTATTGAACTCGATGGGTGTTTTCTACAAATCATAAGGACATTGGTACTTTGTATTTAATCTTCGGAGCAGGAGCTGGTTTAATTGGAACTGCTTTTAGTATGCTTATACGATTGGAGCTTTCTGCGCCGGGGGCGATGTTAGGAGATGATCATCTTTATAATGTAATTGTTACAGCACATGCTTTTATTATGATTTTTTTTTTGGTTATGCCGGTCATGATTGGGGGATTTGGTAATTGATTAGTCCCATTATATATTGGGGCGCCGGATATGGCGTTTCCTCGATTAAACAATATTAGTTTTTGACTTTTGCCTCCTGCGCTTTTTTTATTATTAGGCTCTGCTTTTATTGAA?AAGGGGCGGGGACGGGGTGAACAGTTTATCCTCCTCTTTCTAGTATTCAAGCACACTCCGGAGGTTCTGTTGATATGGTTATTTTTAGTCTTCATTTAGCTGGGGTTTCTTCTATTTTAGGTGCTATTAACTTTATTACTACAATTTTTAATATGCGAGCCCCGGGTGTGTCTTTTAATAAACTACCTTTATTTATCTGATCTATTTTAATAACAGCTTTTTTATTACTTTTATCTTTACCTGTTTTAGCTGGTGCTATTACTATGTTGTTAACAGATAGAAACTTTAATACGACTTTTTTCGATCAGATGGGTGGCGGGGACCCAATATTATTTCAGCATTTATTTTGATTCTTTGGGCATCCAGAAGTTTATATTTTAATTTTGCCTGGTTTTGGTATGATTTCTCAAATAATCCCGACTTTTGTTGCTAAAAAACAAGTTTTTGGGTATTTAGGAATGGTTTATGCCATGCTTTCTATTGGGCTTCTTGGATTTATTGTTTGAGCTCATC????????????????????????????????????????????????????????????????????????????????????????????????????????????????????????????????????????????????????????????????????????????????????????????????????????????????????????????????????????????????????????????????????????????????????????????????????????????????????????????????????????????????????????????????????????????????????????????????????????????????????????????????????????????????????????AGACGATATTCGGATTATGCAGATGCTTTTTTGGGTTGAAATTTAATAAGTTCTTTAGGGTCTATTATTTCTATTTTGAGTGTTGTTTGGTTTTTATATATTGTTTTTGATCTTTTTGTTACAGAAGAAAAATTTTTGGGTTGAAAAGAAGGATTTTCTTTAGAATGAATTCATTCTTCTCCCCCCTTATTTCATACTTATGAGGAGTTGCCCTTTGTACAAAAAGTAAATAATTTTTAGAATAGTGCCGGGTTTATATACCGGTTT

Pacu02 ?????????????????????????????????????????????????????????????????????????????????????-?????????????????????????????????????????????????????????????????????TATTGGTTGTTTCTTCAGAAAAGTGA--TTTTTTTTTATTTGAATTGAAACATCTTATTACTAAGTAAAAATCAAACGAGATTCCGAGAGTAGTGGAGAGTGAAATTGGAGTTGTGCTTGTGGTGGTCATAGATC?????????????????????????????????????????????????????????GAGTTGAAAGGAATTTGAATCTTTTATTTTTGAAGTAGCT???????????????????????????????????????????????????????????????????????????-???????????????????????????????????????????????????????????????????????????AAAAATCTCAGAAAATTTGTGATTAGGGGTGAAAGGCTAATCGAACTTGGAAATAG??????????????????????????????????-??????????????????????????????????????????????????????????????????????????????????????????????????????????????????????-??????????????????????????????????????????????????????????????????????????????????????????????????????????????????????????????????????????????????????????????????????????????????????????????????-?------------------????????????????????????????????????????????????????????????????????????????-???????????????????????????????????????????????????????????????????????????????????????????????????????????????????????????????????????????????????????????????????????????????????????????????????????????????????????????????????????????????????????????????????????????????????????????????????????????????????????????????????????????????????????????????????????????????????????????????????????????????????????????????????????????????????????????????????????????????????????????????????????????????????????????????????????TGGGGCGACAGTTTTTTAAAAAGTAACGAAAATGAGCTATGACGCATGTTTAACTTTGAAATTTTTTT-ATTGATGAGACATTTTTGGTGTGTTTTTTGATCCGTTATTTTGAATGAAAAAAATAACGAAAACAAATAAAAGTTACCCTGGGGATAACAGCGCAATAATGTTTGAGAGTTAATCAACGACAGTGTTTGCGACCTCGATGTTGAATTGTAACGTCCTACGGTGTAGTCACTCGTAAGGGTTGGTTTGTTCATCCATTAAAGTTATACATGATTTGAGTTAAAAGCGTGGTGACACAGCTTGGTTTCTATCTACAATTAGAAAACAAAAATATGTTGTTTTTTCGTACGAAAGGATC????????????????????????????????????????????????????????????????????????????????????????????????????????????????????????????????????????????????????????????????????????????????????????????????????????????????????????????????????????ATTGAAGTTTTATTTAATTCAACAACAACGTATTTGAAATTATGAAAATGATTTGATTCGGGGTTGTTTGTTGTTTTTTTTGGTTTTCAATTTGATGGTTTAGTAGTTATTATGTTATTTGTTGTTTTTATTGTTTCTACTTTGGTTCATATTTTTTCTATTGCTTATATGCGGGGAGATCCTCATATTCCTCGATTTATGACATATCTTTCTTTGTTTACTTTTTTAATGGTTTTATTGGTAACTAGCGATAATTTTCTTCAATTATTTATTGGGTG??????GGTTGGTCTTTGTTCTTATTTATTAATTAATTTTTGATTAACTCGATTAGAGGCAAATAGAGCTGCTATTAAAGCAATGTTAGTGAATAGAATTGGTGATATTGGGTTGCTTTTAGCAATGTTTTTACTTTGGGATC??????????????????????????????????????????????????????????????????????????????????????????????????????????????????????????????????????????????????????????????????????????????????????????????????????????????????????????????????????????????????????????????????????????????????????????????????????????????????????????????????????????????????????????????????????????????????????????????????????????????????????????????????????????????????????????????????????????????????????????????????????????????????????????????????????????????????????????????????????????????????????????????????????????????????????????????????????????????????????????????????????????????????????????????????????????????????????????????????????????????????????????????????????????????????????????????CTTTAAGTGTTACTGAA?????????????????????????????????????????????????CTGTTACAATAATGTTTTTTGCTTCGATTTTAGCAGAGACCAATCGTGCTCCTTTTGATTTAACAGAAGGAGAGTCAGAGCTTGTTTCGGGATATAATGTTGAGTATGCTTCGATGTCTTTTGCTTTATTTTTTCTTGCTGAATATGCCCATATTATTTTAATGAGTTGTTTAACAACGATTTTTTTTGGGGGAGGATGGCTTTCTCCGATTCCAGATTTTAAGGGTGGGGCGGGCTGGTTTGGTTTTAAAGTTGTTTTTATTATTTTTTTTTTTATTTGAGTAAGGGCTTCATTTCCTCGAATTCGATATGATCAGCTTATGGCTTTGTTATGAAAAGGGTATTTGCCTTTAAGTTTAGGAATGGTCATCTTTGTGGCTA???????????????????????????????????????????????????????????????????????????????????????????????????????????????????????????????????????????????????????????????????CATTACTTATTTATGAAATTTTGGATCTTTATTGGGATTGTGTTTGATTTTACAGATAGTGACTGGGTGTTTTTTGTCTATGCACTATTGTTCTGATGTTAATTTTGCTTTTGCTTCAATTGGTCATATTATGCGAGATGTTAATTATGGGTTTTTATTAAGATATCTTCATGCTAACGGTGCTTCTT???TTTTTTTTTGTCTTTATGTTCATAT??????????????????????????????????????????????????????????????????????????????????????????????????????????????GAGGACAAATGTCTTTTTGAGGGGCAACTGTAATAACGAATTTATTATC???????????????????ATTGATATTGTTCAGTGAGTGTGAGGCGGGTTTAGTGTTTCTAATGCAACATTAAATCGGTTTTTTAGTTTACATTTTTTACTTCCTTTTATTTTAGTTTTTCTTGTTATTCTTCATTTAGTTTATTTACATGTTGATGGGTC?????????????????????????????????????????????????????TTTATACATCAAAAGATTTTTTTGGTTTTTTTTTTCTTTTTTTTTTATTTTGTTTTTTTGTTTTTTTTTTGCCTAATTTATTAGGAGATGCTGAAAATTTTATTCAAGCGAATTCTTTGGTTACTCCGGTTCATATTCAACCAGAATGATATTTTTTATTTGCTTATGC???????????????????????????????????????????ATGTTTTGTAGTATTTTTATTTTATTTTTTTTATCTATTTTACATCAAAGTCTTTTAAAGGGACTTTTTTTTCGTCCTTTAGGTCGAATTGCCTTTTGGTTTTTAATTATTGATTTTGCTTTATTAACTTGAATTGGATC????????????????????????????????????????????????????????????????????????????????????????????????????????????????????????????????????????????????????????????????????????????????????????????????????????????????????????????????????????????????????????????GAAAGCTTTTGGTTTTAATTGGTTCTTTTGCTGTCTTTCTTTTATTTGGACCAAAAAAAGAAGAACAAACAGATGTCCCTATTTTAAGTTTAATTATTATTTTTGGAGTTTTTTGTTTAATTTCTTCAAGTAATTGACTTTCTATTTATTTAACCATTG?????????????????????????????????????????????????????????????????????????????????????????????????????????????????????????????????????????GGGG?????????????????????????????CTTTTTAATTCAAAACAAATTTTTTCTGCTGTTTCGATACCAATTGGGTATCTTTTAATTATTGTTGCTCTTTTTTTTAAATTATCAGTTGCTCCTTTTC???????????????????????????????????????????????????????????????????????????????????????????????????????????????????????????????????????????????????????????????????????????????????????????????????????????????????????????????????????????????????????????????????????????????????????????????????????????????????????????????????????????????????ATGTTTATAAAAATTTACTTGTTGAATTTAGTGGACTTTCTCGATACTTACCTTTTTTTTCGATTACTTTAGGTGTTCTTTTTTTTTCT???????????????????????????????????????????????????????????????????????????????????????????????????????????????????????????????????????????????????????????????????????????????????????????????????????????????????????????????????????????????????????????????????????????????????????????????TTTTTTTTTTTTT????????????????????????????????????????????????????????????GATAATTGGCTCAGG???????????????ASYKTTAAATCCTGTTCTTTCTATTTTTTGATTGGTTCTTGTTTTTATAAATTCTGCCGTTTTTTTCCTTTTACTAGGAATAGATTTTCTTGCTTTGATGTTTTTACTTATTTATGTTGGGGCAATAGCTATTTTATTTTTATTTGTTATTAT??????????????????????????????????????????????????????????????????????????????????????????????????????????????????????????????????????????????????????????CTTTCTTTTCCTTGATTTCTTATTTCTTATCATAATATTGAGGCCTTAGGGCAGATTTTATATGTTTCTTGTTTTTGTTTATTTCTTTTGGCCAGTTTTATTTTATTAGTTGCTATGATGGGTGTGATTGTATTAACTCAAGAAACAGAATCTTTAAGTAAAAAACAAGATC????????????????????????????????????????????????????????????????????????????????????????????????????????????????????????????????????????????????????????????????????????????????????????????????????????????????????????????????????????????????????????????????????????????????????????????????????????????????????????????????????????????????????????????????????????????????????????????????????????????????????????????????????????????????????????????????????????????????????????????????????????????????????????????????????????????????????????????????????????????????????????????????????????????????????????????????????????????????????????????????????????????????????????????????????????????????????????????-----------------?????????????????????????????????????????????????????GTGAATTATGGATTTGGTATTGGGTGATATGGTAGTTCTTCTCAAG???????????????????????---????????????????????????????GAGC????????????????????????????????????????????????ACAAGTGAATCTAGATTCAACTGAAGGACCAAATGATGTTGGGGTACAAGTGAATCAAGAGTCTGAACAGTCTGTGGATGTTGTTTTTATAGAATTGGAAGGGCGAAGGGTCCCCGTAAG--GGGGGTGA------------------ATCTACATGTTGAGCGGCGT-GGTTATTTGGATAATCG---GTCTAATCAAGAAGG--TGT-TGA---------------------------------------------GAATAGTTTAGGAGATCA-----ATTTGA-AGATGTGGCTTT---TTTAG-GGGGGAG-TACAAGTACACTTACACCAGAAA----GTGTAAATAGGGGCGCTCCAAGTGGTAATATAAGAAATGTATTAAGTGATTCATTAA-TAGA-G-AG-GAGAGGCCAAGACCAAACTTTAAGTGA--AA-TAAAGGTAGAAACACCTCC----AGGTGCTTCAAGGGGTGGCCGATG-A-------G--CCAGT-AT-----AAATCAAGGTGAGGCTTTAAG---AGAAAACAAGGTAGAAATTGGGG----TTTTAAGAGAAAACAAAGT--------AGAAAT--TGATGAGGTAGTTGGCTCGACTTTTAGTTCGATCCCCTTAGAGGTTGAAGGGATGATGGCTCATGTGTGAAATCAATTTGACATGGTGTTTAACATATTGGCTGATAAGCCCCTGGTAGGTTGCATTTTTTTTTCTTACCAGCTTCTAAGAATAACGAATCAAATGCCAAACCCAC????????????????????????????????????????????????????????????????????????????????????????????????????????????????????????????????????????????????????????????????????????????????????????????????????????????????????????????????????????????????????????????????????????????????????????????????????????????????????????????????????????????????????????????????????????????????????????????????????????????????????????????????????????????????????????????????????????????????????????????????????????????????????????????????????????????????????????????????????????????????????????????????????????????????????????????????????????????????????????????????????????????????????????????????????????????????????????????????????????????????????????????????????????????????????????????????????????????????????????????????????????????????????????????TTTAATTAGCCAAAAATCAATAAAAT???????????????????????????????????????????????????????????????????????????????????????????????????????????????????????????????????????????????????????????????????????????????????????????????????????????????????????????????????????????????????????????????????????????????????ATTAAATTATTTTTAAATATTCAAAAATGAGCCTTAGTCGGGGTTTTTCTTAGTTTTGCGGTGAAACTACCTCTTATTCCATTTCATATTTGATTGCCACAAGCACATGTTGAGGCTCCTGTTGCGGGCTCTGTTATTTTGGCTGGAATTTTATTAAAATTAGGGGGTTATGGCCTTTTGCGTTTTTCTTGACCTCTTTTTCCGGGGGCTTCTTTATATTGGTCTCCAGTTATTGTTTTTTTTAGTGTTGTTGCTGTTGTTTATGGAGGTTTAATGACATGTCGTCAAATTGATTTTAAACGGCTTGTTGCTTACTCTTCTGTTGCTCATATGGGACTTGTGCCTTTGGGTCTTTTTACACATGTTATAGAGGGGTTAATTGGGGCTCTTTTTTTAATGTTGGCGCACGGATTTGTTAGCTCTGCTCTTTTTATTGGAGTAACTTTTTTGTATGATCGCCATCATACTCGTTTAATAAAATATTATCGGGGTTTGACTTTGACAATGCCTCTTTTTGTTATTACAATGTTAATTTTGTCTTTGGCGAACATGGGTTTTCCTCTTAGTTGTAATTTTGTTGGAGAGTTTTTTTCTTTATTAGCAGTCTTTCAATATCATTATGGAGTTGGAATGTTTGTTATTTTAGGGGTTCTTTTTTCTGCCATTTATTCTCTTAGTCTTTTTAATCGTATTTCTTTTGGTGGAGGCTCTAATTATTT???????????????????????????????????????????????????????????????????????????????????????????????????????????????????????????????????????????-------------??????????????????????????????????????????????????????????????????????????-??????????????????????????????????????????????????-?????????????????????????????????????????????????????????????????????????????????????????????????????????????????????????????????-????????????????????????????????????-???????????????????????????????????????????????????????????????????--??????????????????????????????????????????????????????------????????????????????????????????????????????????????????????????????????????????????????????????????????????????????????????????-??????TATTAG?????????????????????ATTATCCGCGAGAGACCTTACCGAAGTTTGAATTTATTAACAGGTGTTGCATGGCCGTCGTCAATTTCTGTTTGAGACAAAAAGGGGTTTAATCCTATCAA???????????????????????????????????????????????????????????????????????????????????????????????????????????????????????????????????????????????????????????????TTA?????AATAGTCCGTCGCCAGCTTAKAAATAGGCTGTAAGTCGTAACATAGTAAGAGTGAGGGAACTGGCTCTTGATGCAGTTTCATTCG??????????????????????????????????????????????????????????????????????????????????????????????????????????????????????????????????????????????????????????????????????????????????????????????????????????????????????????????????????????????????????????????????????????????????????????????????????????????????????????????????????????????????????????????????????????????????????????????????????????????TGGGGCAACGGTTACATGGGCGCATCATGCAATACTTTGTGGTTTAAAAAAAGAGGCTCAATTTGCTTTATTTTTAACTCTTTTTTTGGGTGTTA?????????????????????????????????????????????????????????????TTATGGGGCCACTTTTTTTGTTGCGACAGGGTTTCATGGACTACATGTTATAATTGGTACGACTTTTCTTTTTATTTGTTTTTTACGTTTACTCTCTAATC????????????????????????????????????????????????????????????????????????????????????????????????????????????????????????????????????????????????????????????????????????????????????????????????????AATCATCTTTATTATTCGGATAGTCCAGAGACTTGACTTTTAGAGTTTCAAGATGTTGGGGATCCTATTGTAGAAGAAATTGTTTTTTTTCATGACCAAGTTATGTTTTTATTAATTATTATAGTTACTGTTGTTTTATGACTTTTTGTTGAAGCTTTTAAAAATAAGTTTTATGATCGTCATTTAATCGATGGTACATTTTTAGAAATTGTTTGAACGATTATTCCTGCTGTTATATTGATTTTTATTGCATTGCCCTCTCTTAAATTATTATATTTAATGGATGAGGTTATTTCTCCAGCTTTGACAATTAAAGTTATTGGGCATCAATGATATTGATCCTATGAATATTCTGATTATGAAGGGGATACGTTAGGGTTTGATTCTTATATGATTCCGACTTCGGATTTAGTTTCTGGAGAGAATCGTTTGTTAGAGGTTGATTATAAACTTTTAATCCCTATTCAAACACATATAAGATTTTTAGTTACTGG????????????????????????????????????????????????????????????????????????????????????????????????????????????????????????????????????????????????????????????????????????????????????????????????????????????????????????????????????????????????????????????????????????????????????????????????????????????????????????????????????????????????TTTTTTTTTTTTTTAA?????????????????GATCTTTTAATAGAACAAATTTTTATGATTATGGGGTTAACTATTGCTGCTGCCGAATCTTCAATTGGTTTAGCTATTTTAGTTGCATATTATAGAATTCGAGG??????????????AATCTTTT??????????GAGGATAATGATTAATCTTTTTATTTTTTTTTTTCTTATTTTTATTTTGGCGGGGTTATTTATATTTCTTTCTTTTTTGATAGGGGAAAAAATTCCAGATCGAGAAAAGGTTTCTGCTTATGAGTGTGGTTTTGCTCCCTTTAATTTTTTAGGACGCCCTTTTTCAATACGTTTTTTTTTAATTGGTATTTTATTCTTGATTTTTGATTTAGAAATTTCTTTTTTTTTTCCCTGATGTGTTTTATATAATTCGACTGCTCCGTTTGGGTTTTGAACTA??????????????????????????????????????TATGAATGAGTAATGGGGGGATTAGAATGAGAGTAAAAATAAAGTAAAAGAAAAAGTCCTATCTATTATGAAAATAATAGAAATTTTATACCAACTCCGGT?????????????????????????????????????????????????????????????????????????????????????????????????????????????????????????????????????????????????????????????????ATTTAAAAAAAATAATCGCTTATTCTACTTGTAGTCAATTAGGTTATATGGTTGTTGCTTGTGGGCTTTC????????????????????????????????????????????????????????????????????????????????????????????????????????????????????????????????????????????????????????????????????????????????????????????????????????????????????????????????????????????????????????????????????????????????????????????????????????????????????????????????????????????????ATCCAATATTAATTTAAAACGAGCAAACATTTTTTTTCTTAAAGAAGGAGAATTTTTATTTTTAATTCCTTTGGGCATATTA?????????????????????????????????????????????????????????????????????????????????????????????????????????????????????????????????????????????????????????????????????????????????????????????????????????????????????????????TTTTGGTTCTGCTTGACAAATAAATTTTTTTTTTAATTTTTTCTTTATAAAAAAAATATATAAAATAG???????????????????????????????????????????????????????????????????????????????????????????????????????????????????????????????????????????????????????????????????AATTTTTGCACTTTAATTAAAATTTTTGAAGGGGTTAAGTTAAGCAAAACAG??????????????????????????????????????????????????????????????????????????????????????????????????????????????????????????????????????????????????????????????????????????????????????????????---------------?????????????????????????????????????????????????????????????????????????????????-????????--??????????????????????????-???????????????????????????????????????-????????????????-------------------------------------??????????------------?????????????----------------------------------------------??????????????????????---??????GATC-TTT---GGGGATGGTGTGGA--AAAAATGGAAGTAGATG-------ATGA--AGAGATAGTAGGAG--CGTTTCCATCTTTA--------AGCTTCGTGGAGGGGTGAGATG----------------------------AAGAGGTGATAGCAGCGTTTCCATGTTCAGACT----------CCGAAGAGAGTATGGAAATTGATGGGGAAGA--ACTACCTTTGGATCCCCTCCCGATTAGTGGAGCT--ATTAGAGGAGAA--AACATAGTGGCA-------------------------------CCCGTACCAGTCATGGGAGATGCGGGTCTTTCAGTTCATTTTTGGCGTGCACTCAAGAACACTCCATTTGAATTAT-TGGCAGAAGTTTATCGGTCAGGTGAGGAGGGAGAAG--TT---T--ATTCGTCAGATGAG---GAAGACGAGTTAGTAGAGGTGTTACTTCAGTACGGAAA-AGAGAT---------------------AACATCAAAAGAGTCAGAGGACAAATTTTTGAGTGGGCTAAT--AGT--GGAAATGGTGGAAAACGTAATTGCCT-AAGATAAATACCA-GAA-CAGTT-AGTAAACAGATTATTGAGTGGGTCAGTGGA--CAAAGTAGGGGATAACGTGGTTGGGTAAAAT-ATAGTGTCGGCGGCAACGGAAGTGGGGAGGGGGAG???????????????????????????????????????????????????--????????????????????????????????????????-????????????????????GTCTAAATGCTTTCTGGTTATTGAACTCGATGGGTGTTTTCTACAAATCATAAGGACATTGGTACTTTGTATTTAATCTTCGGAGCAGGAGCTGGTTTAATTGGAACTGCTTTTAGTATGCTTATACGATTGGAGCTTTCTGCGCCGGGGGCGATGTTAGGAGATGATCATCTTTATAATGTAATTGTTACAGCACATGCTTTTATTATGATTTTTTTTTTGGTTATGCCGGTCATGATTGGGGGATTTGGTAATTGATTAGTCCCATTATATATTGGGGCGCCGGATATGGCGTTTCCTCGATTAAACAATATTAGTTTTTGACTTTTGCCTCCTGCGCTTTTTTTATTATTAGGCTCTGCTTTTATTGAACAAGGGGCGGGGACGGGGTGAACAGTTTATCCTCCTCTTTCTAGTATTCAAGCACACTCCGGAGGTTCTGTTGATATGGTTATTTTTAGTCTTCATTTAGCTGGGGTTTCTTCTATTTTAGGTGCTATTAACTTTATTACTACAATTTTTAATATGCGAGCCCCGGGTGTGTCTTTTAATAAACTACCTTTATTTGTTTGATCTATTTTAATAACAGCTTTTTTATTACTTTTATCTTTACCTGTTTTAGCTGGTGCTATTACTATGTTGTTAACAGATAGAAACTTTAATACGACTTTTTTCGATCCAGCGGGTGGCGGGGACCCAATATTATTTCAGCATTTATTTTGATTCTTTGGGCATCCAGAAGTTTATATTTTAATTTTGCCTGGTTTTGGTATGATTTCTCAAATAATCCCGACTTTTGTTGCTAAAAAACAAGTTTTTGGGTATTTAGGAATGGTTTATGCCATGCTTTCTATTGGGCTTCTTGGATTTATTGTTTGAGCTCATCATATGTTTACTGTTGGGATGGATGTAGATACAAGAGCATATTTTACTGCTGCTACTATGATTATTGCTGTGCCAACTGGGATTAAAGTTTTTAGTTGGTTGGCAACTATTTATGGAGGTGTTCTTAGGTTAGAGACTCCAAT?CTTTGAGCTATGGGGTTTGTTTTTTTATTTACAGTTGGTGGTTTAACTGGGGTTGTGTTAGCAAATAGTTCTCTTGATATTGTTCTACATGATACATATTATGTAGTTGAGCATTTTCATTATGTTCTTTCTATGG??????????????????????????????????????????????????????????????????????????????????AGTTCATTTTTGATTAATGTTTATCGGGGTTAATCTAACTTTTTTCCCTCAACATTTTTTAGGTTTAGCAGGATTTCCAAGACGATATTCGGATTATGCAGATGCTTTTTTGGGTTGAAATTTAATAAGTTCTTTAGGGTCTATTATTTCTATTTTGAGTGTTGTTTGGTTTTTATATATTGTTTTTGATCTTTTTGTTACAGAAGAAAAATTTTTGGGTTGAAAAGAAGGATTTTCTTTAGAATGAATTCATTCTTCTCCCCCCTTATTTCATACTTATGAGGAGTT??????????????????????????????????????????????????????????

Pacu01 ?????????????????????????????????????????????????????????????????????????????????????-???????????????????????????????????????????????????????????????????????????????????????AAAAGTGA--TTTTTTTTTATTTGAATTGAAACATCTTATTACTAAGTAAAAATCAAACGAGATTCCGAGAGTAGTGGAGAGTGAAATTGGAGTTGTGCTTGTGGTGGTCATAGATC????????????????????????????????????????????????????????????????????????????????????????????????????????????????????????????????????????????????????????????????????????????-?????????????????????????????????????????????????????????????????????????????????????????????????????????????????????????????????????????????????????????????????????-??????????????????????????????????????????????????????????????????????????????????????????????????????????????????????-??????????????????????????????????????????????????????????????????????????????????????????????????????????????????????????????????????????????????????????????????????????????????????????????????-?------------------????????????????????????????????????????????????????????????????????????????-???????????????????????????????????????????????????????????????????????????????????????????????????????????????????????????????????????????????????????????????????????????????????????????????????????????????????????????????????????????????????????????????????????????????????????????????????????????????????????????????????????????????????????????????????????????????????????????????????????????????????????????????????????????????????????????????????????????????????????????????????????????????????????????????????????TGGGGCGACAGTTTTTTAAAAAGTAACGAAAATGAGCTATGACGCATGTTTAACTTTGAAATTTTTTT-ATTGATGAGACATTTTTGG??????????GATCCGTTATTTTGAATGAAAAAAATAACGAAAACAAATAAAAGTTACCCTGGGGATAACAGCGCAATAATGTTTGAGAGTTAATCAACGACAGTGTTTGCGACCTCGATGTTGAATTGTAACGTCCTACGGTGTA???????GTAAGGGTTGGTTTGTTCATCCATTAAAGTTATACATGATTTGAGTTAAAAGCGTGGTGACACAGCTTGGTTTCTATCTACAATTAGAAAACAAAAATATGTTGTTTTTTCGTACGAAAGGATC????????????????????????????????????????????????????????????????????????????????????????????????????????????????????????????????????????????????????????????????????????????????????????????????????????????????????????????????????????????????????????????????????????????????????????????WTGAKKCGGGGTTGTTTGTTGTTTTTTTTGGTTTTCAATTTGATGGTTTAGTAGTTATTATGTTATTTGTTGTTTTTATTGTTTCTACTTTGGTTCATATTTTTTCTATTGCTTATATGCGGGGAGATCCTCATATTCCTCGATTTATGACATATCTTTCTTTGTTTACTTTTTTAATGGTTTTATTGGTAACTAGCGATAATTTTCTTCAATTATTTATTGGGTGAGAAGGGGTTGGTCTTTGTTCTTAT?????????????????ATTAACTCGATTAGAGGCAAATAGAGCTGCTATTAAAGCAATGTTAGTGAATAGAATTGGTGATATTGGGTTGCTTTTAGCAATGTTTTTACTTTGGGATC????????????????????????????????????????????????????TCAAATATTTTTTATTTGTTTATTTTTATTTTTTGGGGTTATGGGAAAATCTGCT???????????????????????????????????????????????????????????????????????????????????????????????????????????????????????????????????????????????????????????????????????????????????????????????????????????????????????????????????????????????????????????????????????????????????????????????????????????????????????????????????????????????????????????????????????????????????????????????????????????????????????????????????????????????????????????????????????????????????????????????????????????????????????????????????????????????????????????????????????????????????????????????????????????????????????????????????????????????????????????????????????????????????????????????????????????????????????????????????????????????????????????????????????????????????????????????????????????????????????????????????????????????????????????????????????????????????????????????????????????????????????????????????????????????????????????????????????????AGATTTTAAGGGTGGGGCGGGCTGGTTTGGTTTTAAAGTTGTTTTTATTATTTTTTTTTTTATTTGAGTAAGGGCTTCATTTCCTCGAATTCGATATGATCAGCTTATGGCTTTGTTATGAAAAGGGTATTTGCCTTTAAGTTTAGGAATGGTCATCTTTGTGGCTAGTGTTCTCTTTGGGTTTAATGGTCCC????????????????????????????????????????????????????????????????????????????????????????????????????????????????????????????????????????????????????????????????GATCTTTATTGGGATTGTGTTTGATTTTACAGATAGTGACTGGGTGTTTTTTGTCTATGCACTATTGTTCTGATGTTAATTTTGCTTTTGCTTCAATTGGTCATATTATGCGAGATGTTAATTATGGGTTTTTATTAAGATATCTTCATGCTAACGG??????TTTGTTTTTTTTTTGTCTTTATGTTCATATTGGTCGAAGTTTATATTATGGGGGGTATTTAAAATTTCATGTTTGAAGCATTGGAGTTGTTATTTTTTTAT??????????????????????????????????????????????????????????????????????????????????????????????????????????????????????????????????????????????????????????????????????????????????????????????????????????????????????????????????????????????????????????????????????????????????????????????????????????????????????????????????????????????????????????????????????????????????????????????????????????????????????????????????????????????????????????????????????????????????????????????????????????????????????????????????????????????????????????????????????????TTACATCAAAGTTTTTTAAAGGGACTTTTTTTTCGTCCTTTAGGTCGAATTG???????????????????????????????????????????????????????????????????????????????????????????????????????????????????????????????????????????????????????????????????????????????????????????????????????????????????????????????????????????????????????????????????????????????????????????????????????????????????????????????????????????????????????????????????????????????????????????????????????????????????????????????????????????????????????????????????????????????????????????????????????????????????????????????????????????????????????????????????????????????????????????????????????????????????????????????????????????????????????????????????????????????????????????????????????????????????????????????????????????????????????????????????????????????????????????????????????????????????????????????????????????????????????????????????????????????????????????????????????????????????????????????????????????????????????????????????????????????????????????????????????????????????????????????????????????????????????????????????????????????????????????????????????????????????????????????????????????????????????????????????????????????????????????????????????????????????????????????????????????????????????????????????????????????????????????????????????????????????????????????????????????????????????????????????????????????????????????????????????????????????????????????????????????????????????????????????????????????????????????????????????????????????????????????????????????????????????????????????????????????????????????????????????????????????????????????????????????????????????????????????????????????????????????????????????????????????????????????????????????????????????????????????????????????????????????????????????????????????????????????????????????????????????????????????????????????????????????????????????????????????????????????????????????????????????????????????????????CTTAGGGCAGATTTTATATGTTTCTTGTTTTTGTTTATTTCTTTTGGCCAGTTTTATTTTATTAGTTGCTATGATGGGTGTGATTGTATTAACTCAAGAAACAGAATCTTTAAGTAAAAAACAAGATCTTTTTTTCCAAATAAATAGGTAATGAACAGTTCTTCTTATTTTGAACAATTTAACATAGTGTGATTGTTTGGTTTTACGAATTCAAC??????????????????????????????????????????????????????????????????????????????????????????????????????????????????????????????????????????????????????????????????????????????????????????????????????????????????????????????????????????????????????????????????????????????????????????????????????????????????????????????????????????????????????????????????????????????????????????????????????????????????????????????????????????????????????????????????TCTTTTATTTGCAATTTTGGCAGGATTTGGTCTGGTTTTTAAATTTGCAAT??TTATAATGGTGTTTATTACGCTATTAGAGGTGGCAGTTGCAATAATRC??????????????????????????????????????????????????????????????-----------------??????????????????????????????????????????????????????????????????????????????????????????????????????????????????????????---??????????????????????????????????????????????????????????????????????????????GTACAAGTGAATCTAGATTCAACTGAAGGACCAAATGATGTTGGGGTACAAGTGAATCAAGAGTCTGAACAGTCTGTGGATGTTGTTTTTATAGAATTGGAAGGGCGAAGGGTCCCCGTAAG--GGGGGTGA------------------ATCTACATGTTGAGCGGCGT-GGTTATTTGGATAATCG---GTCTAATCAAGAAGG--TGT-TGA---------------------------------------------GAATAGTTTAGGAGATCA-----ATTTGA-AGATGTGGCTTT---TTTAG-GGGGGAG-TACAAGTACACTTACACCAGAAA----GTGTAAATAGGGGCGCTCCAAGTGGTAATATAAGAAATGTATTAAGTGATTCATTAA-TAGA-G-AG-GAGAGGCCAAGACCAAACTTTAAGTGA--AA-TAAAGGTAGAAACACCTCC----AGGTGCTTCAAGGGGTGGCCGATG-A-------G--CCAGT-AT-----AAATCAAGGTGAGGCTTTAAG---AGAAAACAAGGTAGAAATTGGGG----TTTTAAGAGAAAACAAAGT--------AGAAAT--TGATGAGGTAGTTGGCTCGACTTTTAGTTCGATCCCCTTAGAGGTTGAAGGGATGATGGCTCATGTGTGAAATCAATTTGACATGGTGTTTAACATATTGGCTGATAAGCCCCTGGTAGGTTGCATTTTTTTTTCTTACCAGCTTCTAAGAATAACGAATCAAATGCCAAACCCAC?????????????????????????????????????????????????????????????????????????????????????????????????????????????????????????????????????????????????????????????????????????????????????????????????????????????????????????????????????????????????????????????????????????????????????????????????????????????????????????????????????????????????????????????????????????????????????????????????????????????????????????????????????????????????????????????????????????????????????????????????????????????????????????????????????????????????????????????????????????????????????????????????????????????????????????????????????????????????????????????????????????????????????????????????????????????????????????????????????????????????????????????????????????????????????????????????????????????????????????????????????????????????????????????????????????????????????????????????????????????????????????????????????????????????????????????GATCTTCTTTTGTTTTATCTTTTTTTTGAGGGGATATTAATACCAATGTTTTTTTTAATTGGTATTTGAGGCTCTCGAGAAGAAAAGGTTCGTGCTTCTTTTTATTTTTTTTTTTTTACTTTTATAG????????????????????????????????????????????????????????????????????????????????????????????????????????????????????????????????????????????????????????????????????????????????????????????????????????????????????????????????????????????????????????????????????????????????????????????GCTTCTTTATATTGGTCTCCAGTTATTGTTTTTTTTAGTGTTGTTGCTGTTGTTTATGGAGGTTTAATGACATGTCGTCAAATTGATTTTAAACGGCTTGTTGCTTACTCTTCTGTTGCTCATATGGGACTTGTGCCTTTGGGTCTTTTTACACATGTTATAGAGGGGTTAATTGGGGCTCTTTTTTTAATGTTGGCGCACGGATTTGTTAGCTCTGCTCTTTTTATTGGAGTAACTTTTTTGTATGATCGCCATCATACTCGTTTAATAAAATATTATCGGGGTTTGACTTTGACAATGCCTCTTTTTGTTATTACAATGTTAATTTTGTCTTTGGCGAACATGGG???????????????????????????????????????????????????????CAATATCATTATGG?????????????????????????????????????????????????????????????????????????TTTGGTGGAGGCTC????????????????????????????????????????????????????????????????????????????????????????????????????????????????????????????????????????????????????-------------??????????????????????????????????????????????????????????????????????????-??????????????????????????????????????????????????-?????????????????????????????????????????????????????????????????????????????????????????????????????????????????????????????????-????????????????????????????????????-???????????????????????????????????????????????????????????????????--??????????????????????????????????????????????????????------????????????????????????????????????????????????????????????????????????????????????????????????????????????????????????????????-???????????????????????????????????????????????????????????????????????????????????????????????????????????????????????????????????????????????????????????????????????????????????????????????????????????????????????????????????????????????????????????????????????????????????????????????????????????????????????????????????????????????????????????????????????????????????????????????????????????????????????????????????????????????????????????????????????????????????????????????????????????????????????????????????????????????????????????????????????????????????????????????????????????????????????????????????????????????????????????????????????????????????????????????????????????????????????????????????????????????????????????????????????????????????????????????????????????????????????????????????????????????????????????????????????????????????????????????????????????????????????????????????????????????????????????????????????????????????????????????????????????????????????????????????????????????????????????????????????????CTAATCAATTTACTCGTCGTCAACATGTTGGGTTTGAAGCCGCGAGTTGATACTGGCACTTTGTCGATGTGGTTTGATTATTTTTATATTTATGT???????????????????????????????????????????????????????????????????????????????????????????????????????????????????????????????????????????????????????????????????????GATCCTATTGTAGAAGAAATTGTTTTTTTTCATGACCAAGTTATGTTTTTATTAATTATTATAGTTACTGTTGTTTTATGACTTTTTGTTGAAGCTTTTAAAAATAAGTTTTATGATCGTCATTTAATCGATGGTACATTTTTAGAAATTGTTTGAACGATTATTCCTGCTGTTATATTGATTTTTATTGCATTGCCCTCTCTTAAATTATTATATTTAATGGATGAGGTTATTTCTCCAGCTTTGACAATTAAAGTTATTGGGCATCAATGATATTGATCCTATGAATATTCTGATTATGAAGGGGATACGTTAGGGTTTGATTCTTATATGATTCCGACTTCGGATTTAGTTTCTGGAGAGAATCGTTTGTTAGAGGTTGATTATAAACTTTTAATCCCTATTCAAACACATATAAGATTTTTAGTTACT??????????????????????????????????????????????????????????????????????????????????????????????????????????????????????????????????????????????????????????????????????????????????????????????????????????????????????????????????????????????????????????????????????????????GTATAATTTTAAATAGAGGACATTTTATTATTATGCTTGTTTCTATTGAATTAGTTTTATTATCAACTTTTTTTTTTTTTTTAATAAGTTCTAAAGAAATAGATCTTTTAATAGAACAAATTTTTATGATTATGGGGTTAACTATTGCTGCTGCCGAATCTTCAATTGGTTTAGCTATTTTAGTTGCATATTATAGAATTCG???????????????????????????????????GAGGATAATGATTAATCTTTTTATTTTTTTTTTTCTTATTTTTATTTTGGCGGGGTTATTTATATTTCTTTCTTTTTTGATAGGGGAAAAAATTCCAGATCGAGAAAAGGTTTCTGCTTATGAGTGTGGTTTTGCTCCCTTTAATTTTTTAGGACGCCCTTTTTCAATACGTTTTTTTTTAATTGGTATTTTATTCTTGATTTTTGATTTAGAAATTTCTTTTTTTTTTCCCTGATGTGTTTTATATAATTCGACTGCTCCGTTTGGGTTTTGAACTATG????????????????????????TTGGGTTTGATATATGAATGAGTAAT???????????????????????????????????????????????????????????????????????????????????????????GCTTTAATTCATGCTGCAACGATGGTTACTGCAGGTGTTTTTTTATTAATTCGTGCTTCTCCTTTATTTGATGTTGTTCC??????????AT???????????????????????????????????????????????????????????????????????????????????????????????????????????????????????????????????????????????????????????????????????????????????????????????????????????????????????????????????????????????????????????????????????????????????????????????????????????????????????????????????????????????????????????????????????????????????????????????????????????????????????????????????????????????????????????????????????????????????????????????????????????????????????????????????????????????????????????????????????????????????????????????????????????????????????????????????????????????????????????????????????????????????????????????????????????????????????????????????????????????????????????????????????????????????????????????????????????????????????????????????????????????????????????????????????????????????????????????????????????????????????????????????????????????????????????????????????????????????????????????????????????????????????????????????????????????????????????????????????????????????????????????????????????????????????????????????????????????????????????????????????????????????????????????????????????????????????????????????????????????????????????????????????????????????????????????---------------?????????????????????????????????????????????????????????????????????????????????-????????--??????????????????????????-???????????????????????????????????ATGA-TTTTTTTTTTTTTTAT-------------------------------------AAAATTTTGT------------TCTAAGAAGATTC----------------------------------------------GGGCTCGTTTAGCGATCTTTTC---ACCGTTGATC-TTT---GGGGATGGTGTGGA--AAAAATGGAAGTAGATG-------ATGA--AGAGATAGTAGGAG--CGTTTCCATCTTTA--------AGCTTCGTGGAGGGGTGAGATG----------------------------AAGAGGTGATAGCAGCGTTTCCATGTTCAGACT----------CCGAAGAGAGTATGGAAATTGATGGGGAAGA--ACTACCTTTGGATCCCCTCCCGATTAGTGGAGCT--ATTAGAGGAGAA--AACATAGTGGCA-------------------------------CCCGTACCAGTCATGGGAGATGCGGGTCTTTCAGTTCATTTTTGGCGTGCACT????????CTCCATTTGAATTAT-TGGCAGAAGTTTATCGGTCAGGTGAGGAGGGAGAAG--TT---T--ATTCGTCAGATGAG---GAAGACGAGTTAGTAGAGGTGTTACTTCAGTAC?????-??????---------------------??????????????????????????????????????????--???--??????????????????????????-?????????????-???-?????-??????????????????????????????--???????????????????????????????-??????????????????????????????????????????????????????????????????????????????????????--????????????????????????????????????????-???????????????????????????????????????????????CGATGGGTGTTTTCTACAAATCATAAGGACATTGGTACTTTGTATTTAATCTTCGGAGCAGGAGCTGGTTTAATTGGAACTGCTTTTAGTATGCTTATACGATTGGAGCTTTCTGCGCCGGGGGCGATGTTAGGAGATGATCATCTTTATAATGTAATTGTTACAGCACATGCTTTTATTATGATTTTTTTTTTGGTTATGCCGGTCATGATTGGGGGATTTGGTAATTGATTAGTCCCATTATATATTGGGGCGCCGGATATGGCGTTTCCTCGATTAAACAATATTAGTTTTTGACTTTTGCCTCCTGCGCTTTTTTTATTATTAGGCTCTGCTTTTATTGAACAAG?????????CGGGGTGAACAGTTTATCCTCCTCTTTCTAGTATTCAAGCACACTCCGGAGGTTCTGTTGATATGGTTATTTTTAGTCTTCATTTAGCTGGGGTTTCTTCTATTTTAGGTGCTATTAACTTTATTACTACAATTTTTAATATGCGAGCCCCGGGTGTGTCTTTTAATAAACTACCTTTATTTGTTTGATCTATTTTAATAACAGCTTTTTTATTACTTTTATCTTTACCTGTTTTAGCTGGTGCTATTACTATGTTGTTAACAGATAGAAACTTTAATACGACTTTTTTCGATC????????????????????????????????????????????????????????????????????????TAATTTTGCCTGGTTTTGGTATGAT???????????????????????????????????????????????????????????????????????????????????????????????????????????????????????????????ATGGATGTAGATACAAGAGCATATTTTACTGCTGCTACTATGATTATTGCTGTGCCAACTGGGATTAAAGTTTTTAGTTGGTTGGCAACTATTTATGGAGG??????????????????????????????????????????????????????????????????????????GGGTTGTGTTAGCAAATAGTTCTCTTGATATTGTTCTACATGATACATATTATGTAGTTGCGCATTTTCATTATGTTCTTTCTATGGGGGCTGTTTTTGCT?????????????????????????????????ATAA??????????????AATGAATTTTTTGGGAAAGTTCATTTTTGATTAATGTTTATCGGGGTTAATCTAACTTTTTTCCCTCAACATTTTTTAGGTTTAGCAGGATTTCCAAGACG??????GGATTATGCAGATGCTTTTTTGGGTTGAAATTTAATAAGTTCTTTAGGGTCTATTATTTCTATTTTGAGTGTTGTTTGGTTTTTATATATTGTTTTTGATC???????????????????????????????????????????????????????????????????????????????????????????????????????????????????????????????????????????????????????????

SD1 NNNNNNNNNTACAAAGGGTTAGTCATTGGGTTCATGCCCCAACCAAGTGAGTTCGAATCTCTCTCTTACATAAAATAAAAATTAT---AAAAAAAAGAGGATAACTAAGATAAACTAAAATTAGACGATTTTTTTCGAAATGGCGTTCAGTTGCTTATTGGTTGTTTCTTCAGAAAAGTGA--TTTTTTTTTATTTGAATTGAAACATCTTATTACTAAGTAAAAATCAAACGAGATTCCGAGAGTAGTGGAGAGTGAAATTGGAGTTGTGCTTGTGGTGGTCATAGATCTTGCTTAAACATTAGTTTATACTGATAATGAAAGTACTGTAAAGGAAAGTTGAAAGAGAGTTGAAAGGAATTTGAATCTTTTMTTTTTGAAGTAGCTTTAAAAAAGCGTACCTTTTGTATAATGGGTAAAAGAGATTTATTTGGCATATTTAAAATGGAAAATTTTAAGTAA-TTTTTTTTTAGTCAAATTTCCCGAAACCA????????????????????????????????????????????????????????????????????????????????????????????????????????????????????????????????????????-??????????????????????????????????????????????????????????????????????????????????????????????????????????????????????-??????????????????????????????????????????????????????????????????????????????????????????????????????????????????????????????????????????????????????????????????????????????????????????????????-?------------------??????????????????????????????????????????TGTAATAATTTTTACATAAGTAATCTAAATATTA-TTTTTTTTTCTTAAGATTACCTTAGTCAAGGGKAATACAGTTTCTAAAAAAGAAATTTTTTATGAAG???????????????????????????????????AAAAAAAAGAACTGTTTTTCTAATTAAAGAAAGAAAAAAAAAAAGAAAAACATTTTTTTAAGGAACTCGGCAAAGTTAAACTTCGACTGTTTACCAAAAACATAGCTTTTTG????????????????????????????????????TCTTAATATGTTTGTTCCGC???????????????????????????????????????????????????????????????????????????????????????????????????????????????????????????????????????????????????????????????????????????????????????????????????????GTAAGCTTATATATATAATTTTTTTTTTTTTATAAGTAAGACAGTTTTGTTGGGGCGACAGTTTTTTAAAAAGTAACGAAAATGAGCTATGACGCATGTTTAACTTTGAAATTTTTTT-ATTGATGAGACATTTTTGGTGTGTTTTTTGATCCGTTATTTTGAATGAAAAAAATAACGAAAACAAATAAAAGTTACCCTGGGGATAACAGCGCAATAATGTTTGAGAGTTAATCAACGACAGTGTTTGCGACCTCGATGTTGAATTGTAACGTCCTACGGTGTAGTCACTCGTAAGGGTTGGTTTGTTCATCCATTAAAGTTATACATGATTTGAGTTAAAAGCGTGGTGACACAGCTTGGTTTCTATCTACAATTAGAAAACAAAAATATGTTGTTTTTTCGTACGAAAGGATCAAAAATCAATAGTTCTCTTAATATAACTATTCTTTAAATTAGGATTGTTTCTAAAAATAAAAAGAAATATTTTTGTTTGTTGTTTTTATTAATGTATCTTTTAGTTTTATTTTTTCCTTTAATTGGAGCTGTTTTAACAGGTTGTTTTGGAAGAAAAATCGGAGAAAGAGGAGCGGGGATTTTAACTTCAAGTTGTTTAGTTTTTAGTTTATCTTATTCTTTTTTAATCGCGAT????????????????????????????TATTTGAAATTATGAAAATGATTTGATTCGGGGTTGTTTGTTGTTTTTTTTGGTTTTCAATTTGATGGTTTAGTAGTTATTATGTTATTTGTTGTTTTTATTGTTTCTACTTTGGTTCATATTTTTTCTATTGCTTATATGCGGGGAGATCCTCATATTCCTCGATTTATGACATATCTTTCTTTGTTTACTTTTTTAATGGTTTTATTGGTAACTAGCGATAATTTTCTTCAATTATTTATTGGGTGAGAAGGGGTTGGTCTTTGTTCTTATTTATTAATTAATTTTTGATTAACTCGATTAGAGGCAAATAGAGCTGCTATTAAAGCAATGTTAGTGAATAGAATTGGTGATATTGGGTTGCTTTTAGCAATGTTTTTACTTTGGGATCTTTTTGGATCTCTAGATTTTTCTACTATTTTTAATTCTATTTTTTTTTCTAA??????????????????????????????????????????GGGAAAATCTGCTCAATTAGGATTACATACTTGGTTACCGGATGCAATGGAAGGTYATTGGGCCTTTTAATTAAAAAATTAATTAAAAAWATTACTATAC?????????????????????????????????????????????????????????????????????????????????????????????????????????????????????????????????????????????????????????????????????????????????????????????????????????????????????????????????????????????????????????????????????????????????????????????????????????????????????????????????????????????????????????????????????????????????????????????????????????????????????????????????????????????????????????????????????????????????????????????????????????????????????????????????????????????????????????????????????????????????????????????????????????????????????????????????????????????????????????????????????????????????????????????????????????????????????????????????????????????????????????????????????????????????????????????????????????????????????????????????????????????????????????????????????????????????????GATTTTAAGGGTGGGGCGGGCTGGTTTGGTTTTAAAGTTGTTTTTATTATTTTTTTTTTT????????????????????????????????????GATCAGCTTATGGCTTTGTTATGAAAAGGGTATTTGCCTTTAAGTTTAGGAATGGTCATCTTTGTGGCTAGTGTTCTCTTTGGGTTTAATGGTCCCCCTCCTATTTAAAATATTTTAATTAATTATTATGAAAAAAAAAAAGAACTAACAGATTTGCAAGTGCCATTACGAAAAAAAAATCCTATTTTATTTTTAATCAACGGATTTTTAGTTGATTTGGTTTCTCCTTCTAACATTACTTATTTATGAAATTTTGGATCTTTATTGGGATTGTGTTTGATTTTACAGATAGTGACTGGGTGTTTTTTGTCTATGCACTATTGTTCTGATGTTAATTTTGCTTTTGCTTCAATTGGTCATATTATGCGAGATGTTAATTATGGGTTTTTATTAAGATATCTTCATGCTAACGGTGCTTCTTTGTTTTTTTTTTGTCTTTATGTTCATATTGGTCGAAGTTTATATTATGGGGGGT????????????????????????????????????????????????????????????????????????????????????????????????????????????????????????????????????????????????????????????????????????????????????????????????????????????????????????????????????????????????????????????????????????????????????????????????????????????????????????????????????????????????????????????????????????????????????????????????????????????????????????????????????????????????????????????????????????????????????????????????????????????????????????????????????????????????????????????????????????????????????????????????????????????TTTTATCTATTTTACATCAAAGTCTTTTAAAGGGACTTTTTTTTCGTCCTTTAGGTCGAATTGCCTTTTGGTTTTTAATTATTGATTTTGCTTTATTAACTTGAATTGGATCACAAGTTGTAGAAGAACCTTTTATTTTAATTGGTCAAATACTTTCTTTTTTTTATTTTTTTTATTTTTTAGTTTTAATACCAGTTTTGGGTATTATTGAAAATCAATTATTAAATAAAAATTAATGAAATTTTTGTTTTAGGCGGTTTTCTTTTGAGCTTCGTGGTTTTAAGTCTTCGTTATTTTCTTTTAAAACTTTCTCTTTTTT???????????TTTTTTTTTTTTTTTTTTTTTAAATTAGAATGGGGAAAGCTTTTGGTTTTAAT???????????????????????????????????????????????????????????????????????????????????????????????????????????????????????????????????????????????????????????????????????????????????????????????????????????????????????????????????????????????????????????????????????????????????????????????????????????????????????????????????????????????????????????????????????????????????????????????????????????????????????????????????????????????????????????????????????????????????????????????????????????????????????????????????????????????????????????????????????????????????????????????????????????????????????????????????????????????????????????????????????????????????????????????????????????????????????????????????????????????????????????????????????????????????????????????????????????????????????????????????????????????????????????????????????????????????????????????????????????????????????????????????????????????????????????????????????????????????????????????????????????????????????????????????????????????????????????????????????????????????????????????????????????????????????????????????????????????????????????????????????????????????????????????????????????????????????????????????????????????????????????????????????????????????????????????????????????????????????????????????????????????????????????????????????????????????????????????????????????????????????????????????????????????????????????????????????????????????????????????????????????????????????????????????????????????????????????????????????????????????????????????????????????????????????????????????????????????????????????TATGTTTC????????????????????????????????????????????????????????????????????????????????????????????????????GATCTTTTTTTCCAAATAAATAGGTAATGAACAGTTCTTCTTATTTTGAACAATTTAACATAGTGTGATTGTTTGGTTTTACGAATTCAACAATAATGATGACTTTTGTAATTATTGTAGTTTTATTATTTTTTAAAGGAATTGAATTAATTCCAAAAAGATGGCAGTCGGTTTATGAATGTCTAGAAAATTATKTTT??????????????????????????????????????????????????????????????????????????????????????????????????????????????????????????????????????????????????????????????????????????????????????????????????????????????????????????????????????????????????????????????????????????????????????????????????????????????????????????????????????????????????????????????????????????????????????????????????????????????????????????????????????????????????????????????????????????????????????????????????????????????????????????-----------------??????????????????????????????????????????????????????????????????????????????????????????????????????????????????????????---???????????????????????????????????????????????????????????????????????????????????????????????????????????????AATGATGTTGGGGTACAAGTGAATCAAGAGTCTGAACAGTCTGTGGATGTTGTTTTTATAGAATTGGAAGGGCGAAGGGTCCCCGTAAG--GGGGGTGA------------------CTCTACATGTTGAGCGGCGT-GGTTATTTGGATAATCG---GTCTAATCAAGAAGG--TGT-TGA---------------------------------------------TAATAGTTTAGGAGATCA-----ATTTGA-AGATGTGGCTTT---TTTAA-GAGAGAG-TACAAGTACAATTACACCAGAAA----GTGTAAATAGGGGCGCTCCAAGTGGTAATATAAGAAATGTATTAAGTGATTCATTAA-TAGA-G-AA-GAGAGGCCAAGACCAAACTTTAAGTGA--AA-TAAAGGTAGAAACACCTCC----AGGTGCTTCAAGGGGTGGCCGATG-A-------G--CCAGT-AT-----AAATCAAGGTGAGGCTTTAAG---AGAAAACAAGGTAGAAATTGGGG----TTTTAAGAGAAAACAAAGT--------AGAAAT--TGATGAGGTAGTTGGCTCGACTTTTAGTTCGATC????????????????????????????????????????????????????????????????????????????????????????????????????????????????????????????????????????????????????????????????????????????????????????????????????????????????????????????????????????????????????????????????????????????????????????????????????????????????????????????????????????????????????????????????????????????????????????????????????????????????????????????????????????????????????????????????????????????????????????????????????????????????????????????????????????????????????????????????????????????????????????????????????????????????????????????????????????????????????????????????????????????????????????????????????????????????????????????????????????????????????????????????????????????????????????????????????????????????????????????????????????????????????????????????????????????????????????????????????????????????????????????MGGCCCGATTGTTTTTGCAGTTGATGGTGTTTCTTTGGTTTTTTTACTTTTAACGACTTTTTTAATTCCAATTTGTATTTTAATTAGCCAAAAATCAATAAAATTCTTATTTAAAGAATTTCTTTTGTGCTTGTTTTTTTTAGAATTTTTATTAATAGGTGTTTTCATAGTGTTTGATCTTCTTTTGTTTTATCTTTTTTTTGAGGGGATATTAATACCAATGTTTTTTTTAATTGGTATTTGAGGCTCTCGAGAAGAAAAGGTTCGTGCTTCTTTTTATTTTTTTTTTTTTACTTTTATAGGCTCTCTCTTTTTCTTTTTTATAATACTTTTTTTATATCAAAGGATT???????????????????????????????????????????????????????????????????????????????????????????????????????????????????????????????????????????????????????????????????????????????????????????????????????????????????????????????????????????????????????????????????????????????????????????????????????????????????????????????????????????????????????????????????????????????????????TTGGGTCTTTTTACACATGTTATAGAGGGGTTAATTGGGGCTCTTTTTTTAATGTTGGCGCACGGATTTGTTAGCTCTGCTCTTTTTATTGGAGTAACTTTTTTGTATGATCGCCATCATACTCGTTTAATAAAATATTATCGGGGTTTGACTTTGACAATGCCTCTTTTTGTTATTACAATGTTAATTTTGTCTTTGGCGAACATGGGTTTTCCTCTTAGTTGTAATTTTGTTGGAGAGTTTTTTTCTTTATTAGCAG?CTTTC????????????????????????????????????????????????????????????????????????????????????????????????????????????????????????????????????????????????????????????????????????????????????????????????????????????????????????????????????????????????????????-------------??????????????????????????????????????????????????????????????????????????-??????????????????????????????????????????????????-?????????????????????????????????????????????????????????????????????????????????????????????????????????????????????????????????-????????????????????????????????????-???????????????????????????????????????????????????????????????????--??????????????????????????????????????????????????????------????????????????????????????????????????????????????????????????????????????????????????????????????????????????????????????????-?????????????????????????????????????????????????????????????????????????????????????????????????????????????????????????????????????????????????????????????????????????????????????????????????????????????????????????????????????????????????????????????????????????????????????????????????????????????????????????????????????????????????????????????????????????????????????????????????????????????????????????????????????????????????????????????????????????????????????????????????????????????????????????????????????????????????????????????????????????????????????????????????????????????????????????????????????????????????????????????????????????????????????????????????????????????????????????????????????????????????????????????????????????????????????????????????????????????????????????????????????????????????????????????????????????????????????????????????????????????????????????????????????????????????????????????????????????????????????????????????????????????????????????????????????????????????????????????????????????????????????????????????????????????????????????????????????????????????????????????????????????????????????????????????????????????????????????????????????????????????????????????????????????????????????????????????????????????????????????????????????????????????GATCCTATTGTAGAAGAAATTGTTTTTTTTCATGACCAAGTTATGTTTTTATTAATTATTATAGTTACTGTTGTTTTATGACTTTTTGTTGAAGCTTTTAAAAATAAGTTTTATGATCGTCATTTAATCGATGGTACATTTTTAGAAATTGTTTGAACGATTATTCCTGCTGTTATATTGATTTTTATTGCATTGCCCTCTCTTAAATTATTATATTTAATGGATGAGGTTATTTCTCCAGCTTTGACAATTAAAGTTATTGGGCATCAATGATATTGATC???????????????????????????????????????????????????????????????????????????????????????????????????????????????????????????????????????????????????????????????????????????????????????????????????????????????????????????????????????????????????????????????????????????????????????????????????????????????????????????????????????????????????????????????????????????????????????????????????ATTATTATTATTTTATTTTTATTGGGGAGTTGGGGTATAATTTTAAATAGAGGACATTTTATTATTATGCTTGTTTCTATTGAATTAGTTTTATTATCAACTTTTTTTTTTTTTTTAATAAGTTCTAAAGAAATAGATCTTTTAATAGAACAAATTTTTATGATTATGGGGTTAACTATTGCTGCTGCCGAATCTTCAATTGGTTTAGCTATTTTAGTTGCATATTATAGAATTCGAGGAACAATTGTTTTAAAATCTTTTAGTTCTTTGCGAGGATAATGATTAATCTTTTTATTTTTTTTTTTCTTATTTTTATTTTGGCGGGGTTATTTATATTTCTTTCTTTTTTGATAGGGGAAAAAATTCCAGATCGA?????????????????????????????????????????????????????????????????????????????????????TTTTATTCTTGATTTTT??????????????????????????????????????????????????????????????????????????????????????????????????????????????????????????????????????????????????????????????????????????????????????????????????????????????????????????????????????????????????????????????????????????????????????????????????????????????????????????????????????????????????????????????????????????????????????????????????????????????????????????????????????????????????????????????????????????????????????????????????????????????????????????????????????????????????????????????????????????????????????????????????????????????????????????????????????????????????????????????????????????????????????????????????????????????????????????????????????????????????????????????????????????????????????????????????????????TATCCAATATTAATTTAAAACGAGCAAACATTTTTTTTCTTAAAGAAGGAGAATTTTTATTTTTAATTCCTTTGGGCATATTAACTTTAGGAAGTGTTTTTTGGGGCTATTTAAGTAAAGAAATAATTTGGTCTTTTCAAATAGATGTTTTTTCAATACTTTCTTTAAAAATAAAAATATTTCCAATTTTATTTTGTTTTATTGGACTTTTTGGGACGATATTTTTTTTTTTTTTTTTTNCTTCTCAAATTTTTGGTTATCCCCTTCAGGTGATCGGATC?????????????????????????????????????????????????????????????????????????????????????????????????????????????????????????????????????????????????????????????????????????????????????????????????????????????????????????????????????????????????????????????????????????????????????????????????????????????????????????????????????????????????????????????????????????????????????????????????????????????????????????????????????????????????????????????????????????????????????????????????????????????????---------------?????????????????????????????????????????????????????????????????????????????????-????????--??????????????????????????-???????????????????????????????????????-????????????????-------------------------------------??????????------------?????????????----------------------------------------------??????????????????????---??????GATC-TTT---GGGGATGGTGTGGA--AAAAATGGAAGTAGATG-------ATGA--AGAGATAGTAGGAG--CGTTTCCATCTTTA--------AGCTTCGTGGAGGGGTGAGATG----------------------------AAGAGGTGATAGCAGCGTTTCCATGTTCAGACT----------CCGAAGAGAGTATGGAAATTGATGGGGAAGA--ACTACCTTTGGATC????????????????????--????????????--????????????-------------------------------????????????????????????????????????????????????????????????????????????????-????????????????????????????????????--??---?--??????????????---??????????????????????????????????????-??????---------------------??????????????????????????????????????????--???--??????????????????????????-?????????????-???-?????-??????????????????????????????--???????????????????????????????-??????????????????????????????????????????????????????????????????????????????????????--????????????????????????????????????????-?????????????????????????????????????????????????????????????????????????????????????????????????????????????????????????????????????????????????????????????????????????????????????????GATCATCTTTATAATGTAATTGTTACAGCACATGCTTTTATTATGATTTTTTTTTTGGTTATGCCGGTCATGATTGGGGGATTTGGTAATTGATTAGTCCCATTATATATTGGGGCGCCGGATA???????????????????????????????????????????????????????????????????????????????????????????????????????????????????????????????????????????????????????????GATATGGTTATTTTTAGTCTTCATTTAGCTGGGGTTTCTTCTATTTTAGGTGCTATTAACTTTATTACTACAATTTTTAATATGCGAGCCCCGGGTGTGTCTTTTAATAAACTACCTTTATTTATCTGATCTATTTTAATAACAGCTTTTTTATTACTTTTATCTTTACCTGTTTTAGCTGGTGCTATTACTATGTTGTTAACAGATAGAAACTTTAATACGACTTTTTTCGATCAGATGGGTGGCGGGGACCCAAKATTATTTCAGCATTTATTTTGAYTCTTTGGGCATCCAGAAGTTTATATTTTAATTTTGCCTGGTTTTGGTATGATTTCTCAAATAATCCCGACTTTTGTTGCTAAA????????????????????????????????????????????????????????????????????????????????????????????????????????????????????????????????????????????????????????????????????????????????????????????????????????????????????????????????????????????????????????????????????????????????????????????????????????????????????????????????????????????????????????????????????????????????????????????????????????????????????????????????????????????????????????????????????????????????????????????????????????????????????????????CAGGATTTCCAAGACGATATTCGGATTATGCAGATGCTTTTTTGGGTTGAAATTTAATAAGTTCTTTAGGGTCTATTATTTCTATTTTGAGTGTTGTTTGGTTTTTATATATTGTTTTTGATCTTTTTGTTACAGAAGAAAAATTTTTGGGTTGAAAAGAAGGATTTTCTTTAGAATGAATTCATTCTTCTCCCCCCTTATTTCATACTTATGAGGAGTTGCCCTTTGTACAAAAAGTAAATAATTTTTAGAATAGTGCCGGGTTTATATACCGGTTT

SD4 ?????????????????????????????????????????????????????????????????????????????????????-???????????????????????????????????????????????TTCGAAATGGCGTTCAGTTGCTTATTGGTTGTTTCTTCAGAAAAGTGA--TTTTTTTTTATTTGAATTGAAACATCTTATTACTAAGTAAAAATCAAACGAGATTCCGAGAGTAGTGGAGAGTGAAATTGGAGTTGTGCTTGTGGTGGTCATAGATC????????????????????????????????????????????????????????????????????????????????????????????????????????????????????????????????????????????????????????????????????????????-?????????????????????????????????????????????????????????????????????????????????????????????????????????????????????????????????????????????????????????????????????-??????????????????????????????????????????????????????????????????????????????????????????????????????????????????????-??????????????????????????????????????????????????????????????????????????????????????????????????????????????????????????????????????????????????????????????????????????????????????????????????-?------------------????????????????????????????????????????????????????????????????????????????-???????????????????????????????????????????????????????????????????????????????????????????????????????????????????????????????????????????????????????????????????????????????????????????????????????????????????????????????????????????????????????????????????????????????????????????????????????????????????????????????????????????????????????????????????????????????????????????????????????????????????????????????????????????????????????????????????????????????????????????????????????????????????????????????????????????????????????????????????????????????????????????????????????????-?????????????????????????????GATCCGTTATTTTGAATGAAAAAAATAACGAAAACAAATAAAAGTTACCCTGGGGATAACAGCGCAATAATGTTTGAGAGTTAATCAACGACAGTGTTTGCGACCTCGATGTTGAATTGTAACGTCCTACGGTGTAGTCACTCGTAAGGGTTGGTTTGTTCATCCATTAAAGTTATACATGATTTGAGTTAAAAGCGTGGTGACACAGCTTGGTTTCTATCTACAATTAGAAAACAAAAATATGTTGTTTTTTCGTACGAAAGGATCAAAAATCAATAGTTCTCTTAATATAACTATTCTTTAAATTAGGATTGTTTCTAAAAATAAAAAGAAATATTTTTGTTTGTTGTTTTTATTAATGTATCTTTTAGTTTTATTTTTTCCTTTAATTGGAGCTGTTTTAACAGGTTGTTTTGGAAGAAAAATCGGAGAAAGAGGAGCGGGGATTTTAACTTCAAGTTGTTTAGTTTTTAGTTTATCTTAT?????????????????????????????????????????????TATTTGAAATTATGAAAATGATTTGATTCGGGGTTGTTTGTTGTTTTTTTTGGTTTTCAATTTGATGGTTTAGTAGTTATTATGTTATTTGTTGTTTTTATTGTTTCTACTTTGGTTCATATTTTTTCTATTGCTTATATGCGGGGAGATCCTCATATTCCTCGATTTATGACATATCTTTCTTTGTTTACTTTTTTAATGGTTTTATTGGTAACTAGCGATAATTTTCTTCAATTATTTATTGGGTGAGAAGGGGTTGGTCTTTGTTCTTATTTATTAATTAATTTTTGATTAACTCGATTAGAGGCAAATAGAGCTGCTATTAAAGCAATGTTAGTGAATAGAATTGGTGATATTGGGTTGCTTTTAGCAATGTTTTTACTTTGGGATC??????????????????????????????????????????????????????????????????????????????????????????????????????????????????????????????????????????????????????????????????????????????????????????????????????????????????????????????????????????????????????????????????????????????????????????????????????????????????????????????????????????????????????????????????????????????????????????????????????????????????????????????????????????????????????????????????????????????????????????????????????????????????????????????????????????????????????????????????????????????????????????????????????????????????????????????????????????????????????????????????????????????????????????????????????????????????????????????????????????????????????????????????????????????????????????????????????????????????????????????????????????????????????????????????GTTACAATAATGTTTTTTGCTTCGATTTTAGCAGAGACCAATCGTGCTCCTTTTGATTTAACAGAAGGAGAGTCAGAGCTTGTTTCGGGATATAATGTTGAGTATGCTTCGA????????????????????????????????????????????????????TTGTTTAACAACGATTTTTTTTGGGGGAGGATGGCTTTCTCCGATTCCAGATTTTAAGGGTGGGGCGGGCTGGTTTGGTTTTAAAGTTGTTTTTATTATTTTTTTTTTTATTTGAGTAAGGGCTTCATTTCCTCGAATTCGATATGATCAGCTTATGGCTTTGTTATGAAAAGGGTATTTGCCTTTAAGTTTAGGAATGGTCATCTTTGTGGCTAGTGTTCTCTTTGGGTTTAATGGTCCCCCTCCTATTTAAAATATTTTAATTAATTATTATGAAAAAAAAAAAGAACTAACAGATTTGCAAGTGCCATTACGAAAAAAAAATCCTATTTTATTTTTAATCAACGGATTTTTAGTTGATTTGGTTTCTCCTTCTAACATTACTTATTTATGAAATTTTGGATC???????????????????????????????????????????????????????????????????????????????????????????????????????????????????????????????????????????????????????????????????????????????????????????????????????????????????????????????????????????????????????????????????????????????????????????????????????????????????????????????????????????????????????????????????????????????????????????????????????????????????????????????????????????????????????????????????????????????????????????????????????????????????????????????????????????????????????????????????????????????????????????????????????????????????????????????????????????????????????????????????????????????????????????????????????????????????????????????????????????????????????????????????????????ACCAAATAAATTAGGTGGGGTTATTGCAATGTTTTGTAGTATTTTTATTTTATTTTTTTTATCTATTTTACATCAAAGTCTTTTAAAGGGACTTTTTTTTCGTCCTTTAGGTCGAATTGCCTTTTGGTTTTTAATTATTGATTTTGCTTTATTAACTTGAATTGGATC???????????????????????????????????????????????????????????TTTATTTTTTAGTTTTAATACCAGTTTTGGGTATTATTGAAAATCAATT???????????????????????????????????????????????????????????????????????????????????????????????????????????????????????????????????????????????????????????????????????????????????????????????????????????????????????????????????------------------------------------------------------------------------------------------------------------------------------------------------------------------------------------------------------------------------------------------------------------------------------------------------------------------------------------------------------------------------------------------------------------------------------------------------------------------------------------------------------------------------------------------------------------------------------------------------------------------------------------------------------------------------------------------------------------------------------------------------------------------------------------------------------------------------------------------------------------------------------------------------------------------------------------------------------------------------------------------------------------------TCTAA----------------------------------------------AT????????TTTTTCTTTCTCCACATTTTCTTTTTTTTTTTTTTTCTCAAATAATTTTTGATTTATTTTAAAATGGAATTTTTTTCTTTTTTTTTT?????????ATAATTGGCTCAGGAGTAATGGTTGTTTCAGCGTTAAATCCTGTTCTTTCTATTTTTTGATTGGTTCTTGTTTTTATAAATTCTGCCGTTTTTTTCCTTTTACTAGGAATAG????????????????????????????????????????????????????????????????????????????????????????????????????????????????????????????????????????????????????????????????????????????????????????????????????????????????????????????????????????????????????????????????????????????????????????????TATGTTTCTTGTTTTTGTT?????????????????????????????????????????????????????????????????????????????????????????GATCTTTTTTTCCAAATAAATAGGTAATGAACAGTTCTTCTTATTTTGAACAATTTAACATAGTGTGATTGTTTGGTTTTACGAATTCAACAATAATGATGACTTTTGTAATTATTGTAGTTTTATTATTTTTTAAAGGAATTGAATTAATTCCAAAAAGATGGCAGTCGGTTTATGAATGTCTAGAAAATTATTTTTATTATATAACAGTGCAAAATTTAAGTAATGTGGGTTTAATATATTTTTCTTTTATTGTTTCCCTT?????????????????????????????????????????????????????????????????????????????????????????????????????????????????????????????????????????????????????????????????????????????????????????????????????????????????????????????????????????????????????????????????????????????????????????????????????????????????????????????????????????????????????????????????????????????????????????????????????????????????????????????????????????????????????????-----------------??????????????????????????????????????????????????????????????????????????????????????????????????????????????????????????---????????????????????????????????????????????????????????????????????????????????????????????????????????????????????????????????????????????????????????????????????????????????????????????????????????--????????------------------????????????????????-?????????????????---???????????????--???-???---------------------------------------------?????????????GATCA-----ATTTGA-AGATGTGGCTTT---TTTAA-GAGAGAG-TACAAGTACAATTACACCAGAAA----GTGTAAATAGGGGCGCTCCAAGTGGTAATATAAGAAATGTATTAAGTGATTCATTAA-TAGA-G-AA-GAGAGGCCAAGACCAAACTTTAAGTGA--AA-TAAAGGTAGAAACACCTCC----AGGTGCTTCAAGGGGTGGCCGATG-A-------G--CCAGT-AT-----AAATCAAGGTGAGGCTTTAAG---AGAAAACAAGGTAGAAATTGGGG----TTTTAAGAGAAAACAAAGT--------AGAAAT--TGATGAGGTAGTTGGCTCGACTTTTAGTTCGATC?????????????????????????????????????????????????????????????????????????????????????????????????????????????????????????????????????????????????????????????????????????????????????????????????????????????????????????????????????????????????????????????????????????????????????????????????????????????????????????????????????????????????????????????????????????????????????????????????????????????????????????????????????????????????????????????????????????????????????????????????????????????????????????????????????????????????????????????????????????????????????????????????????????????????????????????????????????????????????????????????????????????????????????????????????????????????????????????????????????????????????????????????????????????????????TTTAAAAAAGCGGGCTTTAGAGTGGTCTTTGGCTTTATTGTTTAGTACTTTAGTTTTTTGGGGTGGATTTGACGGAGAAAGTCATTTTCAATTTTTTAGTTTAGTAGAATGAAATATATTTTCTACTTTAGATTGAGGCCCGATTGTTTTTGCAGTTGATGGTGTTTCTTTGGTTTTTTTACTTTTAACGACTTTTTTAATTCCAATTTGTATTTTAATTAGCCAAAAATCAATAAAATTCTTATTTAAAGAATTTCTTTTGTGCTTGTTTTTTTTAGAATTTTTATTAATAGGTGTTTTCATAGTGTTTGATC????????????????????????????????????????????????????????????????????????????????????????????????????????????????????????????????????????????????????????????????????????????????????????????????????????????????????????????????????????????????????????????????????????????????????????????????????????????????????????????????????????????????????????????????????????????????????????????????????????????????????????????????????????????????????????????????????????????????????????????????????????????????????????????????????????????????-------------------------------------------------------------------------------------------------------------------------------------------------------------------------------------------------------------?????????????????????????????????????AATTTT-----------------------------------------------------------------------------------------------------------T?????????????????????????????????????----------------------------------------------------------???????????????????-------------------------------------------------------------------------------------------------------------------------------------------------------------------------------------------------------------------------------------------------------------------------------------------------------------------------------------------------------------------------------------------------------------------------------------------------------------------------------------------------------------------------------------------------??????????????????????????????????????????????????????????????????????????????????????????????-?????????????????????????????????????????????????????????????????????????????????????????????????????????????????????????????????????????????????????????????????????????????????????????????????????????????????????????????????????????????????????????????????????????????????????????????????????????????????????????????????????????????????????????????????????????????????????????????????????????????????????????????????????????????????????????????????????????????????????????????????????????????????????????????????????????????????????????????????????????????????????????????????????????????????????????????????????????????????????????????????????????????????????????????????????????????????????????????????????????????????????????????????????????????????????????????????????????????????????????????????????????????????????????????????????????????????????????????????????????????????????????????????????????????????????????????????????????????????????????????????????????????????????????????????????????????????????????????????????????????????????????????????????????????????????????????????????????????????????????????????????????????????????????T?????????????????????????????????????????????????????????????????????????????????????????????????????????????????????????????????????SMGY---------------ATCCTATTGTAGAAGAAATTGTTTTTTTTCATGACCAAGTTATGTTTTTATTAATTATTATAGTTACTGTTGTTTTATGACTTTTTGTTGAAGCTTTTAAAAATAAGTTTTATGATCGTCATTTAATCGATGGTACATTTTTAGAAATTGTTTGAACGATTATTCCTGCTGTTATATTGATTTTTATTGCATTGCCCTCTCTTAAATTATTATATTTAATGGATGAGGTTATTTCTCCAGCTTTGACAATTAAAGTTATTGGGCATCAATGATATTGATC??????????????????????????????????????????????????????????????????????????????????????????????????????????????????????????????????????????????????????????????????????????????????????????????????????????????????????????????????????????????????????????????????????????????????????????????????????????????????????????????????????????????????????????????????????????????????????????????????????????????????????????????????????????????????????????????????????????????????????????????????????????????????????????????????????????GATCTTTTAATAGAACAAATTTTTATGATTATGGGGTTAACTATTGCTGCTGCCGAATCTTCAATTGGTTTAGCTATTTTAGTTGCATATTATAGAATTCGAGGAACAATTGTTTTAAAATCTTTTAGTTCTTTGCGAGGATAATGATTAATCTTTTTATTTTTTTTTTTCTTATTTTTATTTTGGCGGGGTTATTTATATTTCTTTCTTTTTTGATAGGGGAAAAAATTCCAGATCGAGAAAAGGTTTCTGCTTATGAGTGTGGTTTTGCTCCCTTTAATTTTTTAGGACGCCCTTTTTCAATACGTTTTTTTTTAATTGGTATTTTATTCTTGATTTTTGATTTAGAAATTTCTTTTTTTTTTCCCTGATGTGTTTTATATAATTCGACTGCTCCGTTT???????????????????????????????????????????????????????????????????????????????????????????????????????????????????????????????????????????????????????????????????????????????????????????????????????????????????????????????????????????????????????????????????????????????????????????????????????????????????????????????????????????????????????????????????????????????????????????????????????????????????????????????????????????????????????????????????????????????????????????????????????????????????????????????????????????????????????????????????????????????????????????????????????????????????????????????????????????????????????????????????????????????????????????????????????????????????????????????????????????????????????????????????????????????????????????????????????????????????????????????????????????????????????????????????????????????????????????????????????????????????????????????????????????????????????????????????????????????????????????????????????????????????????????????????????????????????????????????????????????????????????????????????????????????????????????????????????????????????????????????????????????????????????????????????????????????????????????????????????????????????????????????????????????????????????????????????????????????????????????????????????????????????????????????????????????????????????????????????????????????????????????????????????????????????????????????????????????????????????????????????????????????????????????????????????????????????????---------------?????????????????????????????????????????????????????????????????????????????????-????????--?????????TTGTTTAAAATATTTTT-TTGCGTTTTCTTACTTTAAAACATCTTTTTGTTTAATGA-TTTTTTTTTTTTTNAT-------------------------------------AAAATTTGGT------------TCTAAGAAGATTC----------------------------------------------GGGCTCGTTTAGCGATC?????---????-------???---??????????????--???????????????????-????????--??????????????--??????????????--------??????????????????????----------------------------?????????????????????????????????----------???????????????????????????????--??????????????????????????????????--????????????--????????????-------------------------------????????????????????????????????????????????????????????????????????????????-????????????????????????????????????--??---?--??????????????---??????????????????????????????????????-??????---------------------??????????????????????????????????????????--???--??????????????????????????-?????????????-???-?????-??????????????????????????????--???????????????????????????????-??????????????????????????????????????????????????????????????????????????????????????--????????????????????????????????????????-????????????GAAGCCCCGTCTAAATGCTTTCTGGTTATTGAACTCGATGGGTGTTTTCTACAAATCATAAGGACATTGGTACTTTGTATTTAATCTTCGGAGCAGGAGCTGGTTTAATTGGAACTGCTTTTAGTATGCTTATACGATTGGAGCTTTCTGCGCCGGGGGCGATGTTAGGAGATGATCATCTTTATAATGTAATTGTTACAGCACATGCTTTTATTATGATTTTTTTTTTGGTTATGCCGGTCATGATTGGGGGATTTGGTAATTGATTAGTCCCATTATATATTGGGGCGCCGGATATGGCGTTTCCT?????????????????????????????????????????????????????????????????????????????????????????????????????????????????????????????????????????????????ATATGGTTATTTTTAGTCTTCATTTAGCTGGGGTTTCTTCTATTTTAGGTGCTATTAACTTTATTACTACAATTTTTAATATGCGAGCCCCGGGTGTGTCTTTTAATAAACTACCTTTATTTATCTGATCTATTTTAATAACAGCTTTTTTATTACTTTTATCTTTACCTGTTTTAGCTGGTGCTATTACTATGTTGTTAACAGATAGAAACTTTAATACGACTTTTTTCGATCAGAT????????????????????????????????????????????????????????????????????????????????????????????????????????????????????????????????------------------------------------------------------------------------------------------------------------------------------------------------------------------------------------------------------------------------------------------------------------------------------------------------------------------------------------------------------------------------------------TTGGGGGGTTTTACTATTGAATTGGCAAAATAAGTGGTTATTGTTATAATGAATTTTT??????????????????????????????????????????????TTTTTCCCTCAACATTTTTTAGGTT???????ATTTCCAAGACGATATTCGGATTATGCAGATGCTTTTTTGGGTTGAAATTTAATAAGTTCTTTAGGGTCTATTATTTCTATTTTGAGTGTTGTTTGGTTTTTATATATTGTTTTTGATC???????????????????????????????????????????????????????????????????????????????????????????????????????????????????????????????????????????????????????????

Pdam TGTAAGAAAGACAAAGGGTTAGTCATTGGGTTCATGCCCCAACCAAGTGAGTTCGAATCTCTCTCTTACACAAAATAAAAATTAT-AAAAAAAAAAGAGGATAACTAAGATAAACTAAAATTAGACGATTTTTTTCGAAATGGCGTTCAGTTGCTTATTGGTTGTTTCTTCAGAAAAGTGA--TTTTTTTTTATTTGAATTGAAACATCTTATTACTAAGTAAAAATCAAACGAGATTCCGAGAGTAGTGGAGAGTGAAATTGGAGTTGTGCTTGTGGTGGTCATAGATCTTGCTTAAACATTAGTTTATACTGATAATGAAAGTACTGTAAAGGAAAGTTGAAAGAGAGTTGAAAGGAATTTGAATCTTTTATTTTTGAAGTAGCTTTAAAAAAGCGTACCTTTTGTATAATGGGTAAAAGAGATTTATTTGGCATATTTAAAATGGAAAATTTTAAGTAA-TTTTTTTTTAGTCAAATTTCCCGAAACCAAGTGATTTAATCATGAATTGTGTGAGCAAAAACTGGTGATTGTGGCAAAAATCTCAGAAAATTTGTGATTAGGGGTGAAAGGCTAATCGAACTTGGAAATAGCTGGTTTTCTGCGAAAACTATTTAAGTAGTGTTCTTTTTTTTTTTTATTTTAATTGTCATTTTAAAAAAAACAAAAATAAAAAAAAAGTAAACAAACAATAAATGAAAAGATTTTTTGTTAAAAGAGAAAGCTCAAATCAGAAGCTATTGTCA-TTTTTTTTTTTTTAGTTTAAAAAAAAATTAAATTTAGACAATAAAAATGTAGGCTTGGAACCAGCCATCTTTTAAAAAGTACGTAGTTGTTTATTTAAAAATTTTAAGTTTTTTTATAATTTTGATGGTTAAAATAAAACAGAAGCTCTGAAAATAATTATATATTTCGTAGCAGAATAAATTGTTTTCGATAT-C------------------TCCCTAGTTTGAAATTTTTTTTCTTAATACATCGTGTTCAGTTGTAATAATTTTTACATAAGTAATCTAAATATTA-TTTTTTTTTCTTAAGATTACCTTAGTCAAGGGTAATACAGTTTCTAAAAAAGAAATTTTTTATGAAGATTTTTTATAAAAATATATAATATCGGGAAAAGAAAAAAAAAAGAACTGTTTTTCTAATTAAAGAAAGAAAAAAAAAAAGAAAAACATTTTTTTAAGGAACTCGGCAAAGTTAAACTTCGACTGTTTACCAAAAACATAGCTTTTTGATTTTTATAAAAGGTGAAACCTGCCCGATGGTTGAATCTTAATATGTTTGTTCCGCTTACTAATAAAGACAATTAAATGGCCGCGGTAACACTGACTGTGATAATGTAGCGTAATCAATTGTCAATTAATTGTTGACCGGTATGAATGGTATCCCGAAAGTTTTTCTGTCTTAAAAAAATATTCAATGAAATTAAATCTGTAGTGAAGATGCTACATTTTAATTGTTAGACGAGAAGTCCCCATGGAGCTTTACTGTAAGCTTATATATATAATTTTTTTTTTTTTATAAGTAAGACAGTTTTGTTGGGGCGACAGTTTTTTAAAAAGTAACGAAAATGAGCTATGACGCATGTTTAACTTTGAAATTTTTTT-ATTGATGAGACATTTTTGGTGTGTTTTTTGATCCGTTATTTTGAATGAAAAAAATAACGAAAACAAATAAAAGTTACCCTGGGGATAACAGCGCAATAAYGTTTGAGAGTTAATCAACGACAGTGTTTGCGACCTCGATGTTGAATTGTAACGTCCTACGGTGTAGTCACTCGTAAGGGTTGGTTTGTTCATCCATTAAAGTTATACATGATTTGAGTTAAAAGCGTGGTGACACAGCTTGGTTTCTATCTACAATTAGAAAACAAAAATATGTTGTTTTTTCGTACGAAAGGATCAAAAATCAATAGTTCTCTTAATATAACTATTCTTTAAATTAGGATTGTTTCTAAAAATAAAAAGAAATATTTTTGTTTGTTGTTTTTATTAATGTATCTTTTAGTTTTATTTTTTCCTTTAATTGGAGCTGTTTTAACAGGTTGTTTTGGAAGAAAAATCGGAGAAAGAGGAGCGGGGAYTTTAACTTCAAGTTGTTTAGTTTTTAGTTTATCTTATTCTTTTTTAATCGCGATTGAAGTTTTATTTAATTCAACAACAACGTATTTGAAATTATGAAAATGATTTGATTCGGGGTTGTTTGTTGTTTTTTTTGGTTTTCAATTTGATGGTTTAGTAGTTATTATGTTATTTGTTGTTTTTATTGTTTCTACTTTGGTTCATATTTTTTCTATTGCTTATATGCGGGGAGATCCTCATATTCCTCGATTTATGACATATCTTTCTTTGTTTACTTTTTTAATGGTTTTATTGGTAACTAGCGATAATTTTCTTCAATTATTTATTGGGTGAGAAGGGGTTGGTCTTTGTTCTTATTTATTAATTAATTTTTGATTAACTCGATTAGAGGCAAATAGAGCTGCTATTAAAGCAATGTTAGTGAATAGAATTGGTGATATTGGGTTGCTTTTAGCAATGTTTTTACTTTGGGATCTTTTTGGATCTCTAGATTTTTCTACTATTTTTAATTCTATTTTTTTTTCTAATCAAATATTTTTTATTTGTTTATTTTTATTTTTTGGGGTTATGGGAAAATCTGCTCAATTAGGATTACATACTTGGCTACCGGATGCAATGGAAGGTTATTGGGCCTTTTAATTAAAAAATTAATTAAAAAAATTACTATACATTAAAAAAACAATTTGTTAGTAAAATATTTGTTTATTAATGGACAATAAAATATTTATTTTAATGTCTAAGAGACTTTATGTGATTTACTTTTTTTTATTTGATTAAATCTATTATTATTATTATTCCTTTACTTATTGCTGTTGCATTTTTAACTTTAGCAGAACGAAAAATTTTAGGGTATATGCAAATAAGAAAAGGGCCAAATGTTGTTGGGGGAGGGCTTCTTCAACCTTTTGCAGATGGGGTGAAATTGTTTATTAAAGAAATGATTCTTCCCCATCAAGCAAATAAATTTGTTTATCTTTTGGCTCCAGTTATATCTTTTACATTAGCTTTCTTTGTTTGAGGTTTTCTTCCTTATGAGAAAGGAGTGAGTATTAGTGATTTTAAAATTAGTCTTTTATGAATTTTGGCTATTTCTTCTATTAGTGTTTATGCAATTTTAATGTCTGGATGAGGAAGTAAGTCTAAATATGCTTTTTTGGGTGCTATTCGGGCGGCGGCTCAAATGATTAGTTATGAGGTTTCAATTGGGCTAATTCTTATTTCGGTTTTATTATGTGTTGGTTCTTTAAGTGTTACTGAAATTGTTTTAGCGCAAAATAATGGTATTTGGTTTTTTTTTCCACTATTTCCTGTTACAATAATGTTTTTTGCTTCGATTTTAGCAGAGACCAATCGTGCTCCTTTTGATTTAACAGAAGGAGAGTCAGAGCTTGTTTCGGGATATAATGTTGAGTATGCTTCGATGTCTTTTGCTTTATTTTTTCTTGCTGAATATGCCCATATTATTTTAATGAGTTGTTTAACAACGATTTTTTTTGGGGGAGGATGGCTTTCTCCGATTCCAGATTTTAAGGGTGGGGCGGGCTGGTTTGGTTTTAAAGTTGTTTTTATTATTTTTTTTTTTATTTGAGTAAGGGCTTCATTTCCTCGAATTCGATATGATCAGCTTATGGCTTTGTTATGAAAAGGGTATTTGCCTTTAAGTTTAGGAATGGTCATCTTTGTGGCTAGTGTTCTCTTTGGGTTTAATGGTCCCCCTCCTATTTAAAATATTTTAATTAATTATTATGAAAAAAAAAAAGAACTAACAGATTTGCAAGTGCCATTACGAAAAAAAAATCCTATTTTATTTTTAATCAACGGATTTTTAGTTGATTTGGTTTCTCCTTCTAACATTACTTATTTATGAAATTTTGGATCTTTATTGGGATTGTGTTTGATTTTACAGATAGTGACTGGGTGTTTTTTGTCTATGCACTATTGTTCTGATGTTAATTTTGCTTTTGCTTCAATTGGTCATATTATGCGAGATGTTAATTATGGGTTTTTATTAAGATATCTTCATGCTAACGGTGCTTCTTTGTTTTTTTTTTGTCTTTATGTTCATATTGGTCGAAGTTTATATTATGGGGGGTATTTAAAATTTCATGTTTGAAGCATTGGAGTTGTTATTTTTTTATTAACAATGGCTATTGCCTTTATGGGCTATGTTTTGCCTTGAGGACAAATGTCTTTTTGAGGGGCAACTGTAATAACGAATTTATTATCTGCTATTCCTTATTTTGGGATTGATATTGTTCAGTGAGTGTGAGGCGGGTTTAGTGTTTCTAATGCAACATTAAATCGGTTTTTTAGTTTACATTTTTTACTTCCTTTTATTTTAGTTTTTCTTGTTATTCTTCATTTAGTTTATTTACATGTTGATGGGTCTAATAATTCAACAGGATTAAATTCTTCAATTGACGGAGTTTCATTCCATACTTTTTATACATCAAAAGATTTTTTTGGTTTTTTTTTTCTTTTTTTTTTATTTTGTTTTTTTGTTTTTTTTTTGCCTAATTTATTAGGAGATGCTGAAAATTTTATTCAAGCGAATTCTTTGGTTACTCCGGTTCATATTCAACCAGAATGATATTTTTTATTTGCTTATGCAATATTACGTTCTATACCAAATAAATTAGGTGGGGTTATTGCAATGTTTTGTAGTATTTTTATTTTATTTTTTTTATCTATTTTACATCAAAGTCTTTTAAAGGGACTTTTTTTTCGTCCTTTAGGTCGAATTGCCTTTTGGTTTTTAATTATTGATTTTGCTTTATTAACTTGAATTGGATCACAAGTTGTAGAAGAACCTTTTATTTTAATTGGTCAAATACTTTCTTTTTTTTATTTTTTTTATTTTTTAGTTTTAATACCAGTTTTGGGTATTATTGAAAATCAATTATTAAATAAAAATTAATGAAATTTTTGTTTTAGGCGGTTTTCTTTTGAGCTTCGTGGTTTTAAGTCTTCGTTATTTTCTTTTAAAACTTTCTCTTTTTTATTTATTATTATTTTTTTTTTTTTTTTTTTTTAAATTAGAATGGGGAAAGCTTTTGGTTTTAATTGGTTCTTTTGCTGTCTTTCTTTTATTTGGACCAAAAAAAGAAGAACAAACAGATGTCCCTATTTTAAGTTTAATTATTATTTTTGGAGTTTTTTGTTTAATTTCTTCAAGTAATTGACTTTCTATTTATTTAACCATTGAGCTTTTTACTCTTTGTTTTTTTATTTTGATTGCTCGAGGGTCTGGTTATAGTGTGGAAGCAGGATTAAAATATTTTATTTTGGGTGCTCTTTCTTCTGGTTTATTTTTATTTGGGTGTGCTTTATTATGTGGTATTGGGGCGAATATACACTTTTCTCATATAGAACTTCTTTTTAATTCAAAACAAATTTTTTCTGCTGTTTCGATACCAATTGGGTATCTTTTAATTATTGTTGCTCTTTTTTTTAAATTATCAGTTGCTCCTTTTCATATGTGAGTTCCTGATGTTTATGAGGGGGGACCTACTAAAATTATTCTATTATTGGCTATTGTTCCAAAAATAGGGTTTTTTTCTCTTATAATTTCAATTGGATTGCCTGTAAATTTTTTTTTTTTAGGGATTCTTTTTTCTTTGTTTGTTGGAGCTTTGGGTGCTTTAAATCAAACTAAAATAAAACGACTTTTGGCTTATAGTGGGATTGGTCATATGGGCTTTATTTTATGAGGTTTGGAAAATGGTTCTTTTGAAAGTTTACAAGCTAGTCTTGTTTATCTTTTTATATATATTGTGATGACAATTTGTGCTTTTTCTATTATATTAAGTTTTAATGTTTATAAAAATTTACTTGTTGAATTTAGTGGACTTTCTCGATACTTACCTTTTTTTTCGATTACTTTAGGTGTTCTTTTTTTTTCTATTGCAGGAATTCCTCCTTTTGCTGGCTTTTTTGGAAAATGATTTATTTTGTTATCTGGAATTCTTTCTAAATCATATTTTATCTTTTTTTTTGCTGTTTTTTGTTCTGTTATAGCTGGGGTTTATTATATTCGAATTATAAAAATACTTTTTTTTCAAAAAAATTCTTTTCTTTTAATTACAATTAAAGTTTTAAAAAAAGAATCTAAATTAAATTTTAAAAAAGTTTTTTTAATTGGTTTTTGTTTTTATTTTATTCTTTTTTTTTTTCTTTCTCCACATTTTCTTTTTTTTTTTTTTTCTCAAATAATTTTTGATTTATTTTAAAATGGAATTTTTTTCTTTTTTTTTTATTTTGGGGATAATTGGCTCAGGAGTAATGGTTGTTTCAGCGTTAAATCCTGTTCTTTCTATTTTTTGATTGGTTCTTGTTTTTATAAATTCTGCCGTTTTTTTCCTTTTACTAGGAATAGATTTTCTTGCTTTGATGTTTTTACTTATTTATGTTGGGGCAATAGCTATTTTATTTTTATTTGTTATTATGTTATTAAATCTAACTGACTACCCTCCTGTTTTAAAAAGAGAGGTTGATATGACAAATTATATACCAATTGGATTTATAATTGGGATTTTTTTTTTTTCAGAAATTGCTTCAAGTGGATTACTTTTGGGTTCTTTTCAAATAGAGAATTGAGACCTTTCTTTTCCTTGATTTCTTATTTCTTATCATAATATTGAGGCCTTAGGGCAGATTTTATATGTTTCTTGTTTTTGTTTATTTCTTTTGGCCAGTTTTATTTTATTAGTTGCTATGATGGGTGTGATTGTATTAACTCAAGAAACAGAATCTTTAAGTAAAAAACAAGATCTTTTTTTCCAAATAAATAGGTAATGAACAGTTCTTCTTATTTTGAACAATTTAACATAGTGTGATTGTTTGGTTTTACGAATTCAACAATAATGATGACTTTTGTAATTATTGTAGTTTTATTATTTTTTAAAGGAATTGAATTAATTCCAAAAAGATGGCAGTCGGTTTATGAATGTCTAGAAAATTATTTTTATTATATAACAGTGCAAAATTTAAGTAATGTGGGTTTAATATATTTTTCTTTTATTGTTTCCCTTTTTGTTTTTTTAGTTTTTTTAAATATTTTAGGTTTATGTCCTTATGTTTTTACTCCGACAACTCATATTATTGTTACTCTTGGGTTTTCTTTTTCTATTGTTATTGGAGTAACTTTTTCTGGATTTTATAAATTTAAAAAAGATTTTTTTAGTATTTTAATGCCTAGTGGGGCTCCTTTAATCTTGGCACCACTTTTAGTTTTAATAGAAACAGTAAGTTATATTTCTAGGGCTGTTTCTTTGGGGATTCGTTTAGCAGCAAATCTTTCTGCCGGGCATCTTTTATTTGCAATTTTGGCAGGATTTGGTCTGGTTTTTAAATTTGCAATGTTTATAATGGTGTTTATTACGCTATTAGAGGTGGCAGTTGCAATAATACAGGCCTATGTGTTTTGTCTGTTGGTACAAATTTATTTAACAGATACAATTTTTTTACATTAA-----------------AAATGACAGCCGTAGAAATTTTTTTATGCACAATTGTAGGTATATCGTTAACGGTGAATTATGGATTTGGTATTGGGTGATATGGTAGTTCTTCTCAAGCCTGGTTAAGGCACCAAATGGGC---ATAGACGGTGCAGAGAGGGTCCATCCTTGAGCTTATTTTAATTATGTGCGCCAGCATTCTATTGCTCGTAATGTTGGGGTACAAGTGAATCTAGATTCAACTGAAGGACCAAATGATGTTGGGGTACAAGTGAATCAAGAGTCTGAACAGTCTGTGGATGTTGTTTTTATAGAATTGGAAGGGCGAAGGGTCCCCGTAAG--GGGGGTGA------------------CTCTACATGTTGAGCGGCGT-GGTTATTTGGATAATCG---GTCTAATCAAGAAGG--TGT-TGA---------------------------------------------GAATAGTTTAGGAGATCA-----ATTTGA-AGATGTGGCTTT---TTTAA-GAGAGAG-TACAAGTACAATTACACCAGAAA----GTGTAAATAGGGGCGCTCCAAGTGGTAATATAAGAAATGTATTAAGTGATTCATTAA-TAGA-G-AA-GAGAGGCCAAGACCAAACTTTAAGTGA--AA-TAAAGGTAGAAACACCTCC----AGGTGCTTCAAGGGGTGGCCGATG-A-------G--CCAGT-AT-----AAATCAAGGTGAGGCTTTAAG---AGAAAACAAGGTAGAAATTGGGG----TTTTAAGAGAAAACAAAGT--------AGAAAT--TGATGAGGTAGTTGGCTCGACTTTTAGTTCGATCCCCTTAGAGGTTGAAGGGATGATGGCTCATGTGTGAAATCAATTTGACATGGTGTTTAACATATTGGCCGATAAGCCCCTGGTAGGTTGCATTTTTTTTTCTTACCAGCTTCTAAGAATAACGAATCAAATGCCAAACCCACGGGTTTATAGGTGATTTGCTTTGGTGTATCTTTTATTTATTTTTGTTCTTTTAATGGTTTAGCTGTTTGTACGTAGTAGGATAAAAAAGTGGGACATTTTTCTAGGAGTTTATTTGTATGCGTCTGGGGGGATTAGAATAAGAGTAAAAAAAAACTCTTTAAAAGTTTACAAAAAAAGTGGGGTGTTTTTTTTGGAGTTTTTTTTTATGGGTCCGGGAGGATTTTAAGGAGAGGAGAAAAAAAAAACTCTTTAAGAGTTTACAAAAAAAGTGGGGTGTTTTTTTTGGAGTTTTTTTTTATGGGTCCGGGGGGATTTTAAGGAGAGGAGAAAAAAAAAACTCTTCCGGAGTTTAGAATAAAAGTGGGATATTTTTCTAGGAGTTTATTTGTATGCGTCTGGGGGGATTATAATGAGAGTAAAAATAAATCTTTAGGAGTTTAGAATGAGAGAAAAAGTTTTAAAATGGTTCTTCGTATTTGAATTAGTTAAAAATCAATAAAATTTTTATTTAAAGAATTTTTTTTTGTGTTTGTTATTGTAATGGTTCTTATTTATTTAATTGTTATTATGGCATTAGTTAACATCATAGGGACAGCGAGGGAGAGAAAATGTGTTTTAAAAAAGCGGGCTTTAGAGTGGTCTTTGGCTTTATTGTTTAGTACTTTAGTTTTTTGGGGTGGATTTGACGGAGAAAGTCATTTTCAATTTTTTAGTTTAGTAGAATGAAATATATTTTCTACTTTAGATTGAGGCCCGATTGTTTTTGCAGTTGATGGTGTTTCTTTGGTTTTTTTACTTTTAACGACTTTTTTAATTCCAATTTGTATTTTAATTAGCCAAAAATCAATAAAATTCTTATTTAAAGAATTTCTTTTGTGCTTGTTTTTTTTAGAATTTTTATTAATAGGTGTTTTCATAGTGTTTGATCTTCTTTTGTTTTATCTTTTTTTTGAGGGGATATTAATACCAATGTTTTTTTTAATTGGTATTTGAGGCTCTCGAGAAGAAAAGGTTCGTGCTTCTTTTTATTTTTTTTTTTTTACTTTTATAGGCTCTCTCTTTTTCTTTTTTATAATACTTTTTTTATATCAAAGGATTGGAACAACAGACTATTTTCTTTTACTTAATATTAAATTATTTTTAAATATTCAAAAATGAGCCTTAGTCGGGGTTTTTCTTAGTTTTGCGGTGAAACTACCTCTTATTCCATTTCATATTTGATTGCCACAAGCACATGTTGAGGCTCCTGTTGCGGGCTCTGTTATTTTGGCTGGAATTTTATTAAAATTAGGGGGTTATGGCCTTTTGCGTTTTTCTTGACCTCTTTTTCCGGGGGCTTCTTTATATTGGTCTCCAGTTATTGTTTTTTTTAGTGTTGTTGCTGTTGTTTATGGAGGTTTAATGACATGTCGTCAAATTGATTTTAAACGGCTTGTTGCTTACTCTTCTGTTGCTCATATGGGACTTGTGCCTTTGGGTCTTTTTACAAATGTTATAGAGGGGTTAATTGGGGCTCTTTTTTTAATGTTGGCGCACGGATTTGTTAGCTCTGCTCTTTTTATTGGAGTAACTTTTTTGTATGATCGCCATCATACTCGTTTAATAAAATATTATCGGGGTTTGACTTTGACAATGCCTCTTTTTGTTATTACAATGTTAATTTTGTCTTTGGCGAACATGGGTTTTCCTCTTAGTTGTAATTTTGTTGGAGAGTTTTTTTCTTTATTAGCAGTCTTTCAATATCATTATGGAGTTGGAATGTTTGTTATTTTAGGGGTTCTTTTTTCTGCCATTTATTCTCTTAGTCTTTTTAATCGTATTTCTTTTGGTGGAGGCTCTAATTATTTACTTTTTAATAGAGACTTAAGTCGACGAGAGATTTTTGTAATTTTTCCTTTTCTTTTAATTATCTTTTTGGGGGGGATTGTGCCTTTTCCTATTATTGATTTAATTAAAAATAGTCTTGTTTTTAGCCCGGTTAGTTAG-------------TCCTGGGGTTTTAGGGATAAAAAAATATTTAGTCTAGGTTATACATGCTAGTGTATGCGAACAGGTGAGGATAA-AATAAAAAAATTTTTTTTATTTGTTGTATTAAAAAAAGAAAAGTTTTTTA-TTCTTTTTTTGAAGATGCATAGTTTAACCACATTTTCACTGAAACAAAAGAAAACCCTTGGCAGCAGTAATGAATTTTATGTAATATACGAAAGTAAGACATAGGTAGGATAAAAAAAATCTGTCAAAT-TAAGTGCCAGCCGACGCGGTAAGACTTAAGGATTTA-TTTTTTAATAAAAAGCAAAAAGCGTGTTAAGGATTTTTTAAAAAAAAAAATAAATAGAATTTTTTTC--GTAATTGTAATATGCTAAAATGAAAAAAAGAATTTTTTATATGAAGATAATTTA------TTTTTTTTTTCTTAAATACGAAGGTTTGGGGAGCAAATAGGATTAGGTACCCTGGTAGTCTATACAGTAAAATAATATCGCTTGAGTAGTACGGTCGCAAGATTGAAATTCAAAAGACTTGGCTGTTCGGTTGTTAATTAGAGGAGCGCGTCGCTTAATTCGATTATCCGCGAGAGACCTTACCGAAGTTTGAATTTATTAACAGGTGTTGCATGGCCGTCGTCAATTTCTGTTTGAGACAAAAAGGGGTTTAATCCTATCAAGGTTGAAGTCCGGTATTATGACCCTTATATTTCGGGGTTAGGCGCGCTACATTTCTCTCTTAGAAAAGGAGGGGTTCGGATTGTTTTTAAAATAAGATAATGAAGTTGAATTTAATAGTAATTGAAAAGTAGTGCGTTTCAATGAATGTTATTGCAATGTTAGTACAAATAGTCCGTCGCCAGCTTAGAAATAGGCTGTAAGTCGTAACATAGTAAGAGTGAGGGAACTGGCTCTTGATGCAGTTTCATTCGTATCATTTAGTTGAGCCTTCTCCATGGCCTTTTGTTGGAGCAATTGGCTCTTTTTTTATTACTGTTGGTGCAGTGGTTTTTTTTCATTATGGGTTTAGTTTTTTTTTATATTTGGGGCTTTTGGTTGTTGTTGGAGTTATGTTTGTTTGATGACAAGATGTTATACGAGAATCAACTTTTCAAGGTCACCATTCTTTAATTGTAAAACAAGGGATTAAATATGGTATGATTTTATTTATTCTTTCAGAAATTTTGTTTTTTTTTTCTTTTTTTTGGGCTTTTTTTCATAGTAGTTTAGCACCGGTTGTTGAACTTGGAGTCGTTTGGCCTCCACAAGGAATTGTTGCATTAAATCCCTTTTCTGTTCCTTTATTAAATACTGCTGTATTATTAAGTTCTGGGGCAACGGTTACATGGGCGCATCATGCAATACTTTGTGGTTTAAAAAAAGAGGCTCAATTTGCTTTATTTTTAACTCTTTTTTTGGGTGTTATGTTTACGGGGTTGCAAGCATTTGAATATTATGAGGCGCCTTTTACTTTATCTGACTCTGTTTATGGGGCCACTTTTTTTGTTGCGACAGGGTTTCATGGACTACATGTTATAATTGGTACGACTTTTCTTTTTATTTGTTTTTTACGTTTACTCTCTAATCAATTTACTCGTCGTCAACATGTTGGGTTTGAAGCCGCGAGTTGATACTGGCACTTTGTCGATGTGGTTTGATTATTTTTATATTTATGTATTTATTGATGAGGCTCATAAAAATTTCTTTAATGCAAAATCTTTTTGTTTTTACTTTTTTTAGATATTTTATAAATGTTTTGGCTGGGTTGTGTGTTCTCTGGTTTAATCATCTTTATTATTCGGATAGTCCAGAGACTTGACTTTTAGAGTTTCAAGATGTTGGGGATCCTATTGTAGAAGAAATTGTTTTTTTTCATGACCAAGTTATGTTTTTATTAATTATTATAGTTACTGTTGTTTTATGACTTTTTGTTGAAGCTTTTAAAAATAAGTTTTATGATCGTCATTTAATCGATGGTACATTTTTAGAAATTGTTTGAACGATTATTCCTGCTGTTATATTGATTTTTATTGCATTGCCCTCTCTTAAATTATTATATTTAATGGATGAGGTTATTTCTCCAGCTTTGACAATTAAAGTTATTGGGCATCAATGATATTGATCCTATGAATATTCTGATTATGAAGGGGATACGTTAGGGTTTGATTCTTATATGATTCCGACTTCGGATTTAGTTTCTGGAGAGAATCGTTTGTTAGAGGTTGATTATAAACTTTTAATCCCTATTCAAACACATATAAGATTTTTAGTTACTGGGGCAGATGTTTTACATTCTTTTGCAGTGCCTTCTTTAGGGCTAAAAATTGATGCGGTTCCGGGTCGTCTAAATCAAACTGGTGTTTTTATAAAACGAGCCGGGGTTTTTTTTGGACAATGTTCTGAAATTTGTGGGGCAAATCATTCTTTTATGCCCATTGTGATAAAGGGAGTTGGTTTAAATGAATACGTTCAATATTTAAATTATTTAAAATGTATTATAAATACTTAGTTATTATTATTATTTTATTTTTATTGGGGAGTTGGGGTATAATTTTAAATAGAGGACATTTTATTATTATGCTTGTTTCTATTGAATTAGTTTTATTATCAACTTTTTTTTTTTTTTTAATAAGTTCTAAAGAAATAGATCTTTTAATAGAACAAATTTTTATGATTATGGGGTTAACTATTGCTGCTGCCGAATCTTCAATTGGTTTAGCTATTTTAGTTGCATATTATAGAATTCGAGGAACAATTGTTTTAAAATCTTTTAGTTCTTTGCGAGGATAATGATTAATCTTTTTATTTTTTTTTTTCTTATTTTTATTTTGGCGGGGTTATTTATATTTCTTTCTTTTTTGATAGGGGAAAAAATTCCAGGTCGAGAAAAGGTTTCTGCTTATGAGTGTGGTTTTGCTCCCTTTAATTTTTTAGGACGCCCTTTTTCAATACGTTTTTTTTTAATTGGTATTTTATTCTTGATTTTTGATTTAGAAATTTCTTTTTTTTTTCCCTGATGTGTTTTATATAATTCGACTGCTCCGTTTGGGTTTTGAACTATGATAGGTTTTTTTTTTGTATTAGTTTTGGGTTTGATATATGAATGAGTAATGGGGGGATTAGAATGAGAGTAAAAATAAAGTAAAAGAAAAAGTCCTATCTATTATGAAAATAATAGAAATTTTATACCAACTCCGGTTTCTGCTTTAATTCATGCTGCAACGATGGTTACTGCAGGTGTTTTTTTATTAATTCGTGCTTCTCCTTTATTTGATGTTGTTCCTCTTATATTAATTATTATTTCAATCGTTGGGGTTTTAACCGTGTTTATTGCAGGCACAATTGGTTTAGTTCAAAATGATTTAAAAAAAATAATCGCTTATTCTACTTGTAGTCAATTAGGTTATATGGTTGTTGCTTGTGGGCTTTCTCATTATGCAATTAGTCTTTTTCATCTTATGAATCATGCTTTTTTCAAAGCTTTATTATTTTTGAGTGCAGGGTCTCTTATYCATGCGGTAATTGATGAACAAGACATAAGAAAAATGGGGGGTTTATTATCTTTTCTTCCTTTGACTTATGTTTTTTTTCTTATAGGCTCTTTTTCTTTAATGGGGTTTCCTTTTTTAACAGGATTTTATTCAAAAGATTTAATTTTAGAATTTGCTTTTGGACAATTTTATTTAATTTTTGTGTATTGATTAGGTTGTTTTTCTGTTTTATTGACAATTATATACTCTATTCGTTTAATTTATTTAGTTTTTTTATCCAATATTAATTTAAAACGAGCAAACATTTTTTTTCTTAAAGAAGGAGAATTTTTATTTTTAATTCCTTTGGGCATATTAACTTTAGGAAGTGTTTTTTGGGGCTATTTAAGTAAAGAAATAATTTGGTCTTTTCAAATAGATGTTTTTTCAATACTTTCTTTAAAAATAAAAATATTTCCAATTTTATTTTGTTTTATTGGACTTTTTGGGACGATATTTTTTTTTTTTTTTTTTTCTTCTCAAATTTTTGGTTATCCCCTTCAGGTGATCGGATCTTCTGTTTTTTCTCTTTATAATTTTTTTGGTTCTGCTTGACAAATAAATTTTTTTTTTAATTTTTTCTTTATAAAAAAAATATATAAAATAGGACATCTTATTACTAATTTAACAATTGACAAAGGTTTATTAGAGGTTGTTGGGCCTAGGGGTGTTGTTCAATTTTTTATTTTTCAAACTCAAAAGTTGAGTAGTATACAATCGGGGTTGGTATTTAATTATGCTTTAGTTTTTTTTCTTGGGATACTTTTTTTAATTTTTGCACTTTAATTAAAATTTTTGAAGGGGTTAAGTTAAGCAAAACAGTTGACTTTCAAGGTTAATTATGTAGGTGGAAATCTTACACTCTTTGGATGCCACAGTTAGAGGTAGGTACTTATAATATACAATATTGGTGGGGGTTCTCGGTTTTATTTTTCTTGTTAATTTTTTTAGAAGTTATAATCTTTCCTTTTGTAAAGCGTGGTTGATGAATAAGACAGTTCTTATTGGGGTG---------------AACCTTTTTTTTAGGGTGAAAAAAAAATTATGGAAAAAGATTTCCATGAGAACTGTCGCAAAGTGTTTAAAAAAAGCCTTT-TTTTTGCT--AGTGGGATTTTGTTTAAAATATTTTT-TTGAGTTTTCTTACTTTAAAACATCTTTTTGTTTAATGA-TTTTTTTTTTTTTTAT-------------------------------------AAAATTTGGT------------TCTAAGAAGATTC----------------------------------------------GGGCTCGTTTAGCGATCTTTTC---ACCGTTGATC-TTT---GGGGATGGTGTGGA--AAAAATGGAAGTAGATGAG-CCAGATGA--AGAGATAGTAGGAG--CGTTTCCATCTTTA--------AGCTTCGTGGAGGGGTGAGATG----------------------------AAGAGGTGATAGCAGCGTTTCCATGTTCAGACT----------CCGAAGAGAGTATGGAAATTGATGGGGAAGA--ACTACCTTTGGAGCCCCTCCCGATTAGTGGAGCT--ATTAGAGGAGAA--AACATAGTGGYA-------------------------------CCCGTACCAGTCATGGGAGATGCGGGTCTTTCAGTTCATTTTTGGCGTGCACTCAAGAACACTCCATTTGAATTAT-TGGCAGAAGTTTATCGGTCAGGTGAGGAGGGAGAAG--TT---T--RTTCGTCAGATGAG---GAAGACGAGTTAGTAGAGGTGTTACTTCAGTACGGAAA-AGAGAT---------------------AACATCAAAAGAGTCAGAGGACAAATTTTTGAGTGGGCTAAT--AGT--GGAAATGGTGGAAAACGTAATTGCCT-AAGATAAATACCA-GAA-CAGTT-AGTAAACAGATTATTGAGTGGGTCAGTGGA--CAAAGTAGGGGATAACGTGGTTGGGTAAAAT-ATAGTGTCGGCGGCAACGGAAGTGGGGAGGGGGAGAGCTCTCTTGCTCTCTAATTTGTAGTTTTTGAGTGGTTTGTATTATTTTTT--TTTTTAATTTTTTCTTTATGAGTCTGTCTTGGTAGAGGAG-GCGGTTTTTTTAGAAGCCCCGTCTAAATGCTTTCTGGTTATTGAACTCGATGGGTGTTTTCTACAAATCATAAGGACATTGGTACTTTGTATTTAATCTTCGGAGCAGGAGCTGGTTTAATTGGAACTGCTTTTAGTATGCTTATACGATTGGAGCTTTCTGCGCCGGGGGCGATGTTAGGAGATGATCATCTTTATAATGTAATTGTTACAGCACATGCTTTTATTATGATTTTTTTTTTGGTTATGCCGGTCATGATTGGGGGATTTGGTAATTGATTAGTCCCATTATATATTGGGGCGCCGGATATGGCGTTTCCTCGATTAAACAATATTAGTTTTTGACTTTTGCCTCCTGCGCTTTTTTTATTATTAGGCTCTGCTTTTATTGAACAAGGGGCGGGGACGGGGTGAACAGTTTATCCTCCTCTTTCTAGTATTCAAGCACACTCCGGAGGTTCTGTTGATATGGTTATTTTTAGTCTTCATTTAGCTGGGGTTTCTTCTATTTTAGGTGCTATTAACTTTATTACTACAATTTTTAATATGCGAGCCCCGGGTGTGTCTTTTAATAAACTACCTTTATTTGTTTGATCTATTTTAATAACAGCTTTTTTATTACTTTTATCTTTACCTGTTTTAGCTGGTGCTATTACTATGTTGTTAACAGATAGAAACTTTAATACGACTTTTTTCGATCCAGCGGGTGGCGGGGACCCAATATTATTTCAGCATTTATTTTGATTCTTTGGGCATCCAGAAGTTTATATTTTAATTTTGCCTGGTTTTGGTATGATTTCTCAAATAATCCCGACTTTTGTTGCTAAAAAACAAGTTTTTGGGTATTTAGGAATGGTTTATGCCATGCTTTCTATTGGGCTTCTTGGATTTATTGTTTGAGCTCATCATATGTTTACTGTTGGGATGGATGTAGATACAAGAGCATATTTTACTGCTGCTACTATGATTATTGCTGTGCCAACTGGGATTAAAGTTTTTAGTTGGTTGGCAACTATTTATGGAGGTGTTCTTAGGTTAGAGACTCCAATGCTTTGAGCTATGGGGTTTGTTTTTTTATTTACAGYTGGTGGTTTAACTGGGGTTGTGTTAGCAAATAGTTCTCTTGATATTGTTCTACATGATACATATTATGTAGTTGCGCATTTTCATTATGTTCTTTCTATGGGGGCTGTTTTTGCTATTTTTGGGGGGTTTTACTATTGAATTGGCAAAATAAGTGGTTATTGTTATAATGAATTTTTTGGGAAAGTTCATTTTTGATTAATGTTTATCGGGGTTAATCTAACTTTTTTCCCTCAACATTTTTTAGGTTTAGCAGGATTTCCAAGACGATATTCGGATTATGCAGATGCTTTTTTGGGTTGAAATTTAATAAGTTCTTTAGGGTCTATTATTTCTATTTTGAGTGTTGTTTGGTTTTTATATATTGTTTTTGATCTTTTTGTTACAGAAGAAAAATTTTTGGGTTGAAAAGAAGGATTTTCTTTAGAATGAATTCATTCTTCTCCCCCCTTATTTCATACTTATGAGGAGTTGCCCTTTGTACAAAAAGTAAATAATTTTTAGAATAGTGCCGGGTTTATATACCGGTTT
[truncated: 35,784 more chars]
